# Supplementary material for: Direct catalytic asymmetric synthesis of α-chiral bicyclo[1.1.1]pentanes
Source: Nat Commun. 2021 Mar 12;12:1644. doi: 10.1038/s41467-021-21936-4 (PMC7955048; doi:10.1038/s41467-021-21936-4)
Supplement: Supplementary file 1 — Supplementary Information [file 41467_2021_21936_MOESM1_ESM.pdf]

Direct Catalytic Asymmetric Synthesis of α-Chiral Bicyclo[1.1.1]pentanes

Marie L. J. Wong,[a] Alistair J. Sterling,[a] James J. Mousseau,[b] Fernanda Duarte[a] and Edward A. Anderson[a*]

[a]Chemistry Research Laboratory, 12 Mansfield Road, Oxford, OX1 3TA, UK
[b]Pfizer Medicine Design, Eastern Point Road, Groton, Connecticut 06340, USA

Supplementary Information

Contents

1. General experimental considerations **1**

2. Optimization studies **4**

2.1 Reduction conditions, light source and reaction temperature 4

2.2 H-atom transfer catalysts 5

2.3 Organocatalysts 7

2.4 Photocatalysts 8

2.5 Solvents 9

2.6 Equivalents of reactants/reagents and reaction time 10

2.7 Control experiments 11

2.8 Optimized reaction conditions 11

3. Experimental procedures **12**

3.1 General experimental procedures 12

3.2 Characterization of compounds 15

3.2.1 Tricyclo[1.1.1.0^{1,3}]pentane, **1** 15

3.2.2 BCP products 16

3.2.3 HAT catalysts 63

3.2.4 Organocatalysts 79

3.2.5 Aldehyde substrates 95

3.2.6 Unsuccessful substrates 102

4. Computational data **103**

4.1 QM calculations on a model system 103

4.2 Model radical system 105

4.2.1 Methyl substituent 105

4.2.2 Moc substituent 105

4.3 Calculations on the full system 107

4.3.1 Methodology 107

4.3.2 Enamine *E* / *Z* equilibria 110

4.3.3 Transition state models 112

4.3.3.1 Moc substituent (**TS-8’**) 112

4.3.3.2 Moc substituent, diphenylprolinol catalyst (**TS-8’-H**) 112

4.3.3.3 Methyl substituent (**TS-12**) 113

4.3.3.4 Hexyl substituent (**TS-15**) 113
4.3.3.5 NCI plots 118
4.4 Energetics and thermodynamic quantities 119
5. Photophysical experiments 124
5.1 Stern-Volmer quenching experiments 124
5.2 UV/Vis absorption experiments 128
6. NMR spectra 129
7. Supplementary references 178

1. General experimental considerations

NMR Spectroscopy: Proton (^{1}H), carbon (^{13}C) and fluorine (^{19}F) NMR spectra were recorded on a Bruker AVIII HD 400, NEO 400, AVIII HD 500, AVII 500 or AVIII HD 600 spectrometer. Proton, carbon and fluorine chemical shifts (δ) are quoted in parts per million (ppm) to the nearest 0.01 ppm for ^{1}H and ^{19}F NMR spectra, and 0.1 ppm for ^{13}C spectra. ^{1}H NMR spectra were recorded using an internal deuterium lock for the residual protons in $CDCl_3$ (δ = 7.26) or benzene-d_6 (δ = 7.16). ^{13}C NMR spectra were recorded using an internal deuterium lock in $CDCl_3$ (δ = 77.16) or benzene-d_6 (δ = 128.06). ^{19}F NMR spectra were not externally referenced. Assignments were determined either on the basis of unambiguous chemical shift or coupling patterns, COSY, HSQC, HMBC and/or NOESY experiments. Peak multiplicities are defined as s (singlet), d (doublet), t (triplet), q (quartet), quin (quintet), sept (septet), m (multiplet), br (broad) and app (apparent). Coupling constants (*J*) are reported to the nearest 0.1 Hz. We would like to thank Jos J. A. G. Kamps, Jonathan Yong and the Departmental NMR Service, University of Oxford for assistance in acquiring NMR spectra.

Mass Spectrometry: Low-resolution mass spectra were recorded on a Micromass LCT Premier Open Access using electrospray ionization (ESI). High-resolution mass spectra (HRMS) were recorded by the Departmental Mass Spectrometry Service, University of Oxford on a Thermo Scientific Exactive Mass Spectrometer (using a Waters Equity autosampler and pump) for electrospray ionization (ESI) and an Agilent 7200 Accurate Mass QTOF GCMS (using a SIM Direct Insertion Probe) for electron ionization (EI) and chemical ionization (CI). High-resolution values are calculated to 4 decimal places from the molecular formula, and all values are within a tolerance of 5 ppm.

Infrared Spectroscopy: Infrared spectra were obtained in $CHCl_3$ as solvent on a Bruker Tensor 27 FT-IR spectrometer. The sample was prepared as a thin film on a diamond ATR module. Wavelengths of maximum absorbance (ν_{max}) are quoted in cm^{-1}. Only selected, characteristic IR absorption data are provided for each compound.

Melting Points: Melting points were obtained using a Griffin melting point apparatus and are uncorrected.

Polarimetry: Optical rotations were recorded on a Perkin Elmer 241 or 341 polarimeter with a path length of 1 dm (using the sodium D line, 589 nm). $[\alpha]_D$ are reported in units of 10^{-1} deg cm^2 g^{-1}. Concentrations are reported in g/100 mL. Temperatures are reported in °C.

Chromatography: Column chromatography refers to normal phase column chromatography and was performed on silica gel obtained from Merck (Silica gel Si 60, 0.040-0.063 mm) under a positive pressure of nitrogen, using the stated solvent system. Analytical thin-layer chromatography was used to monitor reaction progress and performed on pre-coated aluminium-backed plates (Merck Kieselgel 60 F_{254} plates) with visualization by ultraviolet light (254 nm) and/or by staining with vanillin, phosphomolybdic acid and potassium permanganate. Retention factors (R_f) are reported with the solvent system in parentheses. High performance liquid chromatography (HPLC) for enantiomeric excess (*ee*) determination was carried out using a Phenomenex Lux i-Amylose-1 (250 mm x 4.6 mm ID) or DAICEL CHIRALPAK IB or IC (250 mm x 4.6 mm) column (wavelength: 210 nm or 250 nm) with purified material. Chiral supercritical fluid chromatography (SFC) separations were conducted on a Waters Acquity UPC system using the Empower software with a CHIRALPAK ID (250 mm x 4.6 mm) column.

Materials/procedures: All air- or moisture-sensitive reactions were carried out with anhydrous solvents in flame-dried glassware under an inert atmosphere of argon. Light-sensitive reactions were carried out under aluminium foil protection. Heating was performed using an oil or sand bath. Anhydrous tetrahydrofuran (THF), dichloromethane (CH_2Cl_2), diethyl ether (Et_2O), dimethoxyethane (DME), triethylamine (Et_3N), chloroform ($CHCl_3$), pyridine, toluene (PhMe), *N*,*N*-dimethylformamide (DMF), acetonitrile (MeCN) and 1,4-dioxane were collected fresh from an mBraun SPS-800 solvent purification system, having been passed through anhydrous alumina columns. All other commercially available reagents and solvents, where appropriate, were dried and purified before use using standard procedures.

Photochemical equipment and setup: Three LED lamps were used:

- Tingkam® Waterproof 5M 5050 SMD RGB LED Strips fixed to the inside of a crystallization dish.
- Evoluchem™ 18 W LED (405 nm). The vial was positioned on a stir plate approximately 5 cm away from the LED and under a strong flow of nitrogen to dissipate the heat generated from the LED.
- Evoluchem™ 18 W LED (450 – 455 nm). For reactions run at room temperature, the vial was positioned on a stir plate approximately 5 cm away from the LED and under a strong flow of nitrogen to dissipate the heat generated from the LED. For reactions run at lower temperatures (0 °C, 10 °C and 20 °C), the vial was positioned on a stir plate approximately 5 cm away from the LED and submerged in a cooling bath (isopropanol) in a large crystallization dish with a Huber TC50E immersion cooler.

2. Optimization studies

2.1 Reduction conditions, light source and reaction temperature (Supplementary Table 1)

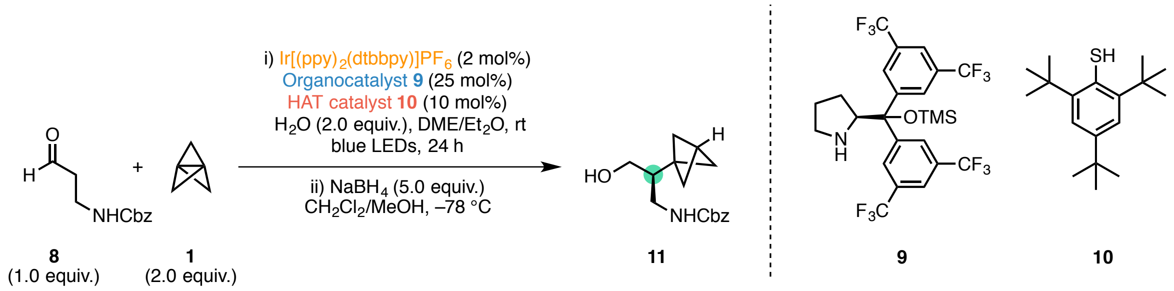

	Entry	Variation of conditions	Yield[a]	ee
reduction	1[b]	$NaBH_4$ (2.0 equiv.)	14%	88%
	2[b]	$NaBH_4$ (5.0 equiv.)	31%	89%
light source	3	18 W LED (405 nm)	45%	70%
	4	18 W LED (450 – 455 nm)	57%	77%
temperature	5	20 °C	45%	80%
	6	10 °C	65%	86%
	7	0 °C	63%	82%

[a]Isolated yields; 0.25 mmol scale; DME:Et_2O (2:1). [b]Blue LED strips in a crystallization dish.

2.2 H-atom transfer catalysts (Supplementary Table 2)

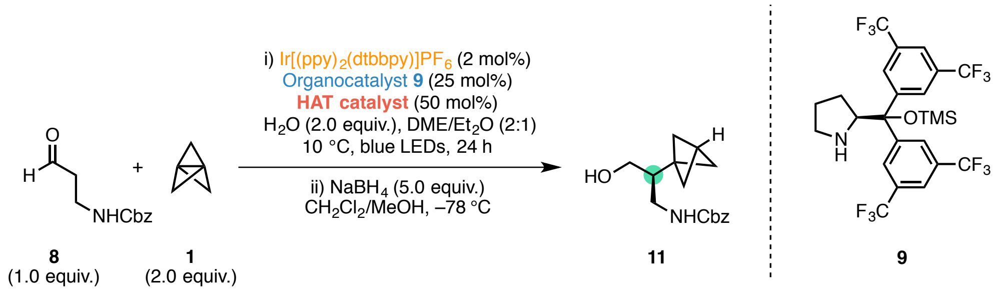

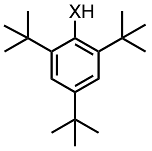

10 X = S 65% 86% *ee*[a]
10 X = S 66% 83% *ee*
S1 X = Se 17% 95% *ee*

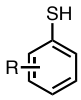

S2 R = H 17% 72% *ee*
S3 R = 2,4,6-Cy 38% 85% *ee*
S4 R = 2,4,6-*c*-Pent 45% 93% *ee*
S5 R = 2,4,6-^{i}Pr 47% 85% *ee*
S6 R = 2,6-^{i}Pr-4-OMe 42% 83% *ee*
S7 R = 3,5-^{t}Bu 25% 83% *ee*

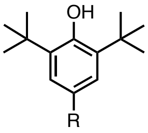

S8 R = Me 39% 90% *ee*
S9 R = NO_2 30% 86% *ee*

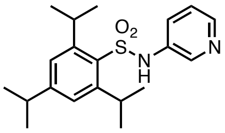

S10 10% 37% *ee*

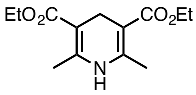

S11 16% 66% *ee*
S11 26% 24% *ee*[b]

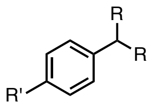

S12 R = Ph, R' = H 24% 77% *ee*
S13 R = CN, R' = H 30% 94% *ee*
S14 R = CN, R' = CN 16% 78% *ee*
S15 R = CN, R' = NO_2 n.r.
S16 R = CN, R' = OMe 20% 95% *ee*

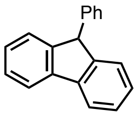

S17 27% 79% *ee*

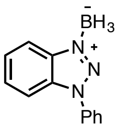

S18 10%[c]

$PhSO_2Na$, **S19**
n.r.
n.r.[d]

HOBt, **S20**
21% 75% *ee*

Phthalimide, **S21**
17% 75% *ee*

N-Hydroxyphthalimide, **S22**
22% 72% *ee*

Ph_3SiH, **S23**
21% 75% *ee*

TTMSS, **S24**
17% 75% *ee*

^{i}Pr$_3$SiSH, **S25**
22% 72% *ee*

Et_3GeH, **S26**
27% 72% *ee*

HCO_2H, **S27**
n.r.[e]

Isolated yields; 0.25 mmol scale. [a]**10** (10 mol%). [b]Bu_3N (50 mol%). [c]ee not determined. [d]NaOAc (50 mol%). [e]**S27** (2.0 equiv.), Bu_3N (2.0 equiv.). n.r.: no reaction.

Thiols are known to react efficiently with [1.1.1]propellane **1**[1] and the reaction between thiol HAT catalyst **10** and [1.1.1]propellane **1** was investigated as the presence of thiyl radicals generated in the asymmetric reaction could lead to undesirable side reactions (Supplementary Figure 1). Thiol **10** (1.0 equiv.) and [1.1.1]propellane **1** (1.0 equiv.) were left to stir in the dark (aluminium foil protection) or under blue LED irradiation for 24 h (the duration of the asymmetric reaction). It was found that the thiol-BCP adduct **S28** was obtained in roughly the same yield under both conditions, along with rearrangement product **S29** and 1,3,5-tri-*tert*-butylbenzene **S30**. However, none of these products were observed in the asymmetric ring-opening of [1.1.1]propellane **1**, indicating the efficiency of thiol **10** as a HAT catalyst.

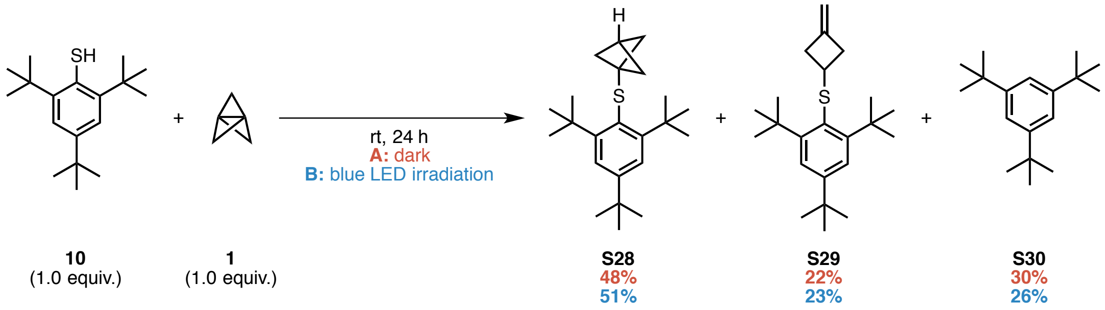

Supplementary Figure 1. *Reaction between HAT catalyst* ***10*** *and [1.1.1]propellane* ***1****.*

2.3 Organocatalysts (Supplementary Table 3)

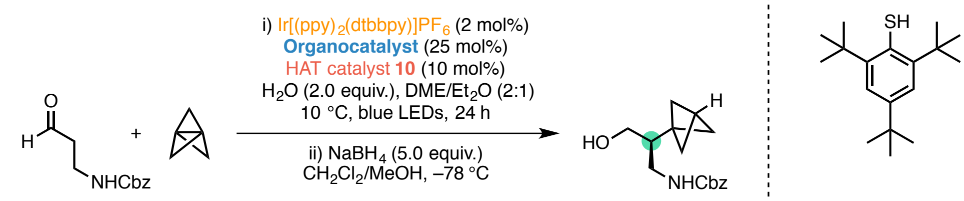

8 (1.0 equiv.) **1** (2.0 equiv.) **11** **10**

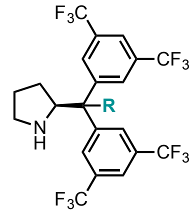

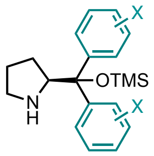

**S31** R = H	52%	12% *ee*	
**S32** R = OH	27%	23% *ee*	*E*° = +0.52 V[a]
**9** R = OTMS	65%	86% *ee*	*E*° = +0.77 V[a]
**S33** R = OTES	57%	80% *ee*	
**S34** R = OTBS	22%	76% *ee*	

**S35** Ar = Ph	32%	44% *ee*	*E*° = +0.56 V[a]
**9** Ar = 3,5-CF_3	65%	86% *ee*	*E*° = +0.77 V[a]
**S36** Ar = 3,5-Me	33%	44% *ee*	
**S37** Ar = 2-naph	51%	49% *ee*	

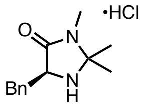

S38 22% 89% *ee*
E° = +0.85 V[a]

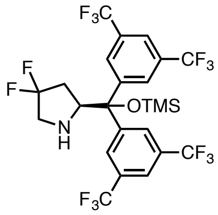

S39 n.r.

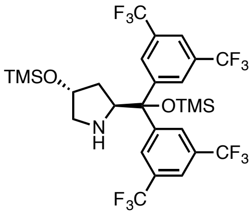

S40 16% 77% *ee*

Isolated yields; 0.25 mmol scale. [a]Reduction potentials of enamine formed from condensation of propionaldehyde and organocatalyst (*E*°[α-iminyl radical cation/enamine] vs. SCE).[2]

2.4 Photocatalysts (Supplementary Table 4)

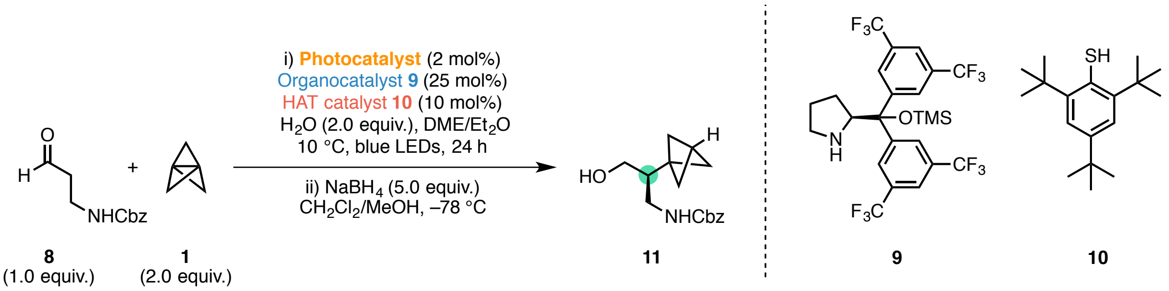

Entry	Photocatalyst	$E°$[a]	Yield[b]	ee
1	$Ir[(ppy)_2(dtbbpy)]PF_6$	+ 0.66 V[3]	65%	86%
2	$Ir[(dF(CF_3)ppy)_2(dtbbpy)]PF_6$	+ 1.21 V[4]	38%	60%
3	$Ir[(dF(CF_3)ppy)_2(bpy)]PF_6$	+ 0.97 V[4]	36%	33%
4	$Ir[(dF(CF_3)ppy)_2(dCF_3bpy)]PF_6$	+ 1.68 V[5]	28%	67%
5	$Ir[(Me(Me)ppy)_2(dtbbpy)]PF_6$	+ 0.55 V[4]	53%	81%
6	*fac*-$Ir(ppy)_3$	+ 0.31 V[4]	n.r.	–
7	$Ir(dFppy)_3$	+ 0.36 V[4]	n.r.	–
8	$Ir(4'\text{-}Fppy)_3$	+ 0.69 V[4]	n.r.	–
9	$Ru(bpy)_3(PF_6)_2$	+ 0.77 V[4]	n.r.	–
10	Mes-Acr-Me^+ ClO_4^-	+ 2.08 V[6]	n.r.	–
11	4CzIPN	+ 1.35 V[7]	n.r.	–
12[c]	$Ir[(ppy)_2(dtbbpy)]PF_6$	+ 0.66 V[3]	61%	92%
13[c]	$Ir[(Me(Me)ppy)_2(dtbbpy)]PF_6$	+ 0.55 V[4]	56%	94%

[a]Reduction potential of excited-state photocatalyst ($E°$ vs. SCE). [b]Isolated yields; 0.15 mmol scale; DME:Et_2O (2:1). [c]Octanal (1.0 equiv.) instead of **8**; 0.20 mmol scale.
n.r.: no reaction.

2.5 Solvents (Supplementary Table 5)

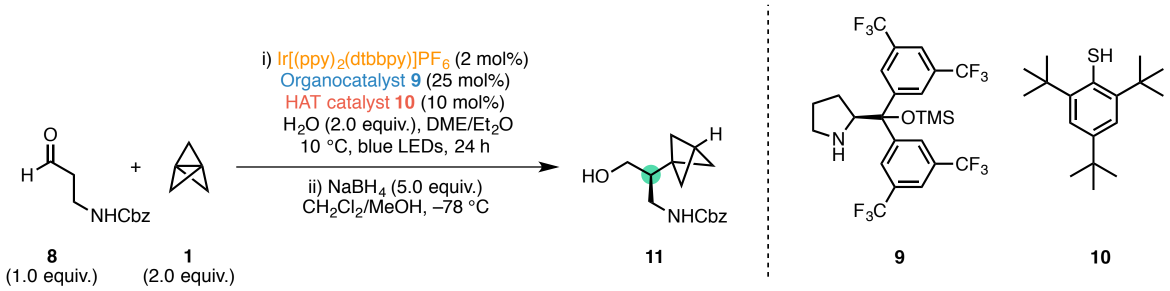

	Entry	Variation of conditions	Yield[a]	ee
co-solvent[b]	1	DME	65%	86%
	2	DCM	62%	73%
	3	MeCN	n.r.	–
	4	1,4-dioxane	46%	86%
	5	Et_2O	63%	90%
	6	THF	19%	80%
	7	MeOH	13%	69%
conc.[c]	8	0.12 M	40%	89%
	9	0.20 M	65%	86%
	10	0.25 M	38%	78%
solvent ratio[d]	11	DME : Et_2O (0.5 : 1.0)	59%	88%
	12	DME : Et_2O (1.0 : 1.0)	65%	86%
	13	DME : Et_2O (1.6 : 1.0)	66%	85%

[a]Isolated yields; 0.15 mmol scale. [b]2:1 ratio of co-solvent to Et_2O from [1.1.1]propellane **1** solution; reaction concentration: 0.20 M. [c]2:1 ratio of DME to Et_2O from [1.1.1]propellane **1** solution. [d]Reaction concentration: 0.20 M.
n.r.: no reaction.

2.6 Equivalents of reactants/reagents and reaction time (Supplementary Table 6)

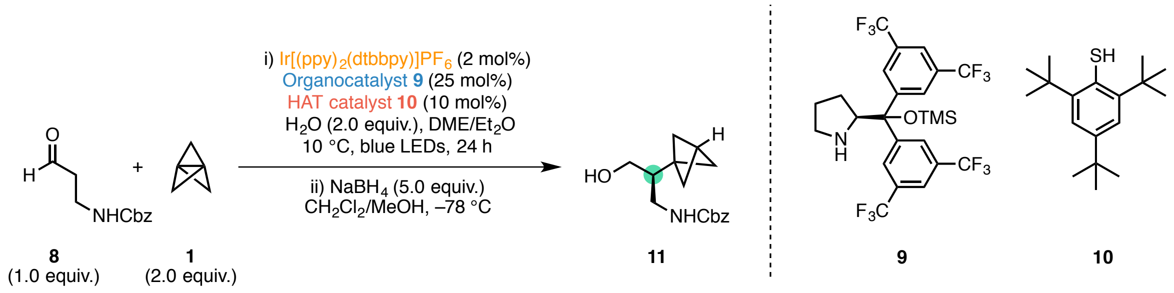

	Entry	Variation of conditions	Yield[a]	ee
HAT cat.	1	**10** (10 mol%)	65%	86%
	2	**10** (30 mol%)	70%	82%
	3[b]	**10** (50 mol%)	66%	83%
organocat.	4	**9** (20 mol%)	50%	85%
	5	**9** (25 mol%)	65%	86%
	6	**9** (30 mol%)	57%	89%
photocat.	7	Ir[(ppy)$_2$(dtbbpy)]PF$_6$ (1.0 mol%)	50%	82%
	8	Ir[(ppy)$_2$(dtbbpy)]PF$_6$ (2.0 mol%)	65%	86%
	9	Ir[(ppy)$_2$(dtbbpy)]PF$_6$ (2.5 mol%)	65%	85%
H_2O	10	H_2O (1.0 equiv.)	62%	88%
	11	H_2O (2.0 equiv.)	65%	86%
	12	H_2O (3.0 equiv.)	62%	83%
[1.1.1]propellane **1**	13	**1** (2.0 equiv.)	65%	86%
	14	**1** (1.5 equiv.)	69%	87%
	15	**1** (1.3 equiv.)	71%	86%
	16	**1** (1.1 equiv.)	71%	87%
	17[c]	**1** (1.3 equiv.)	67%	89%
	18[c]	**1** (1.1 equiv.)	68%	90%
reaction time	19[c]	17 h	61%	92%
	20[c]	24 h	68%	90%
	21[c]	46 h	68%	90%
aldehyde	22[d]	octanal (1.3 equiv.)	88%	95%
	23[d]	octanal (1.5 equiv.)	96%	97%
	24[d]	**8** (1.5 equiv.)	94%	88%

[a]Isolated yields; 0.15 mmol scale; DME:Et$_2$O (1:1). [b]0.25 mmol scale. [c]Octanal (1.0 equiv.) instead of **8**; 0.20 mmol scale. [d][1.1.1]Propellane **1** (1.0 equiv.); 0.20 mmol scale.

2.7 Control experiments (Supplementary Table 7)

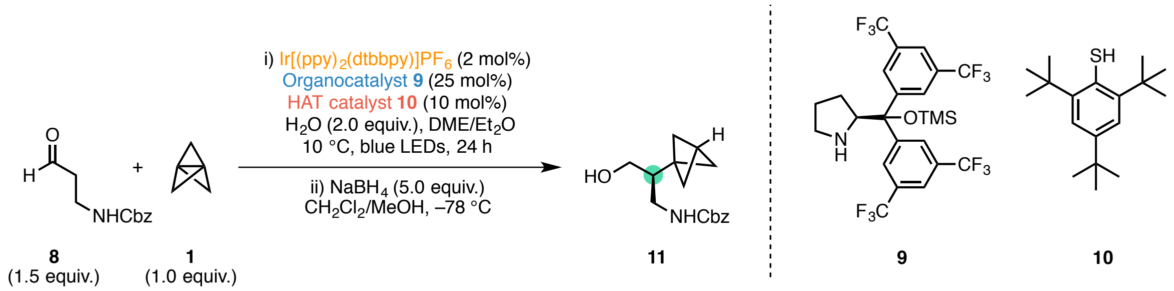

Entry	Variation of conditions	Yield[a]	ee
1	no photocatalyst	n.r.	–
2	no organocatalyst	n.r.	–
3	no HAT catalyst	trace	–
4	no light irradiation	n.r.	–

[a]Isolated yields; 0.15 mmol scale; DME:Et_2O (1:1). n.r.: no reaction.

2.8 Optimized reaction conditions

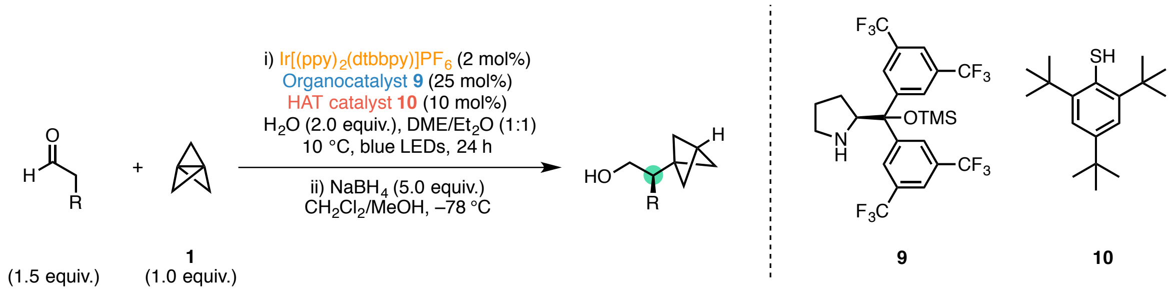

Supplementary Figure 2. *Optimized reaction conditions for the asymmetric ring opening of [1.1.1]propellane* ***1****.*

See General Procedure A (p. 12) for details of the optimized experimental procedure.

3. Experimental procedures

3.1 General experimental procedures

General procedure A: Asymmetric ring opening of [1.1.1]propellane

To a flame-dried vial was added Ir[(ppy)$_2$(dtbbpy)]PF$_6$ (0.02 equiv.), 2,4,6-tri-*tert*-butylbenzenethiol **10** (0.1 equiv.), (*S*)-α,α-bis[3,5-bis(trifluoromethyl)phenyl]-2-pyrrolidinemethanol trimethylsilyl ether **9** (0.25 equiv.) and aldehyde (1.5 equiv., if solid). The vial was sealed with a rubber septum and Et_2O, DME and distilled water (2.0 equiv.) were then added (taking into account Et_2O from the [1.1.1]propellane (tricyclo[1.1.1.0^{1,3}]pentane, TCP) **1** solution, final reaction concentration = 0.2 M with a 1:1 ratio of Et_2O to DME). The vial was then cooled in an ice bath (0 °C) and the solution was degassed by sparging with argon for 15 minutes. The vent needle was removed and TCP **1** (1.0 equiv.) and aldehyde (1.5 equiv., if liquid) were then added. The vial was then capped and double-sealed with parafilm. The reaction mixture was then placed in a cooling bath at 10 °C and irradiated with blue LEDs for 24 h. The reaction mixture was then concentrated *in vacuo*. MeOH (5 mL/mmol of TCP **1**) and CH_2Cl_2 (5 mL/mmol of TCP **1**) were added to the vial and the reaction mixture cooled to –78 °C. Sodium borohydride (5.0 equiv.) was added and the reaction mixture was left to stir for 30 min to 1 h at –78 °C. The reaction mixture was quenched by addition of NH_4Cl (aq., sat., 5 mL/mmol of TCP **1**) and water (5 mL/mmol of TCP **1**). The phases were separated and the aqueous phase was extracted with CH_2Cl_2 (× 3). The combined organic extracts were dried (Na_2SO_4) and concentrated *in vacuo*.

General procedure B: DMAP/EDCI coupling (for HPLC analysis of BCP products)

To a solution of alcohol (1.0 equiv.) and carboxylic acid (1.0 equiv.) in CH_2Cl_2 (10 mL/mmol of alcohol) at room temperature under an inert atmosphere was added 4-(dimethylamino)pyridine (DMAP) (3.0 equiv.) and *N*-ethyl-*N′*-(3-dimethylaminopropyl)carbodiimide hydrochloride (EDCI) (3.0 equiv.). The reaction mixture was left to stir for the stated time. Upon completion, water (20 mL/mmol of alcohol) was added. The phases were separated and the aqueous phase was extracted with CH_2Cl_2 (× 3). The combined organic extracts were dried (Na_2SO_4) and concentrated *in vacuo*.

General procedure C: Benzenethiol and benzeneselenol synthesis

Adapted from the procedure of Ephritikhine et al.[8] To a suspension of magnesium turnings (2.0 equiv.) in THF (2 mL/mmol of aryl bromide) at room temperature under an inert atmosphere was added aryl bromide (1.0 equiv.). A crystal of iodine was then added and the reaction mixture heated to 80 °C in a sealed flask. After 15 h, the reaction mixture was cooled to room temperature and sulfur or selenium (1.05 equiv.) was added. The reaction mixture was then heated to 80 °C. After 1.5 h, the reaction mixture was cooled to room temperature and lithium aluminium hydride (1 M in THF, 0.5 equiv.) was added dropwise. The reaction mixture was heated to 80 °C for 30 min and then cooled to 0 °C in an ice bath. The reaction was quenched at 0 °C by addition of water (2 mL/mmol of aryl bromide) dropwise. HCl (1 N, ~2 mL/mmol of aryl bromide) was then added until good phase separation was observed. The phases were separated and the aqueous phase was extracted with Et_2O (× 3). The combined organic extracts were washed with brine, dried (Na_2SO_4) and concentrated *in vacuo*.

General procedure D: 2-Arylmalononitrile synthesis

Adapted from the procedure of Hayashi et al.[9] To a solution of sodium hydride (60% dispersion in mineral oil, 3.0 equiv.) in THF (1 mL/mmol of aryl halide) at 0 °C under an inert atmosphere was added a solution of malononitrile (2.0 equiv.) in THF (1 mL/mmol of aryl halide) dropwise. After completion of addition, the reaction mixture was allowed to warm to room temperature and, after the evolution of gas ceased, $Pd(PPh_3)Cl_2$ (0.03 equiv.) and aryl halide (1.0 equiv.) were added. The resulting mixture was stirred at 70 °C for 24 h. The reaction mixture was then cooled to room temperature, quenched by the addition of HCl (aq., 2 M, 2 mL/mmol of aryl halide). The phases were separated and the aqueous phase was extracted with Et_2O (× 3). The combined organic extracts were dried (Na_2SO_4), filtered and concentrated *in vacuo*.

General procedure E: Borane dimethyl sulfide complex reduction

Adapted from the procedure of Anderson et al.[10] To a solution of carboxylic acid (1.0 equiv.) in THF (5 mL/mmol of acid) at 0 °C under an inert atmosphere was added borane dimethyl sulfide complex (2 M in THF, 1.1 equiv.) dropwise. The reaction mixture was allowed to warm to room temperature and left to stir for the stated time. Upon completion, MeOH (0.5 mL/mmol of acid) was added and the resulting mixture was concentrated *in vacuo*. CH_2Cl_2 (10 mL/mmol of acid) was added and the organic phase was

washed with $NaHCO_3$ (aq., sat., 5 mL/mmol of acid), HCl (aq., 2 M, 5 mL/mmol of acid) and brine (5 mL/mmol of acid). The organic phase was dried (Na_2SO_4) and concentrated *in vacuo* to afford the corresponding alcohol, which was used without further purification.

General procedure F: TEMPO/BAIB oxidation

To a solution of alcohol (1.0 equiv.) in CH_2Cl_2 (5 mL/mmol of alcohol) at room temperature was added 2,2,6,6-tetramethyl-1-piperidinyloxy, free radical (TEMPO) (0.2 equiv.) and bis(acetoxy)iodobenzene (BAIB) (1.8 equiv. or 2.5 equiv.). The reaction mixture was stirred at room temperature for the stated time. Upon completion, water (3 mL/mmol of alcohol), $Na_2S_2O_3$ (aq., sat., 3 mL/mmol of alcohol) and $NaHCO_3$ (aq., sat., 3 mL/mmol of alcohol) were added. The phases were separated and the aqueous phase was extracted with Et_2O (× 3). The combined organic extracts were dried (Na_2SO_4) and concentrated *in vacuo*.

3.2 Characterization of compounds

3.2.1 Tricyclo[1.1.1.0^{1,3}]pentane (TCP), 1

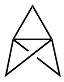

According to the procedure of Baran et al.[11] To a solution of 1,1-dibromo-2,2-bis(chloromethyl)cyclopropane (28.2 g, 95.0 mmol, 1.0 equiv.) in Et_2O (60 mL) under an inert atmosphere at –40 °C was added phenyllithium (1.9 M in dibutyl ether, 100 mL, 190 mmol, 2.0 equiv.) via cannula over 30 min. The resulting yellow mixture was then stirred at 0 °C for 2 h and distilled in a rotary evaporator at room temperature, having both the condenser and collection flask of the rotary evaporator cooled to –78 °C. The title compound was collected as a clear, colorless solution (0.62 M in Et_2O, 85.0 mL, 56%) and used in subsequent reactions as a solution in Et_2O. The yield and approximate concentration of the solution was calculated using quantitative NMR with 1,2-dichloroethane as an internal standard. 0.2 mL of the solution of TCP **1** in Et_2O was diluted with 1,2-dichloroethane (50 µL) and $CDCl_3$ (0.5 mL). The ratio of 1,2-dichloroethane (4H) to TCP **1** (6H) was determined and used to calculate the approximate concentration of the solution. Two runs were carried out and the average used as the final approximated concentration.

^{1}H NMR (200 MHz, $CDCl_3$) δ 1.94 (6H, s).
Spectroscopic data in agreement with that reported previously.[12]

3.2.2 BCP products

Absolute stereochemistry of the BCP product **12** was determined by comparison of SFC traces (after derivatization to (*R*)-2-(bicyclo[1.1.1]pentan-1-yl)propyl 2-naphthoate) to that of its enantiomer synthesized in a previous publication.[13] Absolute stereochemistry of the remaining BCP compounds were assigned by analogy. Racemic BCP products for *ee* determination were synthesized following General Procedure A using racemic organocatalyst *rac*-**9**. Compounds that lacked UV active chromophores were further derivatized to enable HPLC analysis.

SFC Traces: CHIRALPAK ID, 1 – 30% MeOH/CO_2 gradient over 5 min, 1.5 mL/min.

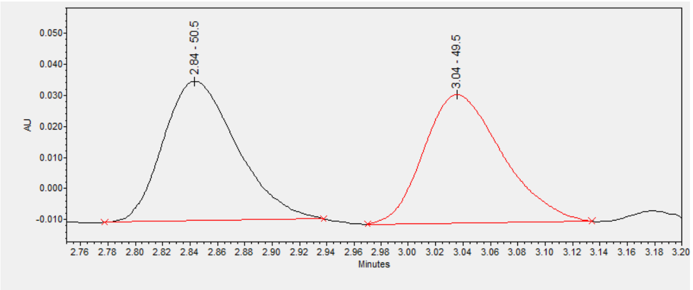

(*S*)-2-(bicyclo[1.1.1]pentan-1-yl)propyl 2-naphthoate (95% *ee*)

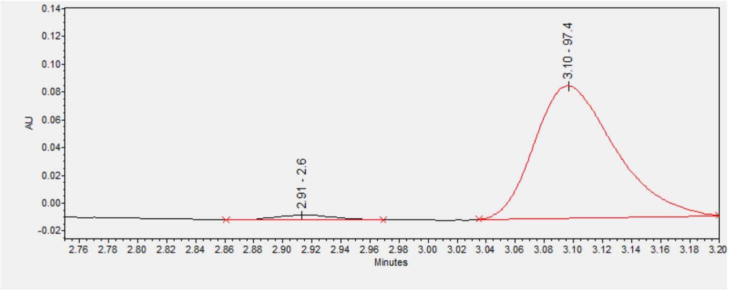

(*R*)-2-(bicyclo[1.1.1]pentan-1-yl)propyl 2-naphthoate (64% *ee*)

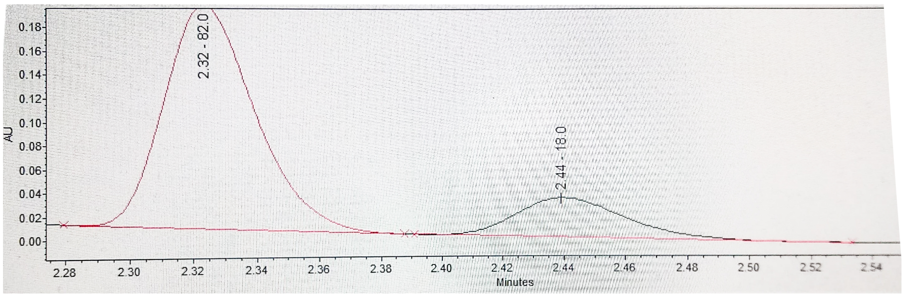

The following aldehydes are commercially available and used directly as supplied: propionaldehyde, isovaleraldehyde, 3,3-dimethylbutyraldehyde, octanal, hydrocinnamaldehyde, (*Z*)-4-hepten-1-al, methional, *N*-Boc-4-piperidineacetaldehyde, 5,5-dimethyl-1,3-dioxane-2-butanal, 3-(5-methyl-2-furyl)propionaldehyde.

General procedure A: Asymmetric ring opening of [1.1.1]propellane

To a flame-dried vial was added Ir[(ppy)$_2$(dtbbpy)]PF$_6$ (0.02 equiv.), 2,4,6-tri-*tert*-butylbenzenethiol **10** (0.1 equiv.), (*S*)-α,α-bis[3,5-bis(trifluoromethyl)phenyl]-2-pyrrolidinemethanol trimethylsilyl ether **9** (0.25 equiv.) and aldehyde (1.5 equiv., if solid). The vial was sealed with a rubber septum and Et_2O, DME and distilled water (2.0 equiv.) were then added (taking into account Et_2O from the [1.1.1]propellane (tricyclo[1.1.1.0^{1,3}]pentane, TCP) **1** solution, final reaction concentration = 0.2 M with a 1:1 ratio of Et_2O to DME). The vial was then cooled in an ice bath (0 °C) and the solution was degassed by sparging with argon for 15 minutes. The vent needle was removed and TCP **1** (1.0 equiv.) and aldehyde (1.5 equiv., if liquid) were then added. The vial was then capped and double-sealed with parafilm. The reaction mixture was then placed in a cooling bath at 10 °C and irradiated with blue LEDs for 24 h. The reaction mixture was then concentrated *in vacuo*. MeOH (5 mL/mmol of TCP **1**) and CH_2Cl_2 (5 mL/mmol of TCP **1**) were added to the vial and the reaction mixture cooled to –78 °C. Sodium borohydride (5.0 equiv.) was added and the reaction mixture was left to stir for 30 min to 1 h at –78 °C. The reaction mixture was quenched by addition of NH_4Cl (aq., sat., 5 mL/mmol of TCP **1**) and water (5 mL/mmol of TCP **1**). The phases were separated and the aqueous phase was extracted with CH_2Cl_2 (× 3). The combined organic extracts were dried (Na_2SO_4) and concentrated *in vacuo*.

(*R*)-2-(Bicyclo[1.1.1]pentan-1-yl)propan-1-ol, 12

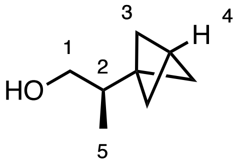

Propionaldehyde (22 µL, 0.30 mmol, 1.5 equiv.), Ir[(ppy)$_2$(dtbbpy)]PF$_6$ (3.7 mg, 4.0 µmol, 0.02 equiv.), 2,4,6-tri-*tert*-butylbenzenethiol **10** (5.6 mg, 0.020 mmol, 0.1 equiv.), (*S*)-α,α-bis[3,5-bis(trifluoromethyl)phenyl]-2-pyrrolidinemethanol trimethylsilyl ether **9** (30 mg, 0.050 mmol, 0.25 equiv.), water (7.2 µL, 0.40 mmol, 2.0 equiv.) and TCP **1** (1.02 M in Et$_2$O, 0.20 mL, 0.20 mmol, 1.0 equiv.) in Et$_2$O (0.3 mL) and DME (0.5 mL), and sodium borohydride (38 mg, 1.0 mmol, 5.0 equiv.) in MeOH (1 mL) and CH$_2$Cl$_2$ (1 mL) were submitted to General Procedure A. Purification by column chromatography (SiO$_2$, pentane/Et$_2$O, 3:2) afforded the title compound as a pale yellow oil (16 mg, 0.13 mmol, 63%).

R*f* 0.31 (pentane/Et$_2$O, 7:3), [vanillin]

^{1}H NMR (400 MHz, CDCl$_3$) δ 3.64 – 3.52 (m, 1H, H1), 3.43 – 3.32 (m, 1H, H1), 2.48 (s, 1H, H4), 1.68 (s, 6H, H3), 1.16 (t, *J* = 5.7 Hz, 1H, H2), 0.85 (d, *J* = 7.0 Hz, 3H, H5).

Note: the O*H* resonance was not observed.

^{13}C NMR (126 MHz, CDCl$_3$) δ 66.6, 49.0 (3C), 47.5, 37.2, 27.4, 13.5.

$[\boldsymbol{\alpha}]_{\mathbf{D}}^{\mathbf{25}}$ – 8.6 (*c* = 1.1, CHCl$_3$)

HPLC 64% *ee* (CHIRALPAK IB, 1% IPA/hexane, 0.8 mL/min, t_R major – 13.2 min, minor – 12.3 min) after derivatization to (*R*)-2-(bicyclo[1.1.1]pentan-1-yl)propyl 4-(dimethylamino)benzoate (*R*)-**S41**.

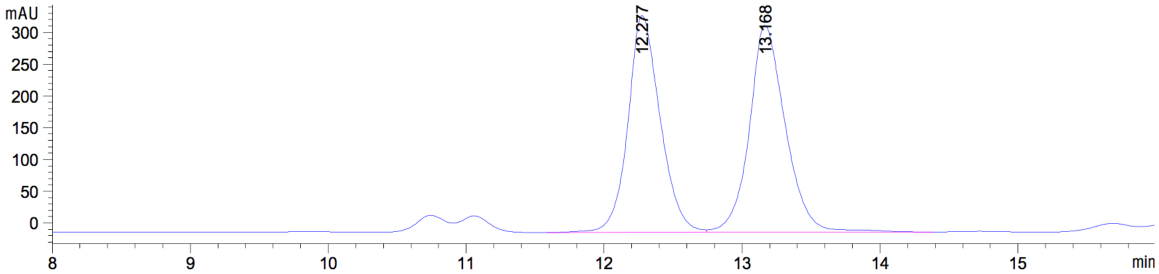

Peak #	RetTime [min]	Type	Width [min]	Area [mAU*s]	Height [mAU]	Area %
1	12.277	BV	0.2416	5550.50684	341.45074	48.3753
2	13.168	VB	0.2682	5923.32666	325.64792	51.6247

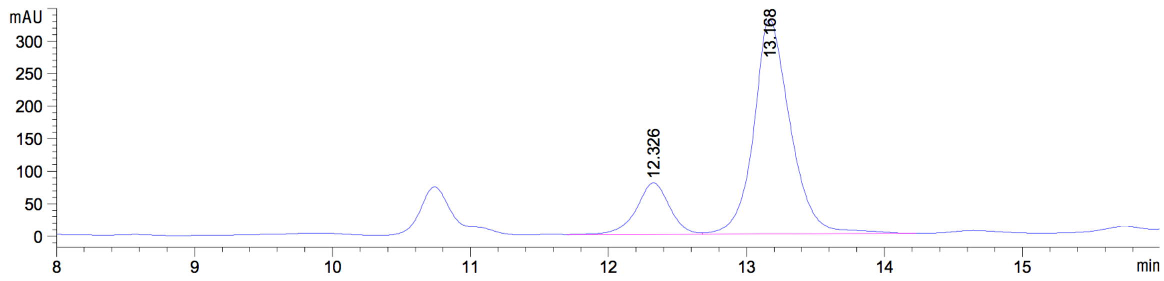

Peak #	RetTime [min]	Type	Width [min]	Area [mAU*s]	Height [mAU]	Area %
1	12.326	BV	0.2507	1319.28052	79.04379	17.8674
2	13.168	VB	0.2705	6064.44385	329.86990	82.1326

Spectroscopic data in agreement with that reported previously.[13]

Derivatization for *ee* determination

2-(Bicyclo[1.1.1]pentan-1-yl)propyl 4-(dimethylamino)benzoate, S41

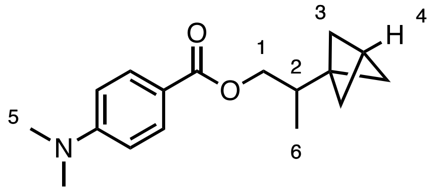

2-(Bicyclo[1.1.1]pentan-1-yl)propan-1-ol *rac*-**12** (6.3 mg, 0.050 mmol, 1.0 equiv.), 4-(dimethylamino)benzoic acid (8.3 mg, 0.050 mmol, 1.0 equiv.), DMAP (18 mg, 0.15 mmol, 3.0 equiv.) and EDCI (29 mg, 0.15 mmol, 3.0 equiv.) in CH_2Cl_2 (0.5 mL) were submitted to General Procedure B for 17 h. Purification by column chromatography (SiO_2, pentane/Et_2O, 4:1) afforded the title compound as a pale yellow oil (10 mg, 0.038 mmol, 76%).

R*f* 0.43 (pentane/Et_2O, 4:1), [UV, vanillin]

^{1}H NMR (400 MHz, $CDCl_3$) δ 7.91 (d, *J* = 9.1 Hz, 2H, Ar*H*), 6.65 (d, *J* = 9.1 Hz, 2H, Ar*H*), 4.15 (dd, *J* = 10.7, 6.7 Hz, 1H, H1), 4.05 (dd, *J* = 10.7, 6.7 Hz, 1H, H1), 3.04 (s, 6H, H5), 2.48 (s, 1H, H4), 1.98 (sept, *J* = 6.7 Hz, 1H, H2), 1.71 (s, 6H, H3), 0.93 (d, *J* = 6.9 Hz, 3H, H6).

^{13}C NMR (151 MHz, $CDCl_3$) δ 167.2, 153.4, 131.4 (2C), 117.5, 110.9 (2C), 67.3, 49.0 (3C), 47.5, 40.2 (2C), 34.2, 27.3, 13.9.

HRMS (ESI$^+$) Found $[M+H]^+$ = 274.1803; $C_{17}H_{24}O_2N$ requires 274.1802.

IR (film) ν_{max}/cm^{-1} 2962, 2870, 1703, 1609, 1527, 1368, 1316, 1279, 1184, 1107.

(*R*)-2-(Bicyclo[1.1.1]pentan-1-yl)-3-methylbutan-1-ol, 13

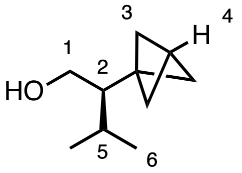

Isovaleraldehyde (32 μL, 0.30 mmol, 1.5 equiv.), Ir[(ppy)$_2$(dtbbpy)]PF$_6$ (3.7 mg, 4.0 μmol, 0.02 equiv.), 2,4,6-tri-*tert*-butylbenzenethiol **10** (5.6 mg, 0.020 mmol, 0.1 equiv.), (*S*)-α,α-bis[3,5-bis(trifluoromethyl)phenyl]-2-pyrrolidinemethanol trimethylsilyl ether **9** (30 mg, 0.050 mmol, 0.25 equiv.), water (7.2 μL, 0.40 mmol, 2.0 equiv.) and TCP **1** (1.02 M in Et_2O, 0.20 mL, 0.20 mmol, 1.0 equiv.) in Et_2O (0.3 mL) and DME (0.5 mL), and sodium borohydride (38 mg, 1.0 mmol, 5.0 equiv.) in MeOH (1 mL) and CH_2Cl_2 (1 mL) were submitted to General Procedure A. Purification by column chromatography (SiO_2, pentane/Et_2O, 7:3) afforded the title compound as a pale yellow oil (25 mg, 0.16 mmol, 80%).

R*f* 0.40 (pentane/Et_2O, 7:3), [vanillin]

^{1}H NMR (400 MHz, $CDCl_3$) δ 3.64 (td, *J* = 6.0, 2.1 Hz, 2H, H1), 2.47 (s, 1H, H4), 1.88 – 1.81 (m, 1H, H5), 1.79 (d, *J* = 1.3 Hz, 6H, H3), 1.45 – 1.42 (m, 1H, H2), 0.97 (d, *J* = 7.0 Hz, 3H, H6), 0.91 (d, *J* = 7.0 Hz, 3H, H6).

Note: the O*H* resonance was not observed.

^{13}C NMR (126 MHz, $CDCl_3$) δ 63.0, 51.3 (3C), 48.0, 47.4, 28.2, 28.1, 21.3, 19.8.

HRMS (ESI$^+$) Found $[M+H]^+$ = 155.1428; $C_{10}H_{19}O$ requires 155.1430.

IR (film) ν_{max}/cm^{-1} 3441, 3360, 2959, 2870, 1607, 1518, 1460, 1360, 1280.

$[\alpha]_D^{25}$ – 74.8 (*c* = 0.1, $CHCl_3$)

HPLC 96% *ee* (Lux i-Amylose-1, 3% IPA/hexane, 0.7 mL/min, t_R major – 15.4 min, minor – 14.5 min) after derivatization to (*R*)-2-(bicyclo[1.1.1]pentan-1-yl)-3-methylbutyl 4-(dimethylamino)benzoate (*R*)-**S42**.

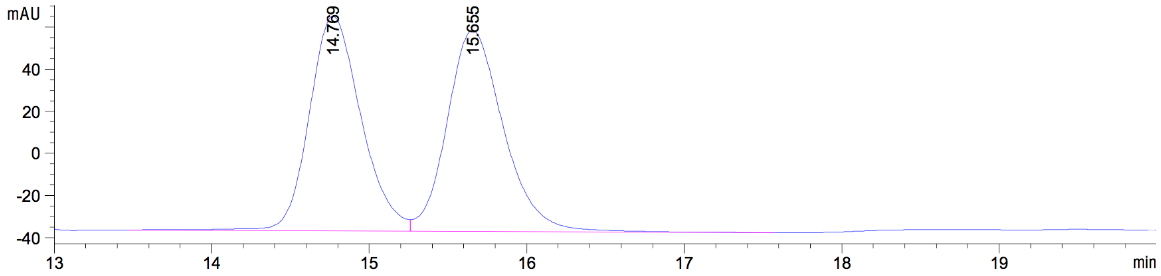

Peak #	RetTime [min]	Type	Width [min]	Area [mAU*s]	Height [mAU]	Area %
1	14.769	BV	0.3510	2337.20679	102.14143	49.5028
2	15.655	VB	0.3806	2384.15771	95.71879	50.4972

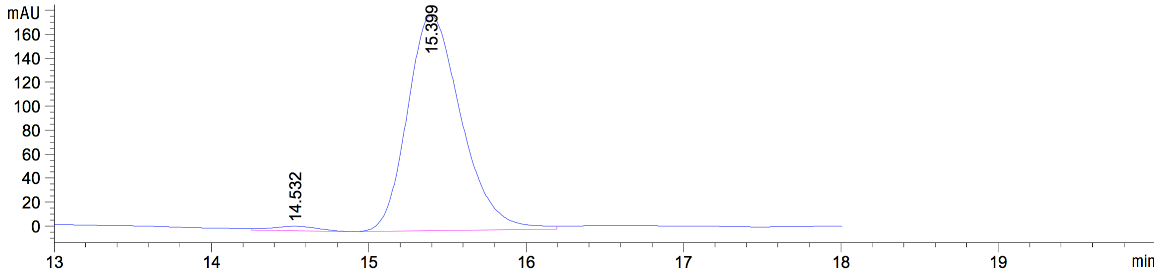

Peak #	RetTime [min]	Type	Width [min]	Area [mAU*s]	Height [mAU]	Area %
1	14.532	VB	0.3060	77.74979	3.77922	1.7877
2	15.399	BV	0.3652	4271.45654	179.79712	98.2123

Derivatization for *ee* determination

2-(Bicyclo[1.1.1]pentan-1-yl)-3-methylbutyl 4-(dimethylamino)benzoate, S42

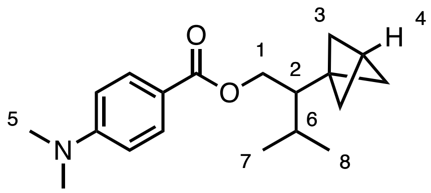

2-(Bicyclo[1.1.1]pentan-1-yl)-3-methylbutan-1-ol *rac*-**13** (6.1 mg, 0.040 mmol, 1.0 equiv.), 4-(dimethylamino)benzoic acid (6.5 mg, 0.040 mmol, 1.0 equiv.), DMAP (15 mg, 0.12 mmol, 3.0 equiv.) and EDCI (23 mg, 0.12 mmol, 3.0 equiv.) in CH_2Cl_2 (0.4 mL) were submitted to General Procedure B for 15 h. Purification by column chromatography (SiO_2, pentane/Et_2O, 19:1) afforded the title compound as a pale yellow oil (9.5 mg, 0.032 mmol, 80%).

R_f 0.31 (pentane/Et_2O, 9:1), [UV, vanillin]

^{1}H NMR (500 MHz, $CDCl_3$) δ 7.90 (d, *J* = 9.1 Hz, 2H, Ar*H*), 6.66 (d, *J* = 9.1 Hz, 2H, Ar*H*), 4.26 (dd, *J* = 11.0, 5.4 Hz, 1H, H1), 4.22 (dd, *J* = 11.0, 6.6 Hz, 1H, H1), 3.04 (s, 6H, H5),

2.45 (s, 1H, H4), 1.95 – 1.87 (m, 1H, H6), 1.81 (d, J = 1.9 Hz, 6H, H3), 1.75 – 1.70 (m, 1H, H2), 1.02 (d, J = 6.9 Hz, 3H, H7), 0.94 (d, J = 7.1 Hz, 3H, H8).

^{13}C NMR (126 MHz, $CDCl_3$) δ 167.3, 153.4, 131.4 (2C), 117.5, 110.9 (2C), 64.2, 51.1 (3C), 47.4, 44.6, 40.2 (2C), 28.5, 27.9, 21.7, 19.5.

HRMS (ESI^+) Found $[M+H]^+$ = 302.2115; $C_{19}H_{28}O_2N$ requires 302.2115.

IR (film) ν_{max}/cm^{-1} 2960, 2919, 2870, 1702, 1608, 1527, 1465, 1446, 1366, 1317, 1278, 1232, 1183, 1107.

(*R*)-2-(Bicyclo[1.1.1]pentan-1-yl)-3,3-dimethylbutan-1-ol, 14

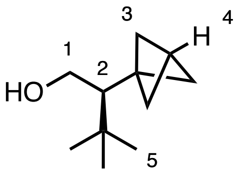

3,3-Dimethylbutyraldehyde (38 μL, 0.30 mmol, 1.5 equiv.), Ir[(ppy)$_2$(dtbbpy)]PF_6 (3.7 mg, 4.0 μmol, 0.02 equiv.), 2,4,6-tri-*tert*-butylbenzenethiol **10** (5.6 mg, 0.020 mmol, 0.1 equiv.), (*S*)-α,α-bis[3,5-bis(trifluoromethyl)phenyl]-2-pyrrolidinemethanol trimethylsilyl ether **9** (30 mg, 0.050 mmol, 0.25 equiv.), water (7.2 μL, 0.40 mmol, 2.0 equiv.) and TCP **1** (1.02 M in Et_2O, 0.20 mL, 0.20 mmol, 1.0 equiv.) in Et_2O (0.3 mL) and DME (0.5 mL), and sodium borohydride (38 mg, 1.0 mmol, 5.0 equiv.) in MeOH (1 mL) and CH_2Cl_2 (1 mL) were submitted to General Procedure A. Purification by column chromatography (SiO_2, pentane/Et_2O, 9:1) afforded the title compound as a pale yellow oil (15 mg, 0.087 mmol, 44%).

R*f* 0.24 (pentane/Et_2O, 9:1), [vanillin]

^{1}H NMR (400 MHz, $CDCl_3$) δ 3.74 (ddd, J = 11.0, 5.8, 3.3 Hz, 1H, H1), 3.65 (dt, J = 11.0, 6.0 Hz, 1H, H1), 2.46 (s, 1H, H4), 1.90 (s, 6H, H3), 1.31 (dd, J = 6.5, 3.3 Hz, 1H, H2), 0.95 (s, 9H, H5).

Note: the O*H* resonance was not observed.

^{13}C NMR (126 MHz, $CDCl_3$) δ 63.4, 52.6, 52.5 (3C), 47.6, 33.9, 29.1 (3C), 28.9.

HRMS (ESI^+) Found $[M+H]^+$ = 169.1589; $C_{11}H_{21}O$ requires 169.1587.

IR (film) ν_{max}/cm^{-1} 2958, 2923, 2871, 1463, 1365, 1325, 1245, 1199.

$[\alpha]_D^{25}$ – 29.5 (c = 0.1, $CHCl_3$)

HPLC 98% *ee* (Lux i-Amylose-1, 6% IPA/hexane, 0.7 mL/min, t_R major – 11.8 min, minor – 12.9 min) after derivatization to (*R*)-2-(bicyclo[1.1.1]pentan-1-yl)-3,3-dimethylbutyl 4-(dimethylamino)benzoate (*R*)-**S43**.

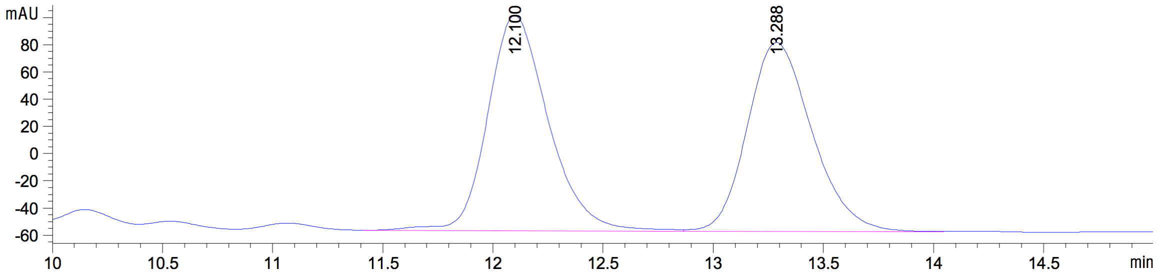

Peak #	RetTime [min]	Type	Width [min]	Area [mAU*s]	Height [mAU]	Area %
1	12.100	BV	0.2913	3025.22168	158.02417	51.5715
2	13.288	VV	0.3165	2840.85010	138.98952	48.4285

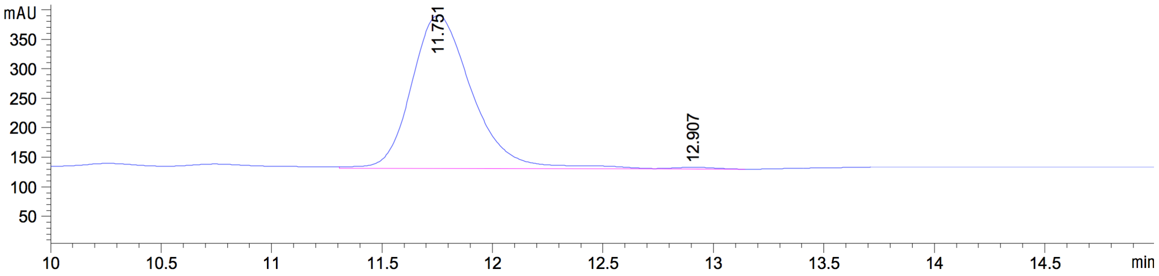

Peak #	RetTime [min]	Type	Width [min]	Area [mAU*s]	Height [mAU]	Area %
1	11.751	VV	0.2913	4967.98535	259.54993	99.2130
2	12.907	VB	0.2020	39.40602	2.98594	0.7870

Derivatization for *ee* determination

2-(Bicyclo[1.1.1]pentan-1-yl)-3,3-dimethylbutyl 4-(dimethylamino)benzoate, S43

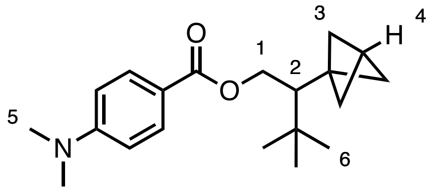

2-(Bicyclo[1.1.1]pentan-1-yl)-3,3-dimethylbutan-1-ol *rac*-**14** (9.4 mg, 0.056 mmol, 1.0 equiv.), 4-(dimethylamino)benzoic acid (9.2 mg, 0.056 mmol, 1.0 equiv.), DMAP (21 mg, 0.17 mmol, 3.0 equiv.) and EDCI (32 mg, 0.17 mmol, 3.0 equiv.) in CH_2Cl_2 (0.6 mL) were submitted to General Procedure B for 16 h. Purification by column chromatography (SiO_2, pentane/Et_2O, 19:1) afforded the title compound as a pale yellow oil (16 mg, 0.050 mmol, 89%).

R$_f$ 0.29 (pentane/Et_2O, 9:1), [UV, vanillin]

^{1}H NMR (500 MHz, $CDCl_3$) δ 7.91 (d, J = 9.1 Hz, 2H, ArH), 6.67 (d, J = 9.0 Hz, 2H, ArH), 4.37 (dd, J = 11.3, 3.7 Hz, 1H, H1), 4.24 (dd, J = 11.3, 6.4 Hz, 1H, H1), 3.04 (s, 6H, H5), 2.42 (s, 1H, H4), 1.91 (s, 6H, H3), 1.63 (dd, J = 6.4, 3.7 Hz, 1H, H2), 1.02 (s, 9H, H6).

^{13}C NMR (126 MHz, $CDCl_3$) δ 167.3, 153.3, 131.4 (2C), 117.7, 111.0 (2C), 64.7, 52.7 (3C), 48.8, 47.4, 40.3 (2C), 34.0, 29.1 (3C), 28.6.

HRMS (ESI^+) Found $[M+H]^+$ = 316.2271; $C_{20}H_{30}O_2N$ requires 316.2271.

IR (film) ν_{max}/cm^{-1} 2961, 2908, 2870, 1702, 1609, 1527, 1477, 1446, 1365, 1280, 1183, 1108.

(*R*)-2-(Bicyclo[1.1.1]pentan-1-yl)octan-1-ol, 15

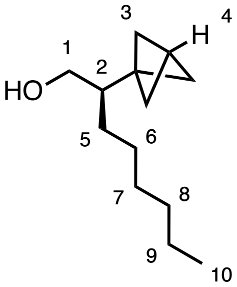

Octanal (47 μL, 0.30 mmol, 1.5 equiv.), Ir[$(ppy)_2$(dtbbpy)]PF_6 (3.7 mg, 4.0 μmol, 0.02 equiv.), 2,4,6-tri-*tert*-butylbenzenethiol **10** (5.6 mg, 0.020 mmol, 0.1 equiv.), (*S*)-α,α-bis[3,5-bis(trifluoromethyl)phenyl]-2-pyrrolidinemethanol trimethylsilyl ether **9** (30 mg, 0.050 mmol, 0.25 equiv.), water (7.2 μL, 0.40 mmol, 2.0 equiv.) and TCP **1** (1.02 M in Et_2O, 0.20 mL, 0.20 mmol, 1.0 equiv.) in Et_2O (0.3 mL) and DME (0.5 mL), and sodium borohydride (38 mg, 1.0 mmol, 5.0 equiv.) in MeOH (1 mL) and CH_2Cl_2 (1 mL) were submitted to General Procedure A. Purification by column chromatography (SiO_2, pentane/Et_2O, 17:3) afforded the title compound as a pale yellow oil (38 mg, 0.19 mmol, 96%).

R$_f$ 0.24 (pentane/Et_2O, 4:1), [vanillin]

^{1}H NMR (500 MHz, $CDCl_3$) δ 3.57 (dd, J = 10.7, 6.0 Hz, 1H, H1), 3.53 (dd, J = 10.7, 5.2 Hz, 1H, H1), 2.48 (s, 1H, H4), 1.71 (s, 6H, H3), 1.52 – 1.48 (m, 1H, H2), 1.31 – 1.24 (m, 10H, H5, H6, H7, H8 and H9), 0.88 (t, J = 6.8 Hz, 3H, H10).

Note: the OH resonance was not observed.

^{13}C NMR (126 MHz, $CDCl_3$) δ 64.7, 49.8 (3C), 47.6, 42.2, 32.0, 29.9, 28.6, 27.8, 27.7, 22.8, 14.2.

HRMS (APCI) Found $[M+H]^+$ = 197.1901; $C_{13}H_{25}O$ requires 197.1900.

IR (film) ν_{max}/cm^{-1} 3351, 2959, 2925, 2868, 1747, 1465.

$[\boldsymbol{\alpha}]_{\mathbf{D}}^{\mathbf{25}}$ – 51.9 (c = 0.1, $CHCl_3$)

HPLC 97% *ee* (CHIRALPAK IB, 3% IPA/hexane, 1.0 mL/min, t_R major – 5.6 min, minor – 6.0 min) after derivatization to (*R*)-2-(bicyclo[1.1.1]pentan-1-yl)octyl 4-(dimethylamino)benzoate (*R*)-**S44**.

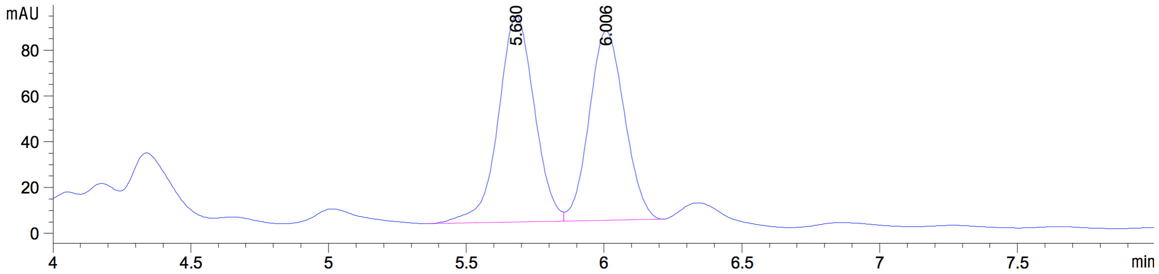

Peak #	RetTime [min]	Type	Width [min]	Area [mAU*s]	Height [mAU]	Area %
1	5.680	BV	0.1403	822.12469	90.24281	52.6855
2	6.006	VB	0.1401	738.31372	82.78297	47.3145

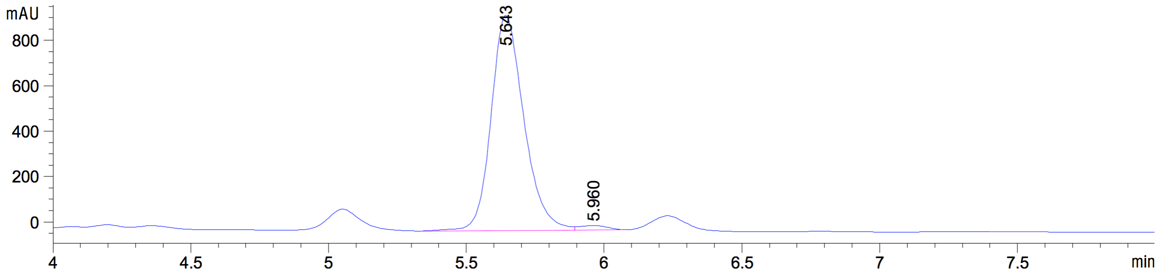

Peak #	RetTime [min]	Type	Width [min]	Area [mAU*s]	Height [mAU]	Area %
1	5.643	BV	0.1244	7833.54150	948.16602	98.2714
2	5.960	VB	0.1086	137.78886	19.45575	1.7286

Derivatization for *ee* determination

2-(Bicyclo[1.1.1]pentan-1-yl)octyl 4-(dimethylamino)benzoate, S44

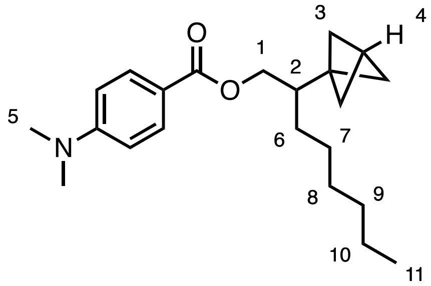

2-(Bicyclo[1.1.1]pentan-1-yl)octan-1-ol *rac*-**15** (13 mg, 0.064 mmol, 1.0 equiv.), 4-(dimethylamino)benzoic acid (11 mg, 0.064 mmol, 1.0 equiv.), DMAP (24 mg, 0.19 mmol, 3.0 equiv.) and EDCI (37 mg, 0.19 mmol, 3.0 equiv.) in CH_2Cl_2 (0.6 mL) were submitted to General Procedure B for 25.5 h. Purification by column chromatography (SiO_2, pentane/Et_2O, 9:1) afforded the title compound as a pale yellow oil (20 mg, 0.059 mmol, 93%).

R*f* 0.76 (pentane/Et_2O, 3:2), [UV, vanillin]
^{1}H NMR (500 MHz, $CDCl_3$) δ 7.90 (d, *J* = 9.1 Hz, 2H, Ar*H*), 6.65 (d, *J* = 9.1 Hz, 2H, Ar*H*), 4.18 (dd, *J* = 10.8, 5.1 Hz, 1H, H1), 4.14 (dd, *J* = 10.9, 6.3 Hz, 1H, H1), 3.04 (s, 6H, H5), 2.46 (s, 1H, H4), 1.82 – 1.75 (m, 1H, H2), 1.74 (s, 6H, H3), 1.40 – 1.26 (m, 10H, H6, H7, H8, H9 and H10), 0.87 (t, *J* = 6.9 Hz, 3H, H11).
^{13}C NMR (126 MHz, $CDCl_3$) δ 167.2, 153.4, 131.4 (2C), 117.6, 110.9 (2C), 65.7, 49.8 (3C), 47.6, 40.2 (2C), 39.1, 31.9, 29.8, 28.9, 27.7, 27.5, 22.8, 14.3.
HRMS (ESI$^+$) Found $[M+H]^+$ = 344.2584; $C_{22}H_{34}O_2N$ requires 344.2584.
IR (film) ν_{max}/cm^{-1} 2959, 2926, 2869, 1704, 1609, 1528, 1446, 1366, 1317, 1279, 1183, 1108.

(*R*)-2-(Bicyclo[1.1.1]pentan-1-yl)-3-phenylpropan-1-ol, 16

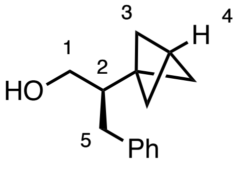

Hydrocinnamaldehyde (40 μL, 0.30 mmol, 1.5 equiv.), Ir[(ppy)$_2$(dtbbpy)]PF_6 (3.7 mg, 4.0 μmol, 0.02 equiv.), 2,4,6-tri-*tert*-butylbenzenethiol **10** (5.6 mg, 0.020 mmol, 0.1 equiv.), (*S*)-α,α-bis[3,5-bis(trifluoromethyl)phenyl]-2-pyrrolidinemethanol trimethylsilyl

ether **9** (30 mg, 0.050 mmol, 0.25 equiv.), water (7.2 µL, 0.40 mmol, 2.0 equiv.) and TCP **1** (1.02 M in Et_2O, 0.20 mL, 0.20 mmol, 1.0 equiv.) in Et_2O (0.3 mL) and DME (0.5 mL), and sodium borohydride (38 mg, 1.0 mmol, 5.0 equiv.) in MeOH (1 mL) and CH_2Cl_2 (1 mL) were submitted to General Procedure A. Purification by column chromatography (SiO_2, pentane/Et_2O, 17:3) afforded the title compound as a pale yellow oil (40 mg, 0.20 mmol, 99%).

Scaled-up procedure

Hydrocinnamaldehyde (0.20 mL, 1.5 mmol, 1.5 equiv.), Ir[(ppy)$_2$(dtbbpy)]PF_6 (9.1 mg, 0.010 mmol, 0.01 equiv.), 2,4,6-tri-*tert*-butylbenzenethiol **10** (28 mg, 0.10 mmol, 0.1 equiv.), (*S*)-α,α-bis[3,5-bis(trifluoromethyl)phenyl]-2-pyrrolidinemethanol trimethylsilyl ether **9** (0.12 g, 0.20 mmol, 0.20 equiv.), water (36 µL, 2.0 mmol, 2.0 equiv.) and TCP **1** (0.71 M in Et_2O, 1.4 mL, 1.0 mmol, 1.0 equiv.) in Et_2O (1.1 mL) and DME (2.5 mL), and sodium borohydride (0.19 g, 5.0 mmol, 5.0 equiv.) in MeOH (5 mL) and CH_2Cl_2 (5 mL) were submitted to General Procedure A. Purification by column chromatography (SiO_2, pentane/Et_2O, 17:3) afforded the title compound as a pale yellow oil (0.20 g, 0.98 mmol, 98%).

$\mathbf{R}_f$ 0.33 (pentane/Et_2O, 7:3), [UV, vanillin]

^{1}H NMR (500 MHz, $CDCl_3$) δ 7.31 – 7.27 (m, 2H, Ar*H*), 7.22 – 7.16 (m, 3H, Ar*H*), 3.58 – 3.48 (m, 2H, H1), 2.66 (dd, *J* = 13.9, 5.6 Hz, 1H, H5), 2.53 (dd, *J* = 13.9, 9.5 Hz, 1H, H5), 2.49 (s, 1H, H4), 1.92 (dq, *J* = 9.5, 5.6 Hz, 1H, H2), 1.74 (dd, *J* = 9.6, 1.6 Hz, 3H, H3), 1.71 (dd, *J* = 9.6, 1.6 Hz, 3H, H3).

Note: the O*H* resonance was not observed.

^{13}C NMR (126 MHz, $CDCl_3$) δ 141.0, 129.1 (2C), 128.5 (2C), 126.0, 64.0, 49.8 (3C), 47.5, 44.0, 35.1, 27.7.

HRMS (ESI$^+$) Found $[M+Na]^+$ = 225.1251; $C_{14}H_{18}ONa$ requires 225.1250.

IR (film) ν_{max}/cm^{-1} 3331, 3027, 2961, 2907, 2869, 1495, 1453, 1279, 1196.

$[\boldsymbol{\alpha}]_{\mathbf{D}}^{\mathbf{25}}$ – 13.1 (*c* = 0.2, $CHCl_3$)

HPLC 96% *ee* (CHIRALPAK IB, 2% IPA/hexane, 1.0 mL/min, t_R major – 9.5 min, minor – 11.5 min).

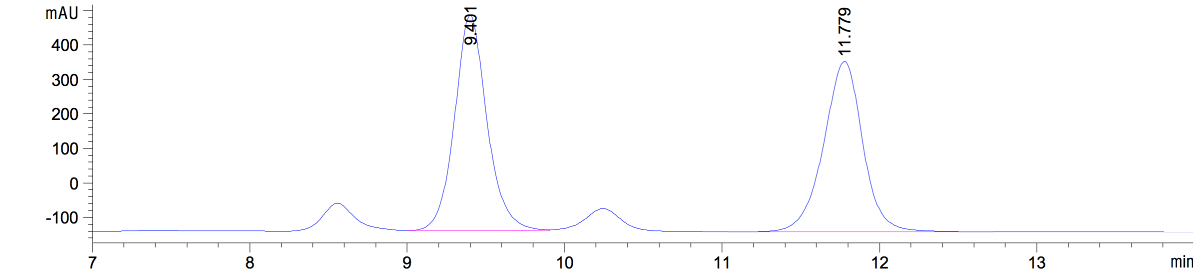

Peak #	RetTime [min]	Type	Width [min]	Area [mAU*s]	Height [mAU]	Area %
1	9.401	BV	0.2160	9006.82031	625.47729	51.2595
2	11.779	BB	0.2643	8564.19629	493.98157	48.7405

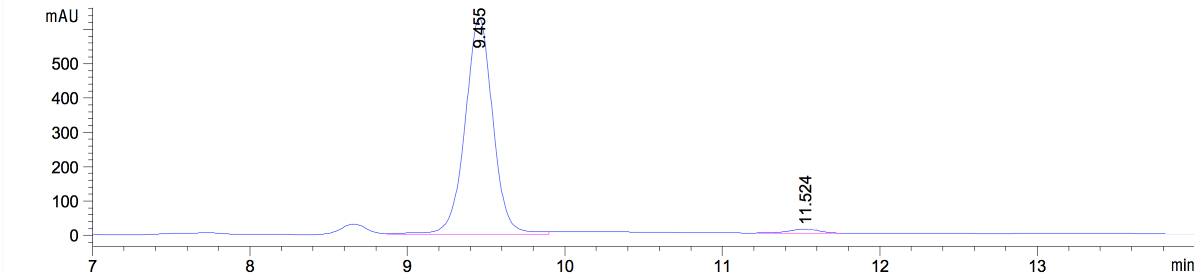

Peak #	RetTime [min]	Type	Width [min]	Area [mAU*s]	Height [mAU]	Area %
1	9.455	VV	0.1877	7746.19580	629.00031	97.9177
2	11.524	MM	0.2275	164.73134	12.06894	2.0823

(*R*)-2-(Bicyclo[1.1.1]pentan-1-yl)-3-(4-methoxyphenyl)propan-1-ol, 17

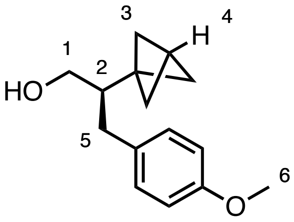

3-(4-Methoxyphenyl)propanal **S76** (49 mg, 0.30 mmol, 1.5 equiv.), Ir[(ppy)$_2$(dtbbpy)]PF$_6$ (3.7 mg, 4.0 µmol, 0.02 equiv.), 2,4,6-tri-*tert*-butylbenzene-thiol **10** (5.6 mg, 0.020 mmol, 0.1 equiv.), (*S*)-α,α-bis[3,5-bis(trifluoromethyl)phenyl]-2-pyrrolidinemethanol trimethylsilyl ether **9** (30 mg, 0.050 mmol, 0.25 equiv.), water (7.2 µL, 0.40 mmol, 2.0 equiv.) and TCP **1** (1.02 M in Et_2O, 0.20 mL, 0.20 mmol, 1.0 equiv.) in Et_2O (0.3 mL) and DME (0.5 mL), and sodium borohydride (38 mg, 1.0 mmol, 5.0 equiv.) in MeOH (1 mL) and CH_2Cl_2 (1 mL) were submitted to General Procedure A. Purification by column chromatography (SiO_2, pentane/Et_2O, 7:3) afforded the title compound as a pale yellow oil (33 mg, 0.14 mmol, 72%).

R*f* 0.29 (pentane/Et_2O, 3:2), [UV, vanillin]

^{1}H NMR (500 MHz, $CDCl_3$) δ 7.10 (d, J = 8.6 Hz, 2H, ArH), 6.82 (d, J = 8.6 Hz, 2H, ArH), 3.79 (s, 3H, H6), 3.54 (dd, J = 10.7, 5.0 Hz, 1H, H1), 3.51 (dd, J = 10.7, 5.4 Hz, 1H, H1), 2.61 (dd, J = 14.1, 5.7 Hz, 1H, H5), 2.49 (s, 1H, H4), 2.46 (dd, J = 14.1, 9.6 Hz, 1H, H5), 1.86 (dq, J = 10.7, 5.4 Hz, 1H, H2), 1.73 (dd, J = 9.6, 1.6 Hz, 3H, H3), 1.71 (dd, J = 9.6, 1.6 Hz, 3H, H3).

Note: the OH resonance was not observed.

^{13}C NMR (126 MHz, $CDCl_3$) δ 158.0, 132.9, 130.0 (2C), 113.9 (2C), 64.1, 55.4, 49.8 (3C), 47.5, 44.1, 34.2, 27.7.

HRMS (ESI^+) Found $[M+H]^+$ = 233.1537; $C_{15}H_{21}O_2$ requires 233.1536.

IR (film) ν_{max}/cm^{-1} 3406, 2961, 2907, 2869, 2835, 1612, 1512, 1246.

$[\alpha]_D^{25}$ – 15.3 (c = 0.3, $CHCl_3$)

HPLC 90% *ee* (Lux i-Amylose-1, 6% IPA/hexane, 1.0 mL/min, t_R major – 10.6 min, minor – 9.9 min).

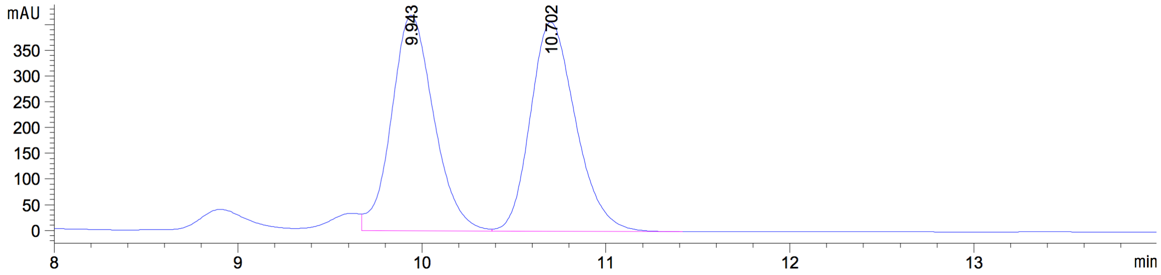

Peak #	RetTime [min]	Type	Width [min]	Area [mAU*s]	Height [mAU]	Area %
1	9.943	VV	0.2448	6708.43457	419.01547	49.1545
2	10.702	VB	0.2611	6939.21582	406.52600	50.8455

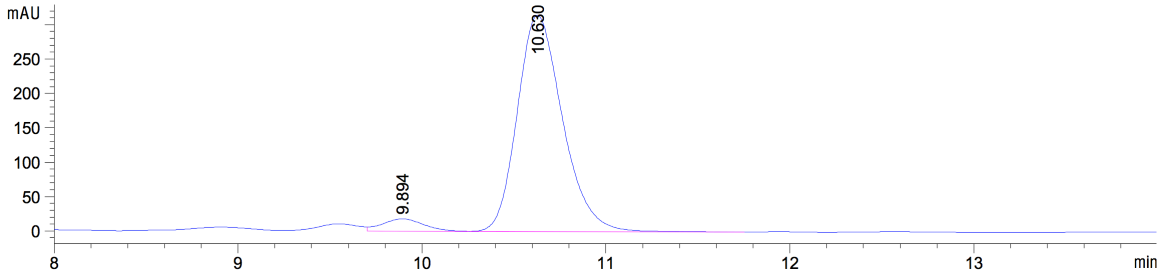

Peak #	RetTime [min]	Type	Width [min]	Area [mAU*s]	Height [mAU]	Area %
1	9.894	VB	0.2390	280.94162	17.90816	4.9705
2	10.630	BV	0.2632	5371.23438	314.64819	95.0295

(*R*)-2-(Bicyclo[1.1.1]pentan-1-yl)-3-(4-(trifluoromethyl)phenyl)propan-1-ol, 18

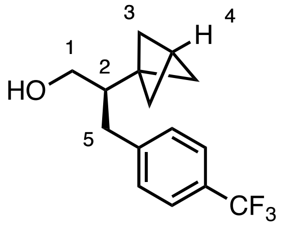

3-(4-(Trifluoromethyl)phenyl)propanal **S78** (61 mg, 0.30 mmol, 1.5 equiv.), Ir[(ppy)$_2$(dtbbpy)]PF$_6$ (3.7 mg, 4.0 μmol, 0.02 equiv.), 2,4,6-tri-*tert*-butylbenzene-thiol **10** (5.6 mg, 0.020 mmol, 0.1 equiv.), (*S*)-α,α-bis[3,5-bis(trifluoromethyl)phenyl]-2-pyrrolidinemethanol trimethylsilyl ether **9** (30 mg, 0.050 mmol, 0.25 equiv.), water (7.2 μL, 0.40 mmol, 2.0 equiv.) and TCP **1** (1.02 M in Et_2O, 0.20 mL, 0.20 mmol, 1.0 equiv.) in Et_2O (0.3 mL) and DME (0.5 mL), and sodium borohydride (38 mg, 1.0 mmol, 5.0 equiv.) in MeOH (1 mL) and CH_2Cl_2 (1 mL) were submitted to General Procedure A. Purification by column chromatography (SiO_2, pentane/Et_2O, 7:3) afforded the title compound as a pale yellow oil (42 mg, 0.16 mmol, 78%).

R*f* 0.36 (pentane/Et_2O, 3:2), [UV, vanillin]

^{1}H NMR (500 MHz, $CDCl_3$) δ 7.52 (d, *J* = 8.0 Hz, 2H, Ar*H*), 7.31 (d, *J* = 8.0 Hz, 2H, Ar*H*), 3.54 (dd, *J* = 10.9, 4.8 Hz, 1H, H1), 3.51 (dd, *J* = 10.9, 5.3 Hz, 1H, H1), 2.71 – 2.62 (m, 2H, H5), 2.50 (s, 1H, H4), 1.95 – 1.89 (m, 1H, H2), 1.74 (dd, *J* = 9.6, 1.7 Hz, 3H, H3), 1.70 (dd, *J* = 9.6, 1.7 Hz, 3H, H3).

Note: the O*H* resonance was not observed.

^{13}C NMR (126 MHz, $CDCl_3$) δ 145.3, 129.4 (2C), 128.4 (q, *J* = 32.3 Hz), 125.3 (q, *J* = 3.8 Hz, 2C), 124.8 (q, *J* = 271.9 Hz), 63.6, 49.8 (3C), 47.2, 43.8, 34.8, 27.8.

^{19}F NMR (471 MHz, $CDCl_3$) δ –62.3.

HRMS (ESI$^-$) Found [M–H]$^-$ = 269.1160; $C_{15}H_{16}OF_3$ requires 269.1159.

IR (film) ν_{max}/cm^{-1} 3343, 2965, 2909, 2872, 1325, 1280, 1163, 1123, 1067, 1019.

$[\alpha]_D^{25}$ – 10.6 (*c* = 0.4, $CHCl_3$)

HPLC 95% *ee* (Lux i-Amylose-1, 1% IPA/hexane, 1.0 mL/min, t_R major – 25.8 min, minor – 23.4 min).

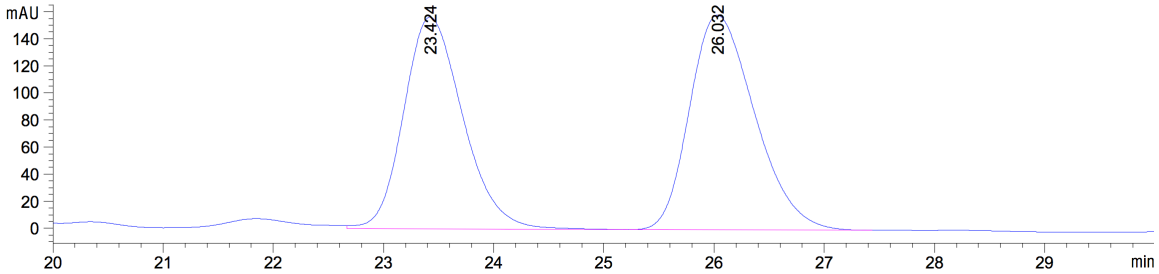

Peak #	RetTime [min]	Type	Width [min]	Area [mAU*s]	Height [mAU]	Area %
1	23.424	VB	0.5797	5844.25537	156.48665	47.3000
2	26.032	BB	0.6411	6511.46191	158.57822	52.7000

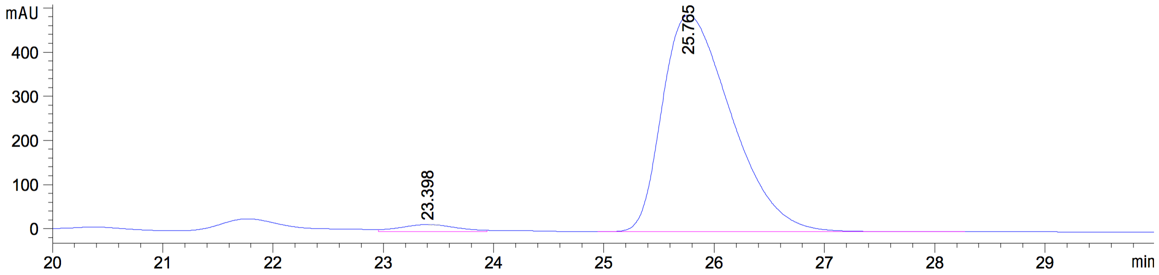

Peak #	RetTime [min]	Type	Width [min]	Area [mAU*s]	Height [mAU]	Area %
1	23.398	MM	0.5850	554.15491	15.78741	2.5339
2	25.765	BB	0.6789	2.13154e4	490.59280	97.4661

(*R*,*Z*)-2-(Bicyclo[1.1.1]pentan-1-yl)hept-4-en-1-ol, 19

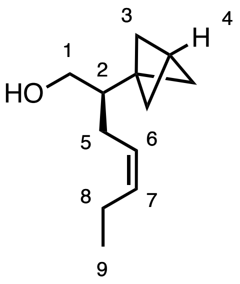

(*Z*)-4-Hepten-1-al (40 µL, 0.30 mmol, 1.5 equiv.), Ir[(ppy)$_2$(dtbbpy)]PF$_6$ (3.7 mg, 4.0 µmol, 0.02 equiv.), 2,4,6-tri-*tert*-butylbenzenethiol **10** (5.6 mg, 0.020 mmol, 0.1 equiv.), (*S*)-α,α-bis[3,5-bis(trifluoromethyl)phenyl]-2-pyrrolidinemethanol trimethylsilyl ether **9** (30 mg, 0.050 mmol, 0.25 equiv.), water (7.2 µL, 0.40 mmol, 2.0 equiv.) and TCP **1** (1.02 M in Et_2O, 0.20 mL, 0.20 mmol, 1.0 equiv.) in Et_2O (0.3 mL) and DME (0.5 mL), and sodium borohydride (38 mg, 1.0 mmol, 5.0 equiv.) in MeOH (1 mL) and CH_2Cl_2 (1 mL) were submitted to General Procedure A. Purification by column chromatography (SiO_2, pentane/Et_2O, 17:3) afforded the title compound as a pale yellow oil (25 mg, 0.14 mmol, 70%).

R$_f$ 0.43 (pentane/Et_2O, 7:3), [UV, vanillin]

^{1}H NMR (500 MHz, $CDCl_3$) δ 5.45 – 5.33 (m, 2H, H6 and H7), 3.58 (dd, J = 10.8, 5.8 Hz, 1H, H1), 3.53 (dd, J = 10.8, 5.8 Hz, 1H, H1), 2.48 (s, 1H, H4), 2.10 – 2.01 (m, 4H, H5 and H8), 1.72 (s, 6H, H3), 1.67 – 1.62 (m, 1H, H2), 0.97 (t, J = 7.5 Hz, 3H, H9).

Note: the O*H* resonance was not observed.

^{13}C NMR (126 MHz, $CDCl_3$) δ 133.0, 127.7, 64.9, 49.8 (3C), 47.3, 42.7, 27.7, 26.8, 20.7, 14.3.

HRMS (ESI$^+$) Found $[M+H]^+$ = 181.1587; $C_{12}H_{21}O$ requires 181.1587.

IR (film) ν_{max}/cm^{-1} 3317, 2962, 2907, 2870, 1462, 1374, 1279, 1196, 1143, 1067, 1025.

$[\alpha]_D^{25}$ – 24.1 (c = 0.2, $CHCl_3$)

HPLC 90% *ee* (CHIRALPAK IC, 2% IPA/hexane, 1.0 mL/min, t_R major – 17.2 min, minor – 15.2 min) after derivatization to (*R*,*Z*)-2-(bicyclo[1.1.1]pentan-1-yl)hept-4-en-1-yl 4-(dimethylamino)benzoate (*R*)-**S45**.

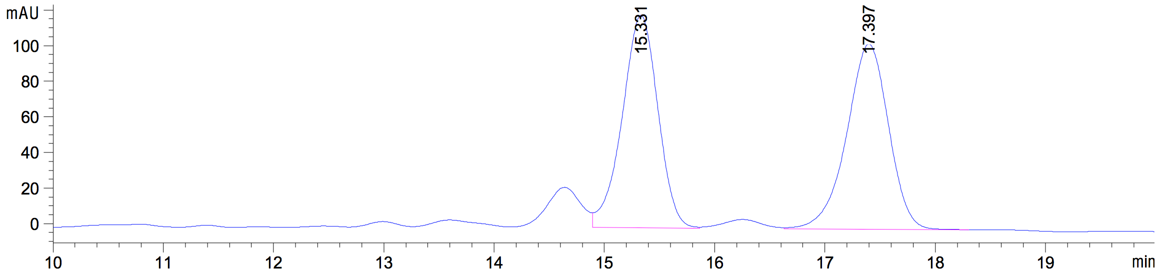

Peak #	RetTime [min]	Type	Width [min]	Area [mAU*s]	Height [mAU]	Area %
1	15.331	VV	0.3468	2688.97925	119.38303	49.5241
2	17.397	VB	0.4037	2740.65942	103.89968	50.4759

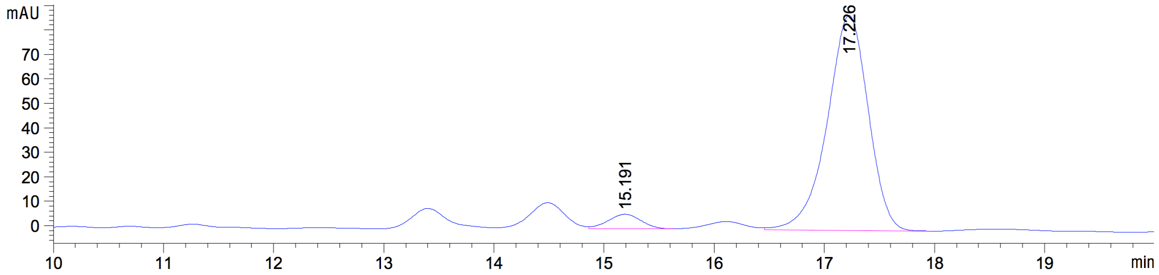

Peak #	RetTime [min]	Type	Width [min]	Area [mAU*s]	Height [mAU]	Area %
1	15.191	VB	0.3159	123.43719	5.90328	5.1538
2	17.226	VB	0.3935	2271.64990	87.91320	94.8462

Derivatization for *ee* determination

(*Z*)-2-(Bicyclo[1.1.1]pentan-1-yl)hept-4-en-1-yl 4-(dimethylamino)benzoate, S45

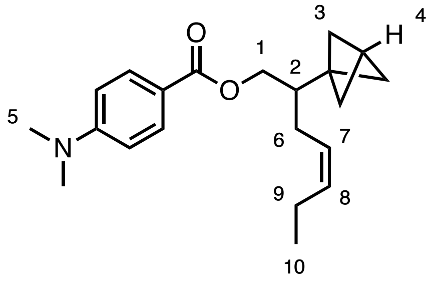

(*Z*)-2-(Bicyclo[1.1.1]pentan-1-yl)hept-4-en-1-ol *rac*-**19** (9.0 mg, 0.050 mmol, 1.0 equiv.), 4-(dimethylamino)benzoic acid (8.3 mg, 0.050 mmol, 1.0 equiv.), DMAP (18 mg, 0.15 mmol, 3.0 equiv.) and EDCI (29 mg, 0.15 mmol, 3.0 equiv.) in CH_2Cl_2 (0.5 mL) were submitted to General Procedure B for 23 h. Purification by column chromatography (SiO_2, pentane/Et_2O, 17:3) afforded the title compound as a clear oil (16 mg, 0.048 mmol, 95%).

R*f* 0.52 (pentane/Et_2O, 4:1), [UV, vanillin]
^{1}H NMR (500 MHz, $CDCl_3$) δ 7.91 (d, *J* = 9.1 Hz, 2H, Ar*H*), 6.68 (d, *J* = 9.1 Hz, 2H, Ar*H*), 5.46 – 5.31 (m, 2H, H7 and H8), 4.18 (dd, *J* = 10.9, 5.2 Hz, 1H, H1), 4.14 (dd, *J* = 10.9, 6.3 Hz, 1H, H1), 3.04 (s, 6H, H5), 2.47 (s, 1H, H4), 2.12 (dd, *J* = 7.5, 6.3 Hz, 2H, H6), 2.08 – 1.99 (m, 2H, H9), 1.88 (quin, *J* = 6.3 Hz, 1H, H2), 1.75 (s, 6H, H3), 0.93 (t, *J* = 7.5 Hz, 3H, H10).
^{13}C NMR (126 MHz, $CDCl_3$) δ 167.1, 153.2, 133.0, 131.4 (2C), 127.1, 117.8, 111.1 (2C), 65.3, 49.8 (3C), 47.3, 40.4 (2C), 39.8, 27.5, 26.7, 20.7, 14.3.
HRMS (ESI^+) Found $[M+H]^+$ = 328.2269; $C_{21}H_{30}O_2N$ requires 328.2271.
IR (film) ν_{max}/cm^{-1} 2962, 2907, 2870, 1703, 1609, 1527, 1367, 1317, 1275, 1183, 1109.

(*R*)-2-(Bicyclo[1.1.1]pentan-1-yl)-5-phenylpent-4-yn-1-ol, 20

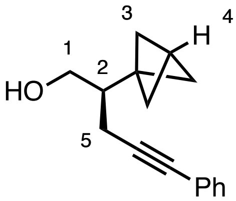

5-Phenylpent-4-ynal **S80** (48 mg, 0.30 mmol, 1.5 equiv.), Ir[$(ppy)_2$(dtbbpy)]PF_6 (3.7 mg, 4.0 µmol, 0.02 equiv.), 2,4,6-tri-*tert*-butylbenzenethiol **10** (5.6 mg, 0.020 mmol, 0.1 equiv.), (*S*)-α,α-bis[3,5-bis(trifluoromethyl)phenyl]-2-pyrrolidinemethanol trimethylsilyl

ether **9** (30 mg, 0.050 mmol, 0.25 equiv.), water (7.2 µL, 0.40 mmol, 2.0 equiv.) and TCP **1** (1.02 M in Et_2O, 0.20 mL, 0.20 mmol, 1.0 equiv.) in Et_2O (0.3 mL) and DME (0.5 mL), and sodium borohydride (38 mg, 1.0 mmol, 5.0 equiv.) in MeOH (1 mL) and CH_2Cl_2 (1 mL) were submitted to General Procedure A. Purification by column chromatography (SiO_2, pentane/Et_2O, 17:3) afforded the title compound as a yellow oil (23 mg, 0.10 mmol, 50%).

R*f* 0.38 (pentane/Et_2O, 3:2), [UV, vanillin]

^{1}H NMR (400 MHz, $CDCl_3$) δ 7.41 – 7.34 (m, 2H, Ar*H*), 7.32 – 7.26 (m, 3H, Ar*H*), 3.82 – 3.66 (m, 2H, H1), 2.56 – 2.38 (m, 3H, H4 and H5), 1.91 (quin, *J* = 6.3 Hz, 1H, H2), 1.80 (s, 6H, H3).

Note: the O*H* resonance was not observed.

^{13}C NMR (126 MHz, $CDCl_3$) δ 131.6 (2C), 128.4 (2C), 127.8, 123.9, 88.7, 82.1, 64.1, 49.9 (3C), 46.5, 41.6, 27.7, 19.2.

HRMS (ESI^+) Found $[M+H]^+$ = 227.1432; $C_{16}H_{19}O$ requires 227.1430.

IR (film) ν_{max}/cm^{-1} 3342, 2963, 2907, 2870, 1490, 1442, 1373, 1279, 1196, 1178, 1140.

$[\boldsymbol{\alpha}]_{\mathbf{D}}^{\mathbf{25}}$ – 23.6 (*c* = 0.2, $CHCl_3$)

HPLC 90% *ee* (CHIRALPAK IB, 6% IPA/hexane, 1.0 mL/min, t_R major – 6.9 min, minor – 6.1 min).

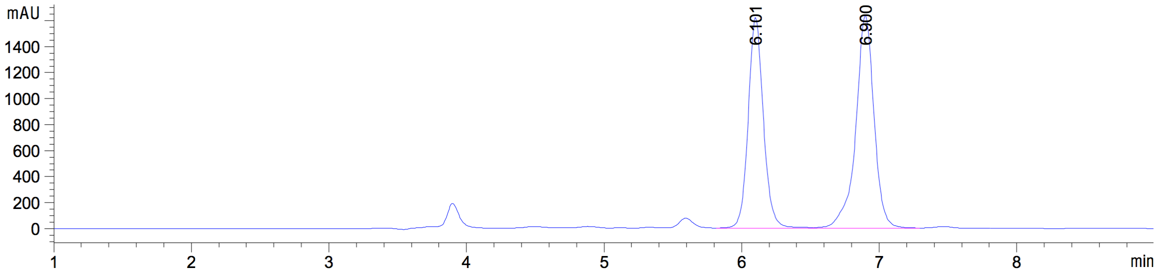

Peak #	RetTime [min]	Type	Width [min]	Area [mAU*s]	Height [mAU]	Area %
1	6.101	BV	0.1159	1.24551e4	1617.19202	45.1461
2	6.900	VV	0.1377	1.51333e4	1638.59021	54.8539

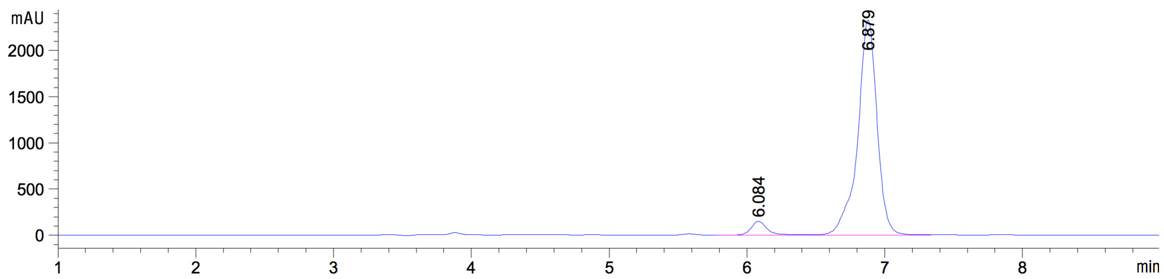

Peak #	RetTime [min]	Type	Width [min]	Area [mAU*s]	Height [mAU]	Area %
1	6.084	BV	0.1229	1210.75781	148.86757	5.0571
2	6.879	VV	0.1480	2.27310e4	2325.56519	94.9429

(*R*)-2-(Bicyclo[1.1.1]pentan-1-yl)-3-(methylthio)propan-1-ol, 21

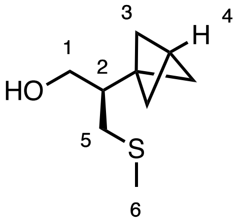

Methional (30 µL, 0.30 mmol, 1.5 equiv.), Ir[(ppy)$_2$(dtbbpy)]PF$_6$ (3.7 mg, 4.0 µmol, 0.02 equiv.), 2,4,6-tri-*tert*-butylbenzenethiol **10** (5.6 mg, 0.020 mmol, 0.1 equiv.), (*S*)-α,α-bis[3,5-bis(trifluoromethyl)phenyl]-2-pyrrolidinemethanol trimethylsilyl ether **9** (30 mg, 0.050 mmol, 0.25 equiv.), water (7.2 µL, 0.40 mmol, 2.0 equiv.) and TCP **1** (1.02 M in Et_2O, 0.20 mL, 0.20 mmol, 1.0 equiv.) in Et_2O (0.3 mL) and DME (0.5 mL), and sodium borohydride (38 mg, 1.0 mmol, 5.0 equiv.) in MeOH (1 mL) and CH_2Cl_2 (1 mL) were submitted to General Procedure A. Purification by column chromatography (SiO_2, pentane/Et_2O, 4:1) afforded the title compound as a pale yellow oil (34 mg, 0.20 mmol, 99%).

R*f* 0.43 (pentane/Et_2O, 3:2), [vanillin]

^{1}H NMR (400 MHz, $CDCl_3$) δ 3.73 (dd, *J* = 11.1, 4.7 Hz, 1H, H1), 3.65 (dd, *J* = 11.1, 6.3 Hz, 1H, H1), 2.60 (dd, *J* = 13.0, 4.7 Hz, 1H, H5), 2.49 (s, 1H, H4), 2.45 (dd, *J* = 13.0, 9.4 Hz, 1H, H5), 2.12 (s, 3H, H6), 1.86 (ddt, *J* = 9.4, 6.3, 4.7 Hz, 1H, H2), 1.73 (s, 6H, H3). Note: the O*H* resonance was not observed.

^{13}C NMR (126 MHz, $CDCl_3$) δ 64.5, 49.7 (3C), 46.5, 41.0, 34.6, 27.7, 16.3.

HRMS (ESI$^+$) Found $[M+H]^+$ = 173.0997; $C_9H_{17}OS$ requires 173.0995.

IR (film) ν_{max}/cm^{-1} 3404, 2962, 2909, 2869, 1426, 1279, 1213, 1197, 1139, 1110, 1063, 1018.

$[\alpha]_D^{25}$ – 10.1 (c = 0.2, $CHCl_3$)

HPLC 97% *ee* (CHIRALPAK IC, 8% IPA/hexane, 1.3 mL/min, t_R major – 15.1 min, minor – 15.9 min) after derivatization to (*R*)-2-(bicyclo[1.1.1]pentan-1-yl)-3-(methylthio)propyl 4-(dimethylamino)benzoate (*R*)-**S46**.

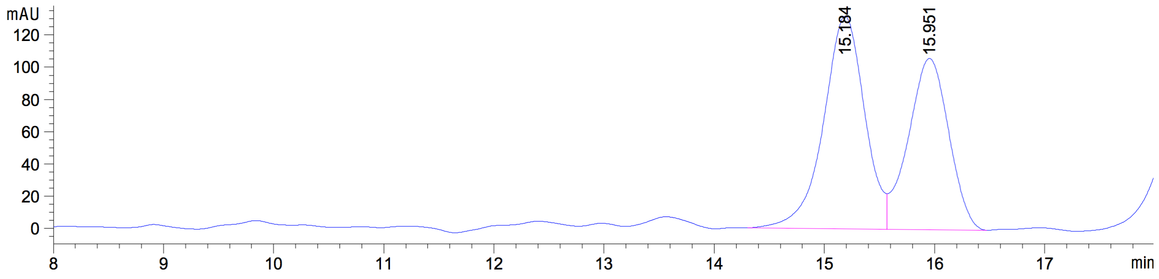

Peak #	RetTime [min]	Type	Width [min]	Area [mAU*s]	Height [mAU]	Area %
1	15.184	BV	0.3954	3425.91675	131.75049	55.8790
2	15.951	VB	0.3913	2705.04321	106.15359	44.1210

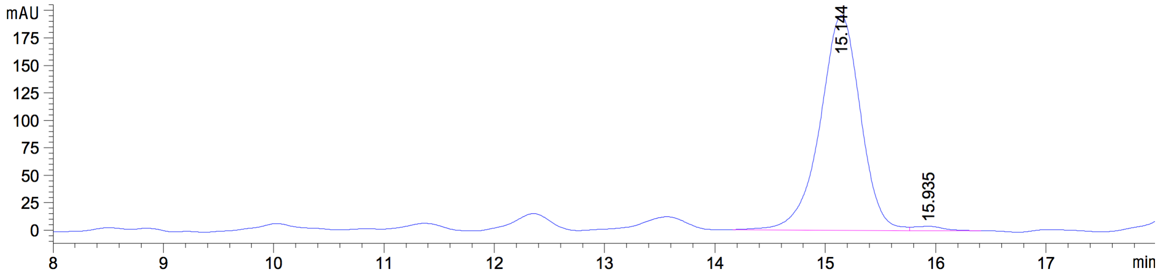

Peak #	RetTime [min]	Type	Width [min]	Area [mAU*s]	Height [mAU]	Area %
1	15.144	BV	0.3866	4968.80811	195.47777	98.4816
2	15.935	VB	0.2691	76.60919	3.97126	1.5184

Derivatization for *ee* determination

2-(Bicyclo[1.1.1]pentan-1-yl)-3-(methylthio)propyl 4-(dimethylamino)benzoate, S46

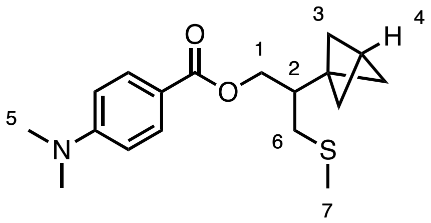

2-(Bicyclo[1.1.1]pentan-1-yl)-3-(methylthio)propan-1-ol *rac*-**21** (8.6 mg, 0.050 mmol, 1.0 equiv.), 4-(dimethylamino)benzoic acid (8.3 mg, 0.050 mmol, 1.0 equiv.), DMAP (18 mg, 0.15 mmol, 3.0 equiv.) and EDCI (29 mg, 0.15 mmol, 3.0 equiv.) in CH_2Cl_2 (0.5 mL) were submitted to General Procedure B for 22 h. Purification by column

chromatography (SiO_2, pentane/Et_2O, 4:1) afforded the title compound as a pale yellow oil (15 mg, 0.048 mmol, 96%).

R*f* 0.43 (pentane/Et_2O, 4:1), [UV, vanillin]
^{1}H NMR (500 MHz, $CDCl_3$) δ 7.93 (d, *J* = 8.9 Hz, 2H, Ar*H*), 6.78 (d, J = 8.9 Hz, 2H, Ar*H*), 4.33 (dd, *J* = 11.0, 5.1 Hz, 1H, H1), 4.31 (dd, *J* = 11.0, 5.7 Hz, 1H, H1), 3.06 (s, 6H, H5), 2.61 (dd, *J* = 13.1, 5.5 Hz, 1H, H6), 2.52 (dd, *J* = 13.1, 8.4 Hz, 1H, H6), 2.48 (s, 1H, H4), 2.10 (s, 3H, H7), 2.10 – 2.05 (m, 1H, H2), 1.78 (s, 6H, H3).
^{13}C NMR (126 MHz, $CDCl_3$) δ 166.8, 152.7, 131.5 (2C), 122.4, 111.9 (2C), 64.6, 49.9 (3C), 46.5, 40.9 (2C), 39.1, 34.0, 27.7, 16.6.
HRMS (ESI^+) Found $[M+H]^+$ = 320.1679; $C_{18}H_{26}O_2NS$ requires 320.1679.
IR (film) ν_{max}/cm^{-1} 2962, 2910, 2869, 1701, 1608, 1527, 1445, 1368, 1317, 1279, 1233, 1183, 1107.

Benzyl (*S*)-(2-(bicyclo[1.1.1]pentan-1-yl)-3-hydroxypropyl)carbamate, 11

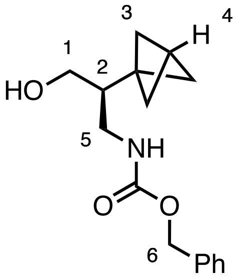

Benzyl (3-oxopropyl)carbamate **8** (62 mg, 0.30 mmol, 1.5 equiv.), Ir[$(ppy)_2$(dtbbpy)]PF_6 (3.7 mg, 4.0 µmol, 0.02 equiv.), 2,4,6-tri-*tert*-butylbenzene-thiol **10** (5.6 mg, 0.020 mmol, 0.1 equiv.), (*S*)-α,α-bis[3,5-bis(trifluoromethyl)phenyl]-2-pyrrolidinemethanol trimethylsilyl ether **9** (30 mg, 0.050 mmol, 0.25 equiv.), water (7.2 µL, 0.40 mmol, 2.0 equiv.) and TCP **1** (1.02 M in Et_2O, 0.20 mL, 0.20 mmol, 1.0 equiv.) in Et_2O (0.3 mL) and DME (0.5 mL), and sodium borohydride (38 mg, 1.0 mmol, 5.0 equiv.) in MeOH (1 mL) and CH_2Cl_2 (1 mL) were submitted to General Procedure A. Purification by column chromatography (SiO_2, pentane/Et_2O, 13:7) afforded the title compound as a pale yellow oil (52 mg, 0.19 mmol, 94%).

R*f* 0.40 (pentane/Et_2O, 1:4), [UV, vanillin]
^{1}H NMR (400 MHz, $CDCl_3$) δ 7.40 – 7.29 (m, 5H, Ar*H*), 5.11 (dd, *J* = 12.3, 2.4 Hz, 2H, H6), 5.04 (br s, 1H, N*H*), 3.63 (ddd, *J* = 11.4, 6.7, 4.2 Hz, 1H, H1), 3.49 (dt, *J* = 11.4, 6.7 Hz,

1H, H1), 3.40 (ddd, J = 14.2, 7.1, 4.3 Hz, 1H, H5), 3.19 (ddd, J = 14.2, 7.1, 5.9 Hz, 1H, H5), 2.74 (t, J = 6.7 Hz, 1H, OH), 2.48 (s, 1H, H4), 1.72 (s, 6H, H3), 1.74 – 1.67 (m, 1H, H2).

^{13}C NMR (126 MHz, $CDCl_3$) δ 157.6, 136.5, 128.7 (2C), 128.4, 128.3 (2C), 67.1, 61.8, 50.0 (3C), 45.3, 42.5, 40.3, 27.9.

HRMS (ESI^+) Found $[M+H]^+$ = 276.1596; $C_{16}H_{22}O_3N$ requires 276.1594.

IR (film) ν_{max}/cm^{-1} 3352, 2961, 2908, 2870, 1698, 1525, 1454, 1257.

$[\boldsymbol{\alpha}]_{\mathbf{D}}^{\mathbf{25}}$ – 58.1 (c = 0.1, $CHCl_3$)

HPLC 89% *ee* (CHIRALPAK IB, 4% IPA/hexane, 1.0 mL/min, t_R major – 23.7 min, minor – 27.5 min).

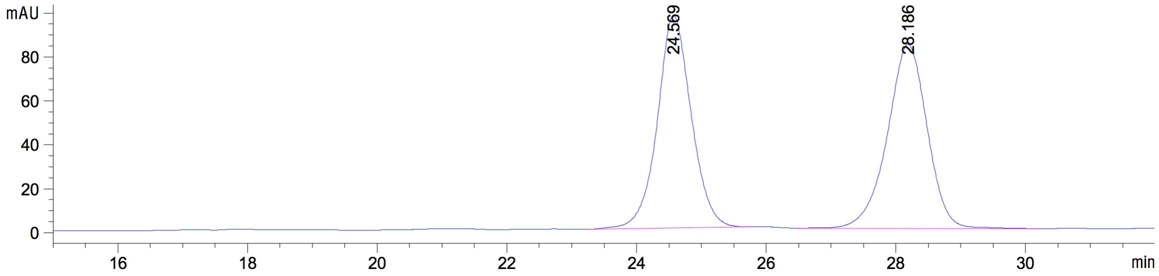

Peak #	RetTime [min]	Type	Width [min]	Area [mAU*s]	Height [mAU]	Area %
1	24.569	BB	0.5466	3524.77490	96.85780	49.5178
2	28.186	BB	0.6477	3593.42212	83.90495	50.4822

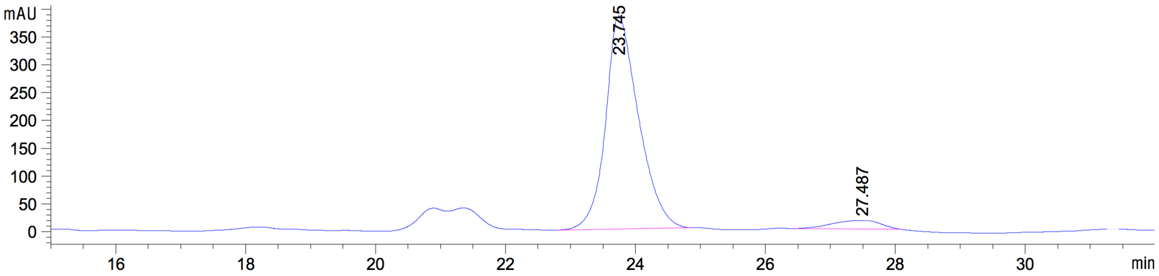

Peak #	RetTime [min]	Type	Width [min]	Area [mAU*s]	Height [mAU]	Area %
1	23.745	BB	0.5227	1.35612e4	383.38873	94.4829
2	27.487	MM	0.8549	791.86609	15.43752	5.5171

(*R*)-2-(Bicyclo[1.1.1]pentan-1-yl)-6-chlorohexan-1-ol, 22

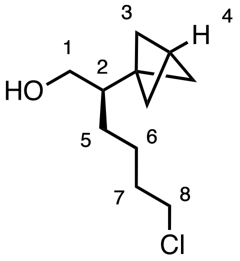

6-Chlorohexanal **S81** (40 mg, 0.30 mmol, 1.5 equiv.), Ir[(ppy)$_2$(dtbbpy)]PF$_6$ (3.7 mg, 4.0 µmol, 0.02 equiv.), 2,4,6-tri-*tert*-butylbenzenethiol **10** (5.6 mg, 0.020 mmol, 0.1 equiv.), (*S*)-α,α-bis[3,5-bis(trifluoromethyl)phenyl]-2-pyrrolidinemethanol trimethylsilyl ether **9** (30 mg, 0.050 mmol, 0.25 equiv.), water (7.2 µL, 0.40 mmol, 2.0 equiv.) and TCP **1** (1.02 M in Et_2O, 0.20 mL, 0.20 mmol, 1.0 equiv.) in Et_2O (0.3 mL) and DME (0.5 mL), and sodium borohydride (38 mg, 1.0 mmol, 5.0 equiv.) in MeOH (1 mL) and CH_2Cl_2 (1 mL) were submitted to General Procedure A. Purification by column chromatography (SiO_2, pentane/Et_2O, 4:1) afforded the title compound as a pale yellow oil (40 mg, 0.20 mmol, 98%).

R*f* 0.24 (pentane/Et_2O, 4:1), [vanillin]

^{1}H NMR (500 MHz, $CDCl_3$) δ 3.63 – 3.49 (m, 4H, H1 and H8), 2.49 (s, 1H, H4), 1.81 – 1.75 (m, 2H, H7), 1.72 (d, *J* = 1.6 Hz, 6H, H3), 1.53 – 1.45 (m, 3H, H2 and H6), 1.34 – 1.27 (m, 2H, H5).

Note: the O*H* resonance was not observed.

^{13}C NMR (126 MHz, $CDCl_3$) δ 64.5, 49.8 (3C), 47.4, 45.1, 42.0, 33.1, 27.9, 27.7, 25.1.

HRMS (ESI$^+$) Found $[M+H]^+$ = 203.1198; $C_{11}H_{20}O^{35}Cl$ requires 203.1197.

IR (film) ν_{max}/cm^{-1} 3337, 2960, 2869, 1447, 1279, 1198, 1140.

$[\alpha]_D^{25}$ – 25.8 (*c* = 0.2, $CHCl_3$)

HPLC 86% *ee* (CHIRALPAK IB, 3% IPA/hexane, 1.0 mL/min, t_R major – 8.8 min, minor – 9.4 min) after derivatization to (*R*)-2-(bicyclo[1.1.1]pentan-1-yl)-6-chlorohexyl 4-(dimethylamino)benzoate (*R*)-**S47**.

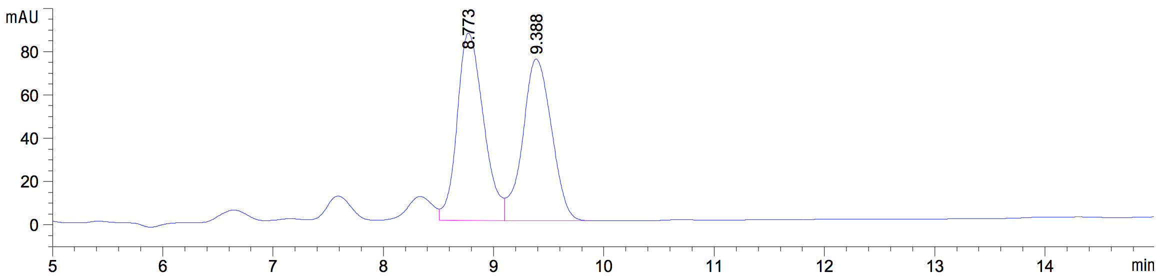

Peak #	RetTime [min]	Type	Width [min]	Area [mAU*s]	Height [mAU]	Area %
1	8.773	VV	0.2582	1440.46741	86.54997	51.4686
2	9.388	VB	0.2844	1358.26331	74.60453	48.5314

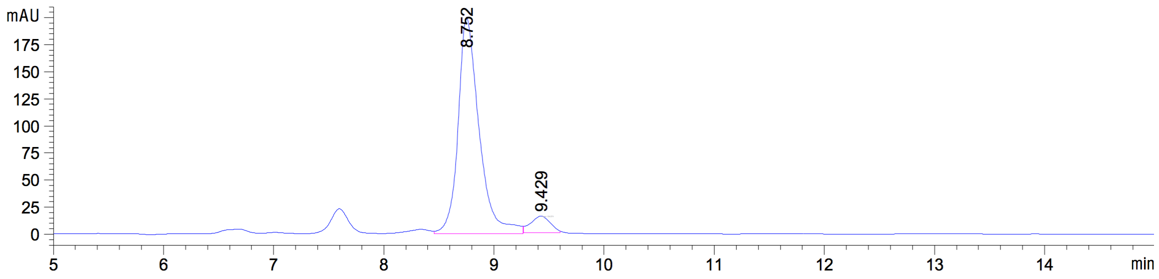

Peak #	RetTime [min]	Type	Width [min]	Area [mAU*s]	Height [mAU]	Area %
1	8.752	VV	0.1946	2647.57764	199.79578	93.1442
2	9.429	MM	0.2083	194.87386	15.59131	6.8558

Derivatization for *ee* determination

2-(Bicyclo[1.1.1]pentan-1-yl)-6-chlorohexyl 4-(dimethylamino)benzoate, S47

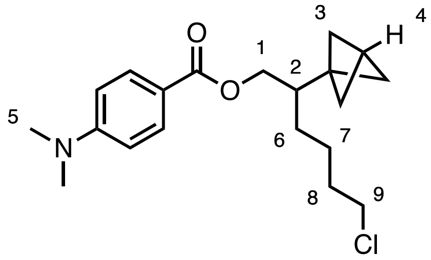

2-(Bicyclo[1.1.1]pentan-1-yl)-6-chlorohexan-1-ol *rac*-**22** (10 mg, 0.050 mmol, 1.0 equiv.), 4-(dimethylamino)benzoic acid (8.3 mg, 0.050 mmol, 1.0 equiv.), DMAP (18 mg, 0.15 mmol, 3.0 equiv.) and EDCI (29 mg, 0.15 mmol, 3.0 equiv.) in CH_2Cl_2 (0.5 mL) were submitted to General Procedure B for 25 h. Purification by column chromatography (SiO_2, pentane/Et_2O, 4:1) afforded the title compound as a clear oil (16 mg, 0.047 mmol, 93%).

R*f* 0.38 (pentane/Et_2O, 4:1), [UV, vanillin]

^{1}H NMR (500 MHz, $CDCl_3$) δ 7.90 (d, *J* = 9.1 Hz, 2H, Ar*H*), 6.68 (d, *J* = 9.1 Hz, 2H, Ar*H*), 4.22 – 4.13 (m, 2H, H1), 3.53 (t, *J* = 6.7 Hz, 2H, H9), 3.04 (s, 6H, H5), 2.47 (s, 1H, H4), 1.86 – 1.77 (m, 3H, H2 and H8), 1.75 (s, 6H, H3), 1.53 – 1.47 (m, 2H, H7), 1.41 – 1.33 (m, 2H, H6).

^{13}C NMR (126 MHz, $CDCl_3$) δ 167.1, 153.3, 131.4 (2C), 117.7, 111.1 (2C), 65.5, 49.8 (3C), 47.3, 45.1, 40.4 (2C), 39.1, 33.0, 28.3, 27.5, 25.0.

HRMS (ESI^+) Found $[M+H]^+$ = 350.1882; $C_{20}H_{29}O_2N^{35}Cl$ requires 350.1881.

IR (film) ν_{max}/cm^{-1} 2961, 2868, 1702, 1608, 1527, 1446, 1367, 1317, 1279, 1183, 1109.

Ethyl (*R*)-5-(bicyclo[1.1.1]pentan-1-yl)-6-hydroxyhexanoate, 23

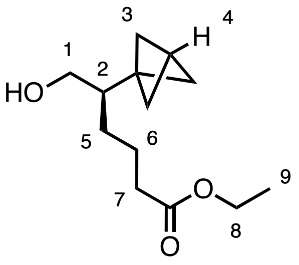

Ethyl 6-oxohexanoate **S82** (48 mg, 0.30 mmol, 1.5 equiv.), Ir[(ppy)$_2$(dtbbpy)]PF_6 (3.7 mg, 4.0 µmol, 0.02 equiv.), 2,4,6-tri-*tert*-butylbenzenethiol **10** (5.6 mg, 0.020 mmol, 0.1 equiv.), (*S*)-α,α-bis[3,5-bis(trifluoromethyl)phenyl]-2-pyrrolidinemethanol trimethylsilyl ether **9** (30 mg, 0.050 mmol, 0.25 equiv.), water (7.2 µL, 0.40 mmol, 2.0 equiv.) and TCP **1** (1.02 M in Et_2O, 0.20 mL, 0.20 mmol, 1.0 equiv.) in Et_2O (0.3 mL) and DME (0.5 mL), and sodium borohydride (38 mg, 1.0 mmol, 5.0 equiv.) in MeOH (1 mL) and CH_2Cl_2 (1 mL) were submitted to General Procedure A. Purification by column chromatography (SiO_2, pentane/Et_2O, 3:2) afforded the title compound as a pale yellow oil (44 mg, 0.19 mmol, 97%).

R*f* 0.07 (pentane/Et_2O, 4:1), [vanillin]

^{1}H NMR (500 MHz, $CDCl_3$) δ 4.13 (q, *J* = 7.1 Hz, 2H, H8), 3.60 (dd, *J* = 10.8, 5.7 Hz, 1H, H1), 3.53 (dd, *J* = 10.8, 5.7 Hz, 1H, H1), 2.48 (s, 1H, H4), 2.30 (t, *J* = 7.3 Hz, 2H, H7), 1.71 (s, 6H, H3), 1.68 – 1.63 (m, 2H, H6), 1.55 – 1.50 (m, 1H, H2), 1.34 – 1.28 (m, 2H, H5), 1.26 (t, *J* = 7.1 Hz, 3H, H9).

Note: the O*H* resonance was not observed.

^{13}C NMR (126 MHz, $CDCl_3$) δ 173.9, 64.3, 60.4, 49.8 (3C), 47.3, 41.9, 34.7, 28.0, 27.7, 23.0, 14.4.

HRMS (ESI^+) Found $[M+Na]^+$ = 249.1463; $C_{13}H_{22}O_3Na$ requires 249.1461.

IR (film) ν_{max}/cm^{-1} 2962, 2908, 2869, 1736, 1449, 1374, 1279.

$[\boldsymbol{\alpha}]_{\mathbf{D}}^{\mathbf{25}}$ – 26.1 (c = 0.2, $CHCl_3$)

HPLC 92% *ee* (Lux i-Amylose-1, 3% IPA/hexane, 1.0 mL/min, t_R major – 21.9 min, minor – 23.6 min) after derivatization to (*R*)-2-(bicyclo[1.1.1]pentan-1-yl)-6-ethoxy-6-oxohexyl 4-(dimethylamino)benzoate (*R*)-**S48**.

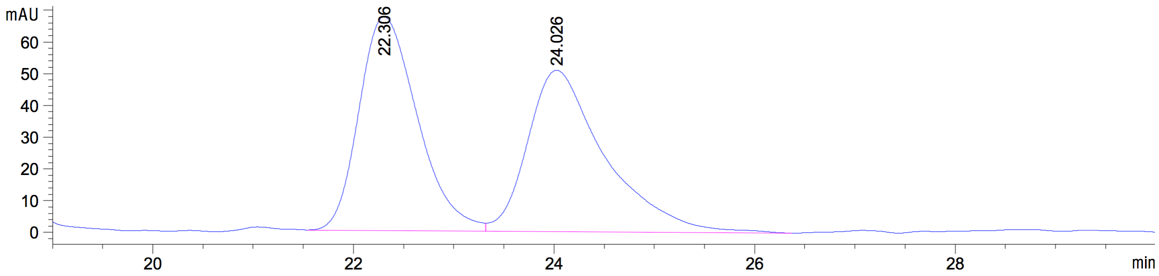

Peak #	RetTime [min]	Type	Width [min]	Area [mAU*s]	Height [mAU]	Area %
1	22.306	BV	0.6241	2726.98193	67.38924	50.0242
2	24.026	VB	0.7814	2724.33911	50.82434	49.9758

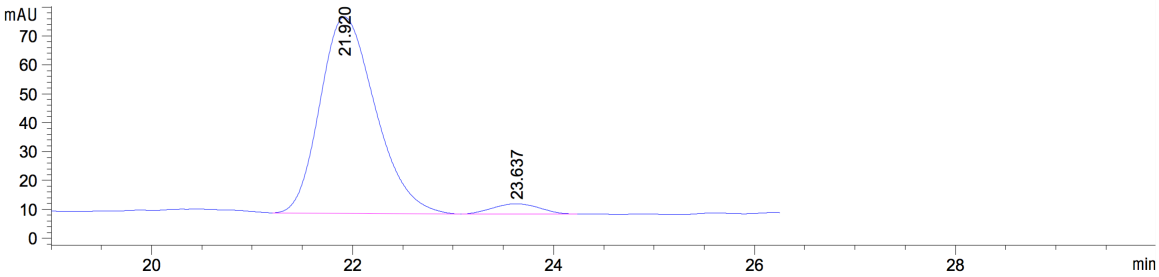

Peak #	RetTime [min]	Type	Width [min]	Area [mAU*s]	Height [mAU]	Area %
1	21.920	BB	0.5914	2607.46069	67.98797	95.7974
2	23.637	BB	0.4122	114.38885	3.54702	4.2026

Derivatization for *ee* determination

2-(Bicyclo[1.1.1]pentan-1-yl)-6-ethoxy-6-oxohexyl 4-(dimethylamino)benzoate, S48

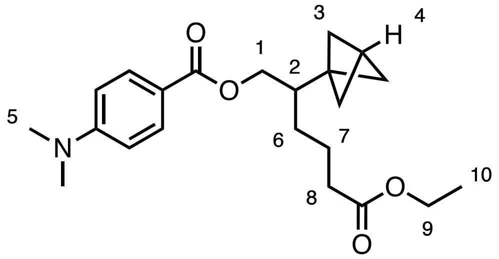

Ethyl 5-(bicyclo[1.1.1]pentan-1-yl)-6-hydroxyhexanoate *rac*-**23** (11 mg, 0.050 mmol, 1.0 equiv.), 4-(dimethylamino)benzoic acid (8.3 mg, 0.050 mmol, 1.0 equiv.), DMAP (18 mg, 0.15 mmol, 3.0 equiv.) and EDCI (29 mg, 0.15 mmol, 3.0 equiv.) in CH_2Cl_2 (0.5 mL) were submitted to General Procedure B for 25 h. Purification by column chromatography (SiO_2, pentane/Et_2O, 3:2) afforded the title compound as a pale yellow oil (17 mg, 0.045 mmol, 89%).

R*f* 0.17 (pentane/Et_2O, 4:1), [UV, vanillin]

^{1}H NMR (500 MHz, $CDCl_3$) δ 7.90 (d, *J* = 9.0 Hz, 2H, Ar*H*), 6.69 (d, *J* = 9.0 Hz, 2H, Ar*H*), 4.20 – 4.15 (m, 2H, H1), 4.12 (q, *J* = 7.1 Hz, 2H, H9), 3.04 (s, 6H, H5), 2.47 (s, 1H, H4), 2.30 (t, *J* = 7.4 Hz, 2H, H8), 1.85 – 1.79 (m, 1H, H2), 1.74 (s, 6H, H3), 1.73 – 1.66 (m, 2H, H7), 1.43 – 1.31 (m, 2H, H6), 1.24 (t, *J* = 7.1 Hz, 3H, H10).

^{13}C NMR (126 MHz, $CDCl_3$) δ 173.7, 167.1, 153.2, 131.4 (2C), 117.9, 111.2 (2C), 65.5, 60.4, 49.8 (3C), 47.3, 40.4 (2C), 39.0, 34.8, 28.6, 27.5, 23.2, 14.4.

HRMS (ESI$^+$) Found $[M+H]^+$ = 374.2323; $C_{22}H_{32}O_4N$ requires 374.2326.

IR (film) ν_{max}/cm^{-1} 2962, 2909, 2869, 1734, 1702, 1608, 1528, 1368, 1317, 1278, 1233, 1183, 1109.

(*R*)-5-(Benzyloxy)-2-(bicyclo[1.1.1]pentan-1-yl)pentan-1-ol, 24

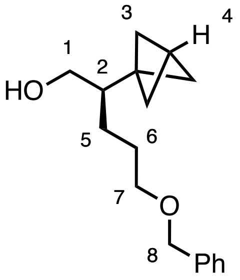

5-(Benzyloxy)pentanal **S84** (58 mg, 0.30 mmol, 1.5 equiv.), Ir[(ppy)$_2$(dtbbpy)]PF$_6$ (3.7 mg, 4.0 μmol, 0.02 equiv.), 2,4,6-tri-*tert*-butylbenzenethiol **10** (5.6 mg, 0.020 mmol, 0.1 equiv.), (*S*)-α,α-bis[3,5-bis(trifluoromethyl)phenyl]-2-pyrrolidinemethanol trimethylsilyl ether **9** (30 mg, 0.050 mmol, 0.25 equiv.), water (7.2 μL, 0.40 mmol, 2.0 equiv.) and TCP **1** (1.02 M in Et$_2$O, 0.20 mL, 0.20 mmol, 1.0 equiv.) in Et$_2$O (0.3 mL) and DME (0.5 mL), and sodium borohydride (38 mg, 1.0 mmol, 5.0 equiv.) in MeOH (1 mL) and CH$_2$Cl$_2$ (1 mL) were submitted to General Procedure A. Purification by column chromatography (SiO$_2$, pentane/Et$_2$O, 3:2) afforded the title compound as a pale yellow oil (44 mg, 0.17 mmol, 85%).

R*f* 0.17 (pentane/Et$_2$O, 3:2), [UV, vanillin]

^{1}H NMR (500 MHz, CDCl$_3$) δ 7.37 – 7.27 (m, 5H, Ar*H*), 4.50 (s, 2H, H8), 3.63 – 3.49 (m, 2H, H1), 3.46 (t, *J* = 6.5 Hz, 2H, H7), 2.48 (s, 1H, H4), 1.71 (s, 6H, H3), 1.69 – 1.63 (m, 2H, H6), 1.56 – 1.51 (m, 1H, H2), 1.44 – 1.34 (m, 2H, H5).

Note: the O*H* resonance was not observed.

^{13}C NMR (126 MHz, CDCl$_3$) δ 138.7, 128.5 (2C), 127.8 (2C), 127.7, 73.1, 70.8, 64.5, 49.8 (3C), 47.5, 41.9, 27.8, 27.7, 25.1.

HRMS (ESI$^+$) Found [M+Na]$^+$ = 283.1668; C$_{17}$H$_{24}$O$_2$Na requires 283.1669.

IR (film) ν_{max}/cm^{-1} 3397, 2959, 2907, 2867, 1454, 1363, 1278, 1197, 1099.

$[\alpha]_D^{25}$ – 14.5 (*c* = 0.4, CHCl$_3$)

HPLC 91% *ee* (CHIRALPAK IB, 2% IPA/hexane, 1.0 mL/min, t_R major – 12.6 min, minor – 11.7 min).

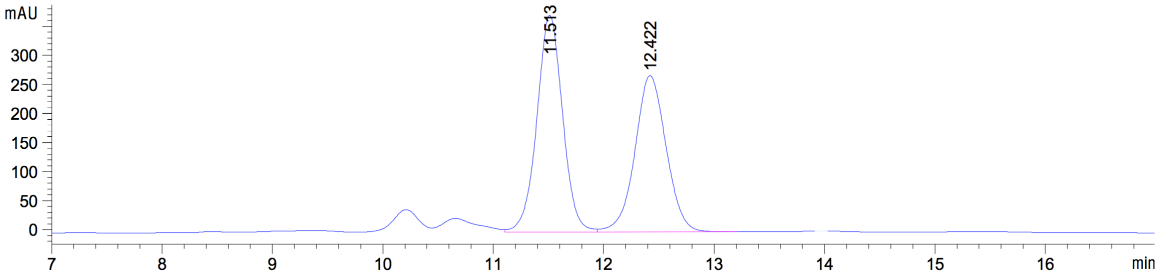

Peak #	RetTime [min]	Type	Width [min]	Area [mAU*s]	Height [mAU]	Area %
1	11.513	VV	0.2468	6032.84424	372.69876	53.3460
2	12.422	VB	0.3011	5276.04639	268.66055	46.6540

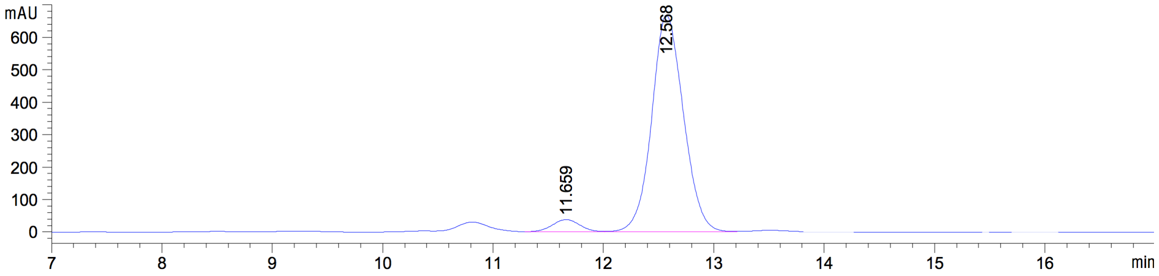

Peak #	RetTime [min]	Type	Width [min]	Area [mAU*s]	Height [mAU]	Area %
1	11.659	BV	0.2690	643.61969	37.34913	4.6101
2	12.568	VV	0.3070	1.33174e4	666.92151	95.3899

***tert*-Butyl (*R*)-4-(1-(bicyclo[1.1.1]pentan-1-yl)-2-hydroxyethyl)piperidine-1-carboxylate, 25**

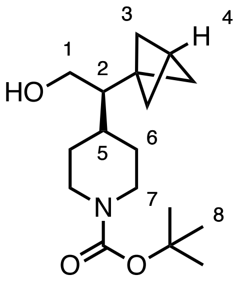

N-Boc-4-piperidineacetaldehyde (68 mg, 0.30 mmol, 1.5 equiv.), Ir[(ppy)$_2$(dtbbpy)]PF$_6$ (3.7 mg, 4.0 µmol, 0.02 equiv.), 2,4,6-tri-*tert*-butylbenzene-thiol **10** (5.6 mg, 0.020 mmol, 0.1 equiv.), (*S*)-α,α-bis[3,5-bis(trifluoromethyl)phenyl]-2-pyrrolidinemethanol trimethylsilyl ether **9** (30 mg, 0.050 mmol, 0.25 equiv.), water (7.2 µL, 0.40 mmol, 2.0 equiv.) and TCP **1** (1.02 M in Et$_2$O, 0.20 mL, 0.20 mmol, 1.0 equiv.) in Et$_2$O (0.3 mL) and DME (0.5 mL), and sodium borohydride (38 mg, 1.0 mmol, 5.0 equiv.) in MeOH (1 mL) and CH$_2$Cl$_2$ (1 mL) were submitted to General Procedure A. Purification by column chromatography (SiO$_2$, pentane/Et$_2$O, 9:11) afforded the title compound as a pale yellow oil (49 mg, 0.17 mmol, 83%).

R$_f$ 0.26 (pentane/Et_2O, 2:3), [vanillin]

^{1}H NMR (500 MHz, $CDCl_3$) δ 4.19 – 4.07 (br m, 2H, H7), 3.67 (dd, J = 10.8, 5.8 Hz, 1H, H1), 3.62 (dd, J = 10.8, 5.6 Hz, 1H, H1), 2.69 – 2.58 (br m, 2H, H7), 2.48 (s, 1H, H4), 1.79 (s, 6H, H3), 1.65 – 1.60 (m, 3H, H5 and H6), 1.45 (s, 10H, H2 and H8), 1.38 (dd, J = 12.6, 4.4 Hz, 1H, H6), 1.28 (dd, J = 12.6, 4.4 Hz, 1H, H6).

Note: the O*H* resonance was not observed.

^{13}C NMR (126 MHz, $CDCl_3$) δ 155.0, 79.4, 62.6, 51.3 (3C), 47.1, 46.8, 44.6 (br, 2C), 36.9, 30.6 (br), 29.4 (br), 28.6 (3C), 28.2.

HRMS (ESI^+) Found $[M+Na]^+$ = 318.2040; $C_{17}H_{29}O_3NNa$ requires 318.2040.

IR (film) ν_{max}/cm^{-1} 3437, 2962, 2925, 2868, 1694, 1672, 1477, 1426, 1366, 1279, 1241, 1171.

$[\boldsymbol{\alpha}]_{\mathbf{D}}^{\mathbf{25}}$ – 21.4 (c = 0.2, $CHCl_3$)

HPLC 97% *ee* (Lux i-Amylose-1, 10% IPA/hexane, 1.3 mL/min, t_R major – 11.1 min, minor – 12.0 min) after derivatization to *tert*-butyl (*R*)-4-(1-(bicyclo[1.1.1]pentan-1-yl)-2-((4-(dimethylamino)benzoyl)oxy)ethyl)piperidine-1-carboxylate (*R*)-**S49**.

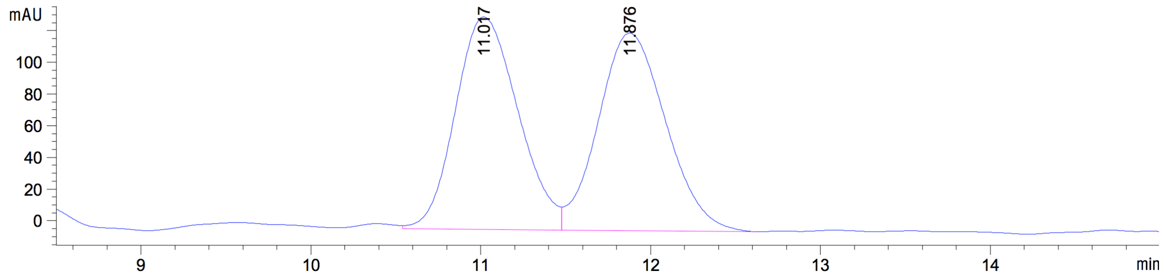

Peak #	RetTime [min]	Type	Width [min]	Area [mAU*s]	Height [mAU]	Area %
1	11.017	VV	0.4030	3434.12524	134.01428	49.8843
2	11.876	VB	0.4330	3450.05664	124.60506	50.1157

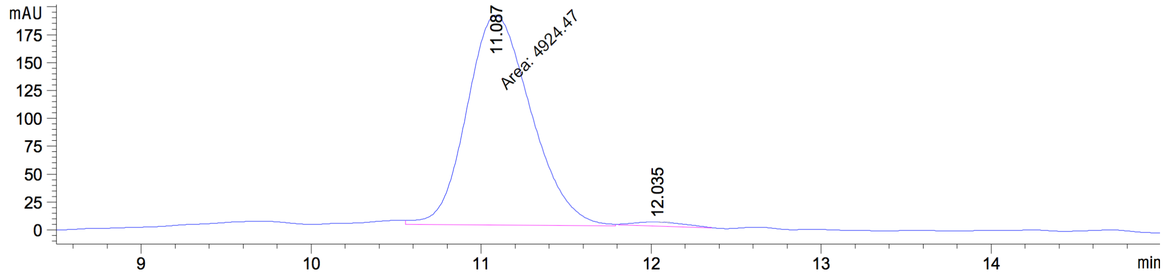

Peak #	RetTime [min]	Type	Width [min]	Area [mAU*s]	Height [mAU]	Area %
1	11.087	MM	0.4367	4924.47266	187.93063	98.4584
2	12.035	MM	0.3392	77.10249	3.78795	1.5416

Derivatization for *ee* determination

***tert*-Butyl 4-(1-(bicyclo[1.1.1]pentan-1-yl)-2-((4-(dimethylamino)benzoyl)oxy)ethyl)piperidine-1-carboxylate, S49**

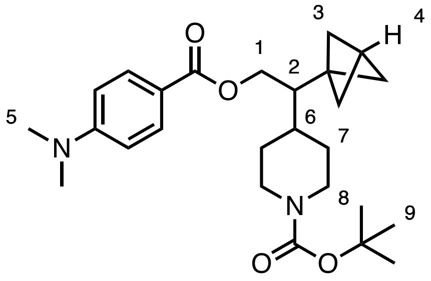

tert-Butyl 4-(1-(bicyclo[1.1.1]pentan-1-yl)-2-hydroxyethyl)piperidine-1-carboxylate *rac*-**25** (15 mg, 0.050 mmol, 1.0 equiv.), 4-(dimethylamino)benzoic acid (8.3 mg, 0.050 mmol, 1.0 equiv.), DMAP (18 mg, 0.15 mmol, 3.0 equiv.) and EDCI (29 mg, 0.15 mmol, 3.0 equiv.) in CH_2Cl_2 (0.5 mL) were submitted to General Procedure B for 19 h. Purification by column chromatography (SiO_2, pentane/Et_2O, 1:1) afforded the title compound as a clear oil (17 mg, 0.039 mmol, 77%).

R*f* 0.36 (pentane/Et_2O, 1:1), [UV, vanillin]

^{1}H NMR (500 MHz, $CDCl_3$) δ 7.89 (d, *J* = 9.0 Hz, 2H, Ar*H*), 6.67 (d, *J* = 9.0 Hz, 2H, Ar*H*), 4.23 (d, *J* = 5.7 Hz, 2H, H1), 4.12 (br s, 2H, H8), 3.04 (s, 6H, H5), 2.65 (br s, 2H, H8), 2.46 (s, 1H, H4), 1.82 (s, 6H, H3), 1.76 – 1.72 (m, 1H, H2), 1.69 – 1.65 (m, 1H, H6), 1.59 – 1.51 (m, 2H, H7), 1.45 (s, 9H, H9), 1.39 – 1.27 (m, 2H, H7).

^{13}C NMR (126 MHz, $CDCl_3$) δ 167.1, 155.0, 153.4, 131.4 (2C), 117.4, 111.0 (2C), 79.4, 63.9, 51.2 (3C), 47.0, 45.1 (br), 45.0 (br), 43.7, 40.3 (2C), 37.4, 30.9 (br), 29.1 (br), 28.6 (3C), 28.0.

HRMS (ESI$^+$) Found $[M+Na]^+$ = 465.2723; $C_{26}H_{38}O_4N_2Na$ requires 465.2724.

IR (film) ν_{max}/cm^{-1} 2963, 2908, 2868, 1693, 1607, 1528, 1423, 1391, 1276, 1181.

(*R*)-2-(Bicyclo[1.1.1]pentan-1-yl)-4-(5,5-dimethyl-1,3-dioxan-2-yl)butan-1-ol, 26

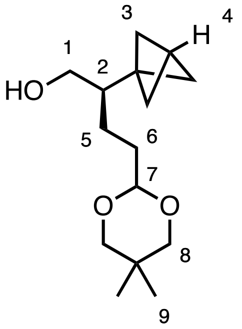

5,5-Dimethyl-1,3-dioxane-2-butanal (56 mg, 0.30 mmol, 1.5 equiv.), Ir[(ppy)$_2$(dtbbpy)]PF$_6$ (3.7 mg, 4.0 μmol, 0.02 equiv.), 2,4,6-tri-*tert*-butylbenzene-thiol **10** (5.6 mg, 0.020 mmol, 0.1 equiv.), (*S*)-α,α-bis[3,5-bis(trifluoromethyl)phenyl]-2-pyrrolidinemethanol trimethylsilyl ether **9** (30 mg, 0.050 mmol, 0.25 equiv.), water (7.2 μL, 0.40 mmol, 2.0 equiv.) and TCP **1** (1.02 M in Et_2O, 0.20 mL, 0.20 mmol, 1.0 equiv.) in Et_2O (0.3 mL) and DME (0.5 mL), and sodium borohydride (38 mg, 1.0 mmol, 5.0 equiv.) in MeOH (1 mL) and CH_2Cl_2 (1 mL) were submitted to General Procedure A. The reduction was quenched by addition of water (2 mL) instead of NH_4Cl. Purification by column chromatography (SiO_2, pentane/Et_2O, 11:9) afforded the title compound as a pale yellow oil (50 mg, 0.20 mmol, 99%).

R*f* 0.36 (pentane/Et_2O, 1:1), [vanillin]

^{1}H NMR (400 MHz, $CDCl_3$) δ 4.41 (t, *J* = 4.9 Hz, 1H, H7), 3.63 – 3.57 (m, 3H, H1 and H8), 3.53 (dd, *J* = 10.9, 5.6 Hz, 1H, H1), 3.42 (d, *J* = 10.9 Hz, 2H, H8), 2.47 (s, 1H, H4), 1.71 (s, 6H, H3), 1.71 – 1.66 (m, 2H, H6), 1.58 – 1.52 (m, 1H, H2), 1.47 – 1.35 (m, 2H, H5), 1.19 (s, 3H, H9), 0.72 (s, 3H, H9).

Note: the O*H* resonance was not observed.

^{13}C NMR (126 MHz, $CDCl_3$) δ 102.6, 77.4 (2C; obscured by solvent signal), 64.3, 49.8 (3C), 47.4, 42.0, 32.6, 30.3, 27.7, 23.1, 22.6, 22.0.

HRMS (ESI$^+$) Found $[M+Na]^+$ = 277.1775; $C_{15}H_{26}O_3Na$ requires 277.1774.

IR (film) ν_{max}/cm^{-1} 3426, 2957, 2907, 2868, 1471, 1394, 1196, 1136.

$[\alpha]_D^{25}$ – 32.9 (*c* = 0.2, $CHCl_3$)

HPLC 94% *ee* (Lux i-Amylose-1, 1% IPA/hexane, 1.3 mL/min, t_R major – 28.1 min, minor – 30.5 min) after derivatization to (*R*)-2-(bicyclo[1.1.1]pentan-1-yl)-4-(5,5-dimethyl-1,3-dioxan-2-yl)butyl 4-(dimethylamino)benzoate (*R*)-**S50**.

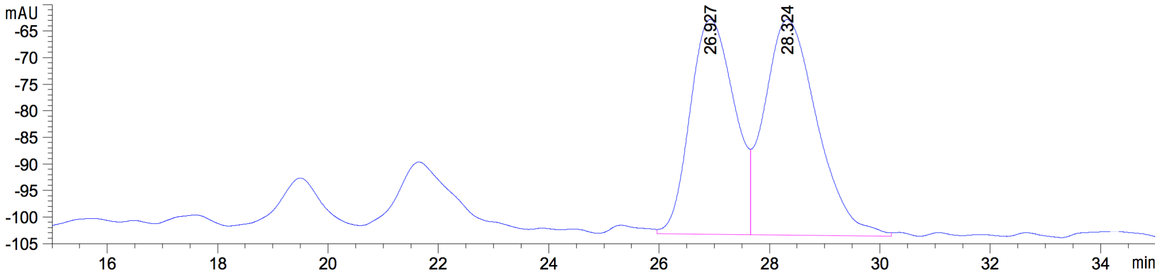

Peak #	RetTime [min]	Type	Width [min]	Area [mAU*s]	Height [mAU]	Area %
1	26.927	VV	0.8465	2254.93286	40.44193	45.5579
2	28.324	VV	0.9768	2694.66943	40.57485	54.4421

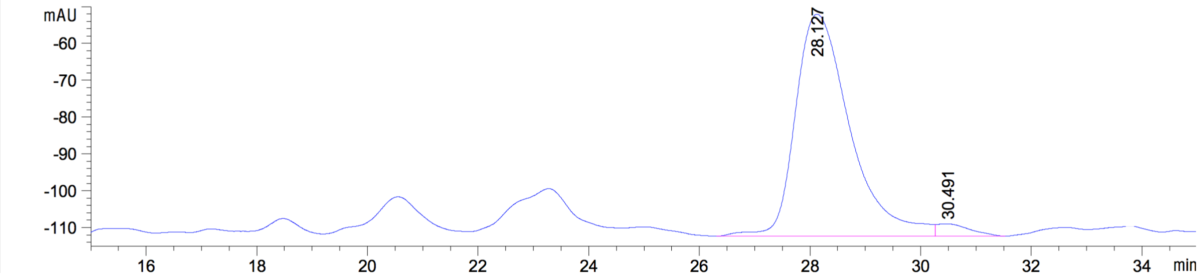

Peak #	RetTime [min]	Type	Width [min]	Area [mAU*s]	Height [mAU]	Area %
1	28.127	BV	1.0321	4144.08887	60.27647	96.7993
2	30.491	VB	0.4909	137.02623	3.35194	3.2007

Derivatization for *ee* determination

2-(Bicyclo[1.1.1]pentan-1-yl)-4-(5,5-dimethyl-1,3-dioxan-2-yl)butyl 4-(dimethylamino)benzoate, S50

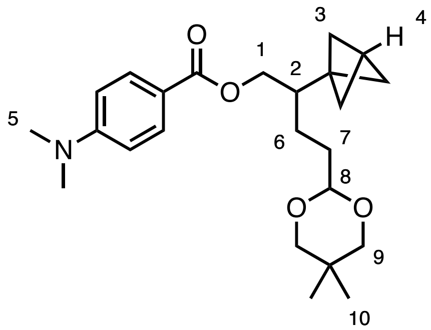

2-(Bicyclo[1.1.1]pentan-1-yl)-4-(5,5-dimethyl-1,3-dioxan-2-yl)butan-1-ol *rac*-**26** (13 mg, 0.050 mmol, 1.0 equiv.), 4-(dimethylamino)benzoic acid (8.3 mg, 0.050 mmol, 1.0 equiv.), DMAP (18 mg, 0.15 mmol, 3.0 equiv.) and EDCI (29 mg, 0.15 mmol, 3.0 equiv.) in CH_2Cl_2 (0.5 mL) were submitted to General Procedure B for 15 h. Purification by column chromatography (SiO_2, pentane/Et_2O, 3:2) afforded the title compound as a pale yellow oil (20 mg, 0.050 mmol, 99%).

R$_f$ 0.31 (pentane/Et_2O, 3:2), [UV, vanillin]

^{1}H NMR (500 MHz, $CDCl_3$) δ 7.91 (d, *J* = 8.7 Hz, 2H, Ar*H*), 6.69 (d, *J* = 8.7 Hz, 2H, Ar*H*), 4.40 (t, *J* = 5.0 Hz, 1H, H8), 4.17 (d, *J* = 5.6 Hz, 2H, H1), 3.59 (d, *J* = 11.1 Hz, 2H, H9), 3.40 (d, *J* = 11.1 Hz, 2H, H9), 3.04 (s, 6H, H5), 2.45 (s, 1H, H4), 1.85 – 1.78 (m, 1H, H2), 1.75 (s, 6H, H3), 1.74 – 1.69 (m, 2H, H7), 1.59 – 1.56 (m, 1H, H6), 1.48 – 1.43 (m, 1H, H6), 1.18 (s, 3H, H10), 0.71 (s, 3H, H10).

^{13}C NMR (126 MHz, $CDCl_3$) δ 167.1, 153.1, 131.4 (2C), 118.0, 111.2 (2C), 102.5, 77.4 (2C; signal partially obscured by solvent signal), 65.6, 49.8 (3C), 47.4, 40.4 (2C), 39.1, 33.0, 30.3, 27.5, 23.4, 23.2, 22.0.

HRMS (ESI$^+$) Found $[M+H]^+$ = 402.2636; $C_{24}H_{36}O_4N$ requires 402.2639.

IR (film) ν_{max}/cm^{-1} 2956, 2868, 1702, 1608, 1527, 1469, 1366, 1279, 1183, 1109.

(*R*)-2-(Bicyclo[1.1.1]pentan-1-yl)-3-(5-methylfuran-2-yl)propan-1-ol, 27

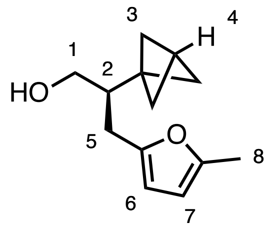

3-(5-Methyl-2-furyl)propionaldehyde (40 µL, 0.30 mmol, 1.5 equiv.), Ir[(ppy)$_2$(dtbbpy)]PF_6 (3.7 mg, 4.0 µmol, 0.02 equiv.), 2,4,6-tri-*tert*-butylbenzene-thiol **10** (5.6 mg, 0.020 mmol, 0.1 equiv.), (*S*)-α,α-bis[3,5-bis(trifluoromethyl)phenyl]-2-pyrrolidinemethanol trimethylsilyl ether **9** (30 mg, 0.050 mmol, 0.25 equiv.), water (7.2 µL, 0.40 mmol, 2.0 equiv.) and TCP **1** (1.02 M in Et_2O, 0.20 mL, 0.20 mmol, 1.0 equiv.) in Et_2O (0.3 mL) and DME (0.5 mL), and sodium borohydride (38 mg, 1.0 mmol, 5.0 equiv.) in MeOH (1 mL) and CH_2Cl_2 (1 mL) were submitted to General Procedure A. Purification by column chromatography (SiO_2, pentane/Et_2O, 17:3) afforded the title compound as a pale yellow oil (37 mg, 0.18 mmol, 89%).

R$_f$ 0.07 (pentane/Et_2O, 9:1), [UV, vanillin]

^{1}H NMR (400 MHz, $CDCl_3$) δ 5.88 (d, *J* = 3.0 Hz, 1H, H6), 5.85 – 5.82 (m, 1H, H7), 3.62 – 3.53 (m, 2H, H1), 2.62 (dd, *J* = 15.2, 5.9 Hz, 1H, H5), 2.53 (dd, *J* = 15.2, 8.6 Hz, 1H, H5), 2.48 (s, 1H, H4), 2.24 (s, 3H, H8), 2.01 – 1.92 (m, 1H, H2), 1.72 (dd, *J* = 9.4, 1.1 Hz, 3H, H3), 1.70 (dd, *J* = 9.4, 1.1 Hz, 3H, H3).

Note: the O*H* resonance was not observed.

^{13}C NMR (126 MHz, $CDCl_3$) δ 152.8, 150.6, 106.8, 106.1, 64.4, 49.6 (3C), 47.0, 41.6, 27.6, 27.4, 13.7.

HRMS (ESI^+) Found $[M+H]^+$ = 207.1382; $C_{13}H_{19}O_2$ requires 207.1380.

IR (film) ν_{max}/cm^{-1} 3385, 2963, 2908, 2870, 1709, 1570, 1433, 1369, 1280, 1198, 1142, 1065, 1020.

$[\alpha]_D^{25}$ – 15.4 (*c* = 0.3, $CHCl_3$)

HPLC 95% *ee* (CHIRALPAK IB, 2% IPA/hexane, 0.8 mL/min, t_R major – 9.3 min, minor – 10.1 min).

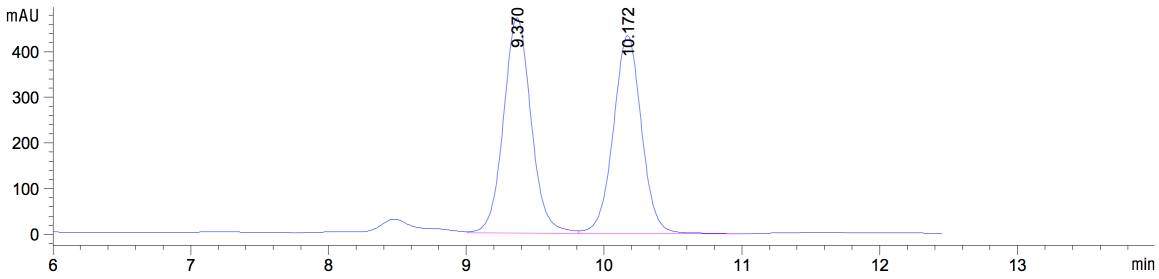

Peak #	RetTime [min]	Type	Width [min]	Area [mAU*s]	Height [mAU]	Area %
1	9.370	VV	0.2145	6572.25195	471.81628	51.6279
2	10.172	VB	0.2178	6157.79199	433.20355	48.3721

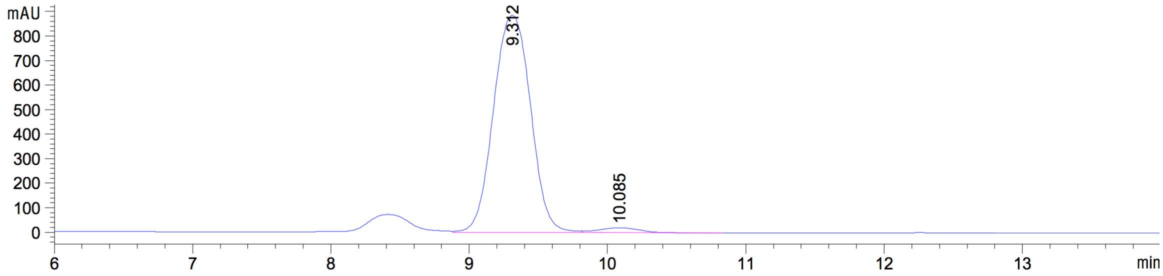

Peak #	RetTime [min]	Type	Width [min]	Area [mAU*s]	Height [mAU]	Area %
1	9.312	VV	0.3012	1.64879e4	885.58411	97.5369
2	10.085	VB	0.3247	416.37497	20.01858	2.4631

(*R*)-2-(Bicyclo[1.1.1]pentan-1-yl)-3-(thiophen-2-yl)propan-1-ol, 28

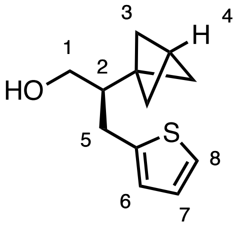

3-(Thiophen-2-yl)propanal **S85** (42 mg, 0.30 mmol, 1.5 equiv.), $Ir[(ppy)_2(dtbbpy)]PF_6$ (3.7 mg, 4.0 μmol, 0.02 equiv.), 2,4,6-tri-*tert*-butylbenzene-

thiol **10** (5.6 mg, 0.020 mmol, 0.1 equiv.), (*S*)-α,α-bis[3,5-bis(trifluoromethyl)phenyl]-2-pyrrolidinemethanol trimethylsilyl ether **9** (30 mg, 0.050 mmol, 0.25 equiv.), water (7.2 µL, 0.40 mmol, 2.0 equiv.) and TCP **1** (1.02 M in Et_2O, 0.20 mL, 0.20 mmol, 1.0 equiv.) in Et_2O (0.3 mL) and DME (0.5 mL), and sodium borohydride (38 mg, 1.0 mmol, 5.0 equiv.) in MeOH (1 mL) and CH_2Cl_2 (1 mL) were submitted to General Procedure A. Purification by column chromatography (SiO_2, pentane/Et_2O, 4:1) afforded the title compound as a pale yellow oil (10 mg, 0.050 mmol, 25%).

R*f* 0.14 (pentane/Et_2O, 4:1), [UV, vanillin]

^{1}H NMR (500 MHz, $CDCl_3$) δ 7.12 (dd, *J* = 5.2, 1.2 Hz, 1H, H8), 6.91 (dd, *J* = 5.2, 3.4 Hz, 1H, H7), 6.82 – 6.79 (m, 1H, H6), 3.59 (d, *J* = 5.2 Hz, 2H, H1), 2.89 – 2.77 (m, 2H, H5), 2.49 (s, 1H, H4), 1.96 – 1.90 (m, 1H, H2), 1.75 (dd, *J* = 9.6, 1.7 Hz, 3H, H3), 1.71 (dd, *J* = 9.6, 1.7 Hz, 3H, H3).

Note: the O*H* resonance was not observed.

^{13}C NMR (126 MHz, $CDCl_3$) δ 143.8, 126.9, 125.2, 123.5, 63.9, 49.8 (3C), 47.1, 44.5, 29.1, 27.7.

HRMS (ESI$^+$) Found $[M+H]^+$ = 209.0997; $C_{12}H_{17}OS$ requires 209.0995.

IR (film) ν_{max}/cm^{-1} 3381, 2962, 2907, 2869, 1586, 1438, 1409, 1373, 1279, 1198, 1176, 1140.

$[\alpha]_D^{25}$ – 92.8 (*c* = 0.1, $CHCl_3$)

HPLC 96% *ee* (Lux i-Amylose-1, 3% IPA/hexane, 1.0 mL/min, t_R major – 11.9 min, minor – 11.3 min).

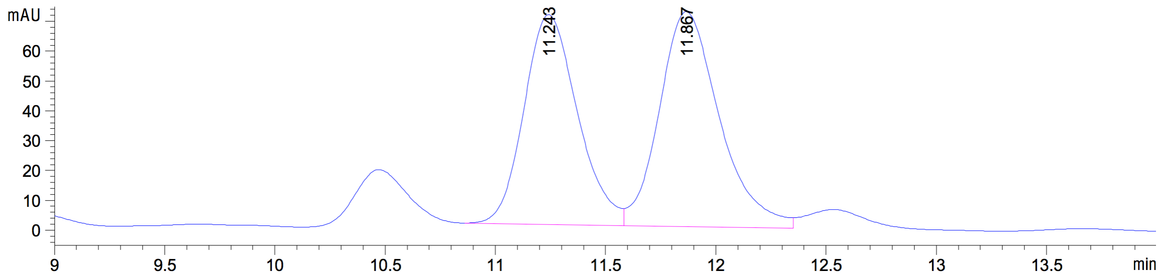

Peak #	RetTime [min]	Type	Width [min]	Area [mAU*s]	Height [mAU]	Area %
1	11.243	BV	0.2589	1187.49854	70.34469	46.4800
2	11.867	VV	0.2895	1367.36218	72.03519	53.5200

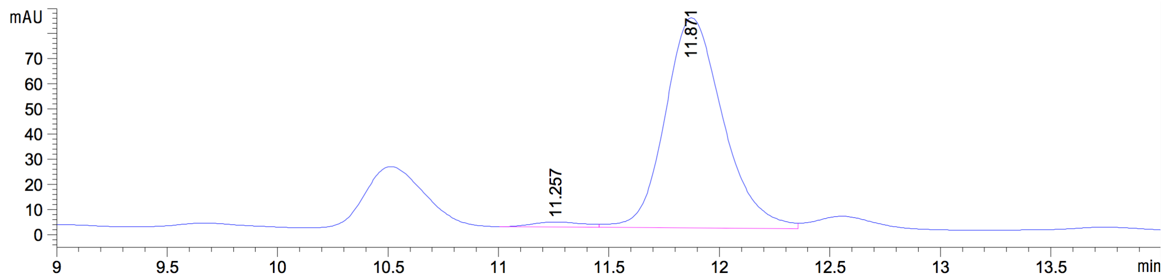

Peak #	RetTime [min]	Type	Width [min]	Area [mAU*s]	Height [mAU]	Area %
1	11.257	BV	0.2452	33.58194	2.04859	2.1539
2	11.871	VV	0.2785	1525.51978	83.74531	97.8461

(*R*)-2-(Bicyclo[1.1.1]pentan-1-yl)-3-(pyridin-3-yl)propan-1-ol, 29

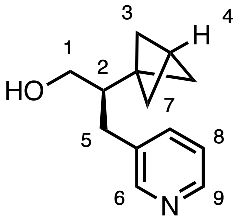

3-(Pyridin-3-yl)propanal **S86** (41 mg, 0.30 mmol, 1.5 equiv.), Ir[(ppy)$_2$(dtbbpy)]PF$_6$ (3.7 mg, 4.0 µmol, 0.02 equiv.), 2,4,6-tri-*tert*-butylbenzenethiol **10** (5.6 mg, 0.020 mmol, 0.1 equiv.), (*S*)-α,α-bis[3,5-bis(trifluoromethyl)phenyl]-2-pyrrolidinemethanol trimethylsilyl ether **9** (30 mg, 0.050 mmol, 0.25 equiv.), water (7.2 µL, 0.40 mmol, 2.0 equiv.) and TCP **1** (1.02 M in Et$_2$O, 0.20 mL, 0.20 mmol, 1.0 equiv.) in Et$_2$O (0.3 mL) and DME (0.5 mL), and sodium borohydride (38 mg, 1.0 mmol, 5.0 equiv.) in MeOH (1 mL) and CH$_2$Cl$_2$ (1 mL) were submitted to General Procedure A. Purification by column chromatography (SiO$_2$, EtOAc) afforded the title compound as a pale yellow oil (12 mg, 0.058 mmol, 29%).

R*$_f$* 0.19 (EtOAc), [UV, vanillin]

^{1}H NMR (500 MHz, CDCl$_3$) δ 8.51 – 8.43 (m, 2H, H6 and H9), 7.61 – 7.56 (m, 1H, H7), 7.25 – 7.23 (m, 1H, H8), 3.56 – 3.52 (m, 2H, H1), 2.68 – 2.59 (m, 2H, H5), 2.50 (s, 1H, H4), 1.94 – 1.88 (m, 1H, H2), 1.73 (dd, *J* = 9.6, 1.8 Hz, 3H, H3), 1.69 (dd, *J* = 9.6, 1.8 Hz, 3H, H3).

Note: the O*H* resonance was not observed.

^{13}C NMR (126 MHz, CDCl$_3$) δ 149.9, 146.8, 137.3, 136.9, 123.6, 63.4, 49.9 (3C), 47.1, 43.6, 32.0, 27.8.

HRMS (ESI$^+$) Found [M+H]$^+$ = 204.1385; C$_{13}$H$_{18}$ON requires 204.1383.

IR (film) ν_{max}/cm^{-1} 3219, 2962, 2917, 2867, 1479, 1279, 1140.

$[\alpha]_D^{25}$ – 31.3 (c = 0.2, $CHCl_3$)

HPLC 90% *ee* (CHIRALPAK IB, 20% IPA/hexane, 1.3 mL/min, t_R major – 5.1 min, minor – 5.9 min).

Note: BCP product **29** is slightly unstable to column chromatography and HPLC (under the specified conditions).

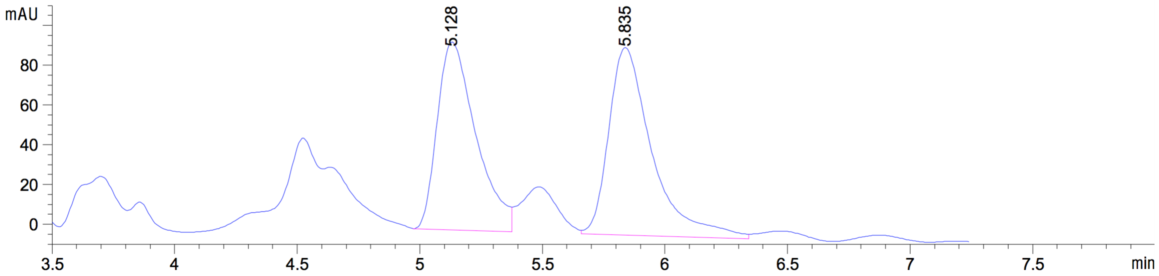

Peak #	RetTime [min]	Type	Width [min]	Area [mAU*s]	Height [mAU]	Area %
1	5.128	BV	0.1643	1039.00916	94.46390	47.2769
2	5.835	VV	0.1832	1158.70203	94.39435	52.7231

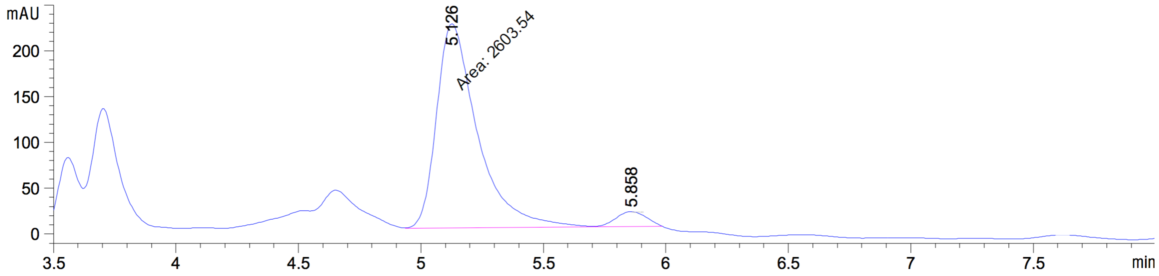

Peak #	RetTime [min]	Type	Width [min]	Area [mAU*s]	Height [mAU]	Area %
1	5.126	MM	0.1945	2603.54468	223.09227	94.8317
2	5.858	MM	0.1436	141.89253	16.47056	5.1683

N-(Phenylthio)phthalimide, S51

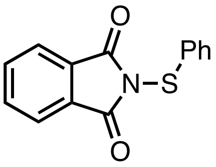

According to the procedure of Zhao et al.[14] To a solution of thiophenol (1.0 mL, 10 mmol, 1.0 equiv.) and Et_3N (0.14 mL, 1.0 mmol, 0.1 equiv.) in CH_2Cl_2 (10 mL) at 0 °C under an inert atmosphere was added a solution of sulfuryl chloride (0.81 mL, 10 mmol, 1.0 equiv.) in CH_2Cl_2 (2 mL). The reaction mixture was left to stir for 15 min at 0 °C and then allowed to warm to room temperature and stirred for a further 30 min. The reaction mixture was then

cooled to 0 °C and the resulting solution was added dropwise to a solution of phthalimide (1.5 g, 10 mmol, 1.0 equiv.) and Et_3N (1.5 mL, 11 mmol, 1.1 equiv.) in CH_2Cl_2 (10 mL) at 0 °C. The resulting mixture was allowed to warm to room temperature and left to stir for 3 h. Water (20 mL) was then added. The phases were separated and the aqueous phase was extracted with CH_2Cl_2 (3 × 10 mL). The combined organic extracts were dried (Na_2SO_4) and concentrated *in vacuo*. Purification by recrystallization (CH_2Cl_2/hexanes) afforded the title compound as white crystals (1.5 g, 5.8 mmol, 58%).

R*f* 0.36 (pentane/Et_2O, 3:2), [UV, potassium permanganate]
^{1}H NMR (500 MHz, $CDCl_3$) δ 7.96 – 7.90 (m, 2H, Ar*H*), 7.81 – 7.75 (m, 2H, Ar*H*), 7.63 – 7.57 (m, 2H, Ar*H*), 7.36 – 7.30 (m, 3H, Ar*H*).
^{13}C NMR (126 MHz, $CDCl_3$) δ 167.9 (2C), 135.2, 134.8 (2C), 132.2 (2C), 131.1 (2C), 129.5 (3C), 124.2 (2C).
Spectroscopic data in agreement with that reported previously.[15]

(*R*)-3-Phenyl-2-(3-(phenylthio)bicyclo[1.1.1]pentan-1-yl)propan-1-ol, 30

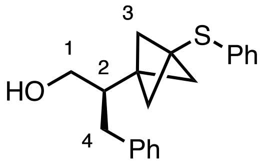

Hydrocinnamaldehyde (40 µL, 0.30 mmol, 1.5 equiv.), Ir[(ppy)$_2$(dtbbpy)]PF_6 (3.7 mg, 4.0 µmol, 0.02 equiv.), *N*-(phenylthio)phthalimide **S51** (0.20 g, 0.80 mmol, 4.0 equiv.), (*S*)-α,α-bis[3,5-bis(trifluoromethyl)phenyl]-2-pyrrolidinemethanol trimethylsilyl ether **9** (30 mg, 0.050 mmol, 0.25 equiv.), water (7.2 µL, 0.40 mmol, 2.0 equiv.) and TCP **1** (1.02 M in Et_2O, 0.20 mL, 0.20 mmol, 1.0 equiv.) in Et_2O (0.3 mL) and DME (0.5 mL), and sodium borohydride (38 mg, 1.0 mmol, 5.0 equiv.) in MeOH (1 mL) and CH_2Cl_2 (1 mL) were submitted to General Procedure A. Purification by column chromatography (SiO_2, pentane/Et_2O, 17:3) afforded the title compound as a pale yellow oil (31 mg, 0.10 mmol, 50%).

R*f* 0.45 (pentane/Et_2O, 1:1), [UV, vanillin]
^{1}H NMR (500 MHz, $CDCl_3$) δ 7.45 – 7.41 (m, 2H, Ar*H*), 7.32 – 7.23 (m, 5H, Ar*H*), 7.20 – 7.13 (m, 3H, Ar*H*), 3.52 (dd, *J* = 10.8, 4.8 Hz, 1H, H1), 3.48 (dd, *J* = 10.8, 5.6 Hz, 1H, H1), 2.60 (dd, *J* = 13.9, 6.1 Hz, 1H, H4), 2.54 (dd, *J* = 13.9, 9.1 Hz, 1H, H4), 2.00 (dq,

J = 9.1, 5.6 Hz, 1H, H2), 1.86 (dd, J = 9.5, 1.6 Hz, 3H, H3), 1.82 (dd, J = 9.5, 1.6 Hz, 3H, H3).

Note: the O*H* resonance was not observed.

^{13}C NMR (126 MHz, $CDCl_3$) δ 140.4, 134.1, 133.8 (2C), 129.0 (2C), 128.9 (2C), 128.6 (2C), 127.7, 126.2, 63.7, 53.6 (3C), 51.9, 43.2, 41.9, 35.2.

HRMS (ESI^+) Found $[M+H]^+$ = 311.1465; $C_{20}H_{23}OS$ requires 311.1464.

IR (film) ν_{max}/cm^{-1} 3381, 3027, 2966, 2911, 2872, 1583, 1496, 1474, 1454, 1439, 1280, 1192.

$[\boldsymbol{\alpha}]_{\mathbf{D}}^{\mathbf{25}}$ – 17.0 (c = 0.3, $CHCl_3$)

HPLC 88% *ee* (Lux i-Amylose-1, 5% IPA/hexane, 1.0 mL/min, t_R major – 13.6 min, minor – 14.9 min).

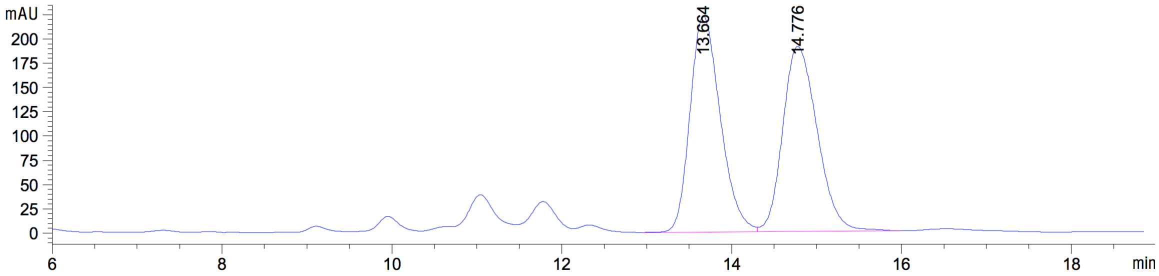

Peak #	RetTime [min]	Type	Width [min]	Area [mAU*s]	Height [mAU]	Area %
1	13.664	BV	0.3845	5505.85449	222.67969	50.5792
2	14.776	VB	0.4435	5379.76074	190.50244	49.4208

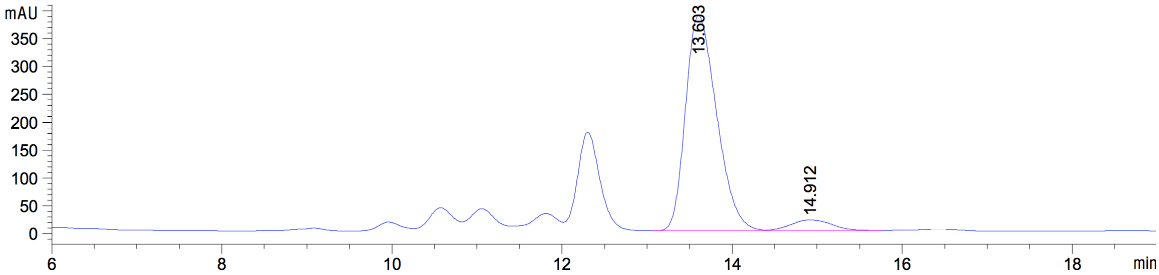

Peak #	RetTime [min]	Type	Width [min]	Area [mAU*s]	Height [mAU]	Area %
1	13.603	BV	0.3972	9848.30762	386.64883	93.9477
2	14.912	VB	0.5252	634.44794	19.11741	6.0523

(*R*)-2-(Bicyclo[1.1.1]pentan-1-yl)-3-phenylpropanoic acid, 32

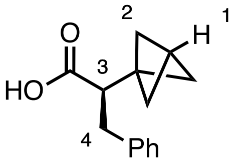

To a solution of (*R*)-2-(bicyclo[1.1.1]pentan-1-yl)-3-phenylpropanal **31** (obtained directly from the photoredox-catalyzed asymmetric reaction) (40 mg, 0.20 mmol, 1.0 equiv.) in CH_2Cl_2 (0.7 mL) and water (0.3 mL) at room temperature was added TEMPO (6.3 mg, 0.040 mmol, 0.2 equiv.) and BAIB (0.13 g, 0.40 mmol, 2.0 equiv.). The reaction mixture was left to stir for 24 h. Upon completion, water (0.6 mL), $Na_2S_2O_3$ (aq., sat., 0.6 mL) and $NaHCO_3$ (aq., sat., 0.6 mL) were added. The phases were separated and the aqueous phase was extracted with CH_2Cl_2 (3 × 1 mL). The combined organic extracts were dried (Na_2SO_4) and concentrated *in vacuo*. Purification by column chromatography (SiO_2, pentane/Et_2O, 4:1) afforded the title compound as a pale yellow (42 mg, 0.19 mmol, 97%).

R*f* 0.31 (pentane/Et_2O, 7:3), [UV, vanillin]

^{1}H NMR (500 MHz, $CDCl_3$) δ 10.0 (br s, 1H, CO_2H), 7.30 – 7.16 (m, 5H, Ar*H*), 2.94 – 2.82 (m, 2H, H4 and H3), 2.74 (dd, *J* = 13.2, 4.0 Hz, 1H, H4), 2.52 (s, 1H, H1), 1.77 (s, 6H, H2).

^{13}C NMR (126 MHz, $CDCl_3$) δ 177.5, 139.5, 128.8 (2C), 128.5 (2C), 126.4, 49.8 (3C), 49.6, 44.9, 34.9, 26.9.

HRMS (ESI$^+$) Found $[M+Na]^+$ = 239.1043; $C_{14}H_{16}O_2Na$ requires 239.1043.

IR (film) ν_{max}/cm^{-1} 3029, 2968, 2909, 2873, 1705, 1496, 1455.

$[\alpha]_D^{25}$ – 78.0 (*c* = 0.1, $CHCl_3$)

HPLC 95% *ee* (CHIRALPAK IC, 2% IPA/hexane, 0.7 mL/min, t_R major – 8.2 min, minor – 8.8 min).

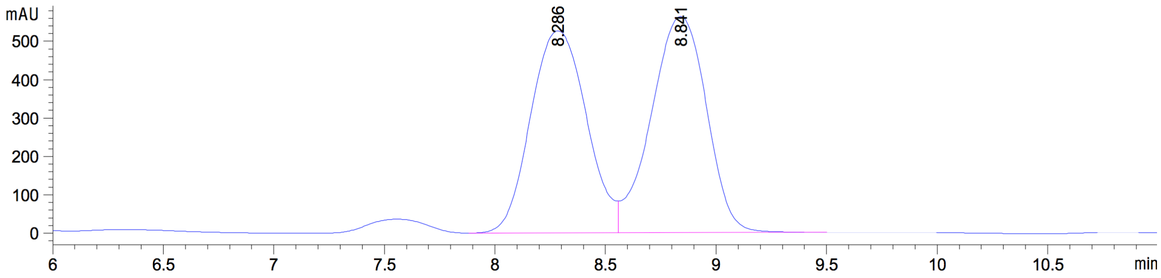

Peak #	RetTime [min]	Type	Width [min]	Area [mAU*s]	Height [mAU]	Area %
1	8.286	BV	0.2810	9254.02637	526.57086	48.5519
2	8.841	VB	0.2770	9806.04980	563.61884	51.4481

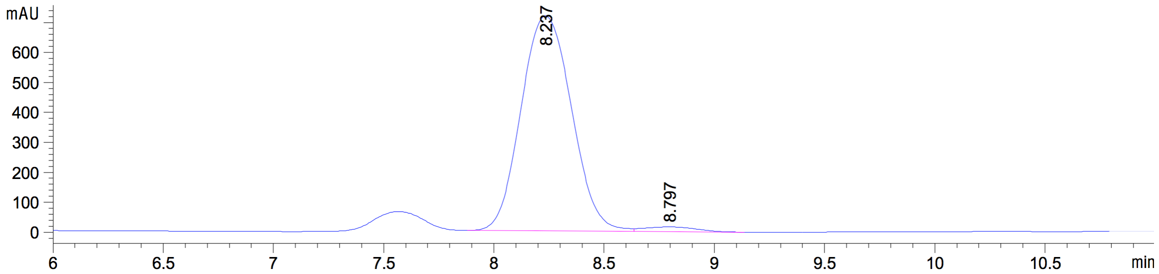

Peak #	RetTime [min]	Type	Width [min]	Area [mAU*s]	Height [mAU]	Area %
1	8.237	BV	0.2559	1.14472e4	718.57477	97.7289
2	8.797	VB	0.2592	266.02200	16.06785	2.2711

(*R*)-*N*-Benzyl-2-(bicyclo[1.1.1]pentan-1-yl)-3-phenylpropan-1-amine, 33

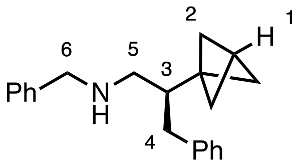

To a solution of (*R*)-2-(bicyclo[1.1.1]pentan-1-yl)-3-phenylpropanal **31** (obtained directly from the photoredox-catalyzed asymmetric reaction) (40 mg, 0.20 mmol, 1.0 equiv.) in DCE (3 mL) at room temperature under an inert atmosphere was added benzylamine (28 µL, 0.26 mmol, 1.3 equiv.), acetic acid (15 µL, 0.26 mmol, 1.3 equiv.) and Et_3N (39 µL, 0.28 mmol, 1.4 equiv.). The reaction mixture was stirred vigorously for 15 min. Sodium triacetoxyborohydride (0.11 g, 0.50 mmol, 2.5 equiv.) was then added and the reaction mixture was stirred vigorously for 19.5 h. Upon completion, $NaHCO_3$ (aq., sat., 1.5 mL) and water (1 mL) were added. The phases were separated and the aqueous phase was extracted with CH_2Cl_2 (3 × 2 mL). The combined organic extracts were dried (Na_2SO_4) and concentrated *in vacuo*. Purification by column chromatography (SiO_2, pentane/Et_2O, 7:3) afforded the title compound as a pale yellow oil (54 mg, 0.19 mmol, 93%).

R$_f$ 0.64 (Et_2O), [UV, vanillin]

^{1}H NMR (500 MHz, $CDCl_3$) δ 7.32 – 7.14 (m, 10H, Ar*H*), 3.67 (d, *J* = 3.4 Hz, 2H, H6), 2.65 (dd, *J* = 13.9, 6.1 Hz, 1H, H4), 2.59 (dd, *J* = 11.8, 6.1 Hz, 1H, H5), 2.54 – 2.46 (m, 2H, H4 and H5), 2.45 (s, 1H, H1), 2.00 (dq, *J* = 8.9, 6.1 Hz, 1H, H3), 1.66 (dd, *J* = 9.5, 1.6 Hz, 3H, H2), 1.65 (dd, *J* = 9.5, 1.6 Hz, 3H, H2).

Note: the N*H* resonance was not observed.

^{13}C NMR (126 MHz, $CDCl_3$) δ 141.4, 140.2, 129.1 (2C), 128.5 (2C), 128.4 (2C), 128.3 (2C), 127.0, 125.9, 54.1, 51.1, 49.6 (3C), 48.4, 41.3, 37.0, 27.4.

IR (film) ν_{max}/cm^{-1} 2961, 2906, 2867, 1603, 1495, 1453, 1265, 1196.

$[\alpha]_D^{25}$ – 85.9 (*c* = 0.1, $CHCl_3$)

HPLC 92% *ee* (CHIRALPAK IB, 5% IPA/hexane, 1.0 mL/min, t_R major – 3.5 min, minor – 3.3 min).

Note: BCP product **33** is slightly unstable to HPLC (under the specified conditions).

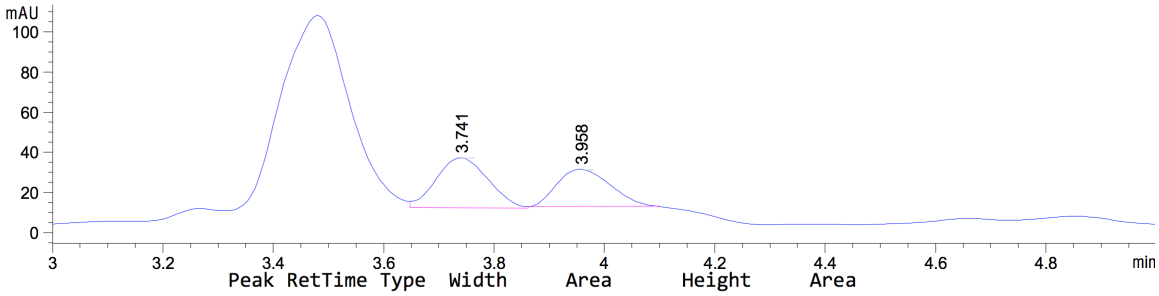

Peak #	RetTime [min]	Type	Width [min]	Area [mAU*s]	Height [mAU]	Area %
1	3.741	MM	0.1077	160.75912	24.87170	56.4887
2	3.958	MM	0.1111	123.82742	18.57020	43.5113

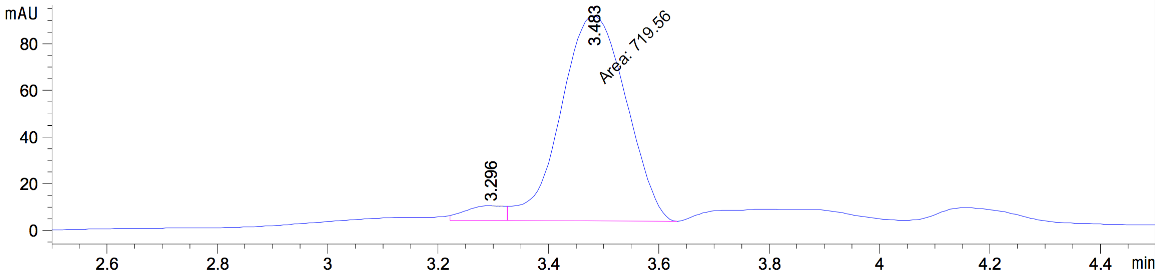

Peak #	RetTime [min]	Type	Width [min]	Area [mAU*s]	Height [mAU]	Area %
1	3.296	MM	0.0839	31.35016	6.22538	4.1750
2	3.483	MM	0.1363	719.56049	87.97800	95.8250

(1*S*,2*R*)-2-(Bicyclo[1.1.1]pentan-1-yl)-1-(4-methoxyphenyl)-3-phenylpropan-1-ol, 34

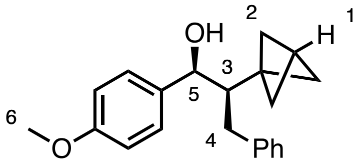

To a solution of (*R*)-2-(bicyclo[1.1.1]pentan-1-yl)-3-phenylpropanal **31** (obtained directly from the photoredox-catalyzed asymmetric reaction) (40 mg, 0.20 mmol, 1.0 equiv.) in THF (1 mL) at –78 °C under an inert atmosphere was added 4-methoxyphenylmagnesium bromide (0.75 M in THF, 0.85 mL, 0.64 mmol, 3.2 equiv.) dropwise. The reaction mixture was allowed to warm to room temperature slowly and left to stir for 14 h. Upon completion, HCl (aq., 1 M, 1 mL) was added. The phases were separated and the aqueous phase was extracted with Et_2O (3 × 1 mL). The combined organic extracts were washed with water (2 mL) and brine (2 mL), dried (Na_2SO_4) and concentrated *in vacuo*. Purification by column chromatography (SiO_2, pentane/Et_2O, 9:1) afforded the title compound as a pale yellow (59 mg, 0.19 mmol, 95%, 14:1 *dr*).

$\mathbf{R}_f$ 0.12 (pentane/Et_2O, 9:1), [UV, vanillin]

^{1}H NMR (500 MHz, $CDCl_3$) δ 7.29 (d, *J* = 8.7 Hz, 2H, Ar*H*), 7.23 – 7.06 (m, 5H, Ar*H*), 6.88 (d, *J* = 8.7 Hz, 2H, Ar*H*), 4.80 (dd, *J* = 5.2, 2.2 Hz, 1H, H5), 3.81 (s, 3H, H6), 2.86 (dd, *J* = 14.3, 3.7 Hz, 1H, H4), 2.60 (dd, *J* = 14.3, 9.3 Hz, 1H, H4), 2.33 (s, 1H, H1), 2.22 (dt, *J* = 8.9, 4.2 Hz, 1H, H3), 1.56 (dd, *J* = 9.7, 1.8 Hz, 3H, H2), 1.52 (dd, *J* = 9.7, 1.8 Hz, 3H, H2).

Note: the O*H* resonance was not observed.

^{13}C NMR (126 MHz, $CDCl_3$) δ 158.9, 142.1, 136.1, 129.1 (2C), 128.2 (2C), 127.5 (2C), 125.6, 113.8 (2C), 75.5, 55.4, 50.5 (3C), 48.4, 47.8, 32.6, 28.1.

IR (film) ν_{max}/cm^{-1} 3482, 3423, 2961, 2907, 2870, 1612, 1511, 1454, 1303, 1248, 1176.

$[\boldsymbol{\alpha}]_\mathbf{D}^{\mathbf{25}}$ – 39.3 (*c* = 0.1, $CHCl_3$)

HPLC 94% *ee* (Lux i-Amylose-1, 5% IPA/hexane, 1.0 mL/min, t_R major – 14.4 min, minor – 15.7 min).

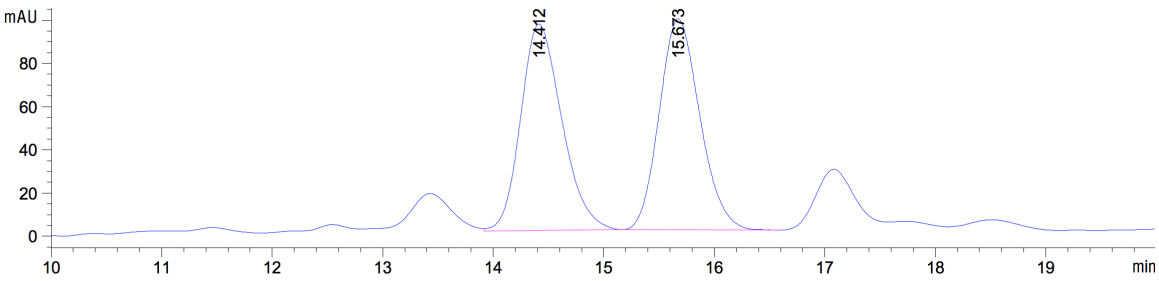

Peak #	RetTime [min]	Type	Width [min]	Area [mAU*s]	Height [mAU]	Area %
1	14.412	VB	0.3955	2467.87769	95.50046	49.7693
2	15.673	BB	0.3938	2490.75415	97.56338	50.2307

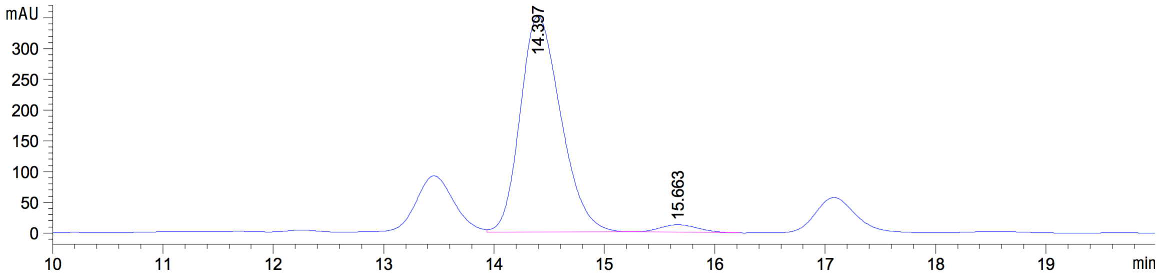

Peak #	RetTime [min]	Type	Width [min]	Area [mAU*s]	Height [mAU]	Area %
1	14.397	VB	0.3930	8950.46387	351.54779	96.7758
2	15.663	BB	0.3699	298.19360	12.42777	3.2242

(*R*)-1-(1-Phenylbut-3-yn-2-yl)bicyclo[1.1.1]pentane, 35

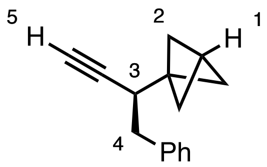

To a solution of (*R*)-2-(bicyclo[1.1.1]pentan-1-yl)-3-phenylpropanal **31** (obtained directly from the photoredox-catalyzed asymmetric reaction) (40 mg, 0.20 mmol, 1.0 equiv.) in MeOH (2 mL) at room temperature under an inert atmosphere was added dimethyl (1-diazo-2-oxopropyl)phosphonate (96 µL, 0.40 mmol, 2.0 equiv.) and potassium carbonate (55 mg, 0.40 mmol, 2.0 equiv.). The reaction mixture was left to stir for 19 h. Upon completion, water (4 mL) was added. The phases were separated and the aqueous phase was extracted with CH_2Cl_2 (3 × 2 mL). The combined organic extracts were washed with brine (5 mL), dried (Na_2SO_4) and concentrated *in vacuo*. Purification by column chromatography (SiO_2, pentane) afforded the title compound as a clear oil (38 mg, 0.19 mmol, 97%).

R*f* 0.10 (pentane), [UV, vanillin]

^{1}H NMR (500 MHz, $CDCl_3$) δ 7.32 – 7.18 (m, 5H, Ar*H*), 2.79 (dd, *J* = 13.1, 4.8 Hz, 1H, H4), 2.71 (ddd, *J* = 10.2, 4.8, 2.4 Hz, 1H, H3), 2.60 (dd, *J* = 13.1, 10.2 Hz, 1H, H4), 2.53 (s, 1H, H1), 2.04 (d, *J* = 2.4 Hz, 1H, H5), 1.74 (s, 6H, H2).

^{13}C NMR (126 MHz, $CDCl_3$) δ 139.8, 129.1 (2C), 128.3 (2C), 126.4, 84.6, 70.7, 48.9 (3C), 46.1, 38.2, 36.3, 27.0.

IR (film) ν_{max}/cm^{-1} 3307, 2967, 2908, 2872, 1496, 1454, 1280, 1198.

$[\boldsymbol{\alpha}]_{\mathbf{D}}^{\mathbf{25}}$ – 82.3 (*c* = 0.1, $CHCl_3$)

HPLC 96% *ee* (Lux i-Amylose-1, hexane, 0.8 mL/min, t_R major – 6.7 min, minor – 6.2 min).

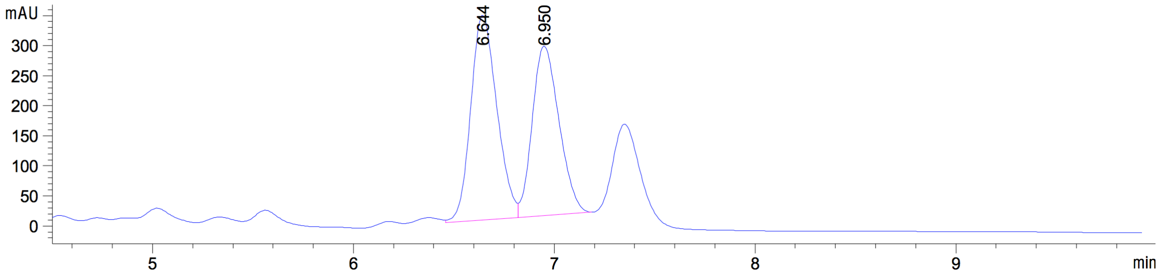

Peak #	RetTime [min]	Type	Width [min]	Area [mAU*s]	Height [mAU]	Area %
1	6.644	VV	0.1416	3082.24292	340.47083	54.0212
2	6.950	VB	0.1445	2623.37793	282.16434	45.9788

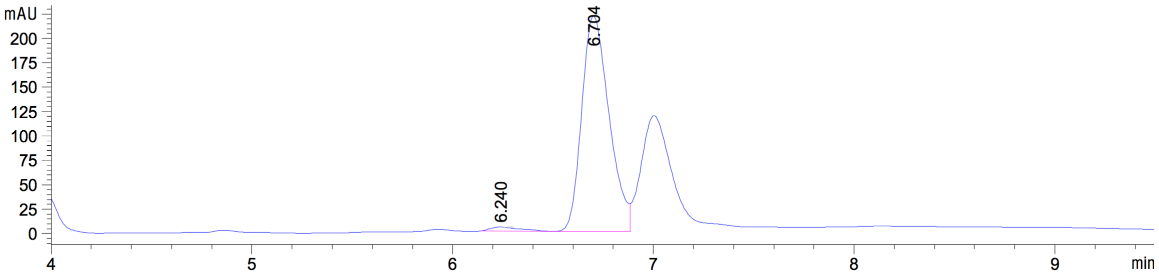

Peak #	RetTime [min]	Type	Width [min]	Area [mAU*s]	Height [mAU]	Area %
1	6.240	MM	0.1769	45.59887	4.29621	2.1412
2	6.704	BV	0.1460	2083.96094	220.96255	97.8588

3.2.3 HAT catalysts

HAT catalysts **S2**, **S8**, **S11**, **S12**, **S19**, **S20**, **S21**, **S22**, **S23**, **S24**, **S25**, **S26** and **S27** are commercially available and used directly as supplied.

2,4,6-Tri-*tert*-butylbenzenethiol, 10

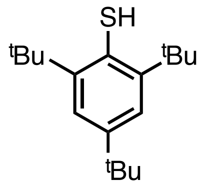

Magnesium turnings (73 mg, 3.0 mmol, 2.0 equiv.), 2-bromo-1,3,5-tri-*tert*-butylbenzene (0.49 g, 1.5 mmol, 1.0 equiv.), sulfur (51 mg, 1.6 mmol, 1.05 equiv.) and lithium aluminium hydride (1 M in THF, 0.75 mL, 0.75 mmol, 0.5 equiv.) in THF (3 mL) were submitted to General Procedure C. Purification by recrystallization (pentane at –20 °C) afforded the title compound as an off-white solid (0.26 g, 0.92 mmol, 61%).

R*f* 0.74 (pentane/CH_2Cl_2, 4:1), [UV, vanillin]
^{1}H NMR (400 MHz, C_6D_6) δ 7.56 (s, 2H, Ar*H*), 3.43 (s, 1H, S*H*), 1.61 (s, 18H, *o*-C(C*H*$_3$)$_3$), 1.30 (s, 9H, *p*-C(C*H*$_3$)$_3$).
^{13}C NMR (101 MHz, C_6D_6) δ 151.2 (2C), 150.4, 122.8 (2C), 119.7, 37.7, 32.8 (2C), 31.8 (3C), 31.6 (6C).
Spectroscopic data in agreement with that reported previously.[8]

Reaction of 2,4,6-tri-*tert*-butylbenzenethiol 10 with [1.1.1]propellane 1

Bicyclo[1.1.1]pentan-1-yl(2,4,6-tri-*tert*-butylphenyl)sulfane, S28

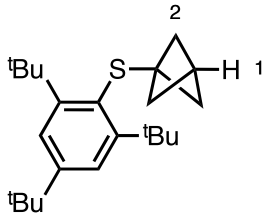

A capped vial was charged with 2,4,6-tri-*tert*-butylbenzenethiol **10** (17 mg, 0.060 mmol, 1.0 equiv.) and TCP **1** (0.63 M in Et_2O, 9.5 µL, 0.060 mmol, 1.0 equiv.) and the mixture was stirred for 24 h at room temperature either in the dark with aluminium foil protection or with blue LED irradiation. The reaction mixture was then concentrated *in vacuo*. Purification by

column chromatography (SiO_2, pentane) afforded the title compound as a colorless oil (dark: 9.9 mg, 0.029 mmol, 48%; blue LED irradiation: 11 mg, 0.031 mmol, 51%).

R*f* 0.67 (pentane), [UV, vanillin]

^{1}H NMR (500 MHz, $CDCl_3$) δ 7.29 (s, 2H, Ar*H*), 2.45 (s, 1H, H1), 1.68 (s, 6H, H2), 1.53 (s, 18H, *o*-C(C*H*$_3$)$_3$), 1.30 (s, 9H, *p*-C(C*H*$_3$)$_3$).

^{13}C NMR (126 MHz, $CDCl_3$) δ 155.3 (2C), 149.1, 130.7, 121.8 (2C), 54.1 (3C), 49.7, 38.4 (2C), 35.0, 33.6 (6C), 31.5 (3C), 27.2.

HRMS (ESI$^+$) Found $[M+H]^+$ = 345.2610; $C_{23}H_{37}S$ requires 345.2611.

IR (film) ν_{max}/cm^{-1} 2961, 2910, 2875, 1587, 1481, 1390, 1359, 1240, 1206.

2,4,6-Tri-*tert*-butylbenzeneselenol, S1

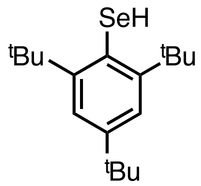

Magnesium turnings (49 mg, 2.0 mmol, 2.0 equiv.), 2-bromo-1,3,5-tri-*tert*-butylbenzene (0.33 g, 1.0 mmol, 1.0 equiv.), selenium (83 mg, 1.1 mmol, 1.05 equiv.) and lithium aluminium hydride (1 M in THF, 0.50 mL, 0.50 mmol, 0.5 equiv.) in THF (2 mL) were submitted to General Procedure C. The resulting residue was filtered through a short pad of silica (eluting with pentane) and the filtrate was concentrated *in vacuo*. Purification by recrystallization (hexane) afforded the title compound as a yellow solid (0.11 g, 0.33 mmol, 33%). The recrystallized sample also contained 1,3,5-tri-*tert*-butylbenzene as an impurity that could not be separated from the title compound (13%).

R*f* 0.64 (pentane), [UV, vanillin]

m.p. 200 – 201 °C

^{1}H NMR (500 MHz, $CDCl_3$) δ 7.44 (s, 2H, Ar*H*), 1.88 (s, 1H, Se*H*), 1.63 (s, 18H, *o*-C(C*H*$_3$)$_3$), 1.31 (s, 9H, *p*-C(C*H*$_3$)$_3$).

^{13}C NMR (126 MHz, $CDCl_3$) δ 152.1 (2C), 148.7, 123.0 (2C), 120.9, 38.5 (2C), 35.1, 32.1 (6C), 31.5 (3C).

HRMS (EI) Found $[M]^+$ = 326.1507; $C_{18}H_{30}Se$ requires 326.1507.

IR (film) ν_{max}/cm^{-1} 3026, 2953, 1361, 1214, 1022.

2,4,6-Tricyclohexylbenzenethiol, S3

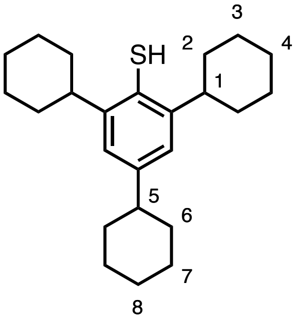

According to the procedure of Buchwald et al.[16] To a solution of benzene (0.45 mL, 5.0 mmol, 1.0 equiv.) and aluminium trichloride (2.1 g, 16 mmol, 3.2 equiv.) in CH_2Cl_2 (3.4 mL) at 0 °C under an inert atmosphere was added cyclohexyl bromide (2.0 mL, 16 mmol, 3.2 equiv.) dropwise. The reaction mixture was allowed to warm to room temperature and stirred for 4 h. Upon completion, the reaction mixture was carefully quenched with ice water (3 mL) and diluted with Et_2O (11 mL). The phases were separated and the organic phase was washed with water (3 mL) and brine (3 mL), dried (Na_2SO_4) and concentrated *in vacuo*. The resulting residue was filtered through a short pad of silica (eluting with pentane) and the filtrate was concentrated *in vacuo* to afford 1,3,5-tricyclohexylbenzene **S52** as an orange oil (1.3 g, 4.0 mmol, 79%).

According to the procedure of Buchwald et al.[16] To a solution of 1,3,5-tricyclohexylbenzene **S52** (1.3 g, 4.0 mmol, 1.0 equiv.) in $CHCl_3$ (3 mL) at 0 °C under an inert atmosphere was added bromine (0.20 mL, 4.0 mmol, 1.0 equiv.) dropwise. The reaction mixture was allowed to warm to room temperature and left to stir for 20 h. The reaction mixture was then diluted with CH_2Cl_2 (7 mL) and the resulting solution was washed with water (10 mL). The aqueous phase was extracted with CH_2Cl_2 (2 × 5 mL). The combined organic extracts were washed with NaOH (aq., 10%, 7 mL) and brine (10 mL), dried (Na_2SO_4) and concentrated *in vacuo*. The resulting residue was filtered through a short pad of silica (eluting with pentane) and the filtrate was concentrated *in vacuo*. The resulting residue was triturated with EtOAc to afford (2-bromobenzene-1,3,5-triyl)tricyclohexane **S53** as a white solid (0.48 g, 1.2 mmol, 30%).

Magnesium turnings (49 mg, 2.0 mmol, 2.0 equiv.), (2-bromobenzene-1,3,5-triyl)tricyclohexane **S53** (0.40 g, 1.0 mmol, 1.0 equiv.), sulfur (34 mg, 1.1 mmol, 1.05 equiv.) and lithium aluminium hydride (1 M in THF, 0.50 mL, 0.50 mmol, 0.5 equiv.) in THF (2 mL) were submitted to General Procedure C. Purification by column

chromatography (SiO_2, pentane) afforded the title compound as a white solid (0.23 g, 0.65 mmol, 65%).

R$_f$ 0.48 (pentane), [UV, vanillin]

m.p. 154 – 155 °C

^{1}H NMR (500 MHz, $CDCl_3$) δ 6.95 (s, 2H, Ar*H*), 3.10 – 3.03 (m, 2H, H1), 3.04 (s, 1H, S*H*), 2.48 – 2.40 (m, 1H, H5), 1.91 – 1.20 (m, 30H, H2, H3, H4, H6, H7 and H8).

^{13}C NMR (126 MHz, $CDCl_3$) δ 147.1 (2C), 146.2, 124.7, 122.6 (2C), 44.8, 42.8 (2C), 34.6 (2C), 33.8 (4C), 27.3 (4C), 27.1 (2C), 26.5 (2C), 26.3.

HRMS (ESI$^-$) Found $[M–H]^-$ = 355.2466; $C_{24}H_{35}S$ requires 355.2465.

IR (film) ν_{max}/cm^{-1} 2923, 2850, 1599, 1447.

2,4,6-Tricyclopentylbenzenethiol, S4

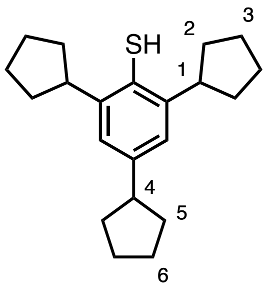

According to the procedure of Buchwald et al.[16] To a solution of benzene (0.45 mL, 5.0 mmol, 1.0 equiv.) and aluminium trichloride (2.1 g, 16 mmol, 3.2 equiv.) in CH_2Cl_2 (3.4 mL) at 0 °C under an inert atmosphere was added cyclopentyl bromide (1.7 mL, 16 mmol, 3.2 equiv.) dropwise. The reaction mixture was allowed to warm to room temperature and stirred for 4 h. Upon completion, the reaction mixture was carefully quenched with ice water (3 mL) and diluted with Et_2O (11 mL). The phases were separated and the organic phase was washed with water (3 mL) and brine (3 mL), dried (Na_2SO_4) and concentrated *in vacuo*. The resulting residue was filtered through a short pad of silica (eluting with pentane) and the filtrate was concentrated *in vacuo* to afford 1,3,5-tricyclopentyl-benzene **S54** as an orange oil (1.2 g, 4.2 mmol, 84%).

According to the procedure of Buchwald et al.[16] To a solution of 1,3,5-tricyclopentylbenzene **S54** (1.2 g, 4.2 mmol, 1.0 equiv.) in $CHCl_3$ (3 mL) at 0 °C under an inert atmosphere was added bromine (0.21 mL, 4.2 mmol, 1.0 equiv.) dropwise. The reaction mixture was allowed to warm to room temperature and left to stir for 20 h. The reaction mixture was then diluted with CH_2Cl_2 (7 mL) and the resulting solution was washed

with water (10 mL). The aqueous phase was extracted with CH_2Cl_2 (2 × 5 mL). The combined organic extracts were washed with NaOH (aq., 10%, 7 mL) and brine (10 mL), dried (Na_2SO_4) and concentrated *in vacuo*. The resulting residue was filtered through a short pad of silica (eluting with hexane) and the filtrate was concentrated *in vacuo* to afford 2-bromo-1,3,5-tricyclopentylbenzene **S55** as a colorless oil (0.76 g, 2.1 mmol, 51%).

Magnesium turnings (49 mg, 2.0 mmol, 2.0 equiv.), 2-bromo-1,3,5-tricyclopentyl-benzene **S55** (0.36 g, 1.0 mmol, 1.0 equiv.), sulfur (34 mg, 1.1 mmol, 1.05 equiv.) and lithium aluminium hydride (1 M in THF, 0.50 mL, 0.50 mmol, 0.5 equiv.) in THF (2 mL) were submitted to General Procedure C. Purification by column chromatography (SiO_2, pentane) afforded the title compound as a pale yellow oil (0.29 g, 0.93 mmol, 93%).

R*f* 0.60 (pentane), [UV, vanillin]

^{1}H NMR (400 MHz, $CDCl_3$) δ 7.02 (s, 2H, Ar*H*), 3.54 (tt, *J* = 9.2, 7.4 Hz, 2H, H1), 3.13 (s, 1H, S*H*), 2.98 – 2.87 (m, 1H, H4), 2.15 – 1.99 (m, 6H, H2 and H5), 1.86 – 1.64 (m, 12H, H3 and H6), 1.64 – 1.54 (m, 6H, H2 and H5).

^{13}C NMR (126 MHz, $CDCl_3$) δ 145.9 (2C), 144.5, 126.1, 122.6 (2C), 46.2, 44.3 (2C), 34.9 (2C), 33.8 (4C), 25.6 (2C), 25.6 (4C).

HRMS (EI) Found $[M]^+$ = 314.2068; $C_{21}H_{30}S$ requires 314.2063.

IR (film) ν_{max}/cm^{-1} 2950, 2867, 1599, 1563, 1452.

2,4,6-Triisopropylbenzenethiol, S5

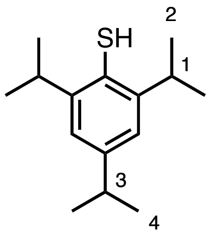

According to the procedure of Knowles et al.[17] To a solution of lithium aluminium hydride (4 M in Et_2O, 0.75 mL, 3.0 mmol, 2.0 equiv.) in Et_2O (0.23 mL) at 0 °C under an inert atmosphere was added a solution of 2,4,6-triisopropylbenzenesulfonyl chloride (0.45 g, 1.5 mmol, 1.0 equiv.) in Et_2O (1.1 mL) dropwise. After completion of addition, lithium aluminium hydride (4 M in Et_2O, 0.38 mL, 1.5 mmol, 1.0 equiv.) was added. The reaction mixture was allowed to warm to room temperature and was left to stir for 19.5 h. Upon completion, the reaction mixture was cooled to 0 °C and diluted with Et_2O (3 mL). The reaction was quenched with water (0.2 mL, 1 mL per gram of lithium aluminium hydride),

NaOH (aq., 15% w/w, 0.2 mL, 1 mL per gram of lithium aluminium hydride) and water (0.5 mL, 3 mL per gram of lithium aluminium hydride). The reaction mixture was stirred for 10 min at 0 °C before $MgSO_4$ was added. The resulting slurry was stirred for 30 min at room temperature. The white solids were then removed by filtration (rinsed with Et_2O). The solution was then concentrated *in vacuo*. Purification by column chromatography (SiO_2, pentane) afforded the title compound as a pale yellow oil (0.24 g, 1.0 mmol, 68%).

R_f 0.43 (pentane), [UV, vanillin]

^{1}H NMR (600 MHz, $CDCl_3$) δ 7.01 (s, 2H, Ar*H*), 3.51 (sept, *J* = 6.8 Hz, 2H, H1), 3.07 (s, 1H, S*H*), 2.87 (sept, *J* = 6.9 Hz, 1H, H3), 1.27 (d, *J* = 6.8 Hz, 12H, H2), 1.25 (d, *J* = 6.9 Hz, 6H, H4).

^{13}C NMR (151 MHz, $CDCl_3$) δ 148.2 (2C), 147.2, 124.4, 121.5 (2C), 34.3, 32.0 (2C), 24.2 (2C), 23.4 (4C).

Spectroscopic data in agreement with that reported previously.[17-18]

2-Bromo-5-iodo-1,3-diisopropylbenzene, S57

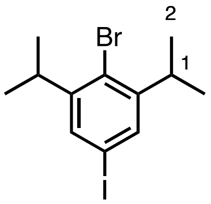

According to the procedure of Nitschke et al.[19] To a solution of 2,6-diisopropyl-aniline (1.89 mL, 10.0 mmol, 1.0 equiv.) in Et_2O (10 mL) at room temperature were added iodine (2.79 g, 11.0 mmol, 1.1 equiv.) and $NaHCO_3$ (aq., sat., 30 mL). The reaction mixture was left to stir at room temperature for 1.5 h, after which, $Na_2S_2O_3$ (500 mg) was added. The phases were separated and the aqueous phase was extracted with Et_2O (3 × 5 mL). The combined organic extracts were washed with water (20 mL), dried (Na_2SO_4) and concentrated *in vacuo* to afford 4-iodo-2,6-diisopropylaniline **S56** as a dark red/brown oil (2.91 g, 9.61 mmol, 96%).

According to the procedure of Yoshifuji et al.[20] To a solution of 4-iodo-2,6-diisopropylaniline **S56** (2.91 g, 9.61 mmol, 1.0 equiv.) in acetic acid (25 mL) and sulfuric acid (conc., 10 mL) at 0 °C was added isopentyl nitrite (2.45 mL, 18.3 mmol, 1.9 equiv.) dropwise. The reaction mixture was stirred at 0 °C for 30 min. The resulting solution was then added dropwise to a separate solution of copper(I) bromide (1.93 g, 13.5 mmol,

1.4 equiv.) in hydrobromic acid (47 – 49%, 35.6 mL) at 0 °C. The reaction mixture was allowed to warm to room temperature and left to stir for 19 h. The reaction mixture was then heated to 80 °C for 30 min. Upon completion, the reaction mixture was poured onto ice water (70 mL). The phases were separated and the aqueous phase was extracted with hexane (3 × 20 mL). The combined organic extracts were washed with $NaHSO_3$ (aq., sat., 50 mL) and brine (50 mL), dried (Na_2SO_4) and concentrated *in vacuo*. Purification by column chromatography (SiO_2, pentane) afforded the title compound as an orange oil (2.39 g, 6.50 mmol, 68%).

R*f* 0.71 (pentane), [UV, vanillin]
^{1}H NMR (600 MHz, $CDCl_3$) δ 7.39 (s, 2H, Ar*H*), 3.42 (sept, *J* = 6.9 Hz, 2H, H1), 1.22 (d, *J* = 6.9 Hz, 12H, H2).
^{13}C NMR (151 MHz, $CDCl_3$) δ 150.3 (2C), 133.5, 126.7 (2C), 93.7, 33.6 (2C), 23.0 (4C).
Spectroscopic data in agreement with that reported previously.[20]

2-Bromo-1,3-diisopropyl-5-methoxybenzene, S58

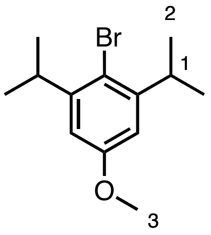

Adapted from the procedure of Buchwald et al.[21] To a solution of 2-bromo-5-iodo-1,3-diisopropylbenzene **S57** (0.37 g, 1.0 mmol, 1.0 equiv.) in MeOH (1.0 mL) at room temperature under an inert atmosphere were added copper(I) iodide (19 mg, 0.10 mmol, 0.1 equiv.), 1,10-phenanthroline (36 mg, 0.20 mmol, 0.2 equiv.) and caesium carbonate (0.46 g, 1.4 mmol, 1.4 equiv.) (in a 21 mL screw-capped vial). The vial was sealed and the reaction mixture was heated to 110 °C and stirred for 21 h. The reaction mixture was cooled to room temperature, MeOH (1.0 mL) was added and the mixture was then heated to 120 °C for 44 h. The resulting mixture was cooled to room temperature, filtered through a short pad of silica (eluting with Et_2O) and concentrated *in vacuo*. Purification by column chromatography (SiO_2, pentane) afforded the title compound as a pale yellow oil (0.12 g, 0.45 mmol, 45%).

R*f* 0.21 (pentane), [UV, phosphomolybdic acid]

^{1}H NMR (400 MHz, $CDCl_3$) δ 6.70 (s, 2H, Ar*H*), 3.81 (s, 3H, H3), 3.48 (sept, *J* = 6.8 Hz, 2H, H1), 1.23 (d, *J* = 6.8 Hz, 12H, H2).
^{13}C NMR (126 MHz, $CDCl_3$) δ 159.1, 149.0 (2C), 117.2, 110.0 (2C), 55.4, 33.7 (2C), 23.1 (4C).
Spectroscopic data in agreement with that reported previously.[22]

2,6-Diisopropyl-4-methoxybenzenethiol, S6

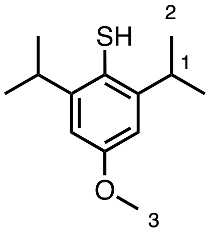

Magnesium turnings (41 mg, 1.7 mmol, 2.0 equiv.), 2-bromo-1,3-diisopropyl-5-methoxybenzene **S58** (0.23 g, 0.85 mmol, 1.0 equiv.), sulfur (29 mg, 0.89 mmol, 1.05 equiv.) and lithium aluminium hydride (4 M in Et_2O, 0.11 mL, 0.42 mmol, 0.5 equiv.) in THF (1.7 mL) were submitted to General Procedure C. Purification by column chromatography (SiO_2, pentane/Et_2O, 49:1) afforded the title compound as a colorless oil (0.15 g, 0.67 mmol, 79%).

R*f* 0.14 (pentane), [UV, vanillin]
^{1}H NMR (400 MHz, $CDCl_3$) δ 6.71 (s, 2H, Ar*H*), 3.81 (s, 3H, H3), 3.61 (sept, *J* = 6.8 Hz, 2H, H1), 2.87 (s, 1H, S*H*), 1.24 (d, *J* = 6.8 Hz, 12H, H2).
^{13}C NMR (126 MHz, $CDCl_3$) δ 159.2, 151.2 (2C), 117.5, 109.3 (2C), 55.3, 32.0 (2C), 23.3 (4C).
HRMS (ESI^+) Found $[M+H]^+$ = 225.1308; $C_{13}H_{21}OS$ requires 225.1308.
IR (film) ν_{max}/cm^{-1} 2961, 2869, 1595, 1460, 1431, 1332, 1279, 1197.

3,5-Di-*tert*-butylbenzenethiol, S7

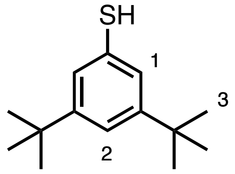

Magnesium turnings (0.11 g, 4.0 mmol, 2.0 equiv.), 1-bromo-3,5-di-*tert*-butyl-benzene (0.54 g, 2.0 mmol, 1.0 equiv.), sulfur (67 mg, 2.1 mmol, 1.05 equiv.) and lithium

aluminium hydride (1 M in THF, 1.0 mL, 1.0 mmol, 0.5 equiv.) in THF (4 mL) were submitted to General Procedure C. Purification by column chromatography (SiO_2, pentane) afforded the title compound as a colorless oil (0.32 g, 1.4 mmol, 71%).

R*f* 0.29 (pentane), [UV, vanillin]

^{1}H NMR (500 MHz, $CDCl_3$) δ 7.22 (t, *J* = 1.8 Hz, 1H, H2), 7.13 (d, *J* = 1.8 Hz, 2H, H1), 3.43 (s, 1H, S*H*), 1.30 (s, 18H, H3).

^{13}C NMR (126 MHz, $CDCl_3$) δ 151.9 (2C), 129.3, 124.1 (2C), 120.3, 35.0 (2C), 31.5 (6C).

HRMS (ESI^-) Found $[M–H]^-$ = 221.1368; $C_{14}H_{21}S$ requires 221.1369.

IR (film) ν_{max}/cm^{-1} 2963, 2905, 2868, 1591, 1575, 1477, 1431, 1363, 1286, 1248.

2,6-Di-*tert*-butyl-4-nitrophenol, S9

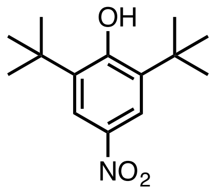

According to the procedure of Savinov et al.[23] To a solution of 2,6-di-*tert*-butylphenol (0.41 g, 2.0 mmol, 1.0 equiv.) in THF (10 mL) at room temperature was added *tert*-butyl nitrite (0.71 mL, 6.0 mmol, 3.0 equiv.). The reaction mixture was left to stir at room temperature for 23 h. Upon completion, the reaction mixture was concentrated *in vacuo*. Purification by column chromatography (SiO_2, pentane/PhMe, 4:1) afforded the title compound as a pale yellow solid (0.18 g, 0.73 mmol, 37%).

R*f* 0.21 (pentane/PhMe, 7:3), [UV, vanillin]

^{1}H NMR (500 MHz, $CDCl_3$) δ 8.12 (s, 2H, Ar*H*), 5.93 (s, 1H, O*H*), 1.48 (s, 18H, *o*-C(CH_3)$_3$).

^{13}C NMR (126 MHz, $CDCl_3$) δ 159.7, 140.9, 136.8 (2C), 121.6 (2C), 34.7 (2C), 30.1 (6C).

Spectroscopic data in agreement with that reported previously.[24]

2,4,6-Triisopropyl-*N*-(pyridin-3-yl)benzenesulfonamide, S10

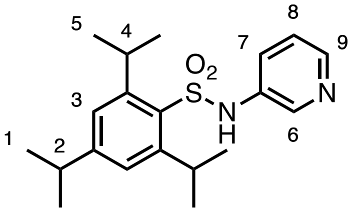

Adapted from the procedure of Kanai et al.[25] To a solution of 2,4,6-triisopropylbenzenesulfonyl chloride (0.30 g, 1.0 mmol, 1.0 equiv.) and pyridine (0.24 mL, 3.0 mmol, 3.0 equiv.) in THF (5 mL) at room temperature under an inert atmosphere was added 3-aminopyridine (0.14 g, 1.5 mmol, 1.5 equiv.). The reaction mixture was left to stir at room temperature for 24 h and then heated to 70 °C for 21 h. The reaction mixture was then cooled to room temperature and concentrated *in vacuo*. Purification by column chromatography (SiO_2, pentane/Et_2O, 1:4) afforded the title compound as a white solid (0.27 g, 0.74 mmol, 74%).

R*f* 0.45 (Et_2O), [UV, vanillin]
m.p. 174 – 175 °C
^{1}H NMR (500 MHz, $CDCl_3$) δ 8.39 (dd, *J* = 4.8, 1.5 Hz, 1H, H6), 8.17 (dd, *J* = 2.7, 0.8 Hz, 1H, H9), 7.52 (ddd, *J* = 8.3, 2.7, 1.5 Hz, 1H, H8), 7.24 (ddd, *J* = 8.3, 4.8, 0.8 Hz, 1H, H7), 7.13 (s, 2H, H3), 6.59 (s, 1H, N*H*), 3.97 (sept, *J* = 6.8 Hz, 2H, H4), 2.88 (sept, *J* = 7.0 Hz, 1H, H2), 1.23 (d, *J* = 7.0 Hz, 6H, H1), 1.17 (d, *J* = 6.8 Hz, 12H, H5).
^{13}C NMR (126 MHz, $CDCl_3$) δ 153.7, 150.6 (2C), 147.2, 144.6, 133.3, 131.7, 131.0, 124.3 (2C), 124.0, 34.3, 30.1 (2C), 24.9 (4C), 23.7 (2C).
HRMS (ESI^+) Found $[M+H]^+$ = 361.1946; $C_{20}H_{29}O_2N_2S$ requires 361.1944.
IR (film) ν_{max}/cm^{-1} 2960, 2869, 1599, 1463, 1425, 1385, 1364, 1314, 1153.

2-Phenylmalononitrile, S13

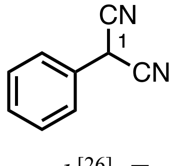

According to the procedure of Mayr et al.[26] To a solution of copper iodide (38 mg, 0.20 mmol, 0.2 equiv.), L-proline (23 mg, 0.20 mmol, 0.2 equiv.), potassium carbonate (0.55 g, 4.0 mmol, 4.0 equiv.) and malononitrile (0.20 g, 3.0 mmol, 3.0 equiv.) in DMSO (5 mL) at room temperature under an inert atmosphere was added iodo-

benzene (0.11 mL, 1.0 mmol, 1.0 equiv.). The reaction mixture was heated to 90 °C and left to stir for 41.5 h. The reaction mixture was then cooled to 0 °C and acidified to pH 3 – 4 with HCl (aq., 2M). The phases were separated and the aqueous phase was extracted with EtOAc (3 × 4 mL). The combined organic extracts were washed with brine (10 mL), dried (Na_2SO_4), filtered and concentrated *in vacuo*. Purification by column chromatography (SiO_2, pentane/Et_2O, 7:3) afforded the title compound as a white solid (91 mg, 0.64 mmol, 64%).

R*f* 0.29 (pentane/Et_2O, 7:3), [UV, potassium permanganate]
^{1}H NMR (400 MHz, $CDCl_3$) δ 7.53 – 7.49 (m, 5H, Ar*H*), 5.06 (s, 1H, H1).
^{13}C NMR (126 MHz, $CDCl_3$) δ 130.6, 130.2 (2C), 127.4 (2C), 126.3, 111.8 (2C), 28.3.
Spectroscopic data in agreement with that reported previously.[9]

2-(4-Cyanophenyl)malononitrile, S14

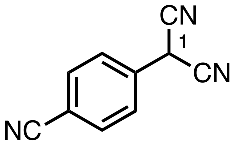

Sodium hydride (60% dispersion in mineral oil, 0.12 g, 3.0 mmol, 3.0 equiv.), malono-nitrile (0.13 g, 2.0 mmol, 2.0 equiv.), $Pd(PPh_3)Cl_2$ (21 mg, 0.030 mmol, 0.03 equiv.) and 4-iodobenzonitrile (0.23 g, 1.0 mmol, 1.0 equiv.) in THF (2 mL) were submitted to General Procedure D. Purification by column chromatography (SiO_2, pentane/Et_2O, 7:3) afforded the title compound as an orange solid (19 mg, 0.11 mmol, 11%).

R*f* 0.05 (Et_2O), [UV, potassium permanganate]
^{1}H NMR (500 MHz, $CDCl_3$) δ 7.84 (d, *J* = 8.4 Hz, 2H, Ar*H*), 7.69 (d, *J* = 8.4 Hz, 2H, Ar*H*), 5.16 (s, 1H, H1).
^{13}C NMR (126 MHz, $CDCl_3$) δ 133.9 (2C), 131.0, 128.3 (2C), 117.3, 115.1, 110.7 (2C), 28.2.
Spectroscopic data in agreement with that reported previously.[27]

2-(4-Nitrophenyl)malononitrile, S15

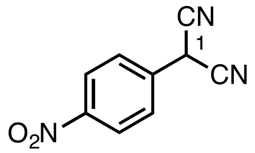

Sodium hydride (60% dispersion in mineral oil, 0.12 g, 3.0 mmol, 3.0 equiv.), malono-nitrile (0.13 g, 2.0 mmol, 2.0 equiv.), $Pd(PPh_3)Cl_2$ (21 mg, 0.030 mmol, 0.03 equiv.) and 1-iodo-4-nitrobenzene (0.25 g, 1.0 mmol, 1.0 equiv.) in THF (2 mL) were submitted to General Procedure D. Purification by column chromatography (SiO_2, pentane/Et_2O, 7:3 → EtOAc) afforded the title compound as a red solid (0.18 g, 0.96 mmol, 96%).

R*f* 0.10 (EtOAc), [UV, potassium permanganate]

^{1}H NMR (500 MHz, $CDCl_3$) δ 8.40 (d, *J* = 8.8 Hz, 2H, Ar*H*), 7.76 (d, *J* = 8.8 Hz, 2H, Ar*H*), 5.21 (s, 1H, H1).

^{13}C NMR (126 MHz, $CDCl_3$) δ 153.7, 132.3, 128.7 (2C), 125.4 (2C), 110.7 (2C), 28.0.

Spectroscopic data in agreement with that reported previously.[28]

2-(4-Methoxyphenyl)malononitrile, S16

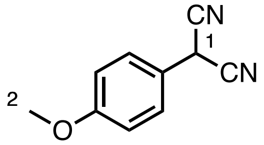

Sodium hydride (60% dispersion in mineral oil, 0.12 g, 3.0 mmol, 3.0 equiv.), malono-nitrile (0.13 g, 2.0 mmol, 2.0 equiv.), $Pd(PPh_3)Cl_2$ (21 mg, 0.030 mmol, 0.03 equiv.) and 1-iodoanisole (0.23 g, 1.0 mmol, 1.0 equiv.) in THF (2 mL) were submitted to General Procedure D. Purification by column chromatography (SiO_2, pentane/Et_2O, 4:1) afforded the title compound as an orange solid (0.14 g, 0.82 mmol, 82%).

R*f* 0.31 (pentane/Et_2O, 3:2), [UV, vanillin]

^{1}H NMR (400 MHz, $CDCl_3$) δ 7.41 (d, *J* = 8.9 Hz, 2H, Ar*H*), 6.99 (d, *J* = 8.9 Hz, 2H), 4.99 (s, 1H, H1), 3.85 (s, 3H, H2).

^{13}C NMR (126 MHz, $CDCl_3$) δ 161.2, 128.7 (2C), 118.0, 115.5 (2C), 112.1 (2C), 55.7, 27.6.

Spectroscopic data in agreement with that reported previously.[29]

[1,1′-Biphenyl]-2-yl(phenyl)methanone, S59

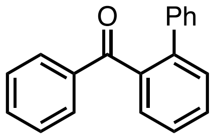

Adapted from the procedure of Itoi et al.[30] To a solution of 2-bromobenzophenone (0.18 mL, 1.0 mmol, 1.0 equiv.) in THF : water (6.4 mL : 1.6 mL) at room temperature under an inert atmosphere was added phenylboronic acid (0.15 g, 1.2 mmol, 1.2 equiv.), $Pd(PPh_3)_4$ (0.12 g, 0.10 mmol, 0.1 equiv.) and potassium carbonate (0.42 g, 3.0 mmol, 3.0 equiv.). The reaction mixture was heated to 80 °C and left to stir for 15 h. After cooling to room temperature, the phases were separated and the aqueous phase was extracted with CH_2Cl_2 (3 × 2 mL). The combined organic extracts were dried (Na_2SO_4) and concentrated *in vacuo*. Purification by column chromatography (SiO_2, pentane/Et_2O, 19:1) afforded the title compound as a pale yellow oil (0.15 g, 0.57 mmol, 57%).

R*f* 0.24 (pentane/CH_2Cl_2, 1:1), [UV, phosphomolybdic acid]
^{1}H NMR (400 MHz, $CDCl_3$) δ 7.66 – 7.62 (m, 2H, Ar*H*), 7.61 – 7.55 (m, 1H, Ar*H*), 7.54 – 7.38 (m, 4H, Ar*H*), 7.29 – 7.24 (m, 4H, Ar*H*), 7.22 – 7.12 (m, 3H, Ar*H*).
^{13}C NMR (101 MHz, $CDCl_3$) δ 198.9, 141.3, 140.3, 139.1, 137.6, 132.9, 130.5, 130.2, 130.0 (2C), 129.1 (2C), 128.9, 128.4 (2C), 128.2 (2C), 127.5, 127.2.
Spectroscopic data in agreement with that reported previously.[31]

9-Phenyl-9*H*-fluorene, S17

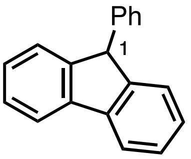

To a rapidly stirred suspension of *p*-toluenesulfonyl hydrazide (88 mg, 0.47 mmol, 1.0 equiv.) in MeOH (0.5 mL) at 60 °C under an inert atmosphere was added [1,1′-biphenyl]-2-yl(phenyl)methanone **S59** (0.12 g, 0.47 mmol, 1.0 equiv.) as a solution in MeOH (0.5 mL) dropwise. The reaction mixture was heated to 60 °C and left to stir for 22.5 h. The reaction mixture was then concentrated *in vacuo*. The residue was dissolved in CH_2Cl_2 (1 mL) and filtered through a short pad of silica (eluting with pentane/Et_2O, 19:1 to 7:3) to afford (*E*)-*N*′-([1,1′-biphenyl]-2-yl(phenyl)methylene)-4-methylbenzenesulfonohydrazide **S60** as a pale pink foam (46 mg, 0.11 mmol, 23%; 71% based on recovered starting material).

According to the procedure of Wang et al.[32] To a solution of (*E*)-*N*′-([1,1′-biphenyl]-2-yl(phenyl)methylene)-4-methylbenzenesulfonohydrazide **S60** (46 mg, 0.11 mmol, 1.0 equiv.) in PhMe (1 mL) at room temperature under an inert atmosphere was added sodium hydride (60% dispersion in mineral oil, 5.2 mg, 0.13 mmol, 1.2 equiv.). The reaction mixture was then heated to 110 °C and left to stir for 5 h. Upon completion, the reaction was concentrated *in vacuo*. Purification by column chromatography (SiO_2, pentane/Et_2O, 19:1) afforded the title compound as a white solid (23 mg, 0.095 mmol, 88%).

R*f* 0.71 (pentane/Et_2O, 4:1), [UV, vanillin]
^{1}H NMR (600 MHz, $CDCl_3$) δ 7.80 (d, *J* = 7.6 Hz, 2H, Ar*H*), 7.38 (t, *J* = 7.4 Hz, 2H, Ar*H*), 7.32 (dd, *J* = 7.6, 1.2 Hz, 2H, Ar*H*), 7.29 – 7.20 (m, 5H, Ar*H*), 7.09 (d, *J* = 6.9 Hz, 2H, Ar*H*), 5.05 (s, 1H, H1).
^{13}C NMR (151 MHz, $CDCl_3$) δ 148.0 (2C), 141.7, 141.2 (2C), 128.8 (2C), 128.5 (2C), 127.4 (4C), 127.0, 125.5 (2C), 120.0 (2C), 54.6.
Spectroscopic data in agreement with that reported previously.[32]

(*E*)-1-(2-Bromophenyl)-3-methyl-3-phenyltriaz-1-ene, S61

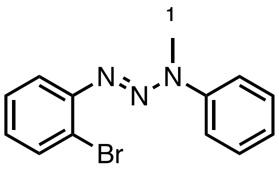

According to the procedure of Ren et al.[33] To a solution of 2-bromoaniline (0.23 mL, 2.0 mmol, 1.0 equiv.) in MeCN (2 mL) at room temperature was added HCl (conc., 0.80 mL, 10 mmol, 5.0 equiv.). The reaction mixture was cooled to –10 °C and stirred for 15 min. A solution of sodium nitrite (0.15 g, 2.1 mmol, 1.05 equiv.) in cold water (2 mL) was then added to the reaction mixture dropwise. The resulting solution was stirred at –10 °C for 30 min and then added to a solution of *N*-methylaniline (0.24 mL, 2.2 mmol, 1.1 equiv.) and potassium carbonate (0.69 g, 5.0 mmol, 2.5 equiv.) in MeCN : water (2 mL : 4 mL), which was previously cooled to –10 °C. The resulting solution was allowed to warm to room temperature over 2 h. Upon completion, the phases were separated and the aqueous phase was extracted with EtOAc (3 × 5 mL). The combined organic extracts were dried (Na_2SO_4) and concentrated *in vacuo*. Purification by column chromatography (SiO_2, pentane/Et_2O, 19:1) afforded the title compound as a bright orange oil (0.56 g, 1.9 mmol, 97%).

R$_f$ 0.71 (pentane/Et_2O, 4:1), [UV, vanillin]

^{1}H NMR (400 MHz, $CDCl_3$) δ 7.64 (dd, *J* = 8.0, 1.4 Hz, 1H, Ar*H*), 7.56 (dd, *J* = 8.1, 1.6 Hz, 1H, Ar*H*), 7.51 – 7.46 (m, 2H, Ar*H*), 7.44 – 7.37 (m, 2H, Ar*H*), 7.31 (ddd, *J* = 8.0, 7.2, 1.4 Hz, 1H, Ar*H*), 7.16 (t, *J* = 7.3 Hz, 1H, Ar*H*), 7.08 (td, *J* = 8.0, 7.2, 1.6 Hz, 1H, Ar*H*), 3.73 (s, 3H, H1).

^{13}C NMR (126 MHz, $CDCl_3$) δ 147.9, 145.1, 133.4, 129.4 (2C), 128.0, 127.6, 124.2, 120.9, 119.3, 117.5 (2C), 33.3.

Spectroscopic data in agreement with that reported previously.[33]

1-Phenyl-1*H*-benzo[*d*][1,2,3]triazole, S62

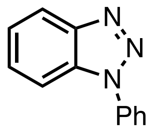

Adapted from the procedure of Ren et al.[33] To a solution of (*E*)-1-(2-bromophenyl)-3-methyl-3-phenyltriaz-1-ene **S61** (0.56 g, 1.9 mmol, 1.0 equiv.) in DMF (8 mL) at room temperature under an inert atmosphere were added $Pd(OAc)_2$ (22 mg, 0.097 mmol, 0.05 equiv.), 1,3-bis(diphenylphosphino)propane (dppp) (80 mg, 0.19 mmol, 0.1 equiv.) and potassium acetate (0.23 g, 2.3 mmol, 1.2 equiv.). The reaction mixture was heated to 110 °C and left to stir for 23 h. The reaction mixture was then heated to 120 °C and left to stir for 17.5 h. The reaction mixture was then cooled to room temperature and water (5 mL) was added. The phases were separated and the aqueous phase was extracted with EtOAc (3 × 5 mL). The combined organic extracts were dried (Na_2SO_4) and concentrated *in vacuo*. Purification by column chromatography (SiO_2, pentane/Et_2O, 9:1) afforded the title compound as a pale yellow solid (0.24 g, 1.2 mmol, 63%).

R$_f$ 0.14 (pentane/Et_2O, 19:1), [UV, vanillin]

^{1}H NMR (400 MHz, $CDCl_3$) δ 8.16 (dt, *J* = 8.2, 1.0 Hz, 1H, Ar*H*), 7.83 – 7.74 (m, 3H, Ar*H*), 7.66 – 7.60 (m, 2H, Ar*H*), 7.59 – 7.49 (m, 2H, Ar*H*), 7.45 (ddd, *J* = 8.2, 7.0, 1.0 Hz, 1H, Ar*H*).

^{13}C NMR (126 MHz, $CDCl_3$) δ 146.7, 137.2, 132.5, 130.0 (2C), 128.8, 128.4, 124.5, 123.1 (2C), 120.5, 110.5.

Spectroscopic data in agreement with that reported previously.[33]

1,2,3-Triazole-borane, S18

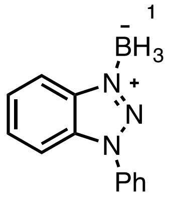

Adapted from the procedure of Shi et al.[34] To a solution of 1-phenyl-1*H*-benzo[*d*][1,2,3]triazole **S62** (0.24 g, 1.2 mmol, 1.0 equiv.) in THF (1.2 mL) at room temperature under an inert atmosphere was added borane-tetrahydrofuran (1 M in THF, 1.3 mL, 1.3 mmol, 1.1 equiv.) dropwise. The reaction mixture was left to stir at room temperature for 28 h, after which borane-tetrahydrofuran (1 M in THF, 1.2 mL, 1.2 mmol, 1.0 equiv.) was added and the resulting mixture left to stir at room temperature for 19.5 h. The reaction mixture was then concentrated *in vacuo*. The residue was dissolved in CH_2Cl_2 (0.5 mL) and filtered through a short pad of silica (eluting with pentane/Et_2O, 3:2) to afford the title compound as a white solid (32 mg, 0.15 mmol, 13%).

R_f 0.17 (pentane/Et_2O, 3:2), [UV, vanillin]

^{1}H NMR (500 MHz, $CDCl_3$) δ 8.33 (dt, J = 8.1, 1.1 Hz, 1H, Ar*H*), 7.82 – 7.77 (m, 3H, Ar*H*), 7.75 – 7.71 (m, 1H, Ar*H*), 7.71 – 7.66 (m, 3H, Ar*H*), 7.66 – 7.62 (m, 1H, Ar*H*), 3.14 – 2.59 (br m, 3H, H1).

^{13}C NMR (126 MHz, $CDCl_3$) δ 140.4, 135.5, 133.3, 130.7, 130.4 (2C), 130.2, 127.8, 123.8 (2C), 118.7, 111.5.

Spectroscopic data in agreement with that reported previously.[34]

3.2.4 Organocatalysts

Organocatalysts **9** and **S38** are commercially available and used directly as supplied.

(*S*)-2-(Bis(3,5-bis(trifluoromethyl)phenyl)methyl)pyrrolidine, S31

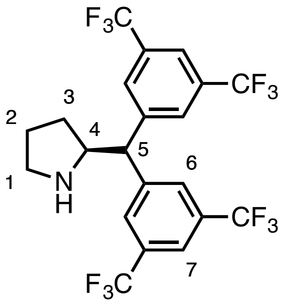

According to the procedure of Lin et al.[35] To a solution of L-proline (2.3 g, 20 mmol, 1.0 equiv.) and potassium carbonate (5.5 g, 40 mmol, 2.0 equiv.) in MeOH (40 mL) at 0 °C under an inert atmosphere was added ethyl chloroformate (4.6 mL, 48 mmol, 2.4 equiv.). The reaction mixture was allowed to warm to room temperature and left to stir for 22 h. The reaction mixture was then concentrated *in vacuo*. Water (10 mL) and $NaHCO_3$ (aq., sat., 10 mL) were added to the resulting residue and the aqueous phase was extracted with EtOAc (3 × 15 mL). The combined organic extracts were dried (Na_2SO_4) and concentrated *in vacuo* to afford 1-ethyl 2-methyl (*S*)-pyrrolidine-1,2-dicarboxylate **S63** as a pale yellow oil (2.8 g, 14 mmol, 70%).

According to the procedure of Matsubara et al.[36] To a solution of 1,3-bis(trifluoromethyl)-5-bromobenzene (0.52 mL, 3.0 mmol, 3.0 equiv.) in THF (2.5 mL) at 0 °C under an inert atmosphere was added isopropylmagnesium chloride (2 M in THF, 1.6 mL, 3.2 mmol, 3.15 equiv.) dropwise. The resulting mixture was stirred for 1 h at 0 °C. A solution of 1-ethyl 2-methyl (*S*)-pyrrolidine-1,2-dicarboxylate **S63** (0.20 g, 1.0 mmol, 1.0 equiv.) in THF (4.5 mL) was then added dropwise at 0 °C. The reaction mixture was then heated to 65 °C and stirred at this temperature for 3 h. The reaction mixture was then cooled to room temperature and NH_4Cl (aq., sat., 7 mL) was added. The phases were separated and the aqueous phase was extracted with EtOAc (3 × 5 mL). The combined organic extracts were dried (Na_2SO_4) and concentrated *in vacuo* to afford (*S*)-1,1-bis(3,5-bis(trifluoromethyl)phenyl)tetrahydro-1*H*,3*H*-pyrrolo[1,2-*c*]oxazol-3-one **S64** as a pale yellow oil (0.52 g, 0.94 mmol, 94%).

According to the procedure of Dixon et al.[37] To a solution of (*S*)-1,1-bis(3,5-bis(trifluoromethyl)phenyl)tetrahydro-1*H*,3*H*-pyrrolo[1,2-*c*]oxazol-3-one **S64** (0.49 g,

0.89 mmol, 1.0 equiv.) in ethanol (16.9 mL) containing 5% Et_3N (0.9 mL) at room temperature under an inert atmosphere was added Pd/C (10% wt., 89 mg, 100 mg/mmol of **S64**) portionwise. The reaction vial was put under vacuum and back-filled with hydrogen three times and the reaction mixture was left to stir at 40 °C under a hydrogen atmosphere for 20 h. The reaction mixture was then filtered through a pad of Celite® and the Celite® was washed with ethanol. The filtrate was then concentrated *in vacuo*. Purification by column chromatography (SiO_2, pentane/Et_2O, 7:3) afforded the title compound as a pale yellow oil (0.23 g, 0.45 mmol, 50%).

$\mathbf{R}_f$ 0.31 (pentane/Et_2O, 4:1), [UV, vanillin]
^{1}H NMR (500 MHz, $CDCl_3$) δ 7.81 (s, 2H, H6), 7.78 (s, 2H, H7), 7.73 (s, 2H, H6), 4.04 (d, J = 9.3 Hz, 1H, H5), 3.91 (ddd, J = 9.3, 7.7, 6.5 Hz, 1H, H4), 3.03 – 2.91 (m, 2H, H1), 1.88 – 1.71 (m, 4H, H2, H3 and N*H*), 1.41 – 1.30 (m, 1H, H3).
^{13}C NMR (126 MHz, $CDCl_3$) δ 144.5 (2C), 132.4 (q, J = 33.3 Hz, 2C), 132.3 (q, J = 33.3 Hz, 2C), 128.6 (d, J = 3.8 Hz, 2C), 128.3 (d, J = 3.8 Hz, 2C), 123.3 (q, J = 273.0 Hz, 2C), 123.2 (q, J = 273.0 Hz, 2C), 121.6 – 121.3 (m, 2C), 61.9, 57.9, 46.8, 31.0, 25.5.
Spectroscopic data in agreement with that reported previously.[37]

(*S*)-Bis(3,5-bis(trifluoromethyl)phenyl)(pyrrolidin-2-yl)methanol, S32

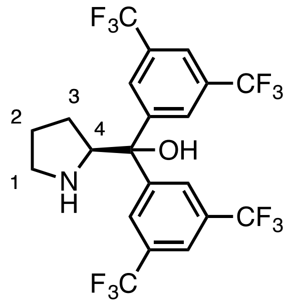

Adapted from the procedure of Matsubara et al.[36] To a solution of (*S*)-1,1-bis(3,5-bis(trifluoromethyl)phenyl)tetrahydro-1*H*,3*H*-pyrrolo[1,2-*c*]oxazol-3-one **S64** (0.52 g, 0.94 mmol, 1.0 equiv.) in MeOH (4 mL) at room temperature was added potassium hydroxide (0.16 g, 2.8 mmol, 3.0 equiv.). The resulting mixture was heated to 65 °C and left to stir for 18 h. The reaction mixture was then heated to 75 °C for 6 h. Potassium hydroxide (0.37 g, 6.6 mmol, 7.0 equiv.) was then added and the reaction mixture left to stir at 75 °C for 17 h. The reaction mixture was then cooled to room temperature and concentrated *in vacuo*. The resulting residue was diluted with water (4 mL) and extracted with EtOAc (3 × 3 mL). The combined organic extracts were washed with brine (5 mL),

dried (Na_2SO_4), filtered and concentrated *in vacuo*. Purification by column chromatography (SiO_2, pentane/Et_2O, 7:3) afforded the title compound as a pale yellow oil (0.31 g, 0.58 mmol, 62%).

R*f* 0.24 (pentane/Et_2O, 7:3), [UV, vanillin]

^{1}H NMR (500 MHz, $CDCl_3$) δ 8.04 (s, 2H, Ar*H*), 7.96 (s, 2H, Ar*H*), 7.76 (d, *J* = 5.2 Hz, 2H, Ar*H*), 5.06 (br s, 1H, O*H*), 4.34 (t, *J* = 7.7 Hz, 1H, H4), 3.14 – 3.00 (m, 2H, H1), 1.85 – 1.46 (m, 5H, H2, H3 and N*H*).

^{13}C NMR (126 MHz, $CDCl_3$) δ 149.5, 146.6, 132.2 (q, *J* = 33.4 Hz, 2C), 131.8 (q, *J* = 32.9 Hz, 2C), 126.2 (d, *J* = 3.9 Hz, 2C), 125.8 (d, *J* = 4.1 Hz, 2C), 123.3 (q, *J* = 272.6 Hz, 2C), 123.3 (q, *J* = 273.1 Hz, 2C), 121.7 (t, *J* = 3.9 Hz), 121.5 (t, *J* = 3.9 Hz), 76.8, 64.4, 47.1, 26.9, 25.7.

Spectroscopic data in agreement with that reported previously.[38]

(*S*)-2-(Bis(3,5-bis(trifluoromethyl)phenyl)((triethylsilyl)oxy)methyl)pyrrolidine, S33

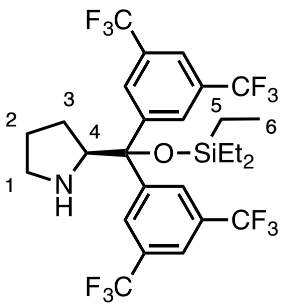

According to the procedure of Lin et al.[35] To a solution of (*S*)-bis(3,5-bis(trifluoromethyl)phenyl)(pyrrolidin-2-yl)methanol **S32** (79 mg, 0.15 mmol, 1.0 equiv.) and 2,6-lutidine (90 µL, 0.75 mmol, 5.0 equiv.) in CH_2Cl_2 (0.3 mL) at 0 °C under an inert atmosphere was added triethylsilyl trifluoromethanesulfonate (0.10 mL, 0.45 mmol, 3.0 equiv.). The reaction mixture was allowed to warm to room temperature and left to stir for 28 h. Upon completion, $NaHCO_3$ (aq., sat., 1 mL) was added. The phases were separated and the aqueous phase was extracted with EtOAc (3 × 1 mL). The combined organic extracts were dried (Na_2SO_4) and concentrated *in vacuo*. Purification by column chromatography (SiO_2, pentane → pentane/Et_2O, 9:1) afforded the title compound as a colorless oil (39 mg, 0.061 mmol, 40%).

R*f* 0.55 (pentane/Et_2O, 9:1), [UV, vanillin]

^{1}H NMR (500 MHz, $CDCl_3$) δ 8.06 (s, 2H, Ar*H*), 7.85 (s, 2H, Ar*H*), 7.75 (s, 2H, Ar*H*), 4.22 (dd, *J* = 8.0, 6.3 Hz, 1H, H4), 2.90 (dt, *J* = 10.2, 6.8 Hz, 1H, H1), 2.52 (ddd, *J* = 10.2, 6.8, 5.6 Hz, 1H, H1), 1.81 – 1.69 (m, 2H, H3 and N*H*), 1.53 – 1.38 (m, 2H, H2 and H3), 1.01 – 0.92 (m, 1H, H2), 0.84 (t, *J* = 7.9 Hz, 9H, H6), 0.39 – 0.25 (m, 6H, H5).
^{13}C NMR (126 MHz, $CDCl_3$) δ 148.1, 146.3, 131.6 (q, *J* = 33.4 Hz, 2C), 130.7 (q, *J* = 33.3 Hz, 2C), 129.1 (d, *J* = 3.8 Hz, 2C), 128.6 (d, *J* = 4.0 Hz, 2C), 123.5 (q, *J* = 272.8 Hz, 2C), 123.3 (q, *J* = 272.9 Hz, 2C), 121.9 (quin, *J* = 3.9 Hz), 121.6 (quin, *J* = 3.9 Hz), 82.3, 64.5, 47.5, 27.9, 25.6, 7.0 (3C), 6.4 (3C).
Spectroscopic data in agreement with that reported previously.[39]

(*S*)-2-(Bis(3,5-bis(trifluoromethyl)phenyl)((*tert*-butyldimethylsilyl)oxy)methyl)pyrrolidine, S34

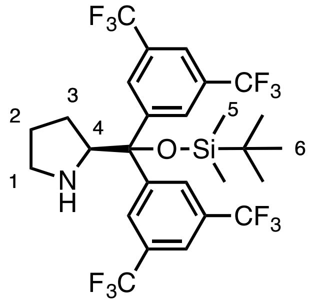

According to the procedure of Lin et al.[35] To a solution of (*S*)-bis(3,5-bis(trifluoromethyl)phenyl)(pyrrolidin-2-yl)methanol **S32** (79 mg, 0.15 mmol, 1.0 equiv.) and Et_3N (0.13 mL, 0.90 mmol, 6.0 equiv.) in CH_2Cl_2 (0.4 mL) at 0 °C under an inert atmosphere was added *tert*-butyldimethylsilyl trifluoromethanesulfonate (0.10 mL, 0.45 mmol, 3.0 equiv.). The reaction mixture was then heated to 100 °C and left to stir for 4 h. The reaction mixture was then cooled to room temperature and water (1 mL) was added. The phases were separated and the aqueous phase was extracted with EtOAc (3 × 1 mL). The combined organic extracts were dried (Na_2SO_4) and concentrated *in vacuo*. Purification by column chromatography (SiO_2, pentane/Et_2O, 19:1) afforded the title compound as a pale yellow oil (51 mg, 0.080 mmol, 53%).

R_f 0.64 (pentane/Et_2O, 9:1), [UV, vanillin]
^{1}H NMR (500 MHz, $CDCl_3$) δ 8.09 (s, 2H, Ar*H*), 7.85 (s, 2H, Ar*H*), 7.73 (s, 2H, Ar*H*), 4.23 (dd, *J* = 8.0, 5.9 Hz, 1H, H4), 2.89 (dt, *J* = 10.2, 7.0 Hz, 1H, H1), 2.53 (ddd, *J* = 10.2, 7.0, 5.2 Hz, 1H, H1), 1.87 – 1.71 (m, 2H, H3 and N*H*), 1.51 – 1.41 (m, 2H, H2 and H3), 0.94 (s, 9H, H6), 0.92 – 0.88 (m, 1H, H2), –0.21 (s, 3H, H5), –0.47 (s, 3H, H5).

^{13}**C NMR** (126 MHz, $CDCl_3$) δ 147.9, 146.2, 131.7 (q, J = 33.4 Hz, 2C), 130.7 (q, J = 33.3 Hz, 2C), 129.2 (2C), 128.9 (2C), 123.5 (q, J = 272.7 Hz, 2C), 123.3 (q, J = 273.0 Hz, 2C), 122.0 – 121.8 (m), 121.6 (t, J = 3.6 Hz), 82.5, 64.1, 47.4, 28.0, 26.0 (3C), 25.4, 19.0, –2.6, –3.2.

Spectroscopic data in agreement with that reported previously.[35]

(*S*)-2-(Diphenyl((trimethylsilyl)oxy)methyl)pyrrolidine, S35

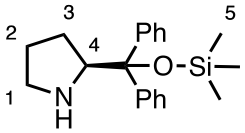

According to the procedure of Bode et al.[40] To a solution of (*S*)-diphenylprolinol (0.51 g, 2.0 mmol, 1.0 equiv.) in CH_2Cl_2 (7.6 mL) at 0 °C under an inert atmosphere was added imidazole (0.41 g, 6.0 mmol, 3.0 equiv.). Trimethylsilyl chloride (0.63 mL, 5.0 mmol, 2.5 equiv.) was then added dropwise and the reaction mixture was allowed to warm to room temperature. The reaction mixture was left to stir for 21 h. MTBE (13 mL) was then added and the reaction mixture was filtered. The organic phase was washed with water (10 mL) and brine (10 mL), dried (Na_2SO_4), filtered and concentrated *in vacuo* to afford the title compound as a colorless oil (0.53 g, 1.6 mmol, 81%).

$\mathbf{R}_f$ 0.17 (pentane/EtOAc, 1:1), [UV, vanillin]

^{1}**H NMR** (500 MHz, $CDCl_3$) δ 7.47 – 7.43 (m, 2H, Ar*H*), 7.37 – 7.33 (m, 2H, Ar*H*), 7.31 – 7.19 (m, 6H, Ar*H*), 4.16 – 4.07 (m, 1H, H4), 2.95 – 2.86 (m, 1H, H1), 2.80 – 2.72 (m, 1H, H1), 1.67 – 1.57 (m, 3H, H2 and H3), 1.44 – 1.34 (m, 1H, H2), –0.09 (s, 9H, H5).

Note: the N*H* resonance was not observed.

^{13}**C NMR** (126 MHz, $CDCl_3$) δ 146.4, 145.5, 128.6 (2C), 127.9 (2C), 127.8 (2C), 127.7 (2C), 127.3, 127.1, 83.2, 65.6, 47.2, 27.6, 25.1, 2.3 (3C).

Spectroscopic data in agreement with that reported previously.[40]

(*S*)-1,1-Bis(3,5-dimethylphenyl)tetrahydro-1*H*,3*H*-pyrrolo[1,2-*c*]oxazol-3-one, S65

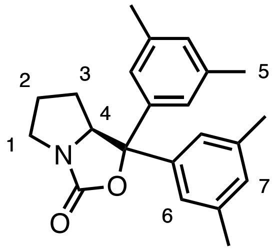

Adapted from the procedure of Matsubara et al.[36] To a solution of 1-iodo-3,5-dimethylbenzene (0.43 mL, 3.0 mmol, 3.0 equiv.) in THF (2.5 mL) at 0 °C under an inert atmosphere was added isopropylmagnesium chloride (2 M in THF, 1.6 mL, 3.2 mmol, 3.15 equiv.) dropwise. The resulting mixture was stirred for 1 h at 0 °C. A solution of 1-ethyl 2-methyl (*S*)-pyrrolidine-1,2-dicarboxylate **S63** (0.20 g, 1.0 mmol, 1.0 equiv.) in THF (4.5 mL) was then added dropwise at 0 °C. The reaction mixture was then heated to 65 °C and stirred at this temperature for 26 h. The reaction mixture was then cooled to room temperature and NH_4Cl (aq., sat., 7 mL) was added. The phases were separated and the aqueous phase was extracted with EtOAc (3 × 5 mL). The combined organic extracts were dried (Na_2SO_4) and concentrated *in vacuo*. Purification by column chromatography (SiO_2, pentane/Et_2O, 7:3) afforded the title compound as a red oil (0.33 g, 0.99 mmol, 99%).

R_f 0.38 (pentane/Et_2O, 3:2), [UV, phosphomolybdic acid]

^{1}H NMR (400 MHz, $CDCl_3$) δ 7.13 (s, 2H, H6), 6.98 (s, 2H, H6), 6.93 (dt, *J* = 1.5, 0.8 Hz, 1H, H7), 6.89 (dt, *J* = 1.7, 0.8 Hz, 1H, H7), 4.50 (dd, *J* = 10.7, 5.4 Hz, 1H, H4), 3.72 (dt, *J* = 11.5, 8.1 Hz, 1H, H1), 3.23 (ddd, *J* = 11.5, 9.5, 3.7 Hz, 1H, H1), 2.30 (d, *J* = 0.8 Hz, 6H, H5), 2.29 (d, *J* = 0.8 Hz, 6H, H5), 2.03 – 1.92 (m, 1H, H2), 1.90 – 1.80 (m, 1H, H2), 1.75 – 1.66 (m, 1H, H3), 1.12 (dtd, *J* = 12.5, 10.7, 8.7 Hz, 1H, H3).

^{13}C NMR (126 MHz, $CDCl_3$) δ 160.9, 143.5, 140.6, 138.2 (2C), 137.9 (2C), 130.0, 129.3, 123.7 (2C), 123.3 (2C), 86.0, 69.3, 46.2, 29.1, 25.0, 21.6 (2C), 21.6 (2C).

Spectroscopic data in agreement with that reported previously.[37]

(*S*)-Bis(3,5-dimethylphenyl)(pyrrolidin-2-yl)methanol, S66

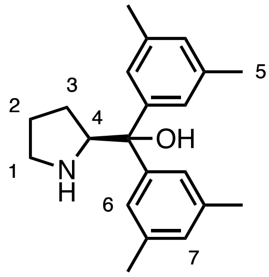

Adapted from the procedure of Matsubara et al.[36] To a solution of (*S*)-1,1-bis(3,5-dimethylphenyl)tetrahydro-1*H*,3*H*-pyrrolo[1,2-*c*]oxazol-3-one **S65** (0.33 g, 0.99 mmol, 1.0 equiv.) in MeOH (4 mL) at room temperature was added potassium hydroxide (0.56 g, 9.9 mmol, 10.0 equiv.). The resulting mixture was heated to 65 °C and left to stir for 19 h. The reaction mixture was then heated to 80 °C for 48 h. The reaction mixture was then cooled to room temperature and concentrated *in vacuo*. The resulting residue was diluted with water (4 mL) and extracted with EtOAc (3 × 3 mL). The combined organic extracts were washed with brine (5 mL), dried (Na_2SO_4), filtered and concentrated *in vacuo*. Purification by column chromatography (SiO_2, pentane/Et_2O, 3:2 → Et_2O) afforded the title compound as a pale yellow oil (0.13 g, 0.42 mmol, 42%).

R*f* 0.14 (pentane/Et_2O, 1:4), [UV, phosphomolybdic acid]
^{1}H NMR (500 MHz, $CDCl_3$) δ 7.17 (s, 2H, H6), 7.10 (s, 2H, H6), 6.79 (s, 2H, H7), 4.21 (t, *J* = 7.7 Hz, 1H, H4), 3.01 (ddd, *J* = 9.3, 6.5, 5.0 Hz, 1H, H1), 2.94 (dt, *J* = 9.3, 7.5 Hz, 1H, H1), 2.28 (s, 6H, H5), 2.27 (s, 6H, H5), 1.76 – 1.68 (m, 2H, H2), 1.63 – 1.56 (m, 3H, H3 and N*H*).
Note: the O*H* resonance was not observed.
^{13}C NMR (126 MHz, $CDCl_3$) δ 148.3, 145.5, 137.7 (2C), 137.4 (2C), 128.3, 128.1, 123.7 (2C), 123.4 (2C), 77.4, 64.7, 46.9, 26.4, 25.7, 21.7 (2C), 21.7 (2C).
Spectroscopic data in agreement with that reported previously.[41]

(*S*)-2-(Bis(3,5-dimethylphenyl)((trimethylsilyl)oxy)methyl)pyrrolidine, S36

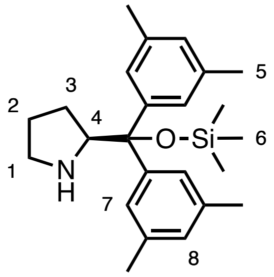

Adapted from the procedure of Pihko et al.[42] To a solution of (*S*)-bis(3,5-dimethylphenyl)(pyrrolidin-2-yl)methanol **S66** (0.13 g, 0.42 mmol, 1.0 equiv.) and Et_3N (0.12 mL, 0.83 mmol, 2.0 equiv.) in CH_2Cl_2 (2 mL) at 0 °C under an inert atmosphere was added trimethylsilyl trifluoromethanesulfonate (0.11 mL, 0.62 mmol, 1.5 equiv.). The reaction mixture was allowed to warm to room temperature and left to stir for 25 h. Water (2 mL) was then added. The phases were separated and the aqueous phase was extracted with CH_2Cl_2 (3 × 2 mL). The combined organic extracts were dried (Na_2SO_4) and concentrated *in vacuo*. Purification by column chromatography (SiO_2, pentane/Et_2O, 2:3) afforded the title compound as a yellow oil (70 mg, 0.18 mmol, 44%).

R*f* 0.19 (pentane/Et_2O, 1:4), [UV, phosphomolybdic acid]

^{1}H NMR (500 MHz, $CDCl_3$) δ 7.03 (s, 2H, H7), 6.95 (s, 2H, H7), 6.85 (s, 1H, H8), 6.83 (s, 1H, H8), 3.96 (t, *J* = 7.3 Hz, 1H, H4), 2.87 – 2.74 (m, 2H, H1), 2.27 (s, 6H, H5), 2.26 (s, 6H, H5), 1.62 – 1.50 (m, 4H, H2, H3 and N*H*), 1.43 – 1.37 (m, 1H, H3), –0.10 (s, 9H, H6).

^{13}C NMR (126 MHz, $CDCl_3$) δ 146.9, 146.0, 136.9 (2C), 136.9 (2C), 128.5, 128.3, 126.1 (2C), 125.5 (2C), 83.4, 65.6, 47.3, 27.7, 25.3, 21.7 (2C), 21.7 (2C), 2.4 (3C).

Spectroscopic data in agreement with that reported previously.[43]

(*S*)-1,1-Di(naphthalen-2-yl)tetrahydro-1*H*,3*H*-pyrrolo[1,2-*c*]oxazol-3-one, S67

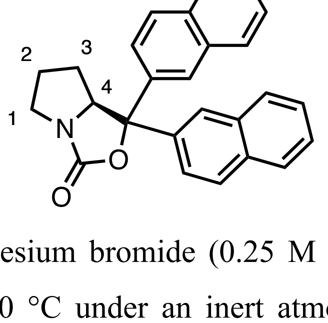

To a solution of 2-naphthylmagnesium bromide (0.25 M in 2-MeTHF, 12 mL, 3.0 mmol, 3.0 equiv.) in THF (2.5 mL) at 0 °C under an inert atmosphere was added a solution of 1-ethyl 2-methyl (*S*)-pyrrolidine-1,2-dicarboxylate **S63** (0.20 g, 1.0 mmol, 1.0 equiv.) in

THF (4.5 mL) dropwise. The reaction mixture was then heated to 65 °C and stirred at this temperature for 24 h. The reaction mixture was then cooled to room temperature and NH_4Cl (aq., sat., 15 mL) was added. The phases were separated and the aqueous phase was extracted with EtOAc (3 × 10 mL). The combined organic extracts were dried (Na_2SO_4) and concentrated *in vacuo*. Purification by column chromatography (SiO_2, pentane/Et_2O, 3:2 and subsequently pentane/CH_2Cl_2, 1:1) afforded the title compound as a white solid (0.14 g, 0.33 mmol, 38%).

$\mathbf{R}_f$ 0.12 (pentane/CH_2Cl_2, 1:1), [UV, phosphomolybdic acid]
^{1}H NMR (400 MHz, $CDCl_3$) δ 8.11 (d, J = 1.8 Hz, 1H, Ar*H*), 8.03 (d, J = 2.0 Hz, 1H, Ar*H*), 7.91 – 7.76 (m, 6H, Ar*H*), 7.64 (dd, J = 8.7, 2.0 Hz, 1H, Ar*H*), 7.54 – 7.47 (m, 4H, Ar*H*), 7.33 (dd, J = 8.6, 1.9 Hz, 1H, Ar*H*), 4.80 (dd, J = 10.6, 5.5 Hz, 1H, H4), 3.79 (dt, J = 11.5, 8.1 Hz, 1H, H1), 3.33 (ddd, J = 11.5, 9.4, 3.7 Hz, 1H, H1), 2.07 – 1.88 (m, 2H, H2), 1.83 – 1.74 (m, 1H, H3), 1.27 – 1.14 (m, 1H, H3).
^{13}C NMR (126 MHz, $CDCl_3$) δ 160.6, 140.3, 137.6, 133.2, 133.0, 132.9, 132.7, 128.8, 128.6, 128.6, 128.3, 127.7, 127.7, 126.9, 126.8, 126.7, 126.7, 125.0, 124.7, 124.4, 123.9, 86.3, 68.9, 46.3, 29.3, 25.2.
Spectroscopic data in agreement with that reported previously.[37]

(*S*)-Di(naphthalen-2-yl)(pyrrolidin-2-yl)methanol, S68

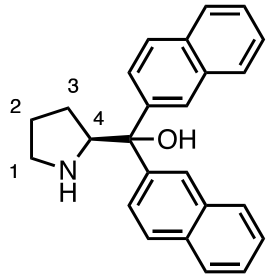

Adapted from the procedure of Matsubara et al.[36] To a solution of (*S*)-1,1-di(naphthalen-2-yl)tetrahydro-1*H*,3*H*-pyrrolo[1,2-*c*]oxazol-3-one **S67** (0.14 g, 0.33 mmol, 1.0 equiv.) in MeOH (1.3 mL) at room temperature was added potassium hydroxide (0.19 g, 3.3 mmol, 10.0 equiv.). The resulting mixture was heated to 100 °C and left to stir for 47 h. The reaction mixture was then cooled to room temperature and concentrated *in vacuo*. The resulting residue was diluted with water (2 mL) and extracted with EtOAc (3 × 2 mL). The combined organic extracts were washed with brine (5 mL), dried (Na_2SO_4), filtered and concentrated *in*

vacuo. Purification by column chromatography (SiO_2, pentane/Et_2O, 2:3) afforded the title compound as a white solid (77 mg, 0.22 mmol, 66%).

R*f* 0.10 (Et_2O), [UV, phosphomolybdic acid]

¹H NMR (400 MHz, $CDCl_3$) δ 8.13 – 8.07 (m, 2H, Ar*H*), 7.85 (t, *J* = 8.1 Hz, 2H, Ar*H*), 7.79 – 7.56 (m, 5H, Ar*H*), 7.55 – 7.37 (m, 5H, Ar*H*), 4.52 (t, *J* = 7.3 Hz, 1H, H4), 3.13 – 2.96 (m, 2H, H1), 1.84 – 1.58 (m, 5H, H2, H3 and N*H*).

Note: the O*H* resonance was not observed.

¹³C NMR (126 MHz, $CDCl_3$) δ 145.4, 142.7, 133.3, 133.2, 132.4, 132.4, 128.4, 128.3, 128.2, 127.8, 127.6, 126.3, 126.2, 126.0, 125.9, 125.8, 125.4, 124.5, 124.2, 123.9, 77.7, 64.2, 47.0, 26.6, 25.7.

Spectroscopic data in agreement with that reported previously.[44]

(*S*)-2-(Di(naphthalen-2-yl)((trimethylsilyl)oxy)methyl)pyrrolidine, S37

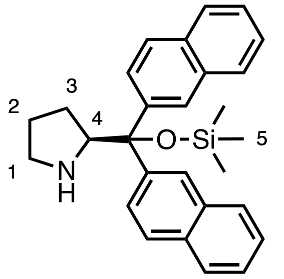

According to the procedure of Pihko et al.[45] To a solution of (*S*)-di(naphthalen-2-yl)(pyrrolidin-2-yl)methanol **S68** (77 mg, 0.22 mmol, 1.0 equiv.) and Et_3N (61 μL, 0.44 mmol, 2.0 equiv.) in CH_2Cl_2 (1.8 mL) at room temperature was added trimethylsilyl chloride (33 μL, 0.26 mmol, 1.2 equiv.) and DMAP (5.3 mg, 0.044 mmol, 0.2 equiv.). The reaction mixture was left to stir at room temperature for 22 h. Upon completion, the reaction mixture was concentrated *in vacuo*. Purification by column chromatography (SiO_2, pentane/Et_2O, 1:4) afforded the title compound as a pale yellow oil (85 mg, 0.20 mmol, 92%).

R*f* 0.29 (Et_2O), [UV, vanillin/phosphomolybdic acid]

¹H NMR (500 MHz, $CDCl_3$) δ 8.09 (s, 1H, Ar*H*), 8.03 (s, 1H, Ar*H*), 7.89 – 7.76 (m, 4H, Ar*H*), 7.68 (t, *J* = 8.5 Hz, 2H, Ar*H*), 7.51 – 7.43 (m, 5H, Ar*H*), 7.31 (dd, *J* = 8.7, 1.8 Hz, 1H, Ar*H*), 4.28 (t, *J* = 7.4 Hz, 1H, H4), 2.97 – 2.88 (m, 1H, H1), 2.87 – 2.79 (m, 1H, H1), 1.78 – 1.52 (m, 4H, H2, H3 and N*H*), 1.47 – 1.38 (m, 1H, H2), –0.05 (s, 9H, H5).

^{13}**C NMR** (126 MHz, $CDCl_3$) δ 144.2, 143.3, 133.0, 132.9, 132.6, 132.5, 128.6 (2C), 127.6 (2C), 127.6, 127.6, 127.4, 127.3, 126.7, 126.6, 126.0 (2C), 126.0, 125.7, 83.7, 65.5, 47.4, 27.8, 25.3, 2.5 (3C).
Spectroscopic data in agreement with that reported previously.[45]

***tert*-Butyl (*S*)-2-(bis(3,5-bis(trifluoromethyl)phenyl)(hydroxy)methyl)-4,4-difluoropyrrolidine-1-carboxylate, S70**

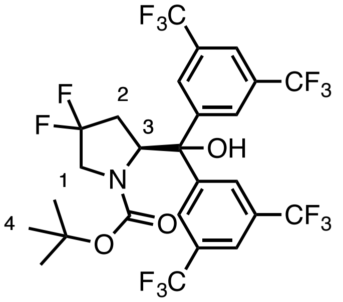

According to the procedure of Burnett et al.[46] To a solution of *N*-Boc-4,4-difluoro-L-proline (0.50 g, 2.0 mmol, 1.0 equiv.) in acetone (5 mL) at room temperature under an inert atmosphere was added potassium carbonate (0.42 g, 3.0 mmol, 1.5 equiv.) and iodo-methane (0.19 mL, 3.0 mmol, 1.5 equiv.). The reaction mixture was stirred for 20 h at room temperature. The reaction mixture was then filtered and washed with EtOAc. The filtrate was concentrated *in vacuo* to afford 1-(*tert*-butyl) 2-methyl (*S*)-4,4-difluoropyrrolidine-1,2-dicarboxylate **S69** as a yellow oil (0.46 g, 1.7 mmol, 86%).
Adapted from the procedure of Matsubara et al.[36] To a solution of 1,3-bis(trifluoromethyl)-5-bromobenzene (0.89 mL, 5.2 mmol, 3.0 equiv.) in THF (2.5 mL) at 0 °C under an inert atmosphere was added isopropylmagnesium chloride (1.5 M in THF, 3.6 mL, 5.4 mmol, 3.15 equiv.) dropwise. The resulting mixture was stirred for 1 h at 0 °C. A solution of 1-(*tert*-butyl) 2-methyl (*S*)-4,4-difluoropyrrolidine-1,2-dicarboxylate **S69** (0.46 g, 1.7 mmol, 1.0 equiv.) in THF (4.5 mL) was then added dropwise at 0 °C. The reaction mixture was then allowed to warm to room temperature and stirred at this temperature for 17 h. NH_4Cl (aq., sat., 7 mL) was then added to the reaction mixture. The phases were separated and the aqueous phase was extracted with EtOAc (3 × 5 mL). The combined organic extracts were dried (Na_2SO_4) and concentrated *in vacuo*. Purification by column chromatography (SiO_2, pentane/Et_2O, 9:1) afforded the title compound as a dark yellow oil (0.57 g, 0.86 mmol, 50%).

R*f* 0.71 (pentane/Et_2O, 4:1), [UV, vanillin]

^{1}H NMR (400 MHz, $CDCl_3$) δ 7.93 (s, 3H, Ar*H*), 7.85 (s, 1H, Ar*H*), 7.81 (s, 2H, Ar*H*), 5.94 (br s, 1H, O*H*), 5.16 (dd, *J* = 9.4, 7.0 Hz, 1H, H3), 4.03 – 3.90 (m, 1H, H1), 3.14 (ddd, *J* = 23.0, 13.0, 7.5 Hz, 1H, H1), 2.64 – 2.46 (m, 1H, H2), 2.31 – 2.13 (m, 1H, H2), 1.33 (s, 9H, H4).

^{13}C NMR (126 MHz, $CDCl_3$) δ 156.2, 145.3, 144.3, 132.2 (q, *J* = 33.6 Hz, 2C), 132.1 (d, *J* = 33.9 Hz, 2C), 127.4 (d, *J* = 4.0 Hz, 4C), 125.9 (t, *J* = 247.6 Hz), 123.2 (q, *J* = 273.0 Hz, 4C), 122.5 (dt, *J* = 6.9, 3.4 Hz, 2C), 83.2, 81.0, 64.6 (d, *J* = 4.4 Hz), 55.0 (dd, *J* = 33.4, 30.9 Hz), 38.7 (dd, *J* = 27.1, 23.8 Hz), 28.0 (3C).

^{19}F NMR (471 MHz, $CDCl_3$) δ –62.9, –62.9, –93.8 – –94.4 (m), –102.3 – –103.6 (m).

HRMS (ESI$^-$) Found $[M–H]^-$ = 660.1224; $C_{26}H_{20}O_3NF_{14}$ requires 660.1225.

IR (film) ν_{max}/cm^{-1} 3021, 2923, 2845, 1673, 1415, 1370, 1279, 1176, 1136.

$[\alpha]_D^{25}$ – 32.1 (*c* = 0.3, $CHCl_3$)

(*S*)-Bis(3,5-bis(trifluoromethyl)phenyl)(4,4-difluoropyrrolidin-2-yl)methanol, S71

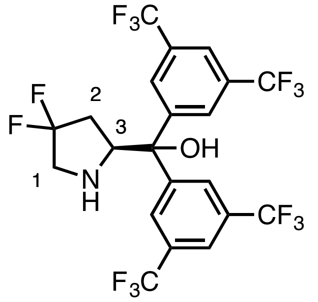

Adapted from the procedure of Corey et al.[47] To a solution of *tert*-butyl (*S*)-2-(bis(3,5-bis(trifluoromethyl)phenyl)(hydroxy)methyl)-4,4-difluoropyrrolidine-1-carboxylate **S70** (0.57 g, 0.86 mmol, 1.0 equiv.) in ethanol (5.0 mL) at room temperature was added sodium hydroxide (0.17 g, 4.3 mmol, 5.0 equiv.). The reaction mixture was heated to reflux (100 °C) for 40.5 h and then concentrated *in vacuo*. The reaction mixture was diluted with water (2.0 mL), neutralized with HCl (aq., 1 M) and the aqueous phase was extracted with Et_2O (3 × 2 mL). The combined organic extracts were washed with brine (5 mL), dried (Na_2SO_4), filtered and concentrated *in vacuo*. Purification by column chromatography (SiO_2, pentane/Et_2O, 9:1 and subsequently pentane/CH_2Cl_2, 4:1) afforded the title compound as an orange oil (0.15 g, 0.27 mmol, 32%).

R$_f$ 0.24 (pentane/CH_2Cl_2, 4:1), [UV, vanillin]

^{1}**H NMR** (400 MHz, $CDCl_3$) δ 8.05 (s, 2H, Ar*H*), 7.94 (s, 2H, Ar*H*), 7.81 (d, *J* = 10.4 Hz, 2H, Ar*H*), 4.67 (s, 1H, O*H*), 4.64 (dd, *J* = 9.5, 7.1 Hz, 1H, H3), 3.50 – 3.32 (m, 2H, H1), 2.26 – 2.07 (m, 1H, H2), 2.03 – 1.89 (m, 1H, H2), 1.77 (br s, 1H, N*H*).

^{13}**C NMR** (126 MHz, $CDCl_3$) δ 147.6, 145.1, 132.7 (q, *J* = 33.4 Hz, 2C), 132.4 (q, *J* = 33.6 Hz, 2C), 128.4 (t, *J* = 254.3 Hz), 126.0 (d, *J* = 3.9 Hz, 2C), 125.4 (d, *J* = 4.0 Hz, 2C), 123.2 (q, *J* = 273.0 Hz, 4C), 122.3 (quin, *J* = 3.8 Hz), 122.1 (quin, *J* = 4.0 Hz), 76.2, 62.6 (dd, *J* = 5.3, 2.1 Hz), 54.0 (t, *J* = 29.5 Hz), 35.7 (t, *J* = 24.8 Hz).

^{19}**F NMR** (471 MHz, $CDCl_3$) δ –62.8, –62.9, –95.8 – –96.8 (m), –99.5 – –100.8 (m).

HRMS (ESI$^+$) Found $[M+H]^+$ = 562.0844; $C_{21}H_{14}ONF_{14}$ requires 562.0846.

IR (film) ν_{max}/cm^{-1} 3016, 2926, 1371, 1279, 1165, 1132.

$[\boldsymbol{\alpha}]_{\mathbf{D}}^{\mathbf{25}}$ – 32.9 (*c* = 0.2, $CHCl_3$)

(*S*)-2-(Bis(3,5-bis(trifluoromethyl)phenyl)((trimethylsilyl)oxy)methyl)-4,4-difluoropyrrolidine, S39

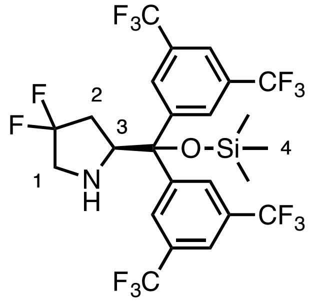

According to the procedure of Pihko et al.[45] To a solution of (*S*)-bis(3,5-bis(trifluoromethyl)phenyl)(4,4-difluoropyrrolidin-2-yl)methanol **S71** (0.15 g, 0.27 mmol, 1.0 equiv.) and Et_3N (77 μL, 0.55 mmol, 2.0 equiv.) in CH_2Cl_2 (2.2 mL) at room temperature was added trimethylsilyl chloride (42 μL, 0.33 mmol, 1.2 equiv.) and DMAP (6.7 mg, 0.055 mmol, 0.2 equiv.). The reaction mixture was left to stir at room temperature for 17.5 h. Upon completion, the reaction mixture was concentrated *in vacuo*. Purification by column chromatography (SiO_2, pentane/Et_2O, 99:1) afforded the title compound as a yellow oil (73 mg, 0.12 mmol, 42%).

$\mathbf{R}_f$ 0.60 (pentane/Et_2O, 9:1), [UV, vanillin]

^{1}**H NMR** (400 MHz, $CDCl_3$) δ 8.00 (s, 2H, Ar*H*), 7.89 (d, *J* = 8.1 Hz, 2H, Ar*H*), 7.72 (s, 2H, Ar*H*), 4.40 (td, *J* = 9.2, 7.4 Hz, 1H, H3), 3.27 – 3.14 (m, 1H, H1), 2.90 – 2.75 (m, 1H, H1), 2.15 – 1.99 (m, 2H, H2 and N*H*), 1.98 – 1.81 (m, 1H, H2), –0.06 (s, 9H, H4).

^{13}**C NMR** (126 MHz, $CDCl_3$) δ 147.0, 145.3, 132.2 (q, *J* = 33.6 Hz, 2C), 131.7 (q, *J* = 33.5 Hz, 2C), 129.6 (t, *J* = 253.8 Hz), 128.7 (d, *J* = 3.8 Hz, 2C), 127.6 (d, *J* = 3.7 Hz, 2C), 123.3 (q, *J* = 272.9, 270.9 Hz, 2C), 123.1 (q, *J* = 272.9 Hz, 2C), 122.4 (q, *J* = 3.8 Hz, 2C), 81.6, 63.1 (d, *J* = 6.5 Hz), 54.2 (t, *J* = 28.9 Hz), 37.4 (t, *J* = 25.0 Hz), 2.0 (3C).
^{19}**F NMR** (471 MHz, $CDCl_3$) δ –62.9, –62.9, –96.9, –97.4.
HRMS (ESI^+) Found $[M+H]^+$ = 634.1239; $C_{24}H_{22}ONF_{14}Si$ requires 634.1242.
IR (film) ν_{max}/cm^{-1} 1371, 1279, 1133, 913, 843.
$[\alpha]_D^{25}$ – 26.6 (*c* = 0.2, $CHCl_3$)

(6*R*,7a*S*)-1,1-Bis(3,5-bis(trifluoromethyl)phenyl)-6-hydroxytetrahydro-1*H*,3*H*-pyrrolo[1,2-*c*]oxazol-3-one, S73

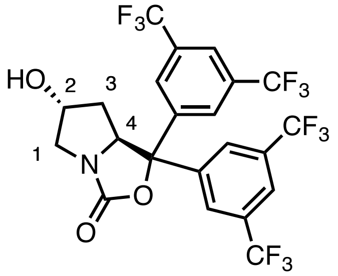

According to the procedure of Vicario et al.[48] To a solution of *trans*-4-hydroxy-L-proline (1.31 g, 10.0 mmol, 1.0 equiv.) and potassium carbonate (1.38 g, 10.0 mmol, 1.0 equiv.) in MeOH (5 mL) at 0 °C under an inert atmosphere was added methyl chloroformate (1.62 mL, 21.0 mmol, 2.1 equiv.) dropwise. The reaction mixture was allowed to warm to room temperature and left to stir for 16.5 h. The reaction mixture was then concentrated *in vacuo*. The resulting residue was dissolved in CH_2Cl_2 (5 mL) and water (5 mL). The phases were separated and the aqueous phase was extracted with CH_2Cl_2 (3 × 5 mL). The combined organic extracts were dried (Na_2SO_4), filtered and concentrated *in vacuo* to afford dimethyl (2*S*,4*R*)-4-hydroxypyrrolidine-1,2-dicarboxylate **S72** as a pale yellow oil (0.95 g, 4.7 mmol, 47%).
Adapted from the procedure of Matsubara et al.[36] To a solution of isopropylmagnesium chloride (1.5 M in THF, 9.8 mL, 15 mmol, 3.15 equiv.) at 0 °C under an inert atmosphere was added 1,3-bis(trifluoromethyl)-5-bromobenzene (2.4 mL, 14 mmol, 3.0 equiv.) in THF (10 mL) dropwise. The resulting mixture was stirred for 1 h at 0 °C. A solution of dimethyl (2*S*,4*R*)-4-hydroxypyrrolidine-1,2-dicarboxylate **S72** (0.95 g, 4.7 mmol, 1.0 equiv.) in THF (18 mL) was then added dropwise at 0 °C. The reaction mixture was then heated to 65 °C and stirred at this temperature for 18.5 h. The reaction mixture was then cooled to

room temperature and NH_4Cl (aq., sat., 15 mL) was added. The phases were separated and the aqueous phase was extracted with EtOAc (3 × 10 mL). The combined organic extracts were dried (Na_2SO_4) and concentrated *in vacuo*. Purification by column chromatography (SiO_2, pentane/Et_2O, 1:1) afforded the title compound as a yellow oil (1.0 g, 1.8 mmol, 39%).

R*f* 0.24 (Et_2O), [UV, vanillin]

^{1}H NMR (400 MHz, $CDCl_3$) δ 7.99 (s, 2H, Ar*H*), 7.93 (s, 1H, Ar*H*), 7.90 (s, 1H, Ar*H*), 7.86 (s, 2H, Ar*H*), 4.94 (dd, *J* = 11.4, 4.8 Hz, 1H, H4), 4.64 (q, *J* = 4.9 Hz, 1H, H2), 4.12 (dd, *J* = 12.9, 5.6 Hz, 1H, H1), 3.27 (d, *J* = 12.9 Hz, 1H, H1), 1.88 (d, *J* = 3.2 Hz, 1H, O*H*), 1.80 (dd, *J* = 13.1, 4.9 Hz, 1H, H3), 1.23 – 1.17 (m, 1H, H3).

^{13}C NMR (126 MHz, $CDCl_3$) δ 158.8, 144.4, 141.5, 133.0 (q, *J* = 33.9 Hz, 2C), 132.9 (q, *J* = 34.0 Hz, 2C), 126.0 (d, *J* = 3.8 Hz, 2C), 125.5 (d, *J* = 3.8 Hz, 2C), 123.6 (quin, *J* = 3.7 Hz), 123.0 (quin, *J* = 3.6 Hz), 122.9 (q, *J* = 273.2 Hz, 2C), 122.8 (q, *J* = 273.1 Hz, 2C), 83.6, 71.2, 67.2, 56.9, 39.4.

Spectroscopic data in agreement with that reported previously.[48]

(2*S*,4*R*)-2-(Bis(3,5-bis(trifluoromethyl)phenyl)((trimethylsilyl)oxy)methyl)-4-((trimethylsilyl)oxy)pyrrolidine, S40

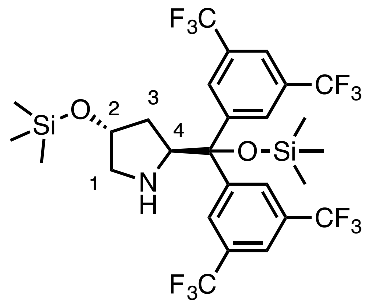

According to the procedure of Cozzi et al.[49] To a solution of (6*R*,7a*S*)-1,1-bis(3,5-bis(trifluoromethyl)phenyl)-6-hydroxytetrahydro-1*H*,3*H*-pyrrolo[1,2-*c*]oxazol-3-one **S73** (0.28 g, 0.5 mmol, 1.0 equiv.) in ethanol (1.4 mL) at room temperature was added potassium hydroxide (0.15 g, 2.6 mmol, 5.2 equiv.) and water (0.33 mL). The reaction mixture was heated to 80 °C for 17 h before being cooled to room temperature. The reaction mixture was then concentrated *in vacuo* and diluted with water (2 mL). The aqueous phase was extracted with EtOAc (3 × 2 mL). The combined organic extracts were dried (Na_2SO_4), filtered and concentrated *in vacuo* to afford (3*R*,5*S*)-5-(bis(3,5-

bis(trifluoromethyl)phenyl)(hydroxy)methyl)pyrrolidin-3-ol **S74** as an orange oil (0.20 g, 0.37 mmol, 75%).

According to the procedure of Pihko et al.[45] To a solution of (3*R*,5*S*)-5-(bis(3,5-bis(trifluoromethyl)phenyl)(hydroxy)methyl)pyrrolidin-3-ol **S74** (0.20 g, 0.37 mmol, 1.0 equiv.) and Et_3N (0.31 mL, 2.2 mmol, 6.0 equiv.) in CH_2Cl_2 (4 mL) at room temperature was added trimethylsilyl chloride (0.17 mL, 1.3 mmol, 3.5 equiv.) and DMAP (11 mg, 0.093 mmol, 0.25 equiv.). The reaction mixture was left to stir at room temperature for 22 h. Upon completion, the reaction mixture was diluted with water (4 mL). The aqueous phase was extracted with CH_2Cl_2 (3 × 3 mL). The combined organic extracts were dried (Na_2SO_4), filtered and concentrated *in vacuo*. Purification by column chromatography (SiO_2, pentane/CH_2Cl_2, 19:1 → pentane/Et_2O, 9:1) afforded the title compound as a yellow oil (0.11 g, 0.16 mmol, 43%).

R*f* 0.67 (pentane/CH_2Cl_2, 4:1), [UV, vanillin]

^{1}H NMR (400 MHz, $CDCl_3$) δ 8.01 (s, 2H, Ar*H*), 7.84 (d, *J* = 4.9 Hz, 2H, Ar*H*), 7.77 (s, 2H, Ar*H*), 4.49 (t, *J* = 7.9 Hz, 1H, H4), 3.86 (br s, 1H, H2), 2.74 (d, *J* = 11.6 Hz, 1H, H1), 2.22 (dd, *J* = 11.6, 3.8 Hz, 1H, H1), 2.14 (br s, 1H, N*H*), 1.73 (dd, *J* = 13.5, 7.6 Hz, 1H, H3), 1.43 (ddd, *J* = 13.5, 8.2, 5.1 Hz, 1H, H3), 0.06 (s, 9H, Si(CH_3)$_3$), –0.10 (s, 9H, Si(CH_3)$_3$).

^{13}C NMR (126 MHz, $CDCl_3$) δ 148.2, 146.2, 131.7 (q, *J* = 33.3 Hz, 2C), 130.9 (q, *J* = 33.2 Hz, 2C), 128.8 (d, *J* = 4.0 Hz, 2C), 128.5 (d, *J* = 3.9 Hz, 2C), 123.5 (q, *J* = 272.6 Hz, 2C), 123.3 (q, *J* = 272.9 Hz, 2C), 121.9 (quin, *J* = 3.9 Hz), 121.6 (quin, *J* = 3.4 Hz), 82.3, 72.4, 62.9, 56.0, 37.8, 2.0 (3C), 0.1 (3C).

^{19}F NMR (471 MHz, $CDCl_3$) δ –62.8, –62.8.

HRMS (ESI^+) Found $[M+H]^+$ = 686.1771; $C_{27}H_{32}O_2NF_{12}Si_2$ requires 686.1775.

IR (film) ν_{max}/cm^{-1} 1374, 1279, 1255, 1174, 1134, 906, 877, 842.

$[\boldsymbol{\alpha}]_{\mathbf{D}}^{\mathbf{25}}$ – 23.1 (*c* = 0.3, $CHCl_3$)

3.2.5 Aldehyde substrates

Benzyl (3-oxopropyl)carbamate, 8

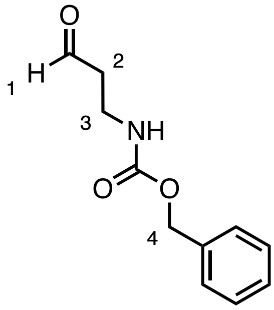

According to the procedure of MacMillan et al.[50] To a solution of 3,3-diethoxypropan-1-amine (6.47 mL, 40.0 mmol, 1.0 equiv.) in EtOAc (80 mL) at room temperature was added $NaHCO_3$ (16.8 g, 200 mmol, 5.0 equiv.) and water (80 mL). Benzyl chloroformate (8.46 mL, 60.0 mmol, 1.5 equiv.) was then added and the reaction mixture was stirred at room temperature for 15 h. The phases were then separated and the aqueous phase was extracted with EtOAc (3 × 50 mL). The combined organic extracts were dried (Na_2SO_4), filtered and concentrated *in vacuo*.

The residue was dissolved in THF (14 mL) and cooled to 0 °C. HCl (aq., 1 M, 7 mL) was then added. The reaction mixture was allowed to warm to room temperature and stirred for 6 h. The reaction mixture was then diluted with Et_2O (50 mL) and $NaHCO_3$ (aq., sat., 50 mL). The phases were separated and the aqueous phase was extracted with Et_2O (3 × 30 mL). The combined organic extracts were dried (Na_2SO_4), filtered and concentrated *in vacuo*. Purification by column chromatography (SiO_2, pentane/Et_2O, 2:3) afforded the title compound as a white solid (3.55 g, 17.2 mmol, 43%).

R*f* 0.40 (Et_2O), [UV, vanillin]

^{1}H NMR (400 MHz, $CDCl_3$) δ 9.81 (s, 1H, H1), 7.39 – 7.29 (m, 5H, Ar*H*), 5.14 (br s, 1H, N*H*), 5.08 (s, 2H, H4), 3.49 (app. q, *J* = 5.9 Hz, 2H, H3), 2.75 (t, *J* = 5.9 Hz, 2H, H2).

^{13}C NMR (101 MHz, $CDCl_3$) δ 201.3, 156.4, 136.5, 128.7 (2C), 128.3, 128.2 (2C), 66.9, 44.2, 34.6.

Spectroscopic data in agreement with that reported previously.[50]

3-(4-Methoxyphenyl)propanal, S76

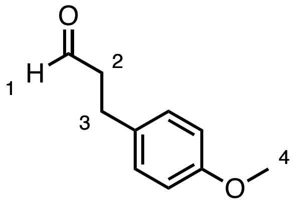

3-(4-Methoxyphenyl)propionic acid (0.90 g, 5.0 mmol, 1.0 equiv.) and borane dimethyl sulfide complex (2 M in THF, 2.8 mL, 5.5 mmol, 1.1 equiv.) in THF (25 mL) were submitted to General Procedure E for 17 h to afford 3-(4-methoxyphenyl)propan-1-ol **S75** as a clear oil (0.78 g, 4.7 mmol, 94%).

3-(4-Methoxyphenyl)propan-1-ol **S75** (0.78 g, 4.7 mmol, 1.0 equiv.), TEMPO (0.15 g, 0.94 mmol, 0.2 equiv.) and BAIB (2.7 g, 8.5 mmol, 1.8 equiv.) in CH_2Cl_2 (25 mL) were submitted to General Procedure F for 14 h. Purification by column chromatography (SiO_2, pentane/Et_2O, 17:3) afforded the title compound as an orange oil (0.57 g, 3.5 mmol, 73%).

R*f* 0.29 (pentane/Et_2O, 4:1), [UV, vanillin]

^{1}H NMR (500 MHz, $CDCl_3$) δ 9.82 (t, *J* = 1.5 Hz, 1H, H1), 7.11 (d, *J* = 8.7 Hz, 2H, Ar*H*), 6.84 (d, *J* = 8.7 Hz, 2H, Ar*H*), 3.79 (s, 3H, H4), 2.91 (t, *J* = 7.6 Hz, 2H, H3), 2.75 (td, *J* = 7.6, 1.5 Hz, 2H, H2).

^{13}C NMR (126 MHz, $CDCl_3$) δ 201.9, 158.2, 132.4, 129.3 (2C), 114.1 (2C), 55.4, 45.7, 27.4.

Spectroscopic data in agreement with that reported previously.[51]

3-(4-(Trifluoromethyl)phenyl)propanal, S78

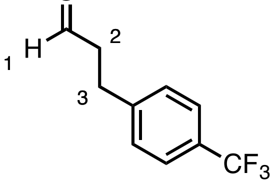

4-(Trifluoromethyl)hydrocinnamic acid (0.55 g, 2.5 mmol, 1.0 equiv.) and borane dimethyl sulfide complex (2 M in THF, 1.4 mL, 2.8 mmol, 1.1 equiv.) in THF (15 mL) were submitted to General Procedure E for 20 h to afford 3-(4-(trifluoromethyl)phenyl)propan-1-ol **S77** as a clear oil (0.49 g, 2.4 mmol, 96%).

3-(4-(Trifluoromethyl)phenyl)propan-1-ol **S77** (0.49 g, 2.4 mmol, 1.0 equiv.), TEMPO (75 mg, 0.48 mmol, 0.2 equiv.) and BAIB (1.4 g, 4.3 mmol, 1.8 equiv.) in CH_2Cl_2 (12 mL) were submitted to General Procedure F for 18 h. Purification by column

chromatography (SiO_2, pentane/Et_2O, 4:1) afforded the title compound as a pale yellow oil (0.26 g, 1.3 mmol, 54%).

R*f* 0.52 (pentane/Et_2O, 1:1), [UV, vanillin]
^{1}H NMR (500 MHz, $CDCl_3$) δ 9.83 (t, *J* = 1.1 Hz, 1H, H1), 7.55 (d, *J* = 8.0 Hz, 2H, Ar*H*), 7.31 (d, *J* = 7.9 Hz, 2H, Ar*H*), 3.01 (t, *J* = 7.5 Hz, 2H, H3), 2.82 (td, *J* = 7.5, 1.1 Hz, 2H, H2).
^{13}C NMR (126 MHz, $CDCl_3$) δ 200.8, 144.6, 128.9 (q, *J* = 32.4 Hz), 128.8 (2C), 125.7 (q, *J* = 3.8 Hz, 2C), 124.3 (q, *J* = 271.9 Hz), 45.0, 28.0.
Spectroscopic data in agreement with that reported previously.[52]

5-Phenylpent-4-yn-1-ol, S79

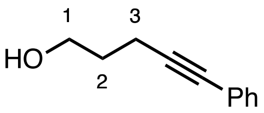

According to the procedure of Ferreira et al.[53] To a solution of iodobenzene (0.56 mL, 5.0 mmol, 1.0 equiv.) in Et_3N (15 mL) at room temperature under an inert atmosphere was added 4-pentyn-1-ol (0.51 mL, 5.5 mmol, 1.1 equiv.) and then degassed by sparging with argon for 15 min. Copper(I) iodide (38 mg, 0.20 mmol, 0.04 equiv.) and $PdCl_2(PPh_3)_2$ (70 mg, 0.10 mmol, 0.02 equiv.) were then added and the reaction mixture was left to stir at room temperature for 24.5 h. Upon completion, the reaction mixture was filtered through a short pad of silica (eluting with EtOAc). The filtrate was then concentrated *in vacuo*. Purification by column chromatography (SiO_2, pentane/Et_2O, 3:2) afforded the title compound as a dark orange oil (0.80 g, 5.0 mmol, 99%).

R*f* 0.26 (pentane/Et_2O, 1:1), [UV, vanillin]
^{1}H NMR (400 MHz, $CDCl_3$) δ 7.41 – 7.36 (m, 2H, Ar*H*), 7.30 – 7.26 (m, 3H, Ar*H*), 3.83 (td, *J* = 6.1, 5.5 Hz, 2H, H1), 2.55 (t, *J* = 6.9 Hz, 2H, H3), 1.87 (tt, *J* = 6.9, 6.1 Hz, 2H, H2), 1.48 (t, *J* = 5.5 Hz, 1H, O*H*).
^{13}C NMR (126 MHz, $CDCl_3$) δ 131.7 (2C), 128.4 (2C), 127.8, 123.8, 89.4, 81.3, 62.0, 31.5, 16.2.
Spectroscopic data in agreement with that reported previously.[54]

5-Phenylpent-4-ynal, S80

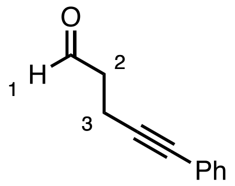

5-Phenylpent-4-yn-1-ol **S79** (0.80 g, 5.0 mmol, 1.0 equiv.), TEMPO (0.16 g, 0.99 mmol, 0.2 equiv.) and BAIB (2.9 g, 8.9 mmol, 1.8 equiv.) in CH_2Cl_2 (25 mL) were submitted to General Procedure F for 21 h. Purification by column chromatography (SiO_2, pentane/Et_2O, 17:3) afforded the title compound as an orange oil (0.30 g, 1.9 mmol, 39%).

R*f* 0.55 (pentane/Et_2O, 1:1), [UV, vanillin]

^{1}H NMR (500 MHz, $CDCl_3$) δ 9.86 (s, 1H, H1), 7.40 – 7.36 (m, 2H, Ar*H*), 7.30 – 7.27 (m, 3H, Ar*H*), 2.80 – 2.72 (m, 4H, H2 and H3).

^{13}C NMR (126 MHz, $CDCl_3$) δ 200.5, 131.6 (2C), 128.3 (2C), 127.9, 123.4, 87.7, 81.5, 42.7, 12.8.

Spectroscopic data in agreement with that reported previously.[55]

6-Chlorohexanal, S81

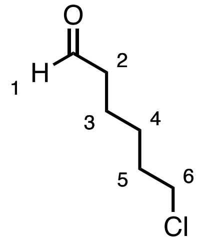

6-Chlorohexanol (0.67 mL, 5.0 mmol, 1.0 equiv.), TEMPO (0.16 g, 1.0 mmol, 0.2 equiv.) and BAIB (2.9 g, 9.0 mmol, 1.8 equiv.) in CH_2Cl_2 (25 mL) were submitted to General Procedure F for 18 h. Purification by column chromatography (SiO_2, pentane/Et_2O, 17:3) afforded the title compound as an orange oil (0.58 g, 4.3 mmol, 86%).

R*f* 0.33 (pentane/Et_2O, 4:1), [vanillin]

^{1}H NMR (500 MHz, $CDCl_3$) δ 9.78 (t, *J* = 1.6 Hz, 1H, H1), 3.55 (t, *J* = 6.6 Hz, 2H, H6), 2.47 (td, *J* = 7.3, 1.6 Hz, 2H, H2), 1.85 – 1.76 (m, 2H, H5), 1.72 – 1.62 (m, 2H, H3), 1.55 – 1.44 (m, 2H, H4).

^{13}C NMR (126 MHz, $CDCl_3$) δ 202.3, 44.8, 43.7, 32.3, 26.4, 21.4.

Spectroscopic data in agreement with that reported previously.[56]

Ethyl 6-oxohexanoate, S82

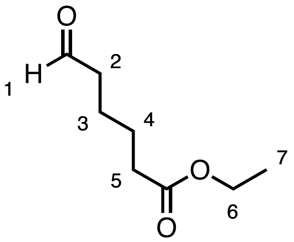

Ethyl 6-hydroxyhexanoate (0.81 mL, 5.0 mmol, 1.0 equiv.), TEMPO (0.16 g, 1.0 mmol, 0.2 equiv.) and BAIB (2.9 g, 9.0 mmol, 1.8 equiv.) in CH_2Cl_2 (25 mL) were submitted to General Procedure F for 18 h. Purification by column chromatography (SiO_2, pentane/Et_2O, 4:1) afforded the title compound as a light orange oil (0.73 g, 4.6 mmol, 93%).

R_f 0.17 (pentane/Et_2O, 4:1), [vanillin]
^{1}H NMR (500 MHz, $CDCl_3$) δ 9.77 (t, J = 1.6 Hz, 1H, H1), 4.13 (q, J = 7.1 Hz, 2H, H6), 2.50 – 2.43 (m, 2H, H2), 2.36 – 2.29 (m, 2H, H5), 1.69 – 1.64 (m, 4H, H3 and H4), 1.25 (t, J = 7.1 Hz, 3H, H7).
^{13}C NMR (126 MHz, $CDCl_3$) δ 202.2, 173.4, 60.5, 43.7, 34.1, 24.5, 21.6, 14.4.
Spectroscopic data in agreement with that reported previously.[57]

5-(Benzyloxy)pentanal, S84

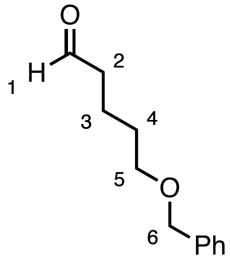

According to the procedure of Zhu et al.[58] To a solution of sodium hydride (60% dispersion in mineral oil, 0.66 g, 17 mmol, 3.3 equiv.) in THF (7.5 mL) at 0 °C under an inert atmosphere was added a solution of 1,5-pentanediol (3.7 mL, 35 mmol, 7.0 equiv.) in THF (15 mL) dropwise. The reaction mixture was left to stir at 0 °C for 30 min. A solution of benzyl bromide (0.59 mL, 5.0 mmol, 1.0 equiv.) in THF (7.5 mL) was then added dropwise. The reaction mixture was allowed to warm to room temperature and was left to stir for 21 h. Water (5 mL) was then added and the reaction mixture was concentrated *in vacuo*. The residue was taken up in water (5 mL) and EtOAc (5 mL). The phases were separated and the aqueous phase was extracted with EtOAc (3 × 5 mL). The combined organic extracts were washed with water (10 mL) and brine (10 mL), dried (Na_2SO_4) and concentrated *in vacuo* to

afford 5-(benzyloxy)pentan-1-ol **S83** as a pale yellow oil, which was used without further purification.

The crude residue containing 5-(benzyloxy)pentan-1-ol **S83** obtained directly from the above procedure, TEMPO (0.16 g, 1.0 mmol, 0.2 equiv.) and BAIB (2.9 g, 9.0 mmol, 1.8 equiv.) in CH_2Cl_2 (25 mL) were submitted to General Procedure F for 24 h. Purification by column chromatography (SiO_2, pentane/Et_2O, 9:1) afforded the title compound as a pale orange oil (0.16 g, 0.83 mmol, 17%).

R*f* 0.52 (pentane/Et_2O, 1:1), [UV, vanillin]

^{1}H NMR (400 MHz, $CDCl_3$) δ 9.76 (t, *J* = 1.7 Hz, 1H, H1), 7.38 – 7.27 (m, 5H, Ar*H*), 4.50 (s, 2H, H6), 3.49 (t, *J* = 6.1 Hz, 2H, H5), 2.46 (td, *J* = 7.2, 1.7 Hz, 2H, H2), 1.78 – 1.63 (m, 4H, H3 and H4).

^{13}C NMR (126 MHz, $CDCl_3$) δ 202.6, 138.6, 128.5 (2C), 127.8 (2C), 127.7, 73.1, 69.9, 43.7, 29.3, 19.1.

Spectroscopic data in agreement with that reported previously.[59]

3-(Thiophen-2-yl)propanal, S85

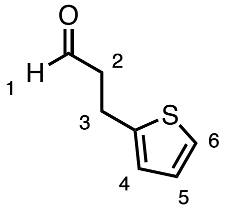

According to the procedure of Su et al.[60] To a flame-dried vial containing 2-iodo-thiophene (0.33 mL, 3.0 mmol, 1.0 equiv.) at room temperature was added $Pd(OAc)_2$ (6.7 mg, 0.030 mmol, 0.01 equiv.), benzyltriethylammonium chloride (TEBAC) (0.68 g, 3.0 mmol, 1.0 equiv.) and $NaHCO_3$ (0.63 g, 7.5 mmol, 2.5 equiv.). Allyl alcohol (0.31 mL, 4.5 mmol, 1.5 equiv.) and DMF (12 mL) were then added. The reaction mixture was heated to 50 °C and left to stir for 5 h. The reaction mixture was then cooled to room temperature and filtered through a short pad of silica (eluting with EtOAc). The filtrate was washed with water (2 × 10 mL), dried (Na_2SO_4) and concentrated *in vacuo*. Purification by column chromatography (SiO_2, pentane/Et_2O, 9:1) afforded the title compound as a yellow oil (0.22 g, 1.6 mmol, 53%).

R*f* 0.24 (pentane/Et_2O, 9:1), [UV, vanillin]

^{1}H NMR (500 MHz, $CDCl_3$) δ 9.83 (t, *J* = 1.3 Hz, 1H, H1), 7.14 (dd, *J* = 5.2, 1.0 Hz, 1H, H6), 6.92 (dd, *J* = 5.1, 3.4 Hz, 1H, H5), 6.82 (dt, *J* = 3.4, 1.0 Hz, 1H, H4), 3.18 (td, *J* = 7.3, 1.0 Hz, 2H, H3), 2.85 (td, *J* = 7.3, 1.3 Hz, 2H, H2).
^{13}C NMR (126 MHz, $CDCl_3$) δ 201.0, 143.0, 127.1, 124.9, 123.8, 45.5, 22.5.
Spectroscopic data in agreement with that reported previously.[60]

3-(Pyridin-3-yl)propanal, S86

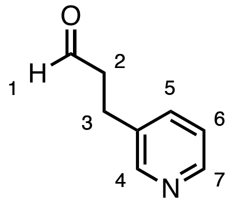

According to the procedure of Su et al.[60] To a flame-dried vial containing 3-iodo-pyridine (0.62 g, 3.0 mmol, 1.0 equiv.) at room temperature was added $Pd(OAc)_2$ (6.7 mg, 0.030 mmol, 0.01 equiv.), benzyltriethylammonium chloride (TEBAC) (0.68 g, 3.0 mmol, 1.0 equiv.) and $NaHCO_3$ (0.63 g, 7.5 mmol, 2.5 equiv.). Allyl alcohol (0.31 mL, 4.5 mmol, 1.5 equiv.) and DMF (12 mL) were then added. The reaction mixture was heated to 50 °C and left to stir for 5 h. The reaction mixture was then heated to 60 °C and stirred for a further 23 h. Upon completion, the reaction mixture was cooled to room temperature and filtered through a short pad of silica (eluting with EtOAc). The filtrate was washed with water (2 × 10 mL), dried (Na_2SO_4) and concentrated *in vacuo*. Purification by column chromatography (SiO_2, pentane/Et_2O, 1:1 → Et_2O) afforded the title compound as a yellow oil (0.18 g, 1.3 mmol, 45%).

R_f 0.07 (pentane/Et_2O, 3:2), [UV, vanillin]
^{1}H NMR (500 MHz, $CDCl_3$) δ 9.83 (t, *J* = 1.1 Hz, 1H, H1), 8.50 – 8.44 (m, 2H, H4 and H7), 7.52 (ddd, *J* = 7.7, 2.4, 1.7 Hz, 1H, H5), 7.22 (dd, *J* = 7.7, 4.8 Hz, 1H, H6), 2.96 (t, *J* = 7.4 Hz, 2H, H3), 2.82 (t, *J* = 7.4 Hz, 2H, H2).
^{13}C NMR (126 MHz, $CDCl_3$) δ 200.6, 150.0, 148.0, 136.0, 135.9, 123.6, 45.0, 25.3.
Spectroscopic data in agreement with that reported previously.[60]

3.2.6 Unsuccessful substrates

The following aldehyde substrates failed in the asymmetric ring-opening of [1.1.1]propellane **1**.

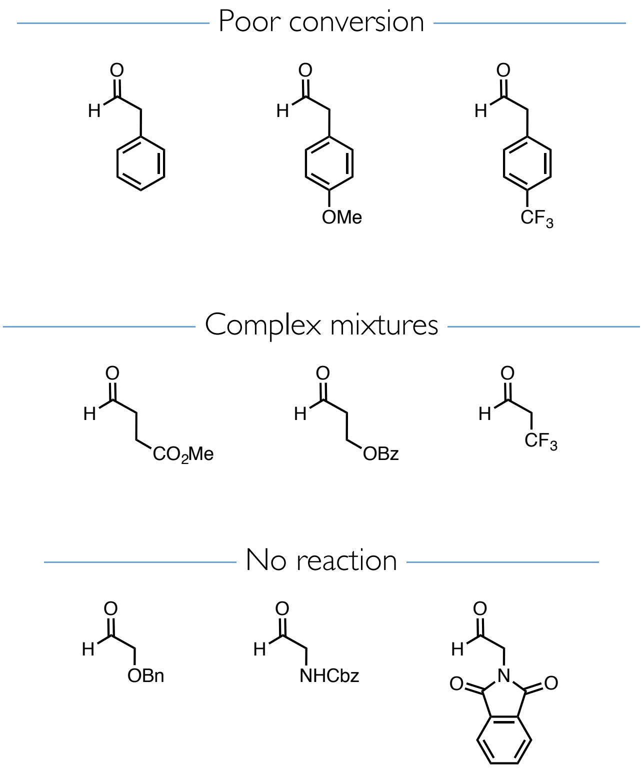

4. Computational data

4.1 QM calculations on a model system

QM calculations were run using ORCA (v. 4.1.1).[61] Optimizations and frequency calculations were carried out at the PBE0-D3BJ/def2-TZVP level of theory[62] using the SMD implicit solvent model for DME (user-defined parameters from ref 57[63]: Epsilon = 7.54, Refrac = 1.3772, Rsolv = 1.30, Soln = 1.3813, Soln25 = 1.3739, Sola = 0.00, Solb = 0.00, Solg = 0.0246, Solc = 0.00, Solh = 0.00),[64] Grimme's D3 empirical dispersion correction with Becke-Johnson (BJ) damping,[65] the RIJCOSX approximation using the appropriate auxiliary basis sets, and 'tight' optimization criteria (10^{-8} Ha tolerance for SCF, 10^{-6} tolerance for optimization step). The accuracy of this method was tested through comparison with results obtained at the B2GP-PLYP-D3BJ/def2-TZVP level of theory[66] with the same solvent model / resolution of the identity / optimization criteria, which we have previously shown to provide accurate geometries and energetics for intermediates in reactions of [1.1.1]propellane **1**.[67] Single point energies were calculated at the DLPNO-CCSD(T)/def2-TZVPP level of theory,[68] using the same solvent model, and 'Tight' PNO cut-offs ($T_{\text{CutPairs}} = 10^{-5}$, $T_{\text{CutPNO}} = 10^{-7}$, $T_{\text{CutMKN}} = 10^{-4}$). All calculations used Grid6/GridX6, corresponding to Lebedev-590 angular grid, and a radial integral accuracy (IntAcc) of 5.34. Stationary points for the model systems were characterized through calculation of the Hessian. Spurious imaginary frequencies below –50 cm^{-1} were ignored and converted to their real counterparts, as these correspond to numerical errors due to the solvent model, and all frequencies below 100 cm^{-1} were subsequently damped through the use of Grimme's quasi-RRHO approximation.[69] Free energy corrections were applied for a 1 M standard state at 283.15 K, using the *otherm* Python package.[70]

Transition state analogue conformational sampling was carried out using GFN2-xTB (v. 6.1), using the simulated annealing algorithm with the GBSA implicit solvent model for dichloromethane.[71] Conformers were generated for each compound in triplicate, where each run consisted an initial 16.67 ps molecular dynamics (Δt = 4 fs) at 149.07 K, followed by annealing from 298.15 K to 1000.00 K in three steps of 50 ps (150 ps total). Each conformer was then optimized at the GFN2-xTB level, and conformers above 20 kcal mol^{-1} of the lowest energy were removed. The structures from the three runs (~450 per system) were

combined, and conformers with RMSD < 0.7 Å on heavy atoms were removed using the RMSD filter built into the Python package *autodE*.[72] Single point energy calculations on the surviving conformers were obtained at the PBE-D3BJ/def2-SV(P) level of theory using the SMD solvent model for DME, and conformers within 3 kcal mol^{-1} of the lowest were optimized for 10 cycles at the SMD(DME)-PBE0-D3BJ/def2-SVP level of theory. The lowest energy conformer from this process was then reoptimized with 'normal' optimization criteria at the same level of theory.

Distortion / interaction analysis (DIA, also referred to as activation / strain analysis) was carried out on the model addition system,[73] where the barrier to addition ($\Delta E^{\ddagger}$) is divided into the distortion of the fragments required to adopt the transition state (TS) geometry (ΔE_{dist}) and the interaction of the two fragments in this distorted geometry (ΔE_{int}). NPA charges and spin populations were calculated using NBO v7.0. Non-covalent interaction (NCI) surfaces were calculated using Multiwfn v.3.6,[74] and are shown at an isovalue of 0.6 a.u.

4.2 Model radical system

4.2.1 Methyl substituent

To understand the nature of the addition of [1.1.1]propellane **1** to an α-iminyl radical, we first studied a model system in which the radical has a single methyl substituent and an unsubstituted pyrrolidine (**I**, Supplementary Figure 3). Addition of [1.1.1]propellane **1** to *E*/*Z*-**I** is endergonic and the activation free energy ($\Delta G^{\ddagger}$) primarily entropic in nature. As expected, a preference towards the *E* isomer along the pathway is observed. These results suggest that reversible addition is feasible, which may explain the dependence of enantioselectivity on the HAT catalyst.

Examination of the forming and breaking C–C bond distances at the *E*/*Z*-**I** TSs indicates that an earlier TS is observed for *Z*-**I** compared to *E*-**I**, which is corroborated by the smaller pyramidalization of the iminyl α-carbon (C_p) in the former. Despite this, the greater distortion energy observed for *Z*-**I** ($\Delta\Delta E_{dist}$ = 2.6 kcal mol^{-1}), arising from steric repulsion between the methyl substituent and the pyrrolidine ring, makes this pathway unfavourable.

The barrier to *E* / *Z* isomerisation of the α-iminyl radical was calculated to be 17.3 kcal mol^{-1}, so interception of this radical by **1** is faster than equilibriation and the *E* and *Z* α-iminyl radicals will not be found at their thermodynamic populations.

4.2.2 Moc substituent

Extending this model system to include a truncated methyl carbamate (NHMoc, **II**) sidechain to mimic the benzyl carbamate (NHCbz, **8**) led to a reduction in the activation energy and a slightly exergonic reaction compared with system **I**, with a small increase in the preference for *E* addition (Supplementary Figure 4). This is in line with the fact that now the *E*-**II** TS is earlier than *Z*-**II**, as shown by the longer forming C–C distance in the former ($\Delta r_{C\text{–}C}$ = 0.03 Å) and smaller pyramidalization of the iminyl α-carbon (ΔC_p = –1.3°). DIA reveals that the preference for the *E* TS is primarily due to minimization of distortion of the substrate at the TS compared to *Z*-**II**.

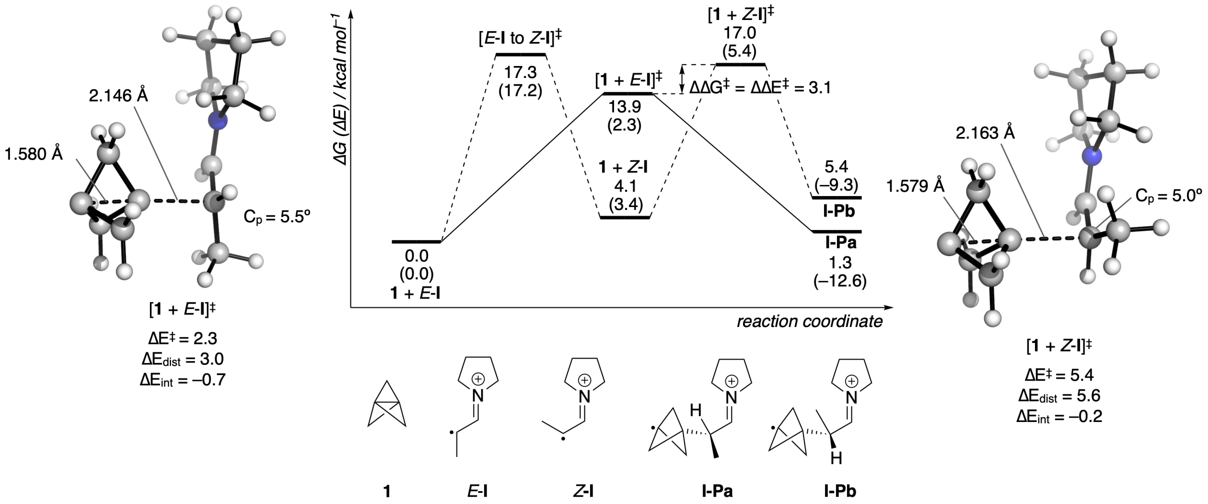

Supplementary Figure 3. *Free energy and (electronic energy) profiles of E/Z isomerization and the addition of [1.1.1]propellane* ***1*** *to E/Z-****I****, calculated at the SMD(DME)-DLPNO-CCSD(T)/def2-TZVPP (Tight PNO)//SMD(DME)-PBE0-D3BJ/def2-TZVP level of theory. Thermal corrections were applied for a 1 M standard state at 283.15 K.*

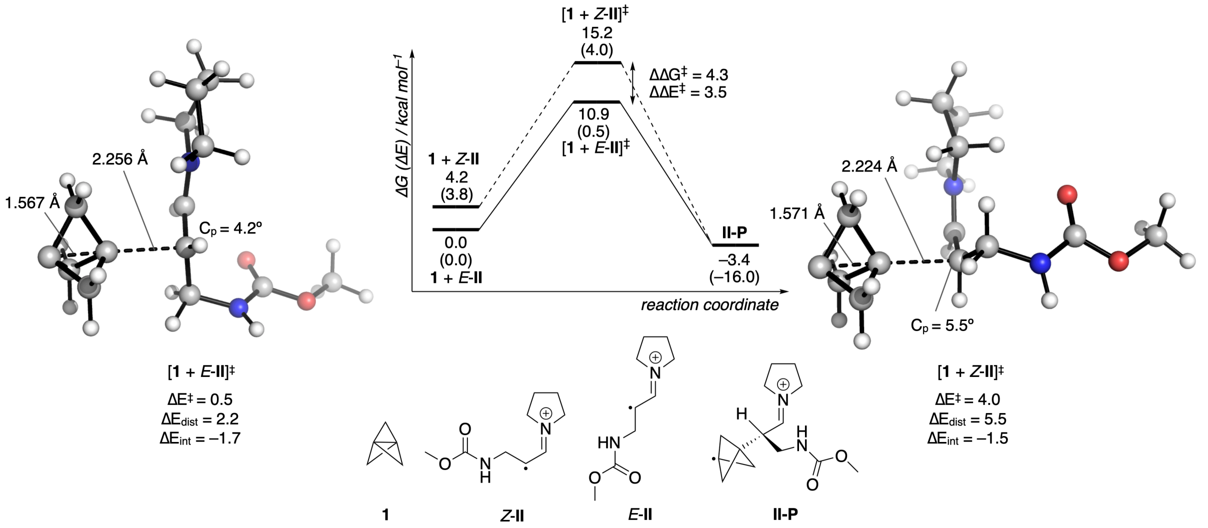

Supplementary Figure 4. *Free energy and (electronic energy) profiles for the addition of [1.1.1]propellane* ***1*** *to E/Z-****II****, calculated at the SMD(DME)-DLPNO-CCSD(T)/def2-TZVPP (Tight PNO)//SMD(DME)-PBE0-D3BJ/def2-TZVP level of theory. Thermal corrections were applied for a 1 M standard state at 283.15 K.*

4.3 Calculations on the full system

4.3.1 Methodology

Previous studies have found that reactions of [1.1.1]propellane **1** may be poorly described by single-hybrid DFT and by basis sets of less than triple-ζ quality.[67] Therefore, to extend our study to the full system (> 80 atoms) we explored the use of compatible methodologies based on the benchmark undertaken in reference.[67] Employing model system **I**, we performed relaxed PES scans using B2GP-PLYP-D3BJ and PBE0-D3BJ functionals in combination with def2-SVP and def2-TZVP basis sets (Supplementary Figure 5). We found that the cheaper PBE0-D3BJ/def2-SVP level of theory significantly underestimates the already small electronic barrier to addition. Indeed, attempts to find TSs of the full system using this methodology resulted in the loss of any small imaginary frequencies corresponding to the addition of [1.1.1]propellane **1** to the α-iminyl radical. B2GP-PLYP-D3BJ/def2-SVP gave comparable results to the reference B2GP-PLYP-D3BJ/def2-TZVP scan; however, it was found to still be too expensive to be applied to the full system.

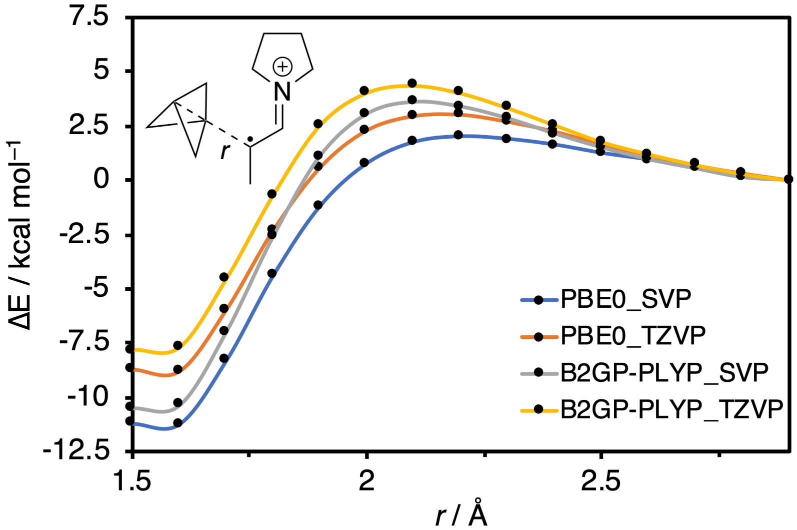

Supplementary Figure 5. *Relaxed potential energy surface scan for the addition of [1.1.1]propellane **1** to **I**.*

As a result, we chose to use constrained TS analogues with the forming C–C bond distances fixed to the obtained for model systems **I** and **II** at the SMD(DME)-PBE0-D3BJ/def2-TZVP level of theory. For model **I**, this methodology was found to reproduces the distances

obtained at the higher level of theory SMD(DME)-B2GP-PLYP-D3BJ/def2-TZVP, with a difference of only 0.06 Å which corresponds to an energy difference of < 0.1 kcal mol^{-1} at the SMD(DME)-B2GP-PLYP-D3BJ/def2-TZVPP level of theory. With this forming C–C distance fixed, conformational sampling of the full system was carried out as described above using GFN2-xTB, followed by constrained optimization at the SMD(DME)-PBE0-D3BJ/def2-SVP level of theory and single-point energies at SMD(DME)-B2GP-PLYP-D3BJ/def2-TZVPP.

To test the validity of the use of the constrained TS analogues, we constructed a model system (**III**) of an intermediate size and performed both a constrained optimization with the forming bond lengths fixed according to Supplementary Table 8, and a full TS optimization (Supplementary Figure 6). We found that this approach resulted in constrained structures within 0.2 kcal mol^{-1} of the fully optimized TSs, and almost no difference in the calculated $\Delta\Delta E^{\ddagger}$ between the *Si*-*E*, *Re*-*E* and *Re*-*Z* approaches. We also noted that the differences in free energies ($\Delta\Delta G^{\ddagger}$) and electronic energies ($\Delta\Delta E^{\ddagger}$) for the TS barriers were in excellent agreement, therefore electronic energy differences could be used to study the selectivity of the full system, as the increased size and flexibility inevitably results in many low energy rotational/vibrational modes that will introduce errors into the free energy calculations.

Supplementary Table 8. *Key C–C bond forming distance for the TSs with each model system used for constraints in the full system.*

Geometry	Sidechain	$R_{C–C}$ / Å*
E	Me	2.146
Z	Me	2.163
E	Moc	2.256
Z	Moc	2.182

*Calculated at SMD(DME)-PBE0-D3BJ/def2-TZVP

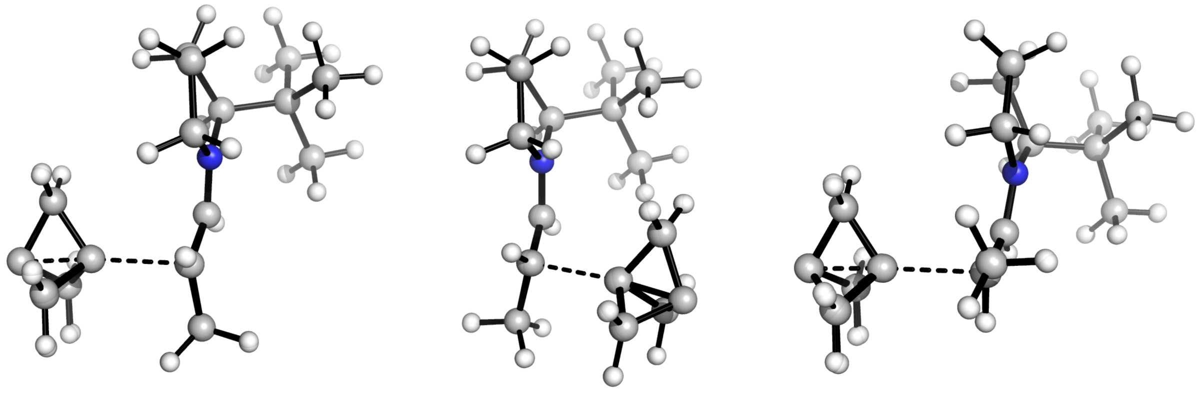

Si-*E*-III	Re-*E*-III	Re-*Z*-III
$\Delta\Delta G^{\ddagger}_{fullTS} = 0.0$	$\Delta\Delta G^{\ddagger}_{fullTS} = 0.1$	$\Delta\Delta G^{\ddagger}_{fullTS} = 2.9$
$\Delta\Delta E^{\ddagger}_{fullTS} = 0.0$	$\Delta\Delta E^{\ddagger}_{fullTS} = -0.1$	$\Delta\Delta E^{\ddagger}_{fullTS} = 3.0$
$\Delta\Delta E^{\ddagger}_{constrained} = 0.0$	$\Delta\Delta E^{\ddagger}_{constrained} = -0.2$	$\Delta\Delta E^{\ddagger}_{constrained} = 3.0$
$[\Delta E_{constrained-fullTS} = <0.1]$	$[\Delta E_{constrained-fullTS} = -0.2]$	$[\Delta E_{constrained-fullTS} = -0.1]$

Supplementary Figure 6. *Overlaid structures of constrained and fully optimized TSs for* Si-*E*-***III***, Re-*E*-***III*** *and* Re-*Z*-***III*** *approaches, and their respective free energy and electronic energy differences, calculated at the SMD(DME)-PBE0-D3BJ/def2-SVP level of theory. All energies are reported in kcal mol*$^{-1}$.

We also tested whether the electron-deficient pyrrolidine substituents might play a (stereo)electronic role in the reaction through altering the charge or spin population of the α-iminyl radical. Natural population analysis (NPA) charges and spin populations were calculated for the lowest energy conformers of **I**, **II** and **8'** using NBO, and are shown in Supplementary Figure 7. No significant differences are seen, suggesting that the pyrrolidine substituents do not alter the nature of the α-iminyl radical.

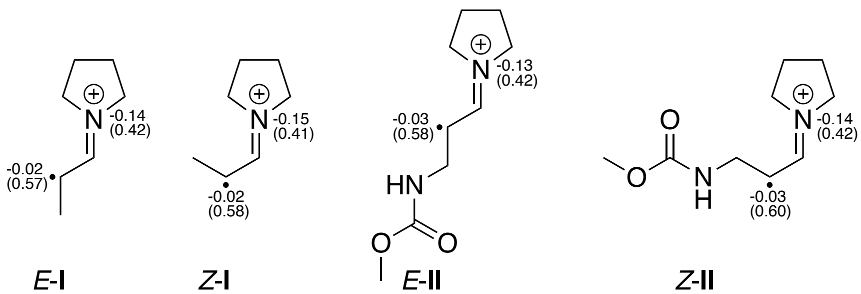

E-**I** *Z*-**I** *E*-**II** *Z*-**II**

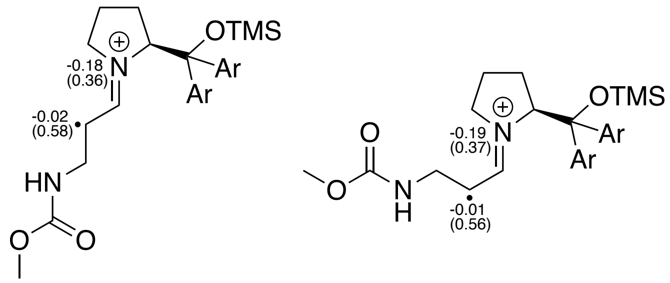

E-**8’** *Z*-**8’**

Supplementary Figure 7. *NPA charges and (spin populations), calculated at the SMD(DME)-PBE0-D3BJ/def2-SVP level of theory.*

4.3.2 Enamine *E* / *Z* equilibria

Equilibrium constants, K_{eq} = [*E*-enamine] / [*Z*-enamine], were calculated for enamines derived from **8’**, **8’-H**, **12** and **15** (**en-8’**, **en-8’-H**, **en-12** and **en-15**, respectively), using free energies obtained at the [SMD(DME)-B2GP-PLYP-D3BJ/def2-TZVPP//SMD(DME)-PBE0-D3BJ/def2-SVP] level of theory at 283.15 K (Supplementary Figure 8). In each case, the *E* isomer was found to be favoured over *Z*, primarily due to 1,3-allylic strain between the pyrrolidine and the α-subtituent.

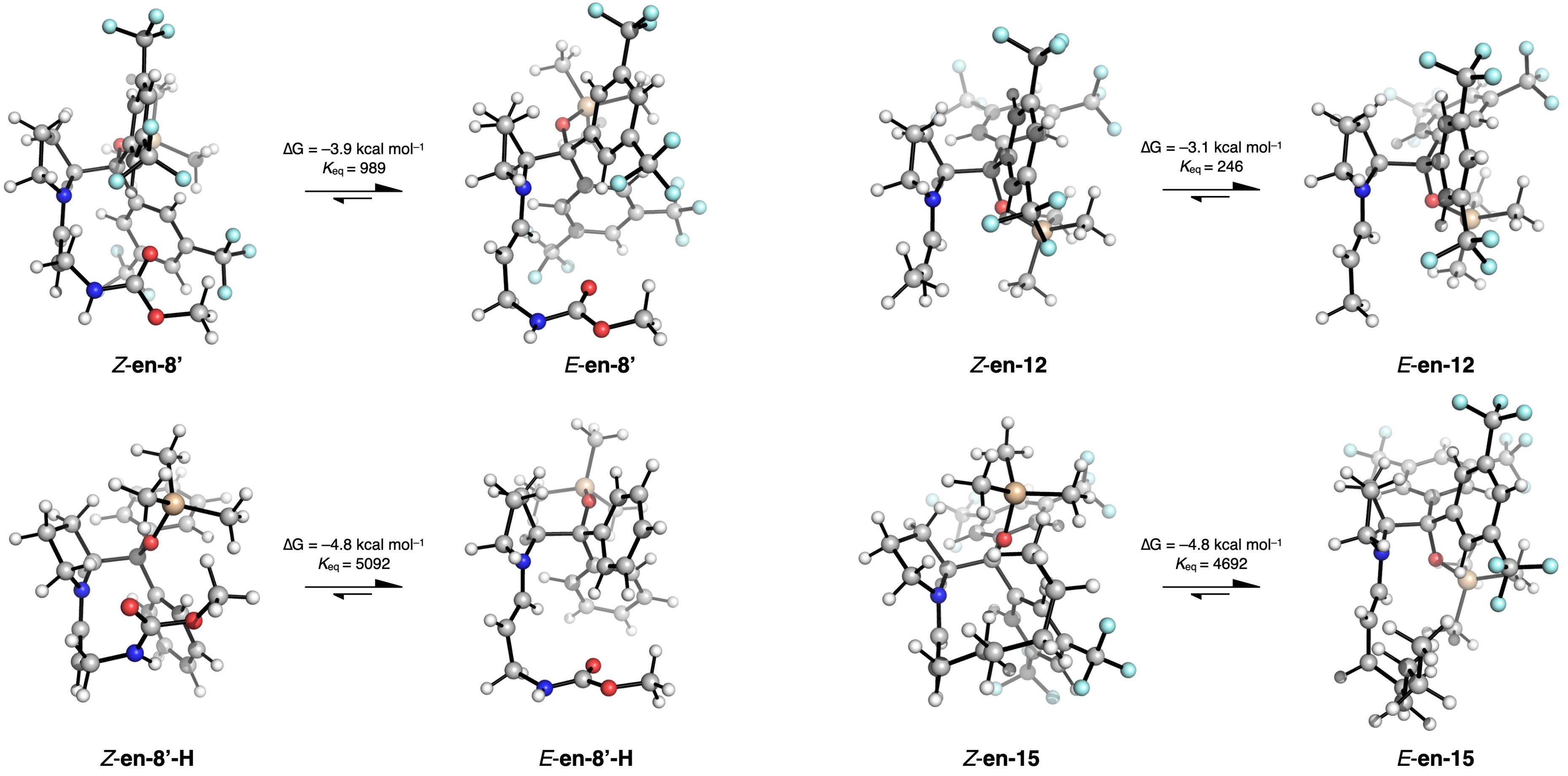

Supplementary Figure 8. *Enamine E/Z isomer equilibria for* ***en-8’***, ***en-8’-H***, ***en-12*** *and* ***en-15*** *at 283.15 K. Free energies are reported in kcal mol^{-1}, and were calculated at the SMD(DME)-B2GP-PLYP-D3BJ/def2-TZVPP//SMD(DME)-PBE0-D3BJ/def2-SVP level of theory.*

4.3.3 Transition state models

Conformational sampling with the forming C–C bond distance constrained to mimic the TS revealed the lowest energy conformers for s-*trans* *Si*-*E*, *Re*-*E* and *Re*-*Z* addition paths. No conformers corresponding *Si*-*Z* or s-*cis* addition were found, suggesting that these pathways are > 10 kcal mol^{-1} higher in energy.

4.3.3.1 Moc substituent (TS-8')

For system **8'** (Supplementary Figure 9), we observed high selectivity for *Si*-*E* addition ($\Delta\Delta E^{\ddagger}_{Re/Si}$ = 4.9 kcal mol^{-1}, Supplementary Figure 9a). This selectivity arises due to an increase in distortion of the catalyst to adopt either *Re* TS. These effects can be attributed to both the enhanced CH_2 / pyrrolidine steric repulsion for the *Re*-*Z* conformer (Supplementary Figure 9b), and the loss of stabilizing C–H···O, C–H···F and dispersion interactions between the Moc sidechain and the pyrrolidine substituents upon *Re*-*E* addition (Supplementary Figure 9c). In the case of *Re*-*E* addition, we also note the formation of stabilizing C–H···F and dispersion interactions between [1.1.1]propellane **1** and the pyrrolidine substituents, in this case increasing the favourability of this addition mode over the *Re*-*Z* addition in addition to reduced CH_2 / pyrrolidine steric repulsion.

4.3.3.2 Moc substituent, diphenylprolinol catalyst (TS-8'-H)

To further examine how these non-covalent interactions manifest in the selectivity of the addition, we took the lowest energy TS conformers for **8'** and replaced the CF_3 groups with H atoms (Supplementary Figure 10). A C–H···F interaction is lost through this exchange, and the remaining C–H···O and dispersion interactions are weakened as the aryl rings become less electron deficient. As a result, selectivity for *Si*-*E* addition decreases ($\Delta\Delta E^{\ddagger}_{Re/Si}$ = 0.4 kcal mol^{-1}, Supplementary Figure 10a). $\Delta\Delta E_{dist}$ drops to 2.0 kcal mol^{-1} for *Re*-*E* addition (Supplementary Figure 10c), compared with 6.0 kcal mol^{-1} for *Re*-*E*-**TS-8'**, which is primarily due to the lower cost of removing the weaker stabilizing interactions between the Moc sidechain and the aryl groups of the pyrrolidine.

4.3.3.3 Methyl substituent (TS-12)

There is also a decrease in preference for *Si-E* addition in **TS-12** compared with **TS-8’** ($\Delta\Delta E^{\ddagger}_{Re/Si}$ = 2.2 kcal mol^{-1}, Supplementary Figure 11a). This difference can again be attributed to the weakened non-covalent interactions, where in this case only CH_2 / pyrrolidine steric repulsion and weak dispersion interactions between the methyl sidechain and an aryl group are responsible for the selectivity between *Si-E* and *Re-Z* addition.

4.3.3.4 Hexyl substituent (TS-15)

By increasing the length of the alkyl sidechain from methyl to hexyl (i.e. propanal to octanal), selectivity is recovered due to an increase in dispersion and C–H···F interactions between the sidechain and one aryl group (Supplementary Figure 11a). An increase in distortion from the *Si-E* to *Re-Z* TS accounts for the enhanced selectivity, with an increased steric clash between CH_2 / pyrrolidine and a weakening of attractive NCIs between the sidechain and the pyrrolidine substituents. No conformers could be located for the *Re-E* pathway within the 10 kcal mol^{-1} window used for conformer generation and pruning.

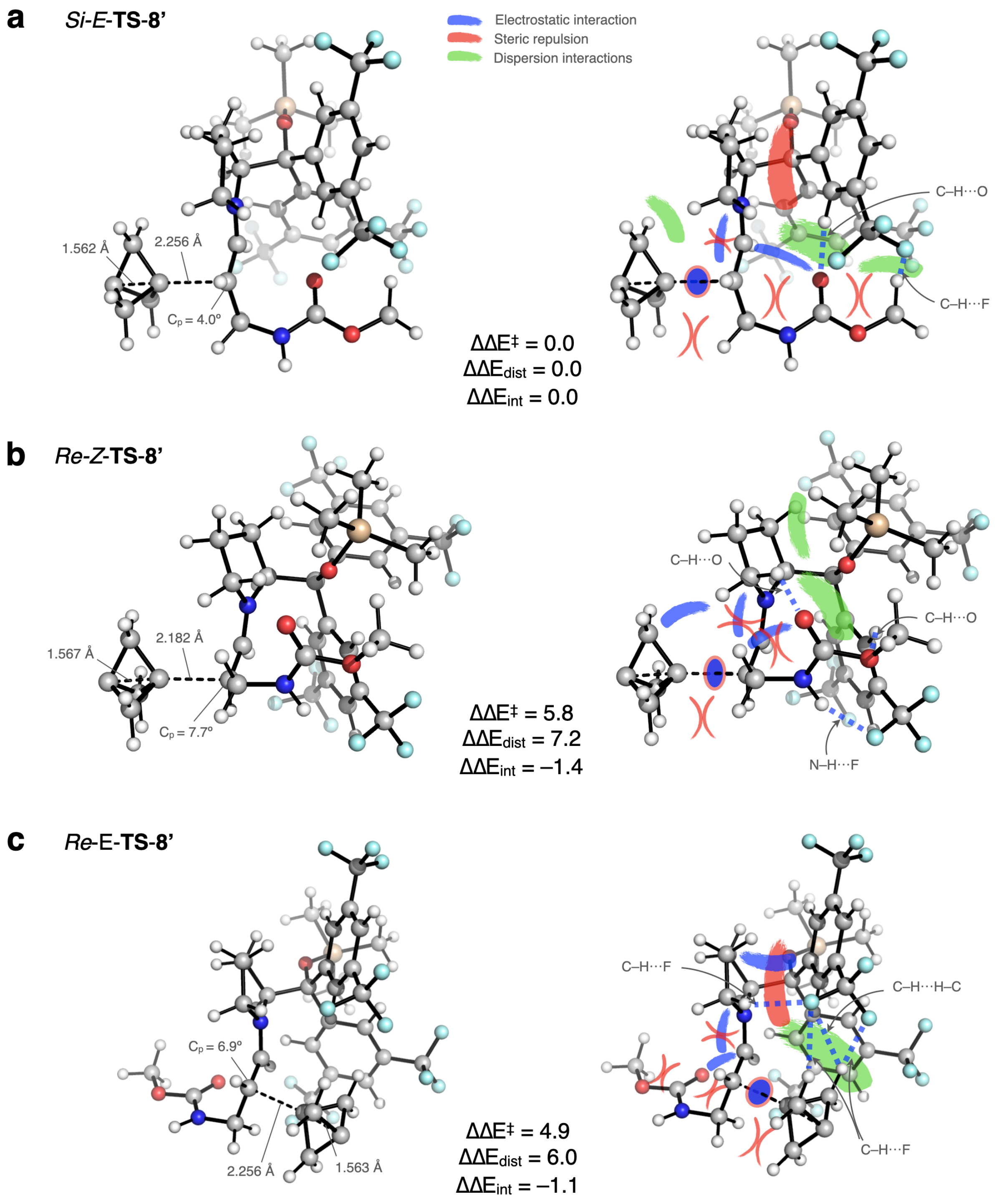

Supplementary Figure 9. *Constrained TS analogues for system* ***TS-8’*** *showing key geometric features (left) and stylized non-covalent interactions (right). Electronic energies and distortion / interaction values are reported in kcal mol^{-1}, and were calculated at the SMD(DME)-B2GP-PLYP-D3BJ/def2-TZVPP//SMD(DME)-PBE0-D3BJ/def2-SVP level of theory, with the forming C–C distances constrained to their distances from Supplementary Table 8. Calculated NCI plots are shown in Supplementary Figure 13. Calculated values are reported in Supplementary Table 17.*

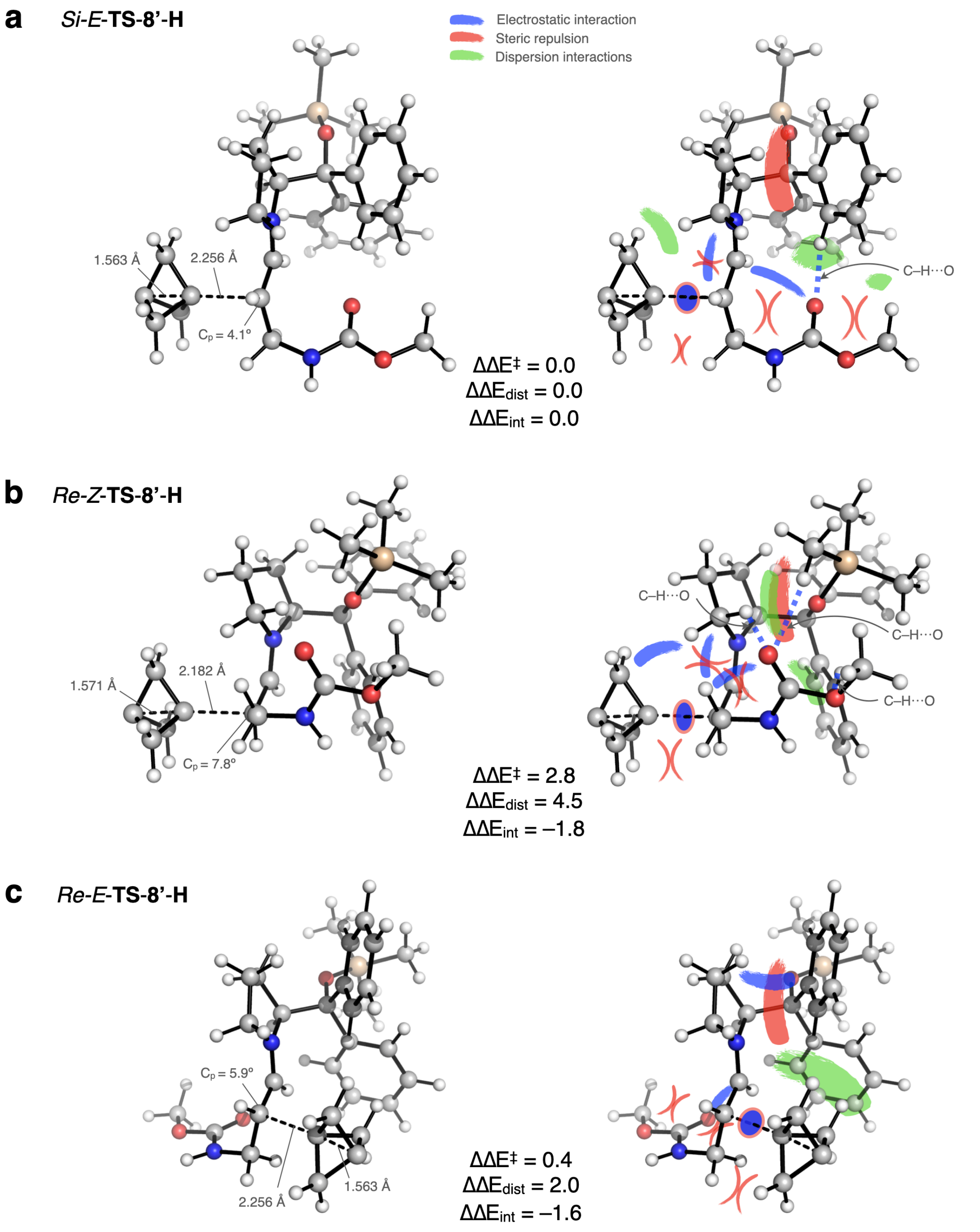

Supplementary Figure 10. *Constrained TS analogues for system* ***TS-8'-H*** *showing key geometric features (left) and stylized non-covalent interactions (right). Electronic energies and distortion / interaction values are reported in kcal mol^{-1}, and were calculated at the SMD(DME)-B2GP-PLYP-D3BJ/def2-TZVPP//SMD(DME)-PBE0-D3BJ/def2-SVP level of theory, with the forming C–C distances constrained to their distances from Supplementary Table 8. Calculated NCI plots are shown in Supplementary Figure 13. Calculated values are reported in Supplementary Table 18.*

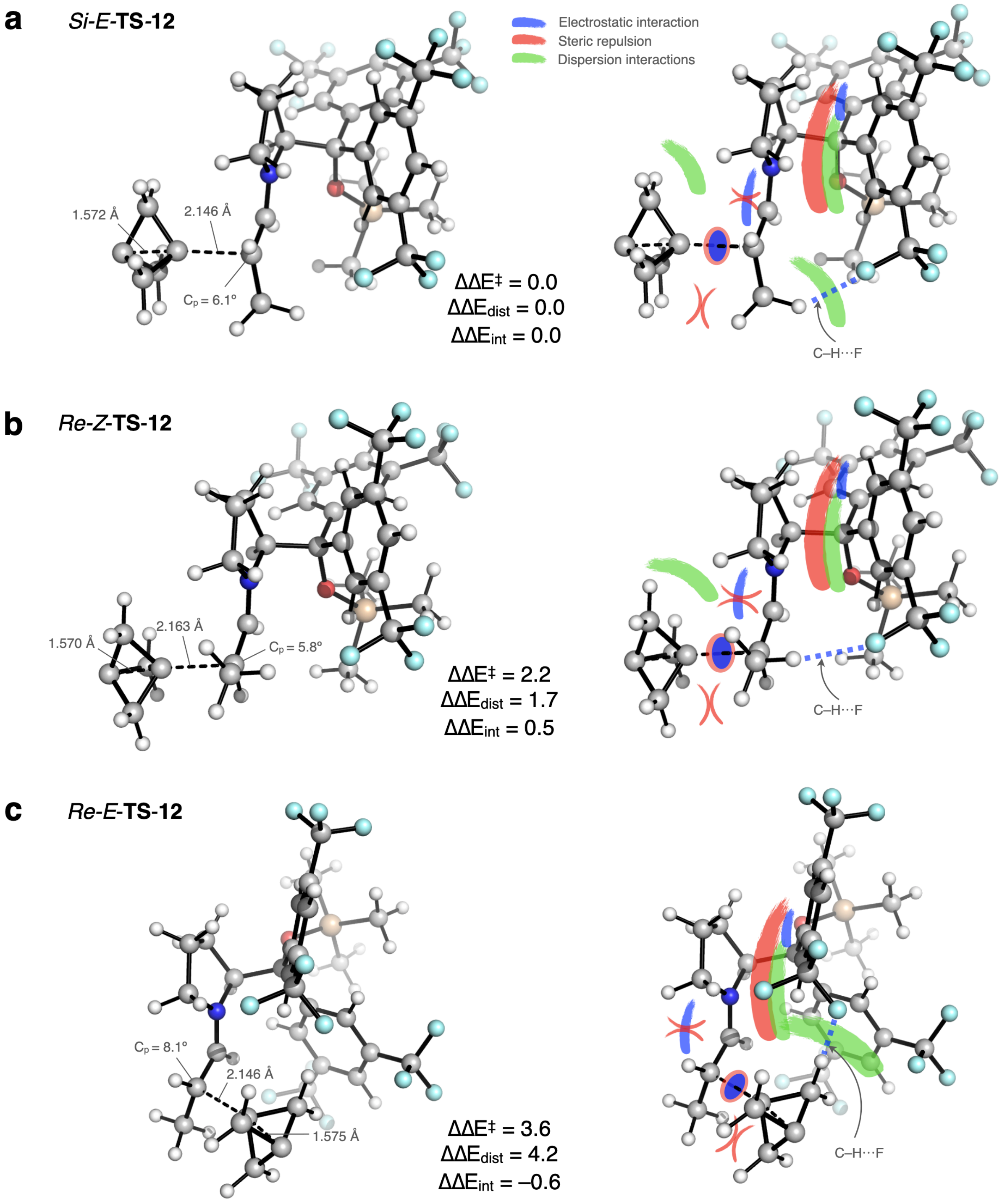

Supplementary Figure 11. *Constrained TS analogues for system* ***TS-12*** *showing key geometric features (left) and stylized non-covalent interactions (right). Electronic energies and distortion / interaction values are reported in kcal mol*$^{-1}$*, and were calculated at the SMD(DME)-B2GP-PLYP-D3BJ/def2-TZVPP//SMD(DME)-PBE0-D3BJ/def2-SVP level of theory, with the forming C–C distances constrained to their distances from Supplementary Table 8. Calculated NCI plots are shown in Supplementary Figure 13. Calculated values are reported in Supplementary Table 19.*

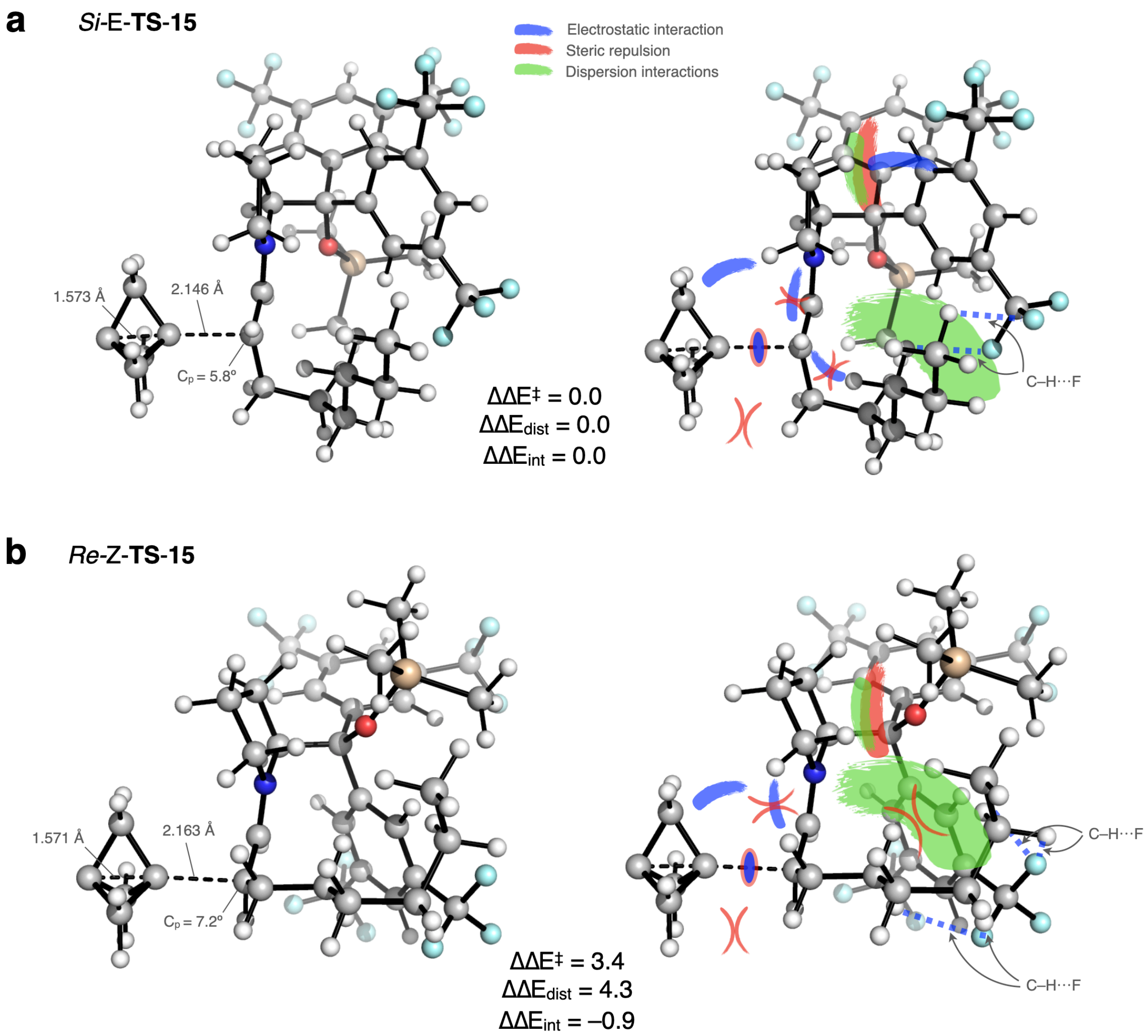

Supplementary Figure 12. *Constrained TS analogues for system* ***TS-15*** *showing key geometric features (left) and stylized non-covalent interactions (right). Electronic energies and distortion / interaction values are reported in kcal mol^{-1}, and were calculated at the SMD(DME)-B2GP-PLYP-D3BJ/def2-TZVPP//SMD(DME)-PBE0-D3BJ/def2-SVP level of theory, with the forming C–C distances constrained to their distances from Supplementary Table 8. Calculated NCI plots are shown in Supplementary Figure 13. Calculated values are reported in Supplementary Table 20.*

4.3.3.5 NCI plots

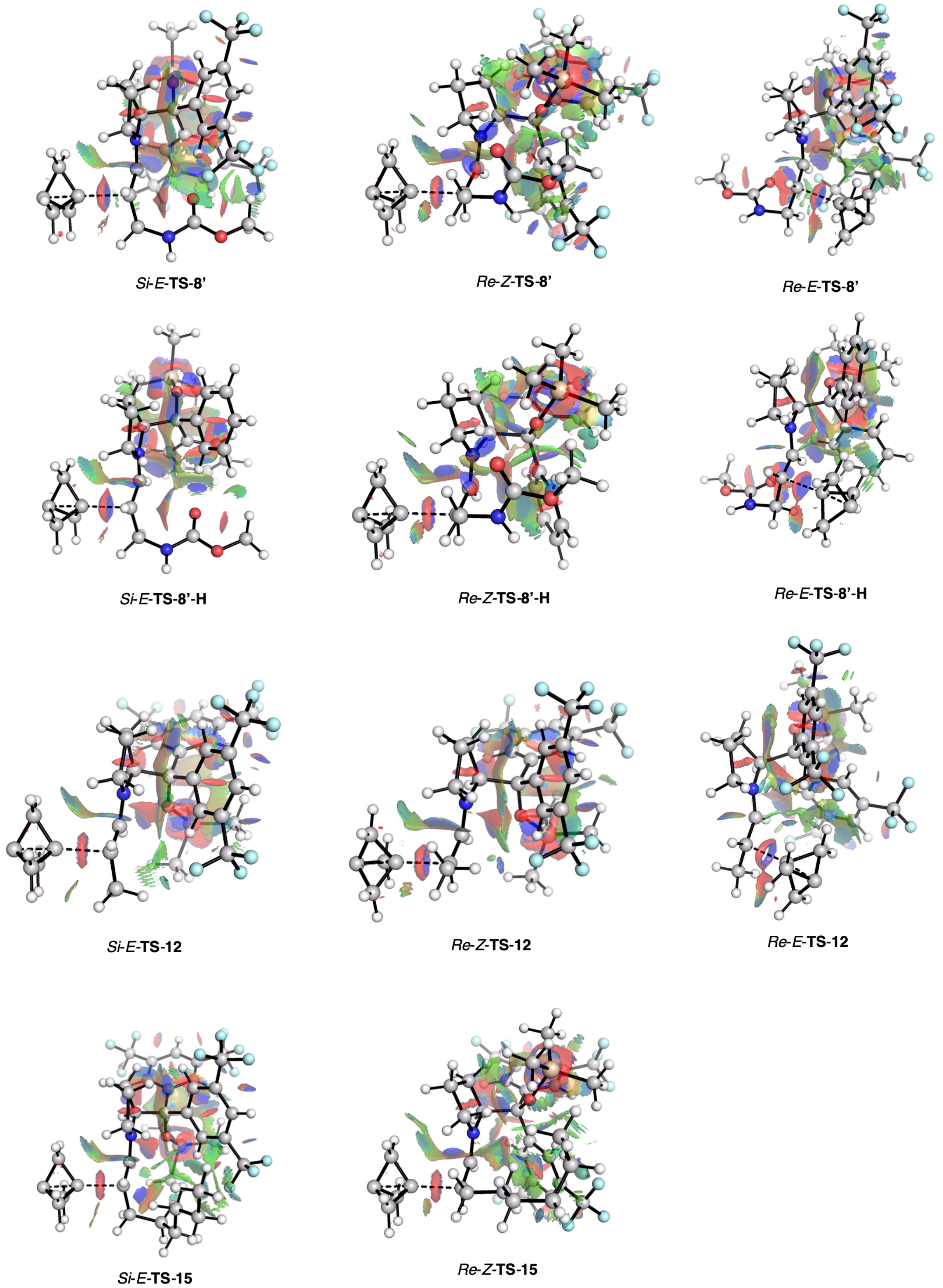

Supplementary Figure 13. *Non-covalent interactions present in each constrained TS analogue for **TS-8’**, **TS-8’-H**, **TS-12** and **TS-15** calculated at the SMD(DME)-PBE0-D3BJ/def2-SVP level of theory. NCI isovalue = 0.6.*

4.4 Energetics and thermodynamic quantities

Supplementary Table 9. *Thermodynamic quantities for [1.1.1]propellane **1** addition to model system **I**. Geometries and thermodynamic corrections calculated at SMD(DME)-PBE0-D3BJ/def2-TZVP for a 1 M standard state and 283.15 K, single point energies at SMD(DME)-B2GP-PLYP-D3BJ/def2-TZVPP and SMD(DME)-DLPNO-CCSD(T) (Tight PNO)/def2-TZVPP. All energies are reported in Ha, TS imaginary frequency in* cm^{-1}.

Species	E_{el}	ZPE	H	Tqh-S	Total corr	qh-G	E_{el}(B2GP-PLYP)	E_{el}(DLPNO)	G(B2GP-PLYP)	G(DLPNO)	TS freq.
[1.1.1]Propellane (**1**)	-193.87362	0.09345	-193.77565	0.02420	0.07377	-193.79985	-193.88712	-193.65880	-193.81335	-193.58502	-
E-**I**	-328.87700	0.18941	-328.67853	0.03819	0.16029	-328.71672	-328.89961	-328.52881	-328.73933	-328.36852	-
[*E*-**I** to *Z*-**I**]‡	-328.84661	0.18765	-328.65070	0.03553	0.16039	-328.68622	-328.87168	-328.50133	-328.71129	-328.34094	-305
Z-**I**	-328.87113	0.18989	-328.67227	0.03743	0.16143	-328.70969	-328.89372	-328.52334	-328.73229	-328.36191	-
[**1** + *E*-**I**]‡	-522.74639	0.28536	-522.44883	0.04511	0.25246	-522.49393	-522.78040	-522.18389	-522.52794	-521.93143	-214
[**1** + *Z*-**I**]‡	-522.74166	0.28572	-522.44378	0.04527	0.25262	-522.48904	-522.77555	-522.17907	-522.52293	-521.92645	-203
**I-Pa**	-522.76609	0.28859	-522.46576	0.04400	0.25634	-522.50976	-522.80040	-522.20775	-522.54406	-521.95141	-
**I-Pb**	-522.76070	0.28913	-522.46007	0.04321	0.25742	-522.50328	-522.79477	-522.20240	-522.53735	-521.94498	-

Supplementary Table 10. *Differences in thermodynamic quantities for [1.1.1]propellane **1** addition to model system **I**. Geometries and thermodynamic corrections calculated at SMD(DME)-PBE0-D3BJ/def2-TZVP for a 1 M standard state and 283.15 K, single point energies at SMD(DME)-B2GP-PLYP-D3BJ/def2-TZVPP and SMD(DME)-DLPNO-CCSD(T) (Tight PNO)/def2-TZVPP. All energies are reported in kcal* mol^{-1}.

System	ΔE_{el}	ΔZPE	ΔH	TΔqh-S	ΔTotal corr	Δqh-G	ΔE_{el}(B2GP-PLYP)	ΔE_{el}(DLPNO)	ΔG(B2GP-PLYP)	ΔG(DLPNO)
**1** + *E*-**I**	0.0	0.0	0.0	0.0	0.0	0.0	0.0	0.0	0.0	0.0
**1** + [*E*-**I** to *Z*-**I**]‡	19.1	-1.1	17.5	-1.7	0.1	19.1	17.5	17.2	17.6	17.3
**1** + *Z*-**I**	3.7	0.3	3.9	-0.5	0.7	4.4	3.7	3.4	4.4	4.1
[**1** + *E*-**I**]‡	2.7	1.6	3.4	-10.8	11.5	14.2	4.0	2.3	15.5	13.9
[**1** + *Z*-**I**]‡	5.6	1.8	6.5	-10.7	11.6	17.3	7.0	5.4	18.7	17.0
**I-Pa**	-9.7	3.6	-7.3	-11.5	12.1	2.4	-8.6	-12.6	5.4	1.3
**I-Pb**	-6.3	3.9	-3.7	-12.0	12.8	6.4	-5.0	-9.3	9.6	5.4

Supplementary Table 11. *Thermodynamic quantities for [1.1.1]propellane **1** addition to model system **II**. Geometries and thermodynamic corrections calculated at SMD(DME)-PBE0-D3BJ/def2-TZVP for a 1 M standard state and 283.15 K, single point energies at SMD(DME)-DLPNO-CCSD(T) (Tight PNO)/def2-TZVPP. All energies are reported in Ha, TS imaginary frequency in* cm^{-1}.

Name	E_{el}	ZPE	H	Tqh-S	Total corr	qh-G	E_{el}(DLPNO)	G(DLPNO)	TS freq.
[1.1.1]Propellane (**1**)	-193.87362	0.09345	-193.77565	0.02420	0.07377	-193.79985	-193.65880	-193.58502	-
E-**II**	-611.93872	0.25056	-611.67579	0.04580	0.21713	-611.72159	-611.36109	-611.14396	-
Z-**II**	-611.93108	0.25048	-611.66804	0.04663	0.21641	-611.71467	-611.35413	-611.13773	-
[**1** + *E*-**II**]‡	-805.80946	0.34621	-805.44652	0.05539	0.30755	-805.50191	-805.01911	-804.71156	-119
[**1** + *Z*-**II**]‡	-805.80293	0.34627	-805.43911	0.05777	0.30605	-805.49688	-805.01187	-804.70582	-182
**II-P**	-805.83290	0.34977	-805.46621	0.05556	0.31113	-805.52177	-805.04545	-804.73432	-

Supplementary Table 12. *Differences in thermodynamic quantities for [1.1.1]propellane **1** addition to model system **II**. Geometries and thermodynamic corrections calculated at SMD(DME)-PBE0-D3BJ/def2-TZVP for a 1 M standard state and 283.15 K, single point energies SMD(DME)-DLPNO-CCSD(T) (Tight PNO)/def2-TZVPP. All energies are reported in kcal* mol^{-1}.

System	Δ E_{el}	ΔZPE	ΔH	TΔqh-S	ΔTotal corr	Δqh-G	ΔE_{el}(DLPNO)	ΔG(DLPNO)
**1** + *E*-**II**	0.0	0.0	0.0	0.0	0.0	0.0	0.0	0.0
**1** + *Z*-**II**	4.8	-0.1	4.9	0.5	-0.5	4.3	4.4	3.9
[**1** + *E*-**II**]‡	1.8	1.4	3.1	-9.2	10.4	12.3	0.5	10.9
[**1** + *Z*-**II**]‡	5.9	1.4	7.7	-7.7	9.5	15.4	5.0	14.5
**II-P**	-12.9	3.6	-9.3	-9.1	12.7	-0.2	-16.0	-3.4

Supplementary Table 13. *Thermodynamic quantities for [1.1.1]propellane* ***1*** *addition to model system* ***III****. Geometries and thermodynamic corrections calculated at SMD(DME)-PBE0-D3BJ/def2-SVP for a 1 M standard state and 283.15 K. Constrained optimizations at the same level of theory used fixed forming C–C bond lengths from Supplementary Table 8. All energies are reported in Ha, TS imaginary frequency in cm^{-1}.*

Name	E_{el}	ZPE	H	Tqh-S	Total corr	qh-G	TS freq.	E_{el}(constrained)
Si-E-**III** TS	-679.16429	0.39825	-678.74921	0.05399	0.36109	-678.80320	-181	-679.16436
Re-E-**III** TS	-679.16447	0.39827	-678.74939	0.05368	0.36140	-678.80307	-165	-679.16471
Re-Z-**III** TS	-679.15945	0.39836	-678.74414	0.05443	0.36088	-678.79857	-141	-679.15966

Supplementary Table 14. *Differences in thermodynamic quantities for [1.1.1]propellane* ***1*** *addition to model system* ***III****. Geometries and thermodynamic corrections calculated at SMD(DME)-PBE0-D3BJ/def2-SVP for a 1 M standard state and 283.15 K. Constrained optimizations at the same level of theory used fixed forming C–C bond lengths from Supplementary Table 8. All energies are reported in kcal mol^{-1}.*

Name	ΔE_{el}	ΔZPE	ΔH	TΔqh-S	ΔTotal corr	Δqh-G	ΔE_{el}(constrained)
Si-E-**III** TS	0.0	0.0	0.0	0.0	0.0	0.0	0.0
Re-E-**III** TS	-0.1	0.0	-0.1	-0.2	0.2	0.1	-0.2
Re-Z-**III** TS	3.0	0.1	3.2	0.3	-0.1	2.9	3.0

Supplementary Table 15. *Thermodynamic quantities for E/Z-enamines* ***en-8’***, ***en-8’-H***, ***en-12*** *and* ***en-15***. *Geometries and thermodynamic corrections calculated at SMD(DME)-PBE0-D3BJ/def2-SVP for a 1 M standard state and 283.15 K. All energies are reported in Ha.*

System	E_{el}	ZPE	H	Tqh-S	Total corr	qh-G	E_{el}(B2GP-PLYP)	G(B2GP-PLYP)
E-**en-8'**	-2941.26350	0.56772	-2940.65081	0.11135	0.50134	-2940.76216	-2944.82208	-2944.32074
Z-**en-8'**	-2941.26035	0.56846	-2940.64729	0.11118	0.50187	-2940.75848	-2944.81643	-2944.31456
E-**en-8'-H**	-1595.33970	0.54900	-1594.75982	0.08236	0.49752	-1594.84218	-1596.96102	-1596.46350
Z-**en-8'-H**	-1595.33579	0.54971	-1594.75538	0.08176	0.49866	-1594.83713	-1596.95450	-1596.45585
E-**en-12**	-2658.51297	0.50642	-2657.96666	0.10164	0.44467	-2658.06829	-2661.70151	-2661.25684
Z-**en-12**	-2658.50852	0.50679	-2657.96188	0.10123	0.44541	-2658.06311	-2661.69731	-2661.25190
E-**en-15**	-2854.70886	0.65073	-2854.01216	0.11483	0.58188	-2854.12698	-2858.10936	-2857.52748
Z-**en-15**	-2854.70227	0.65122	-2854.00537	0.11399	0.58291	-2854.11935	-2858.10281	-2857.51990

Supplementary Table 16. *Differences in thermodynamic quantities for E/Z-enamines* ***en-8’***, ***en-8’-H***, ***en-12*** *and* ***en-15***. *Geometries and thermodynamic corrections calculated at SMD(DME)-PBE0-D3BJ/def2-TZVP for a 1 M standard state and 283.15 K, single point energies at SMD(DME)-B2GP-PLYP-D3BJ/def2-TZVPP. All energies are reported in kcal mol^{-1}.*

System	Δ E_{el}	ΔZPE	ΔH	TΔqh-S	ΔTotal corr	Δqh-G	ΔE_{el}(B2GP-PLYP)	ΔG(B2GP-PLYP)
E-**en-8'**	0.0	0.0	0.0	0.0	0.0	0.0	0.0	0.0
Z-**en-8'**	2.0	0.5	2.2	-0.1	0.3	2.3	3.5	3.9
E-**en-8'-H**	0.0	0.0	0.0	0.0	0.0	0.0	0.0	0.0
Z-**en-8'-H**	2.5	0.4	2.8	-0.4	0.7	3.2	4.1	4.8
E-**en-12**	0.0	0.0	0.0	0.0	0.0	0.0	0.0	0.0
Z-**en-12**	2.8	0.2	3.0	-0.3	0.5	3.2	2.6	3.1
E-**en-15**	0.0	0.0	0.0	0.0	0.0	0.0	0.0	0.0
Z-**en-15**	4.1	0.3	4.3	-0.5	0.7	4.8	4.1	4.8

Supplementary Table 17. *Electronic energies and distortion / interaction analysis for constrained TS models of system* ***8'****, calculated at the SMD(DME)-B2GP-PLYP-D3BJ/def2-TZVPP//SMD(DME)-PBE0-D3BJ/def2-SVP level of theory. The forming bond lengths were constrained to their lengths in Supplementary Table 8. Energies are reported in kcal mol^{-1}.*

TS model	E_{el}	$\Delta\Delta E_{el}$	$\Delta\Delta E_{dist}$	$\Delta\Delta E_{int}$
Si-*E*-**TS**-**8'**	-3138.51692	0.0	0.0	0.0
Re-*E*-**TS**-**8'**	-3138.50918	4.9	6.0	-1.1
Re-*Z*-**TS**-**8'**	-3138.50768	5.8	7.2	-1.4

Supplementary Table 18. *Electronic energies for constrained TS models of system* ***8'-H****, calculated at the SMD(DME)-B2GP-PLYP-D3BJ/def2-TZVPP//SMD(DME)-PBE0-D3BJ/def2-SVP level of theory. The forming bond lengths were constrained to their lengths in Supplementary Table 8. Energies are reported in kcal mol^{-1}.*

TS model	E_{el}	$\Delta\Delta E_{el}$	$\Delta\Delta E_{dist}$	$\Delta\Delta E_{int}$
Si-*E*-**TS**-**8'-H**	-1790.65868	0.0	0.0	0.0
Re-*E*-**TS**-**8'-H**	-1790.65799	0.4	2.0	-1.6
Re-*Z*-**TS**-**8'-H**	-1790.65429	2.8	4.5	-1.8

Supplementary Table 19. *Electronic energies and distortion / interaction analysis for constrained TS models of system* ***12****, calculated at the SMD(DME)-B2GP-PLYP-D3BJ/def2-TZVPP//SMD(DME)-PBE0-D3BJ/def2-SVP level of theory. The forming bond lengths were constrained to their lengths in Supplementary Table 8. Energies are reported in kcal mol^{-1}.*

TS model	E_{el}	$\Delta\Delta E_{el}$	$\Delta\Delta E_{dist}$	$\Delta\Delta E_{int}$
Si-*E*-**TS**-**12**	-2855.39839	0.0	0.0	0.0
Re-*E*-**TS**-**12**	-2855.39261	3.6	4.2	-0.6
Re-*Z*-**TS**-**12**	-2855.39487	2.2	1.7	0.5

Supplementary Table 20. *Electronic energies and distortion / interaction analysis for constrained TS models of system* ***15****, calculated at the SMD(DME)-B2GP-PLYP-D3BJ/def2-TZVPP//SMD(DME)-PBE0-D3BJ/def2-SVP level of theory. The forming bond lengths were constrained to their lengths in Supplementary Table 8. Energies are reported in kcal mol^{-1}.*

TS model	E_{el}	$\Delta\Delta E_{el}$	$\Delta\Delta E_{dist}$	$\Delta\Delta E_{int}$
Si-*E*-**TS**-**15**	-3051.81976	0.0	0.0	0.0
Re-*Z*-**TS**-**15**	-3051.81430	3.4	4.3	-0.9

5. Photophysical experiments

5.1 Stern-Volmer quenching experiments

Emission spectra were recorded at 20 ºC using an Edinburgh Instruments FS5 spectrofluorimeter, equipped with a xenon arc lamp (400 nm excitation), an SC-20 thermostatic sample holder, and a Hamamatsu R13456 PMT detector measuring at 425 – 800 nm. A quartz cuvette (10 mm path length) was charged with 2.5 mL of a 5 μM solution of photocatalyst Ir[(ppy)$_2$(dtbbpy)]PF$_6$ dissolved in a 1:1 mixture of anhydrous DME/Et$_2$O and was degassed by sparging with argon for 10 minutes. The appropriate volume of a 0.5 M solution (1:1 DME/Et$_2$O) of each quencher was added sequentially and the emission of the solution was measured. The quenching constant of the enamine derived from 3-phenylpropanal and organocatalyst **9** was obtained from the measurement of a 15:1 solution of this aldehyde and organocatalyst in which the amine was assumed to be completely consumed. The quenching constant for this enamine was then calculated by adjustment for concentration and background aldehyde quenching, to obtain a value of 83.4 M^{-1}. These measurements show that the enamine is the most efficient quencher of Ir[(ppy)$_2$(dtbbpy)]PF$_6$ of the components in the reaction.

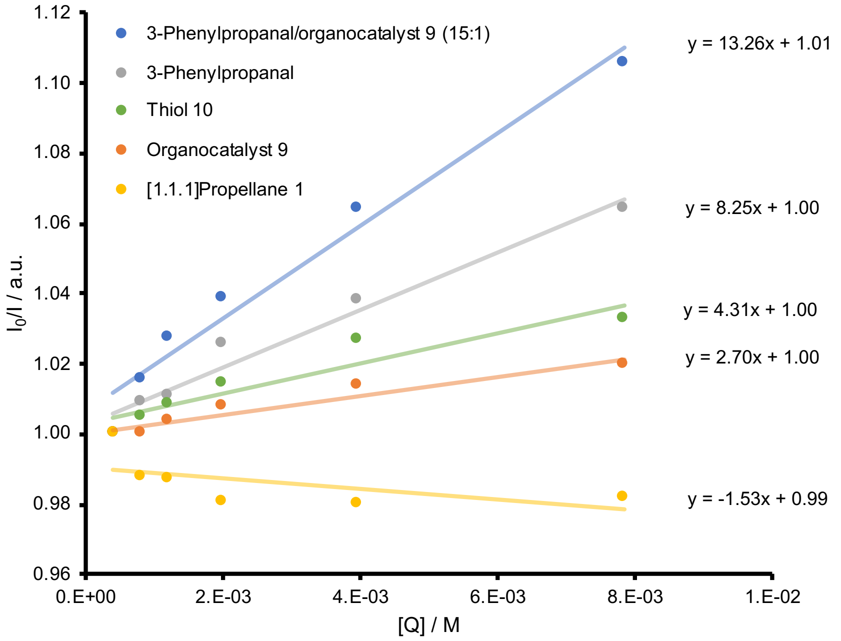

Supplementary Figure 14. *Stern-Volmer fluorescence quenching plot for each component in the reaction, for a 5 μM solution of Ir[(ppy)$_2$(dtbbpy)]PF$_6$.*

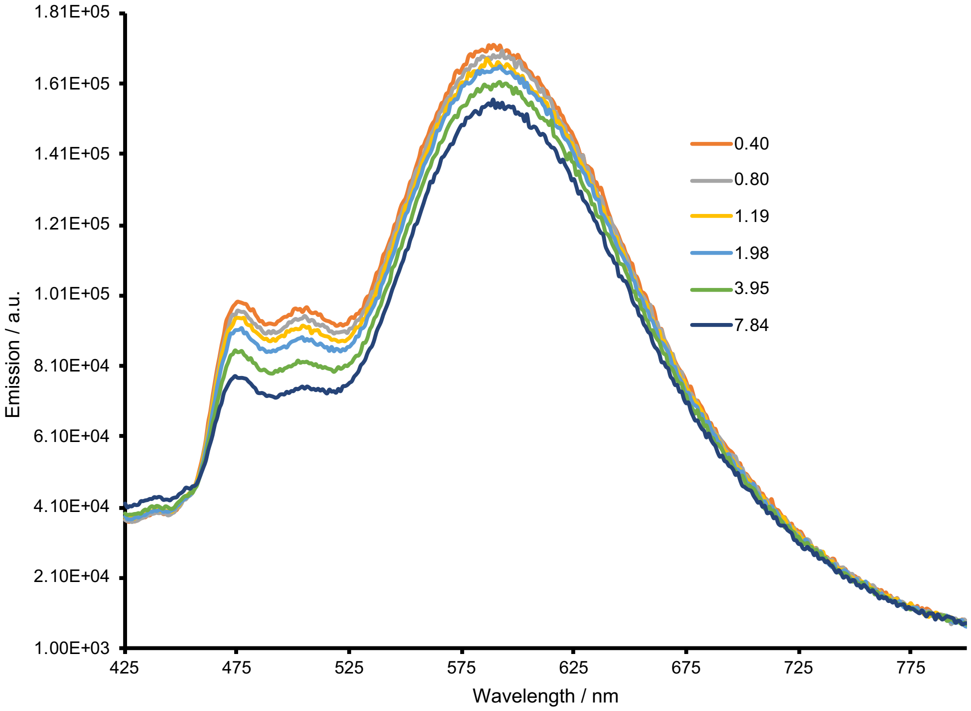

Supplementary Figure 15. *Fluorescence quenching titration for a 15:1 solution of 3-phenylpropanal and organocatalyst* ***9****, with a 5 μM solution of Ir[(ppy)$_2$(dtbbpy)]PF$_6$. Quencher concentrations reported in mM.*

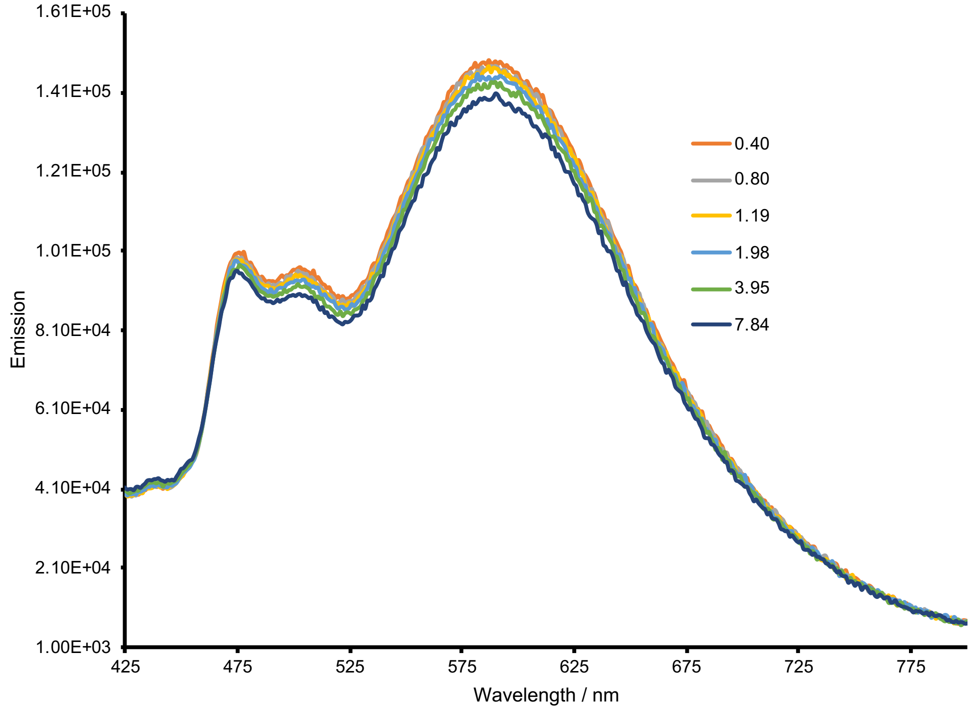

Supplementary Figure 16. *Fluorescence quenching titration for 3-phenylpropanal with a 5 μM solution of Ir[(ppy)$_2$(dtbbpy)]PF$_6$. Quencher concentrations reported in mM.*

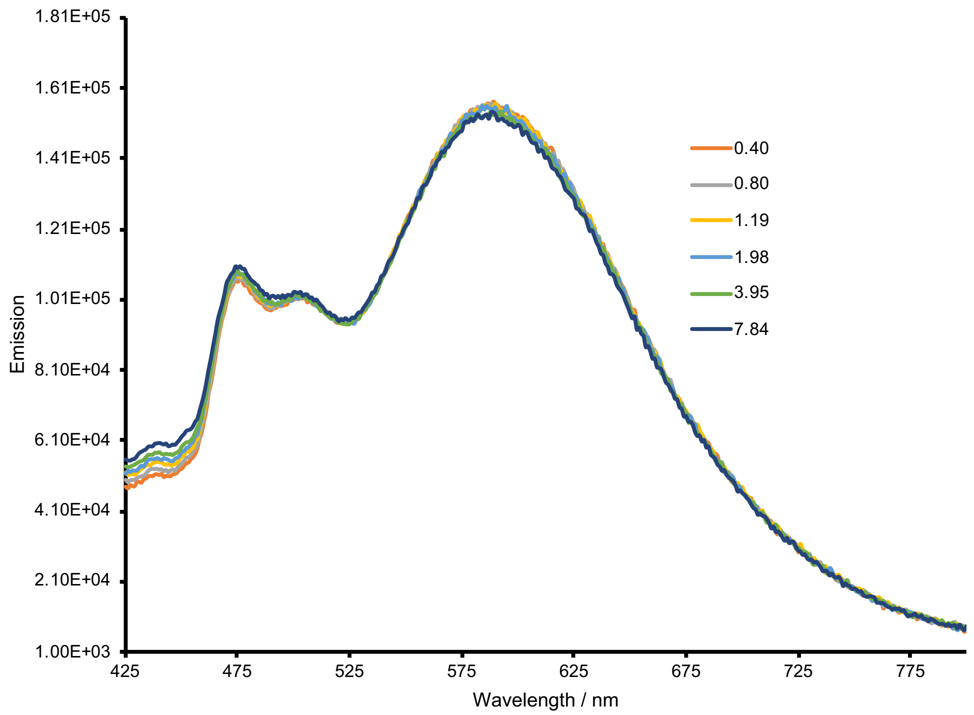

Supplementary Figure 17. *Fluorescence quenching titration for organocatalyst* ***9*** *with a 5 μM solution of Ir[(ppy)$_2$(dtbbpy)]PF$_6$. Quencher concentrations reported in mM.*

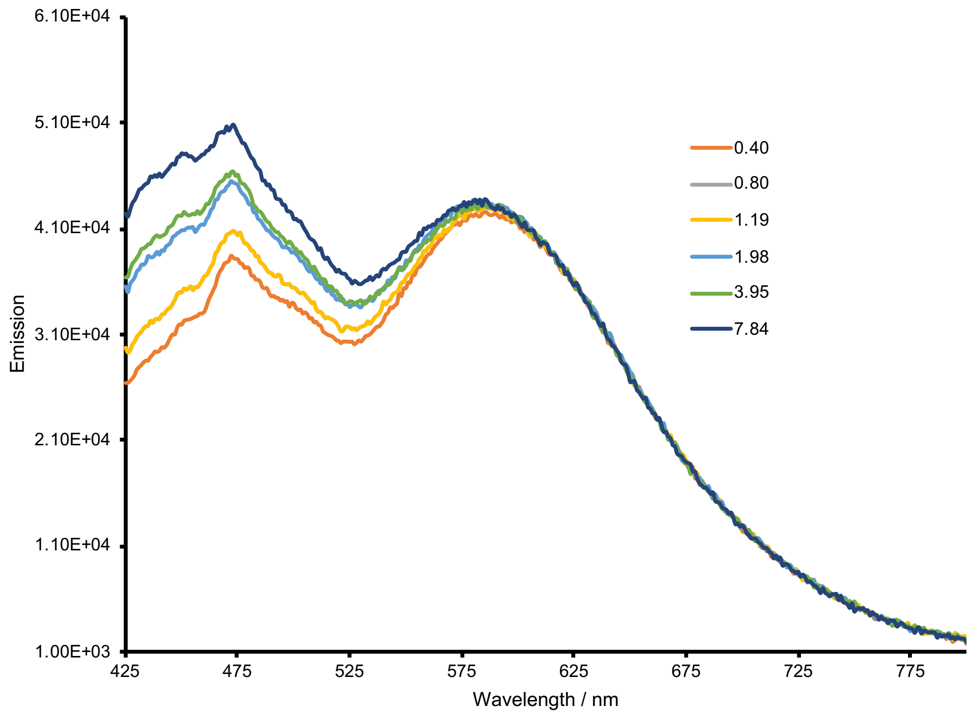

Supplementary Figure 18. *Fluorescence quenching titration for [1.1.1]propellane* ***1*** *with a 5 μM solution of Ir[(ppy)$_2$(dtbbpy)]PF$_6$. Quencher concentrations reported in mM. The increasing intensity of the 480 nm emission may indicate reaction of* ***1*** *with the photocatalyst.*

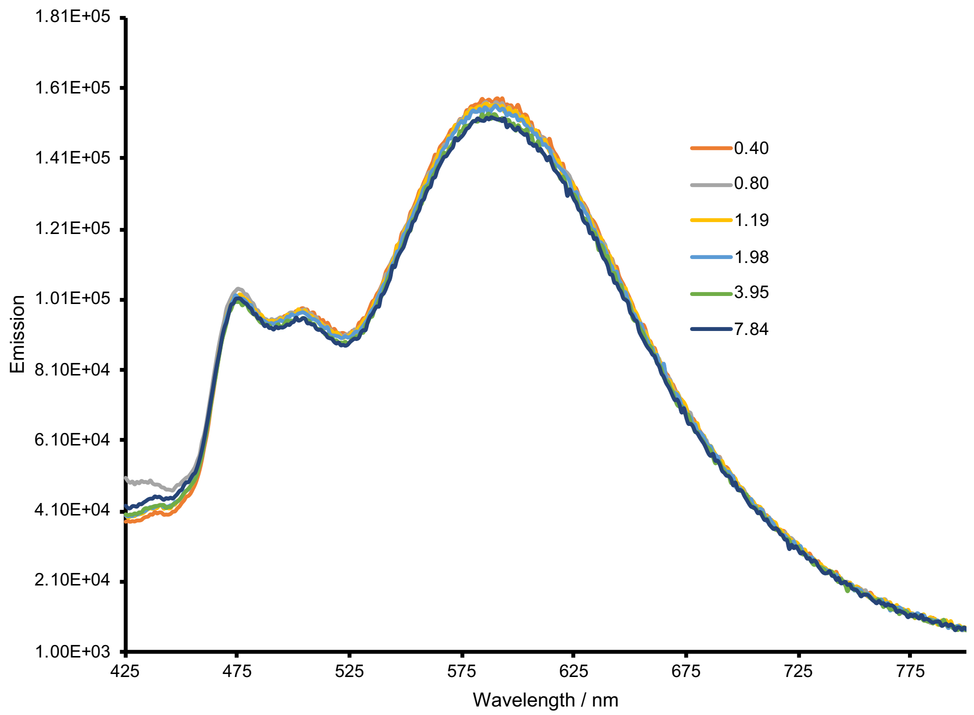

Supplementary Figure 19. *Fluorescence quenching titration for HAT catalyst* ***10*** *with a 5 μM solution of Ir[(ppy)$_2$(dtbbpy)]PF$_6$. Quencher concentrations reported in mM.*

5.2 UV/Vis absorption experiments

UV-vis absorption spectra were measured using a Perkin Elmer Lambda 20 spectrometer at 20 ºC (temperature controlled by a Perkin Elmer PTP-1 Peltier). The absorption spectrum of a 0.25 mM 15:1 3-phenylpropanal/organocatalyst **9** solution in a 1:1 mixture of anhydrous DME/Et_2O was measured, and showed no absorption above 375 nm suggesting that direct photoexcitation of the enamine is not involved in the reaction mechanism.

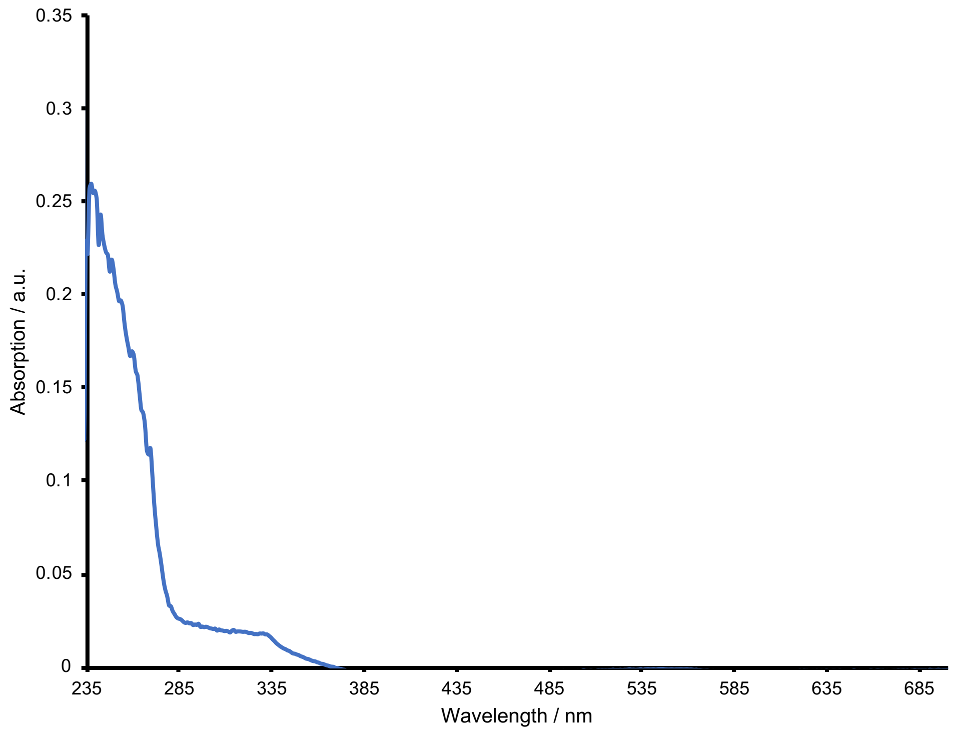

Supplementary Figure 20. *UV-vis absorption spectrum of a 15:1 solution of 3-phenylpropanal and organocatalyst* ***9****.*

6. NMR Spectra

(*R*)-2-(Bicyclo[1.1.1]pentan-1-yl)-3-methylbutan-1-ol, 13

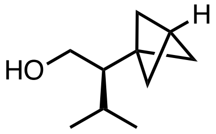

^{1}**H NMR** (400 MHz, $CDCl_3$)

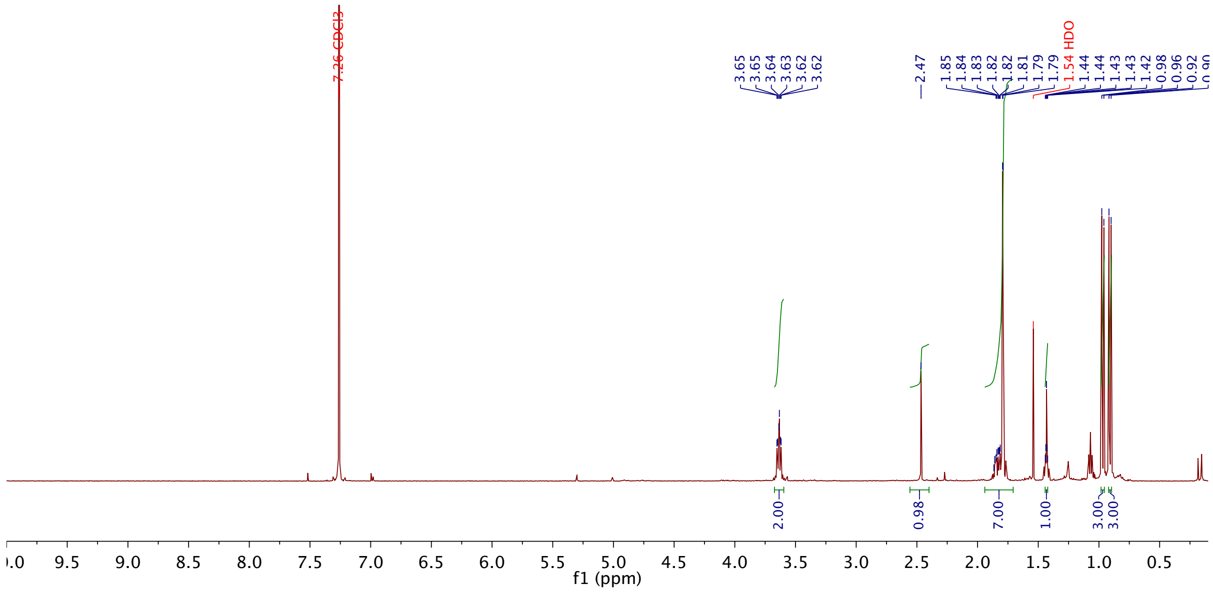

^{13}**C NMR** (126 MHz, $CDCl_3$)

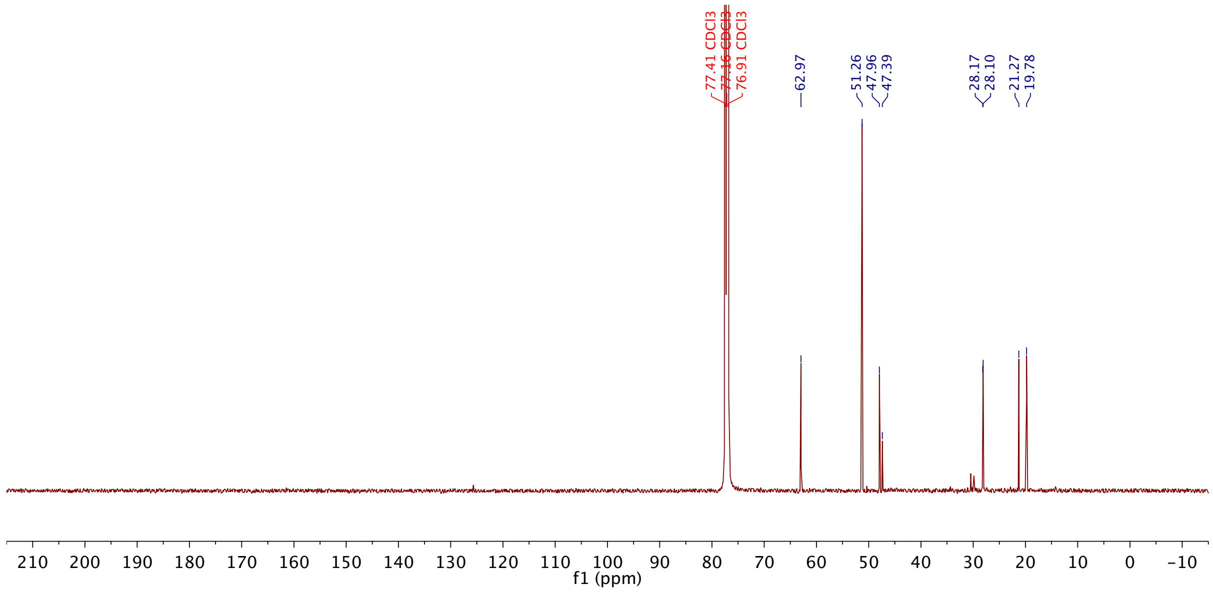

(*R*)-2-(Bicyclo[1.1.1]pentan-1-yl)-3,3-dimethylbutan-1-ol, 14

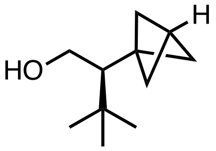

^{1}H NMR (400 MHz, $CDCl_3$)

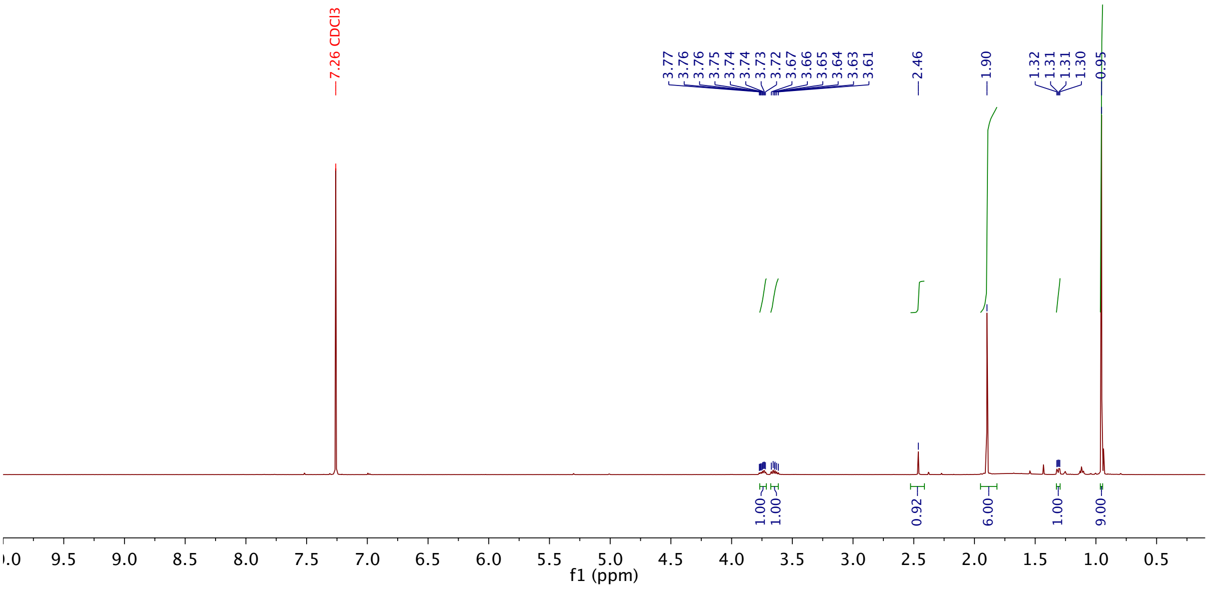

^{13}C NMR (126 MHz, $CDCl_3$)

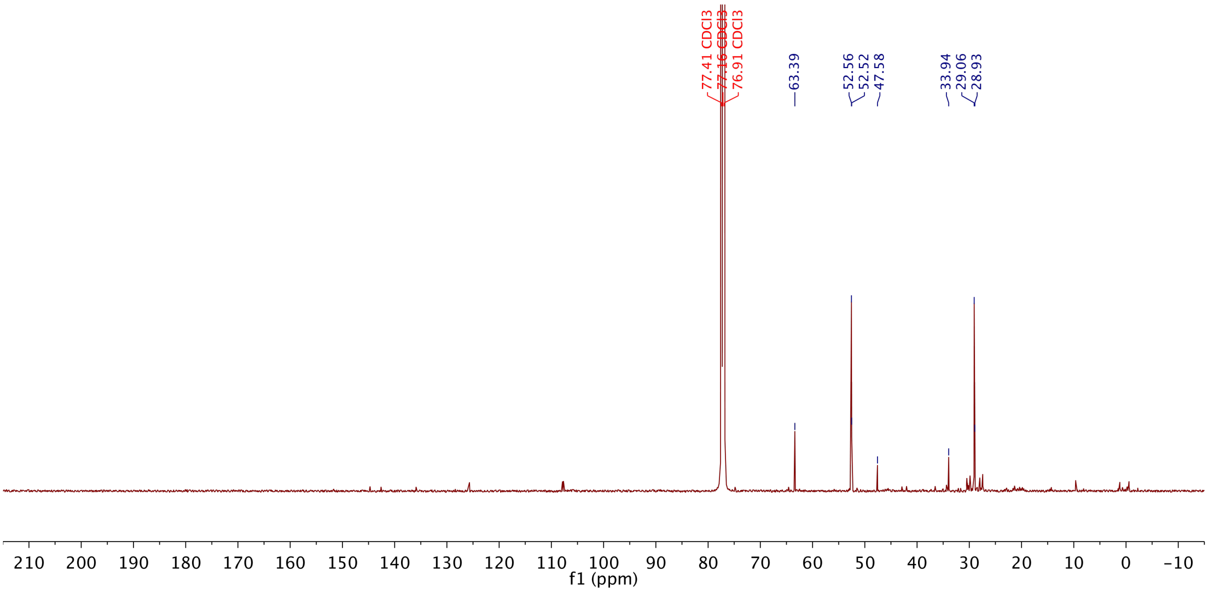

(*R*)-2-(Bicyclo[1.1.1]pentan-1-yl)octan-1-ol, 15

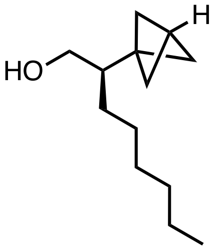

^{1}H NMR (500 MHz, $CDCl_3$)

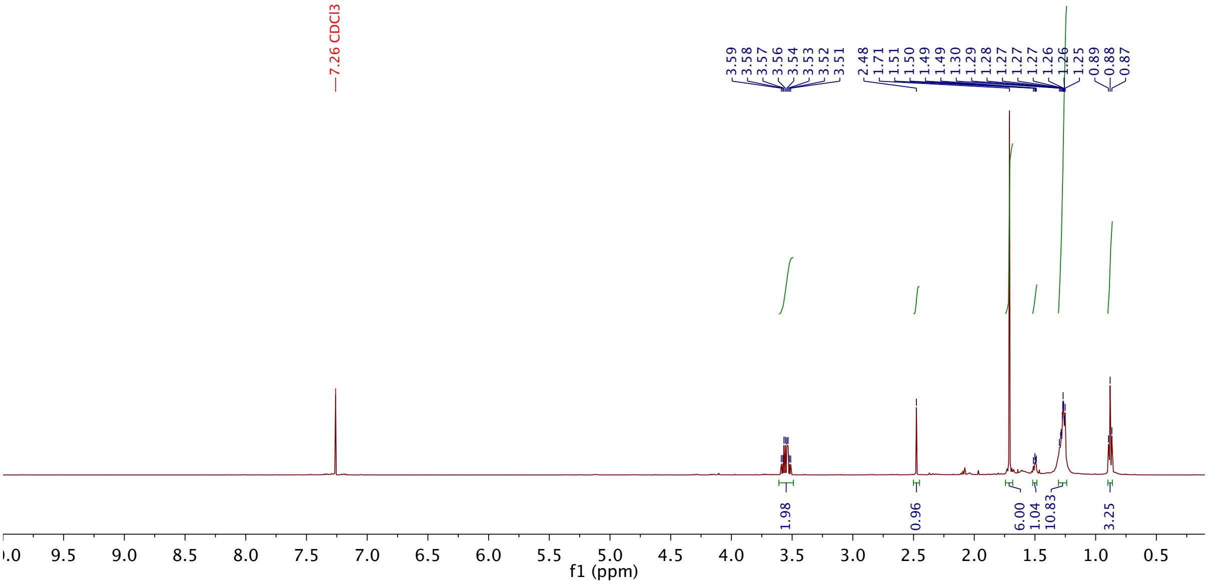

^{13}C NMR (126 MHz, $CDCl_3$)

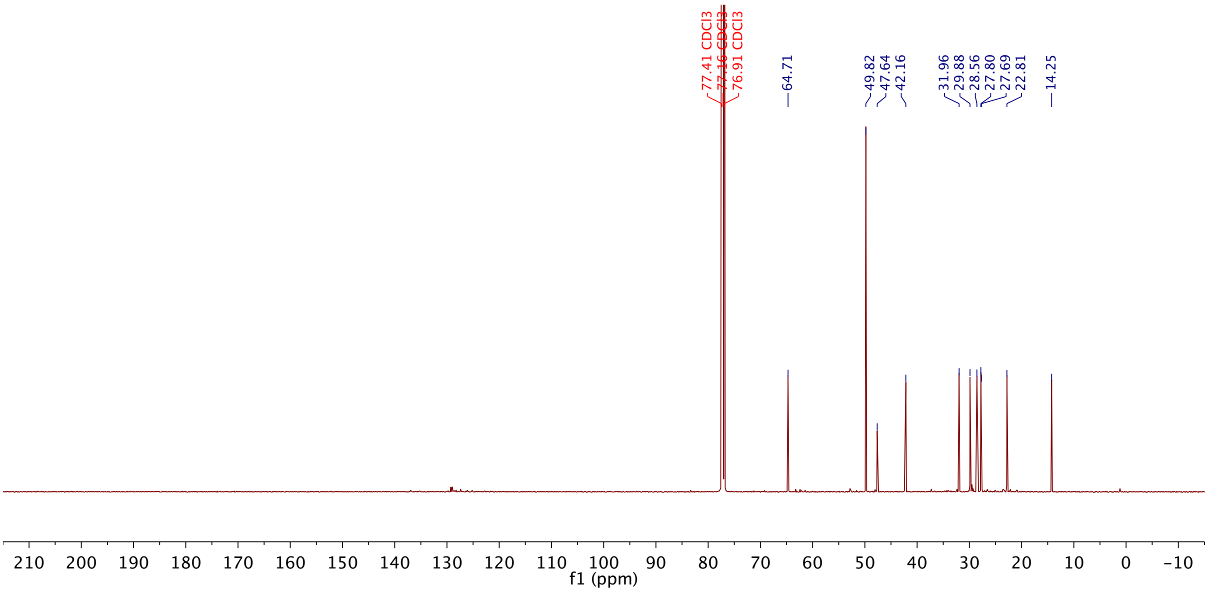

(*R*)-2-(Bicyclo[1.1.1]pentan-1-yl)-3-phenylpropan-1-ol, 16

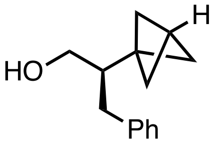

^{1}H NMR (500 MHz, $CDCl_3$)

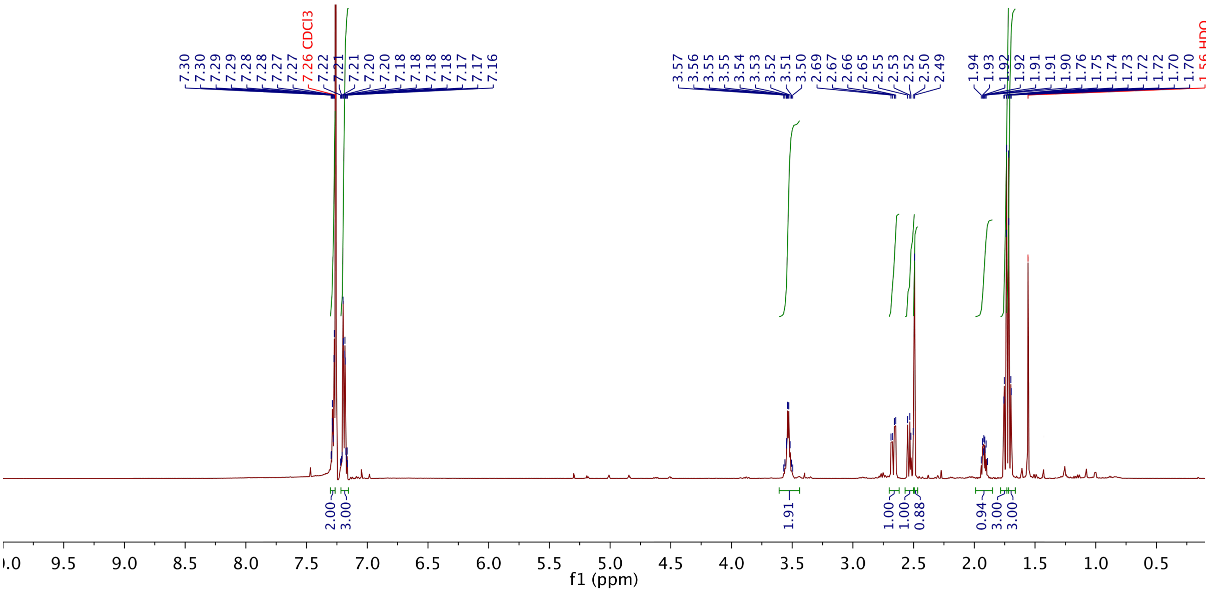

^{13}C NMR (126 MHz, $CDCl_3$)

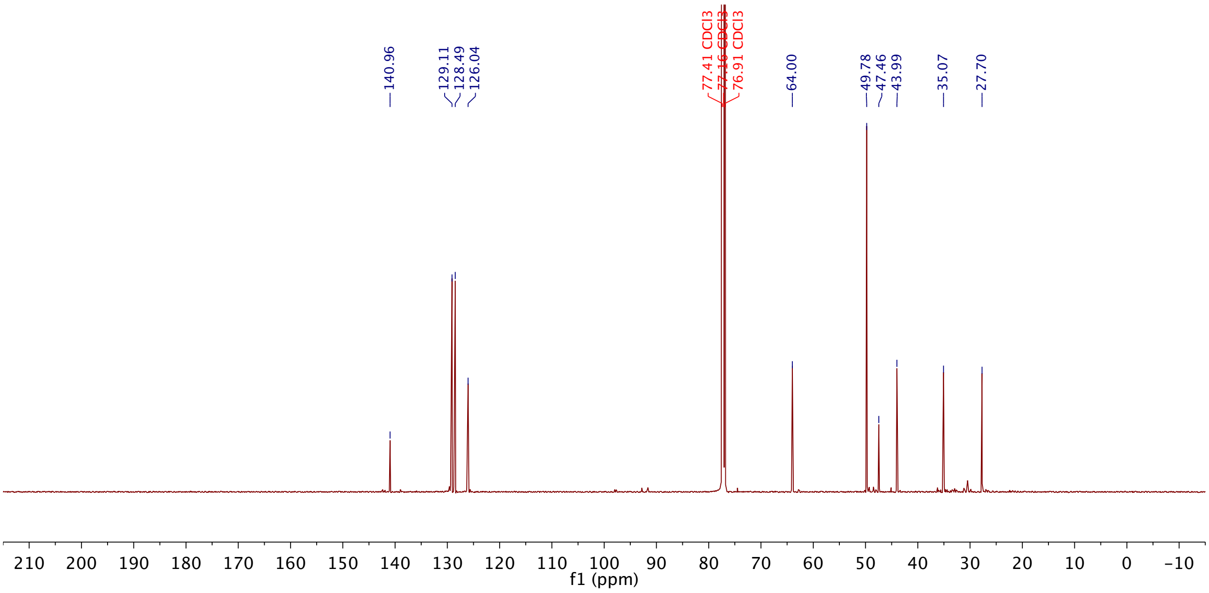

(*R*)-2-(Bicyclo[1.1.1]pentan-1-yl)-3-(4-methoxyphenyl)propan-1-ol, 17

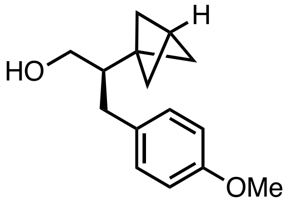

^{1}H NMR (500 MHz, $CDCl_3$)

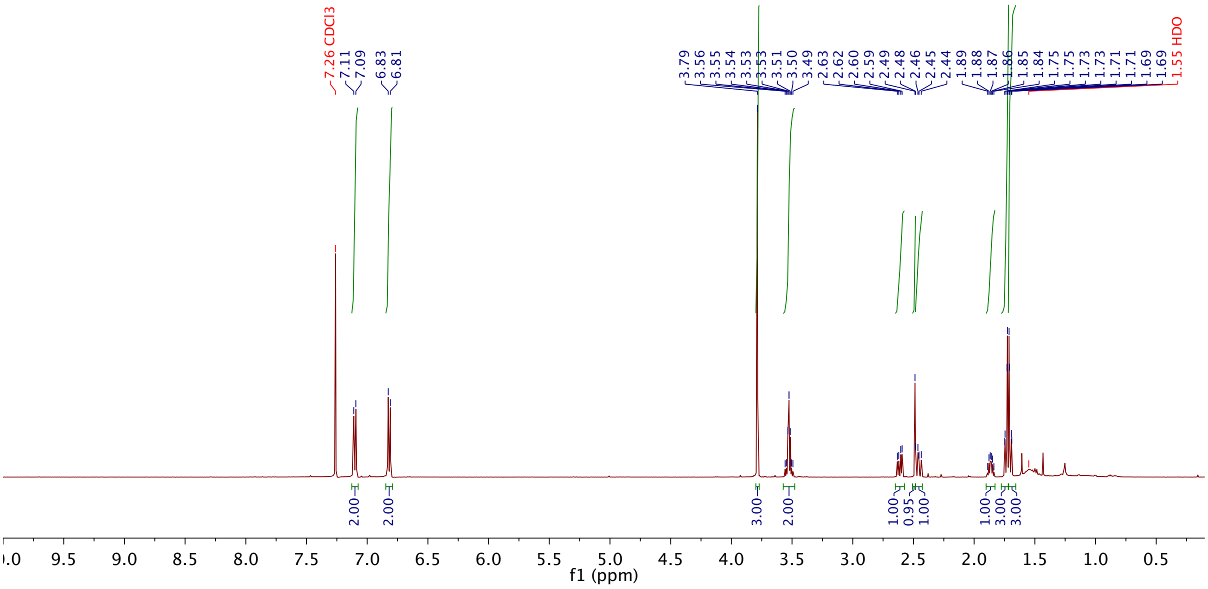

^{13}C NMR (126 MHz, $CDCl_3$)

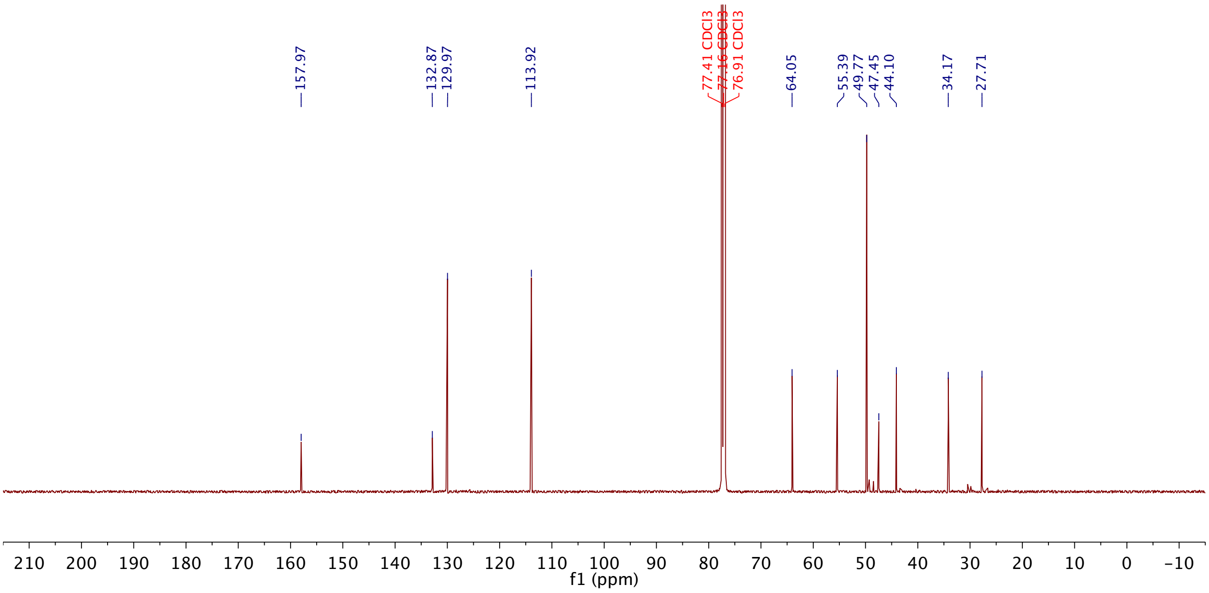

(*R*)-2-(Bicyclo[1.1.1]pentan-1-yl)-3-(4-(trifluoromethyl)phenyl)propan-1-ol, 18

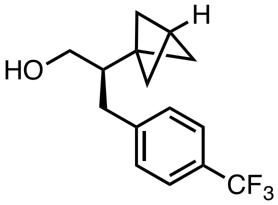

^{1}H NMR (500 MHz, $CDCl_3$)

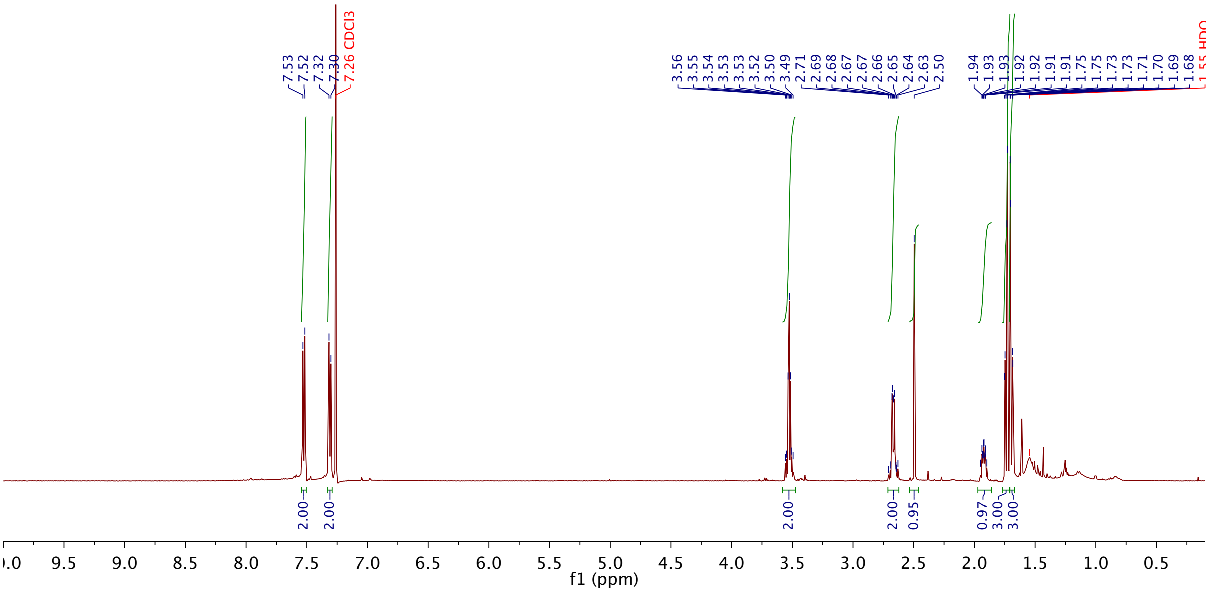

^{13}C NMR (126 MHz, $CDCl_3$)

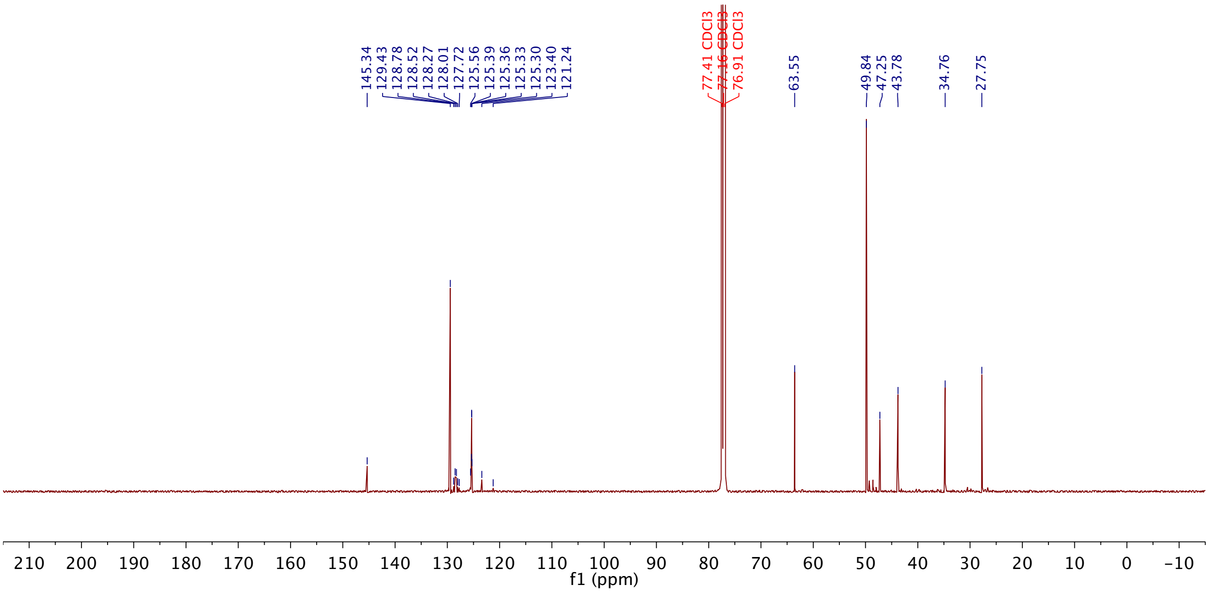

^{19}F NMR (471 MHz, $CDCl_3$)

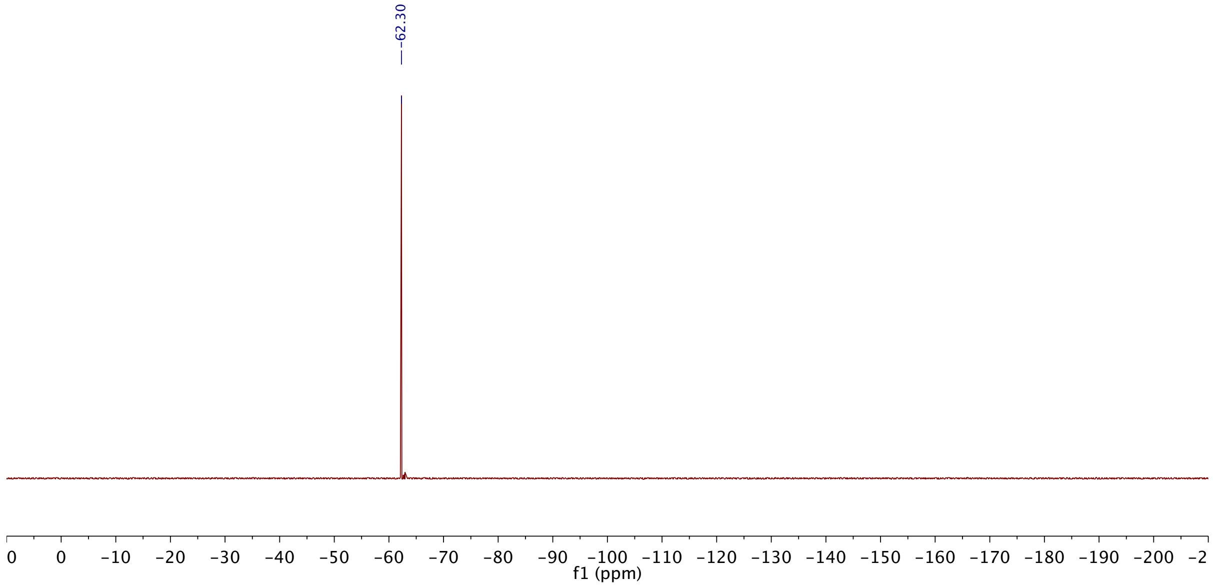

(*R*,*Z*)-2-(Bicyclo[1.1.1]pentan-1-yl)hept-4-en-1-ol, 19

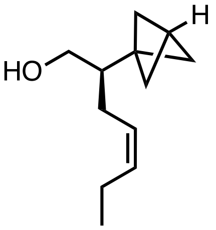

^{1}H NMR (500 MHz, $CDCl_3$)

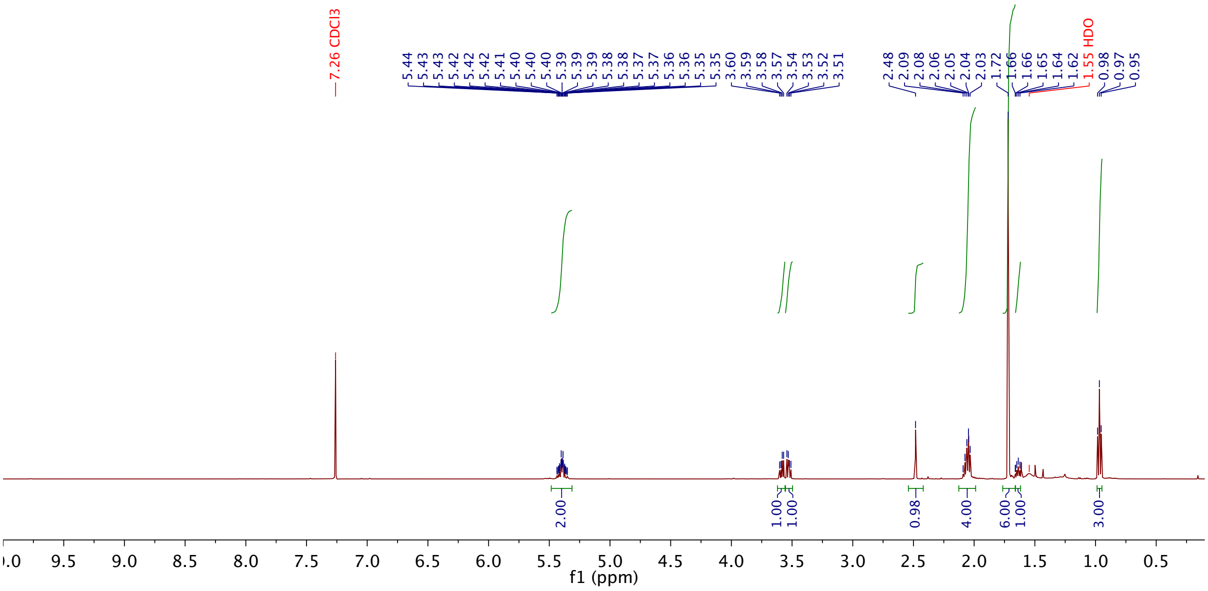

^{13}C NMR (126 MHz, $CDCl_3$)

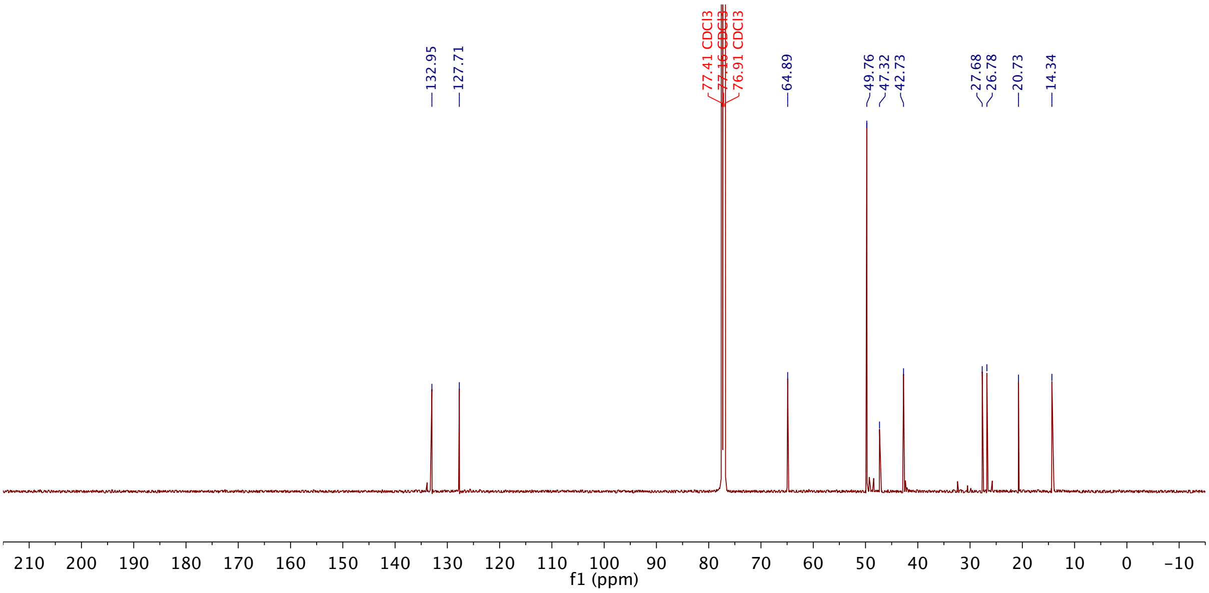

(*R*)-2-(Bicyclo[1.1.1]pentan-1-yl)-5-phenylpent-4-yn-1-ol, 20

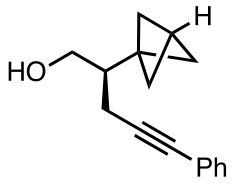

^{1}H NMR (400 MHz, $CDCl_3$)

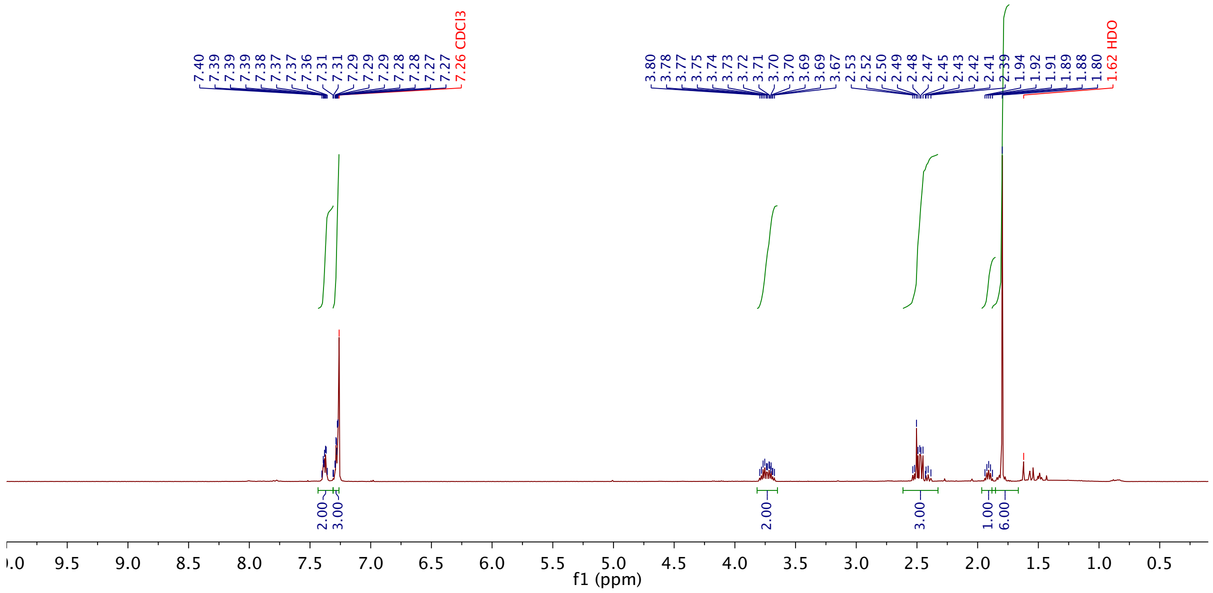

^{13}C NMR (126 MHz, $CDCl_3$)

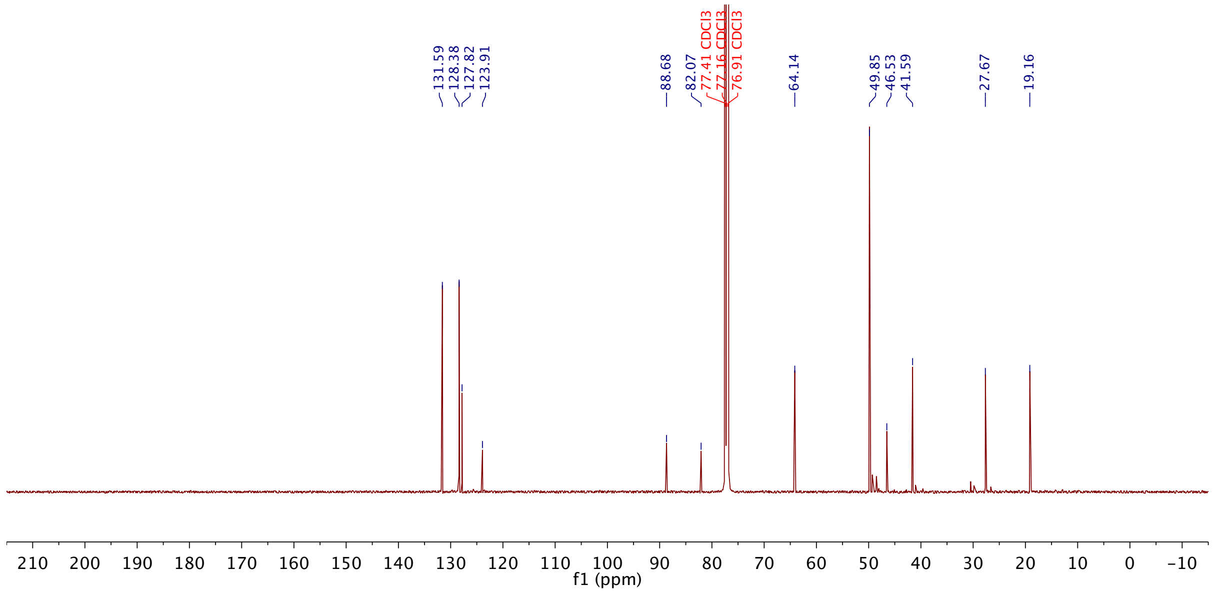

(*R*)-2-(Bicyclo[1.1.1]pentan-1-yl)-3-(methylthio)propan-1-ol, 21

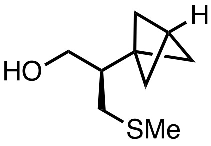

^{1}H NMR (400 MHz, $CDCl_3$)

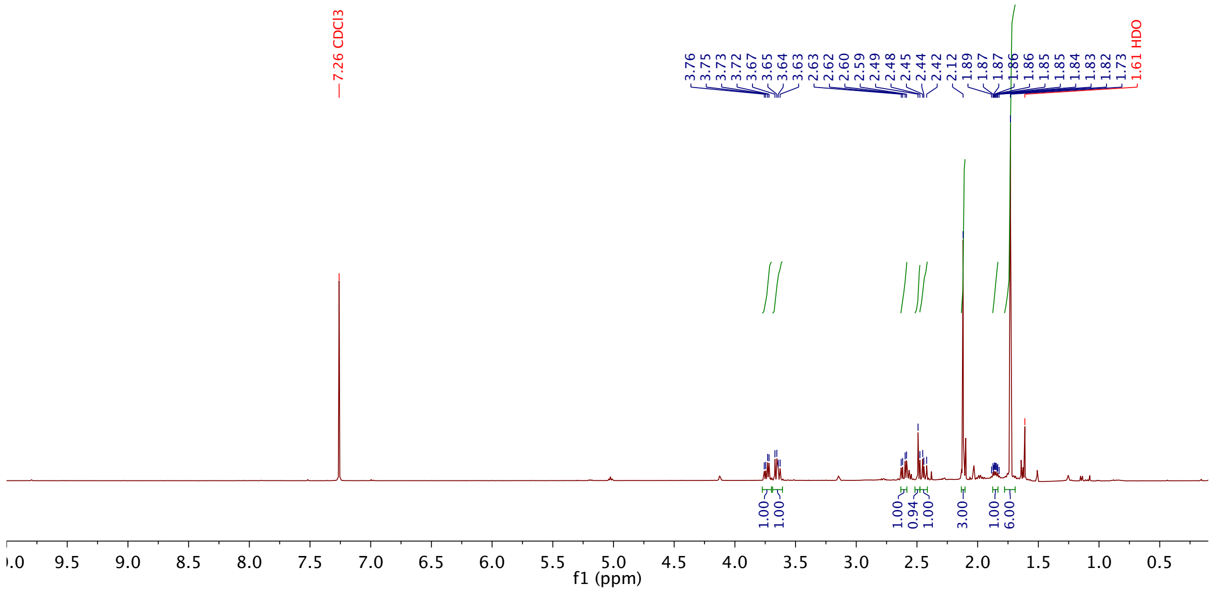

^{13}C NMR (126 MHz, $CDCl_3$)

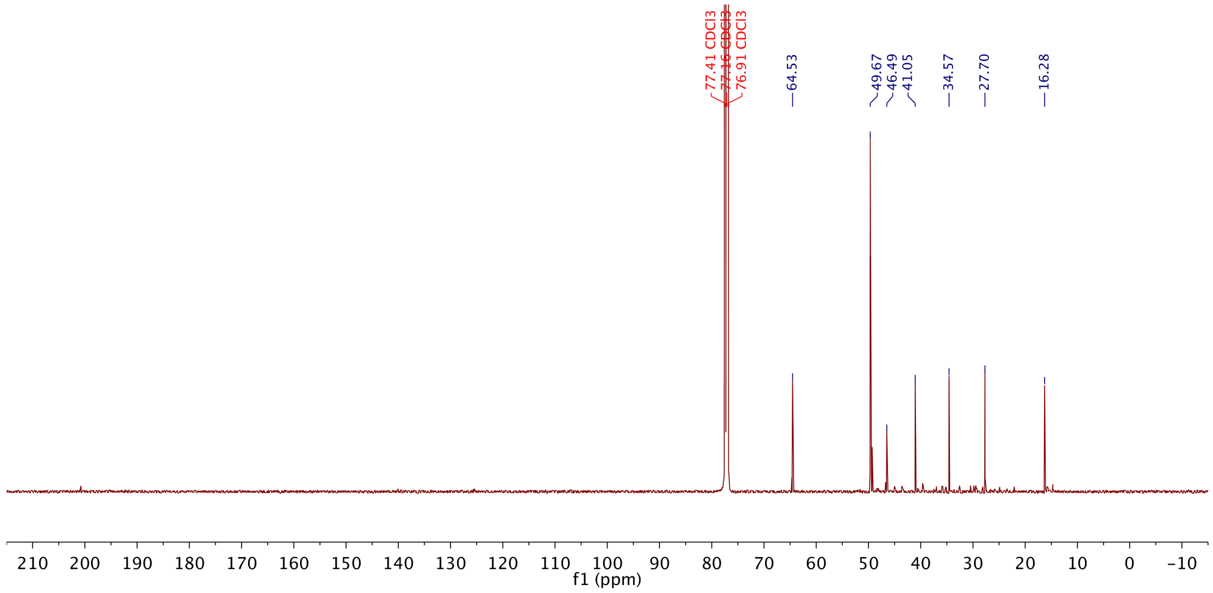

Benzyl (*S*)-(2-(bicyclo[1.1.1]pentan-1-yl)-3-hydroxypropyl)carbamate, 11

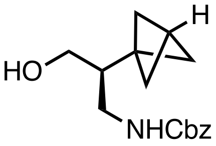

^{1}H NMR (400 MHz, $CDCl_3$)

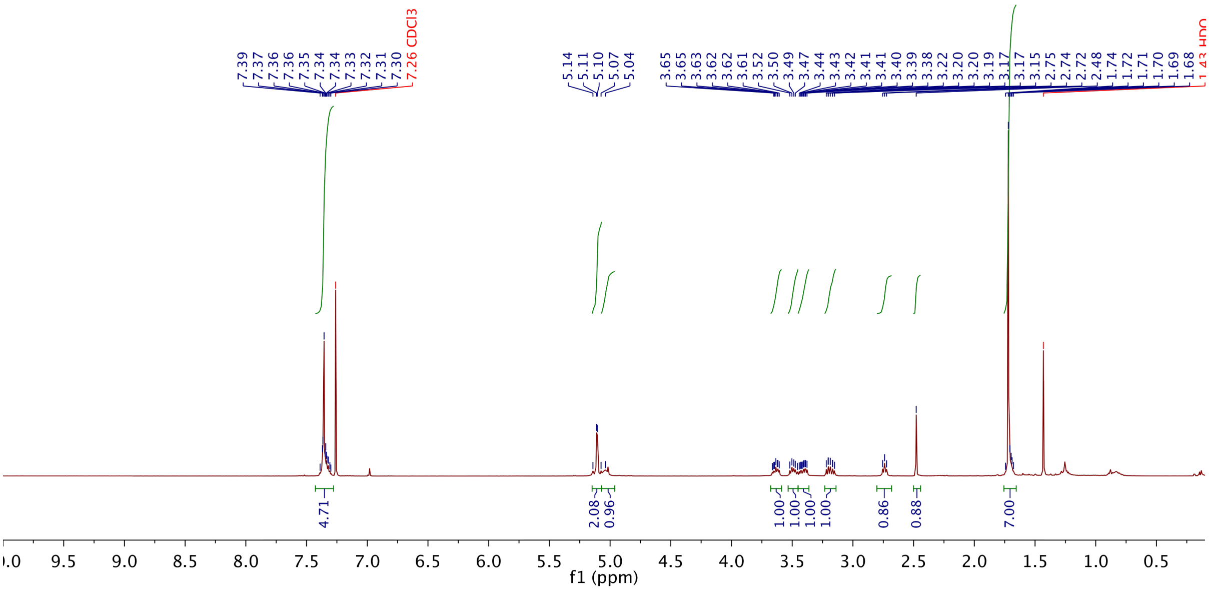

^{13}C NMR (126 MHz, $CDCl_3$)

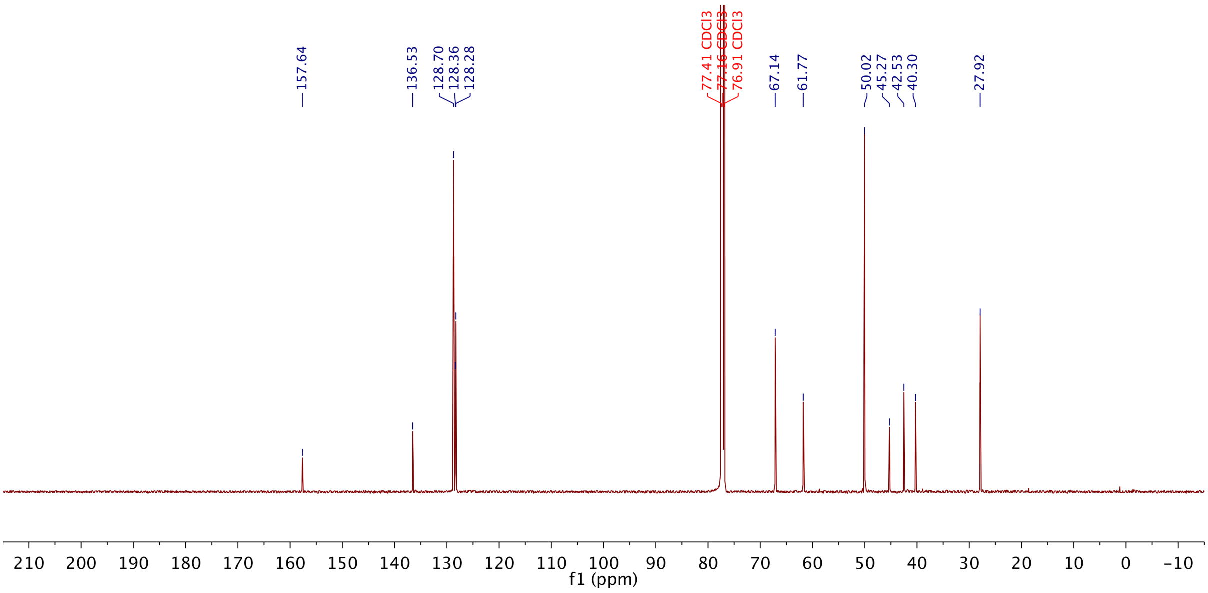

(*R*)-2-(Bicyclo[1.1.1]pentan-1-yl)-6-chlorohexan-1-ol, 22

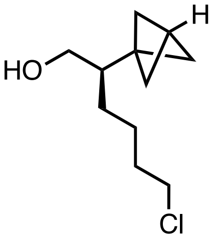

^{1}H NMR (500 MHz, $CDCl_3$)

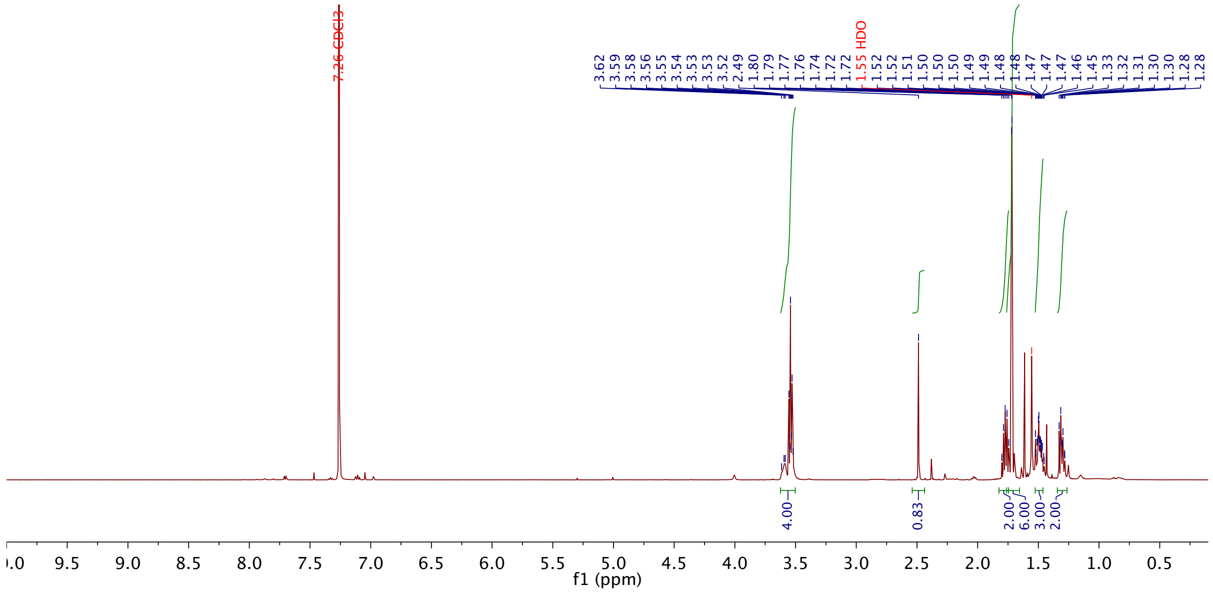

^{13}C NMR (126 MHz, $CDCl_3$)

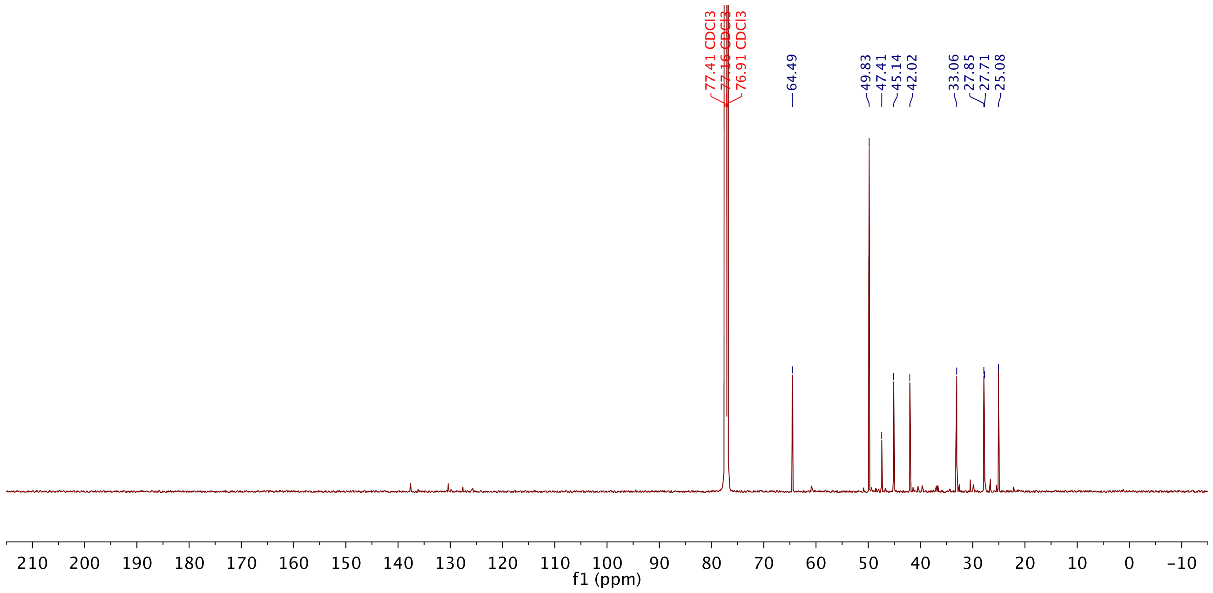

Ethyl (*R*)-5-(bicyclo[1.1.1]pentan-1-yl)-6-hydroxyhexanoate, 23

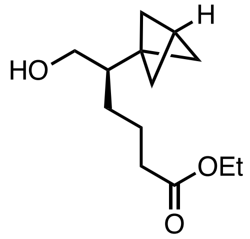

^{1}H NMR (500 MHz, $CDCl_3$)

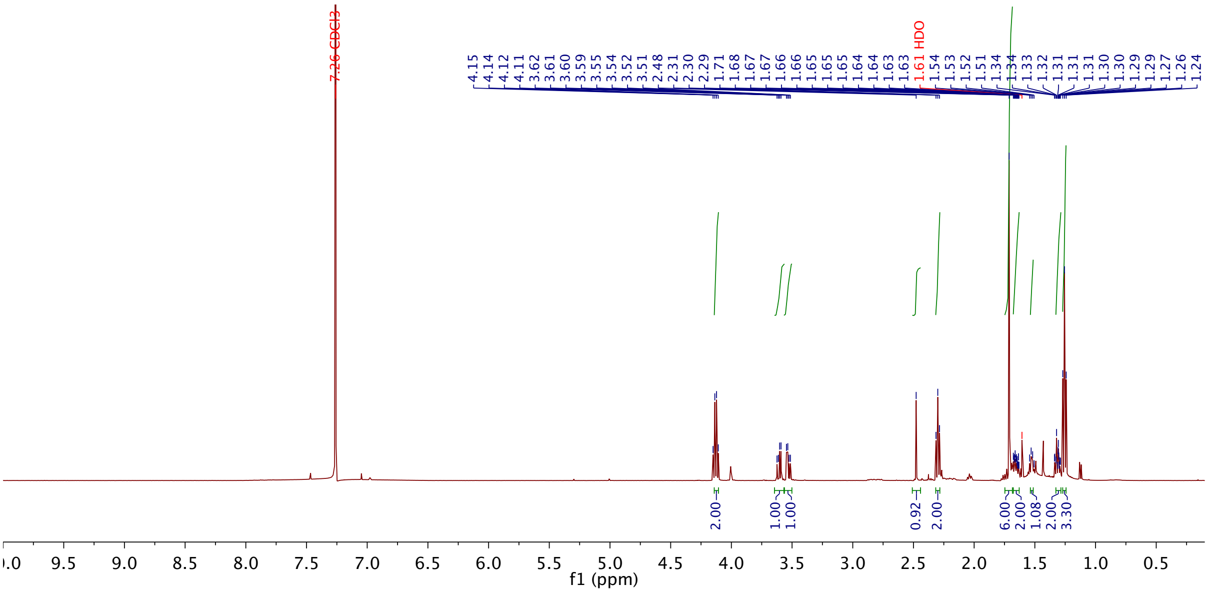

^{13}C NMR (126 MHz, $CDCl_3$)

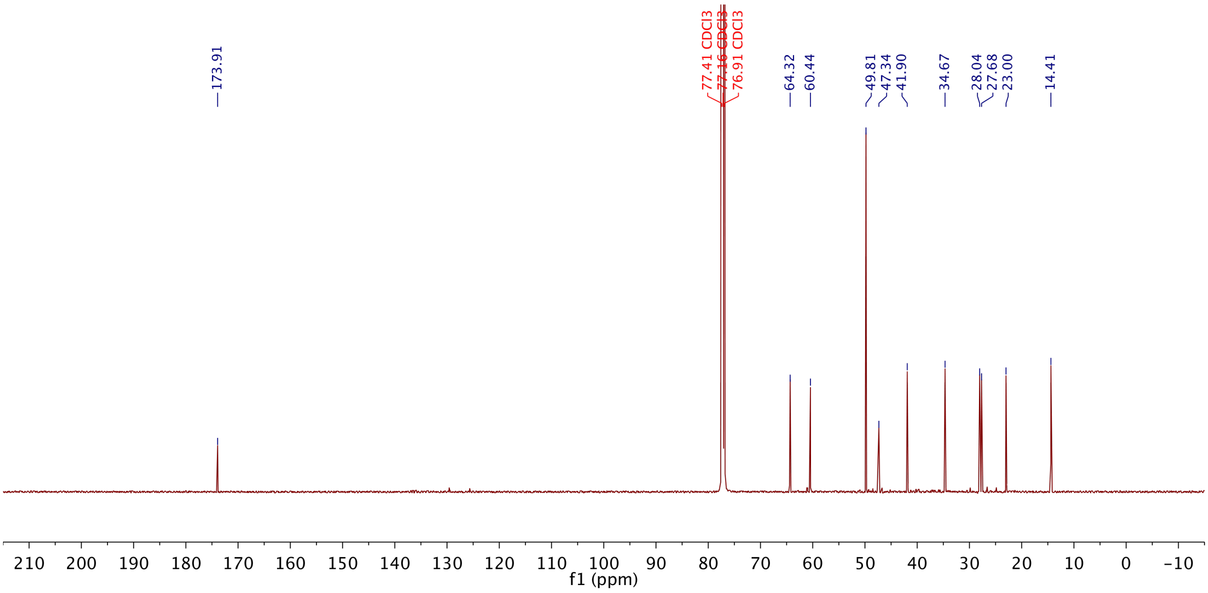

(*R*)-5-(Benzyloxy)-2-(bicyclo[1.1.1]pentan-1-yl)pentan-1-ol, 24

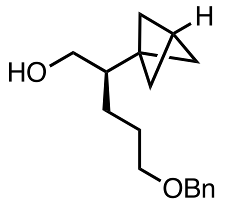

^{1}H NMR (500 MHz, $CDCl_3$)

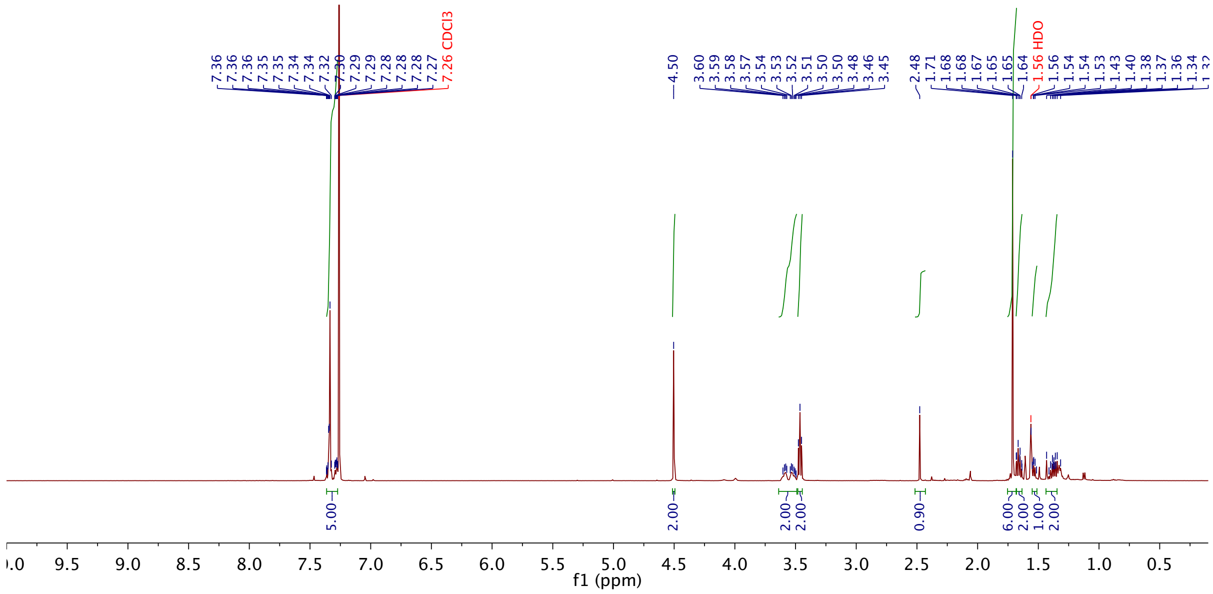

^{13}C NMR (126 MHz, $CDCl_3$)

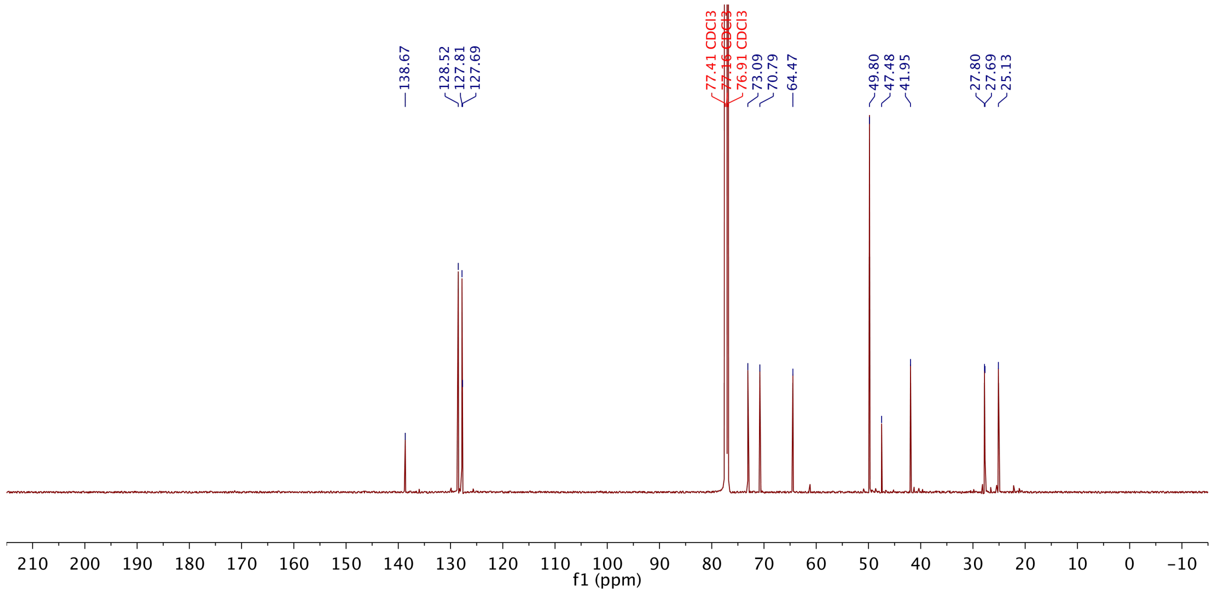

***tert*-Butyl (*R*)-4-(1-(bicyclo[1.1.1]pentan-1-yl)-2-hydroxyethyl)piperidine-1-carboxylate, 25**

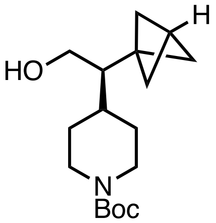

^{1}H NMR (500 MHz, $CDCl_3$)

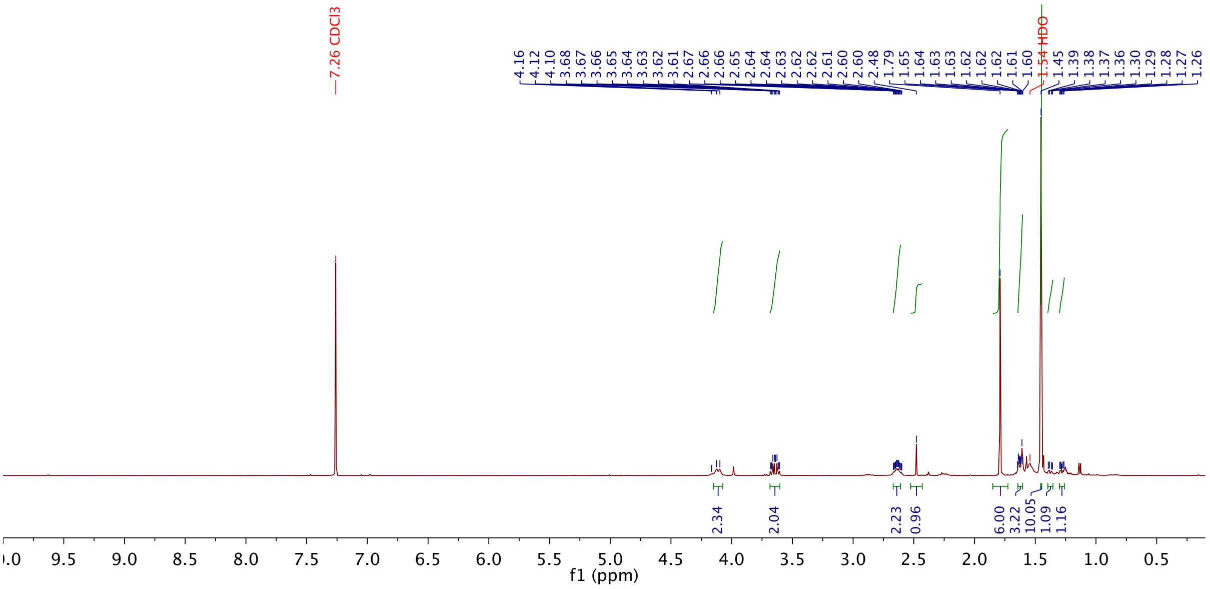

^{13}C NMR (126 MHz, $CDCl_3$)

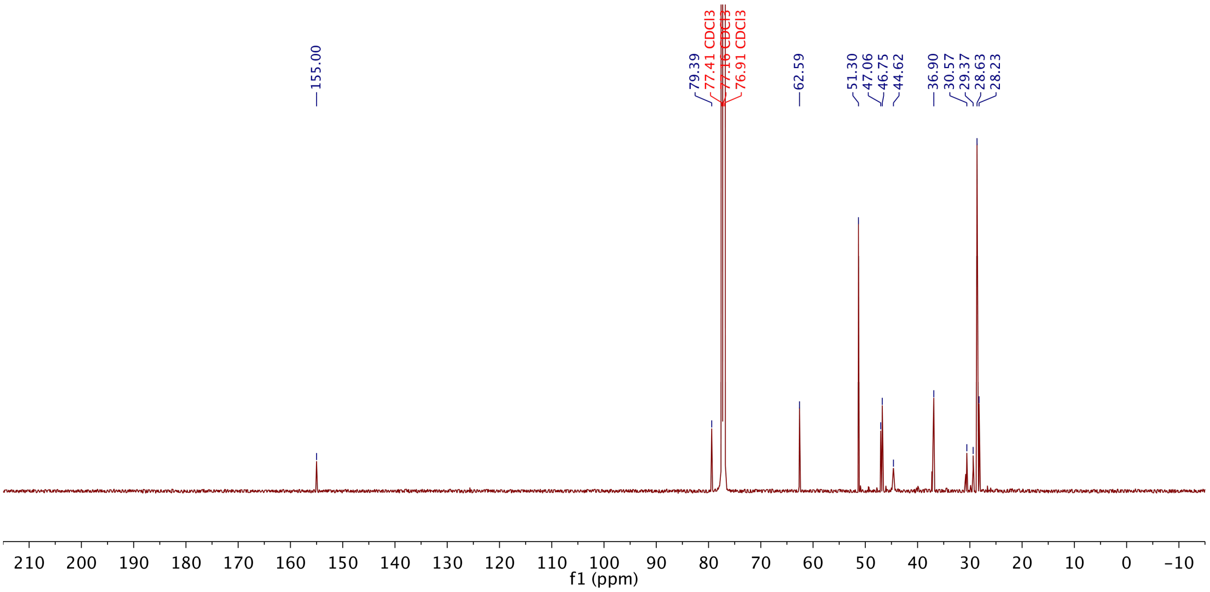

(*R*)-2-(Bicyclo[1.1.1]pentan-1-yl)-4-(5,5-dimethyl-1,3-dioxan-2-yl)butan-1-ol, 26

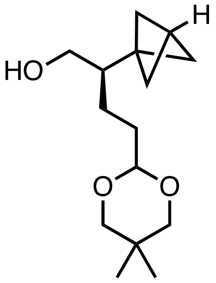

^{1}H NMR (400 MHz, $CDCl_3$)

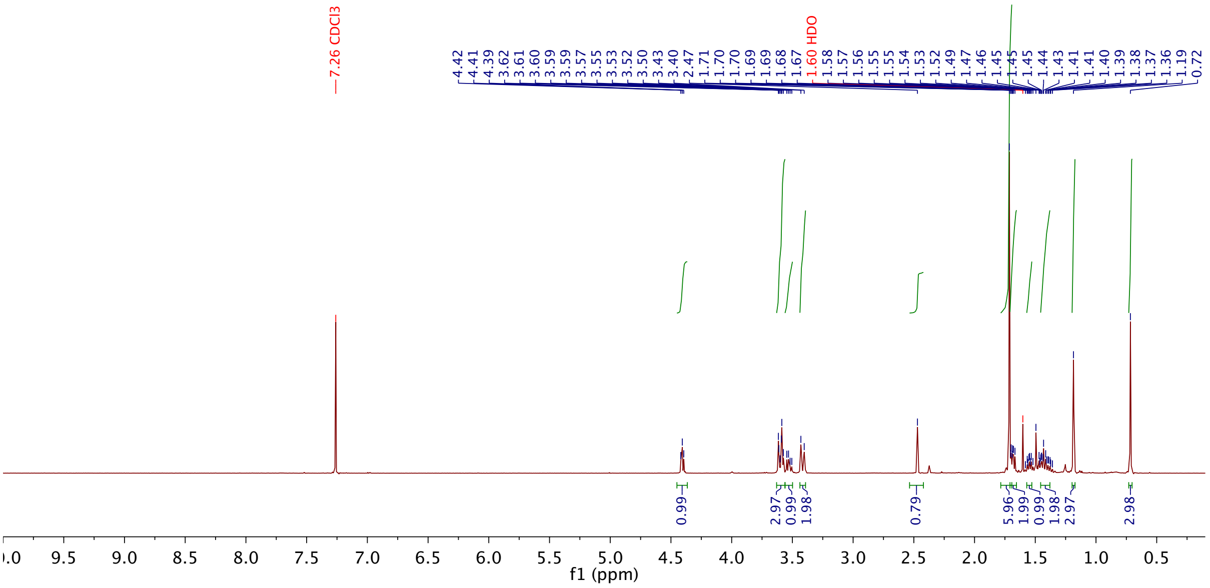

^{13}C NMR (126 MHz, $CDCl_3$)

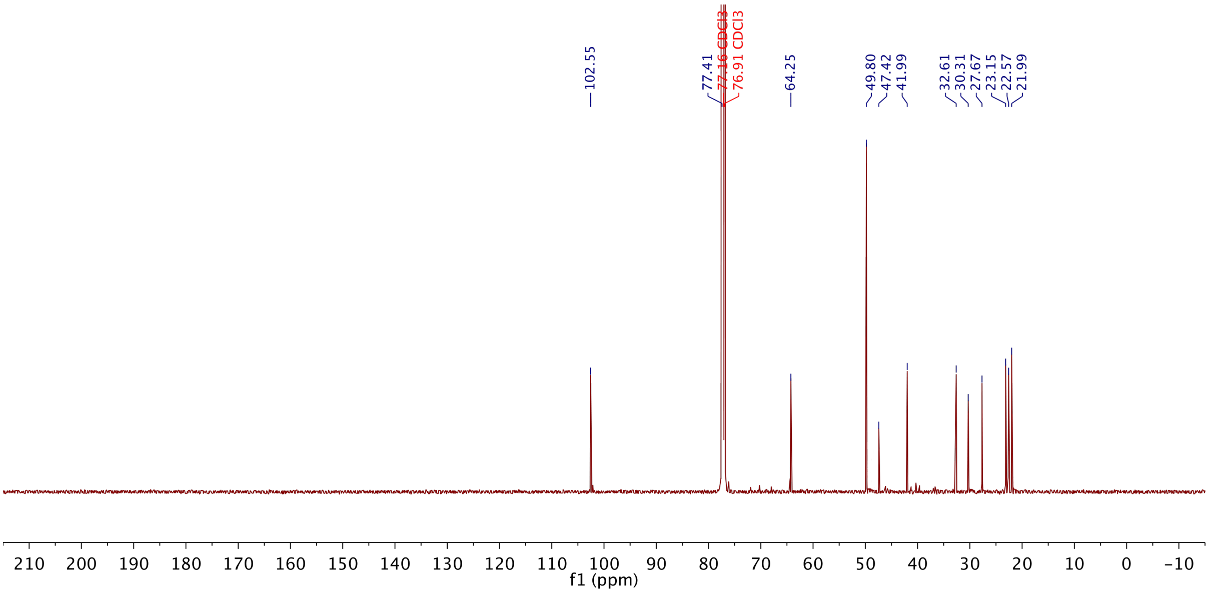

(*R*)-2-(Bicyclo[1.1.1]pentan-1-yl)-3-(5-methylfuran-2-yl)propan-1-ol, 27

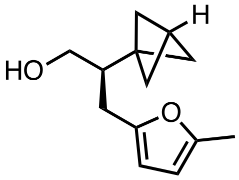

^{1}H NMR (400 MHz, $CDCl_3$)

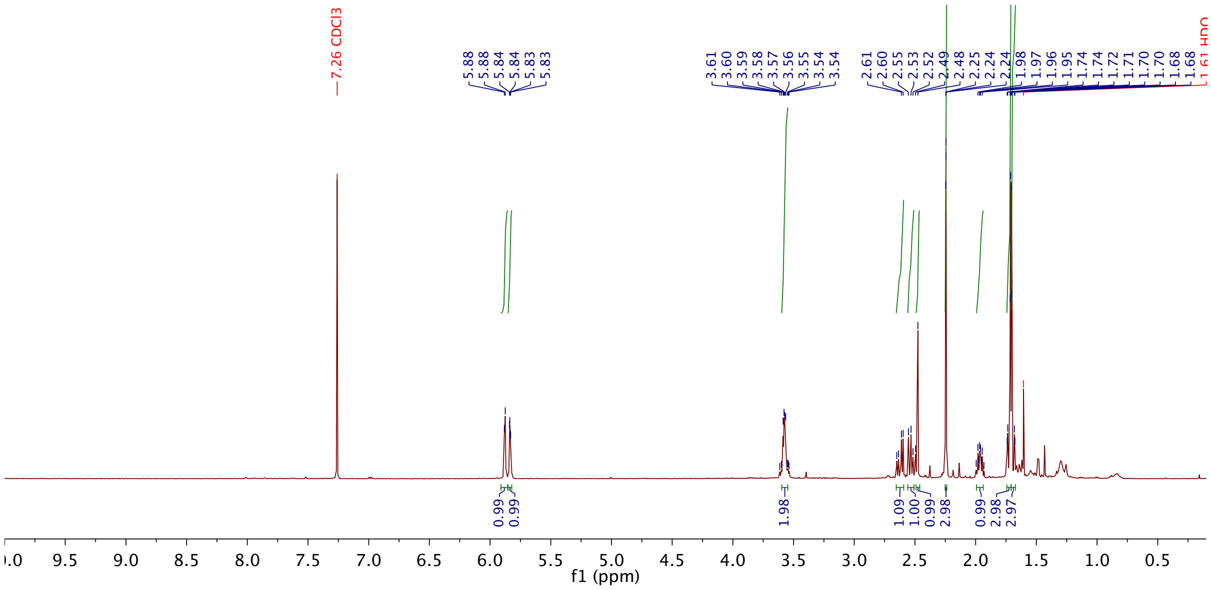

^{13}C NMR (126 MHz, $CDCl_3$)

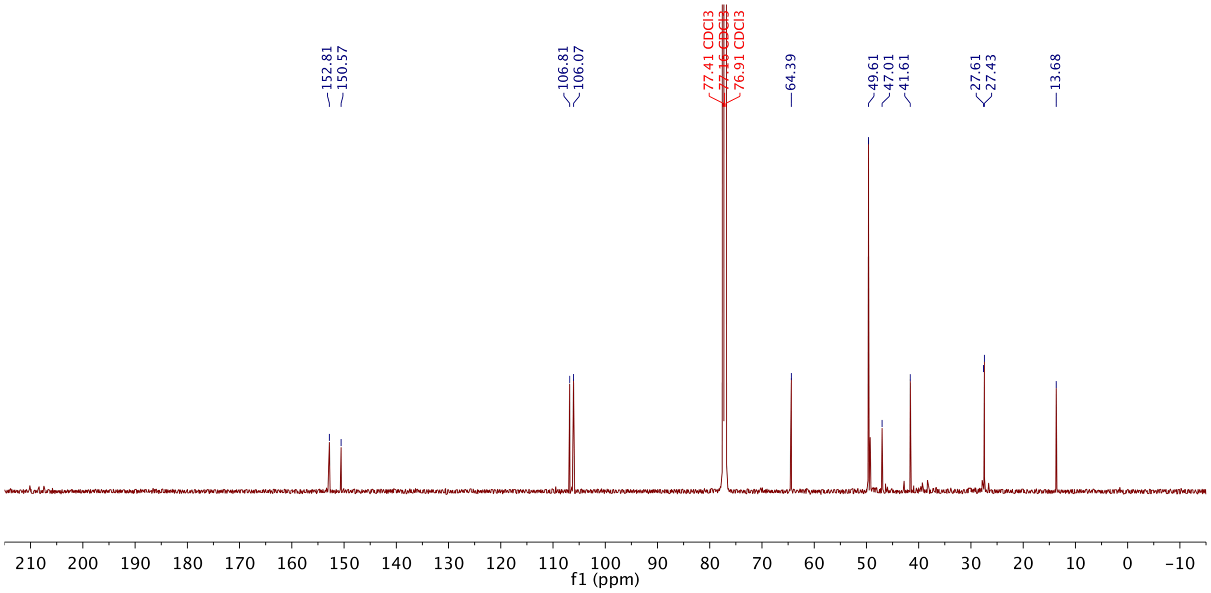

(*R*)-2-(Bicyclo[1.1.1]pentan-1-yl)-3-(thiophen-2-yl)propan-1-ol, 28

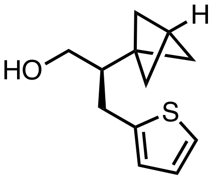

^{1}H NMR (500 MHz, $CDCl_3$)

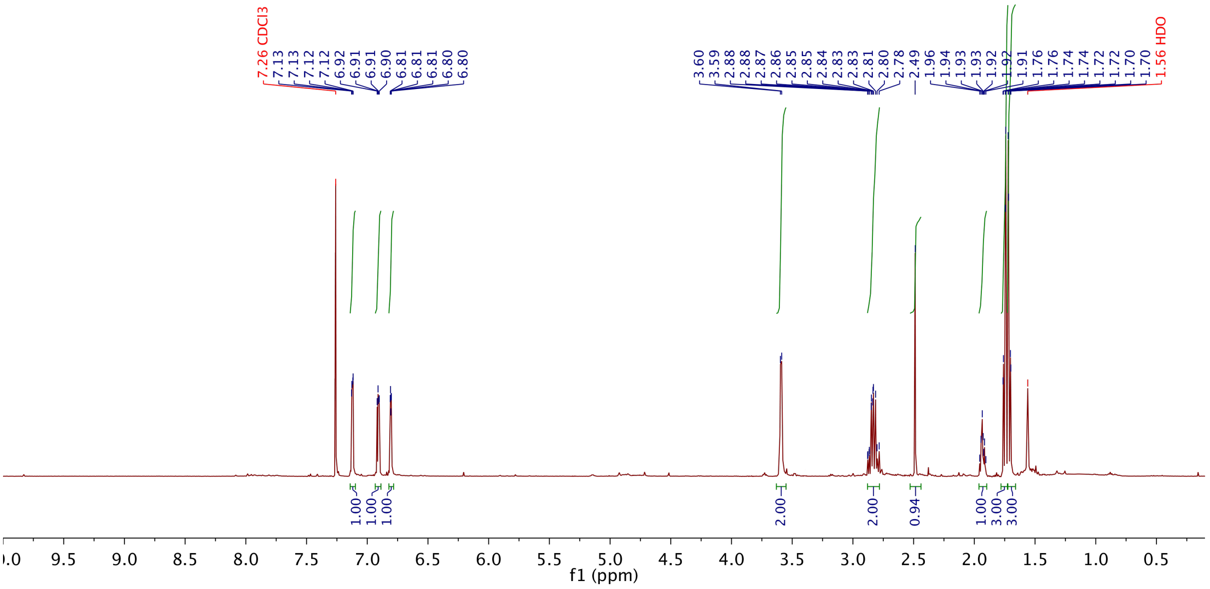

^{13}C NMR (126 MHz, $CDCl_3$)

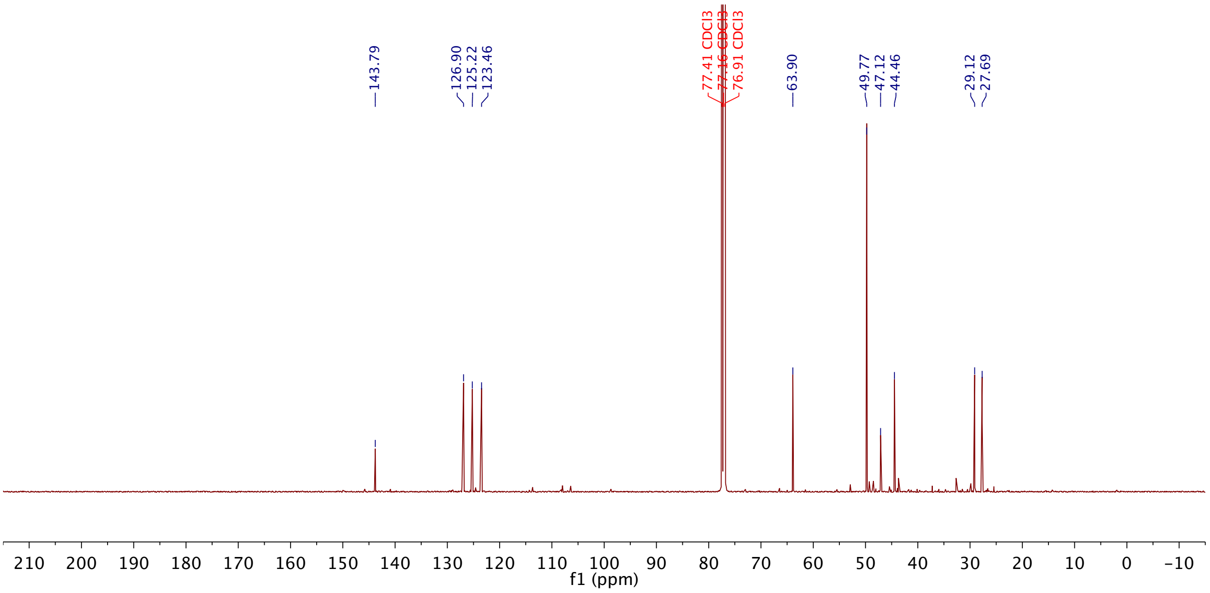

(*R*)-2-(Bicyclo[1.1.1]pentan-1-yl)-3-(pyridin-3-yl)propan-1-ol, 29

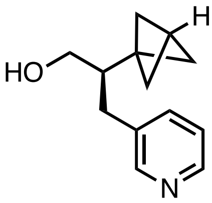

^{1}H NMR (500 MHz, $CDCl_3$)

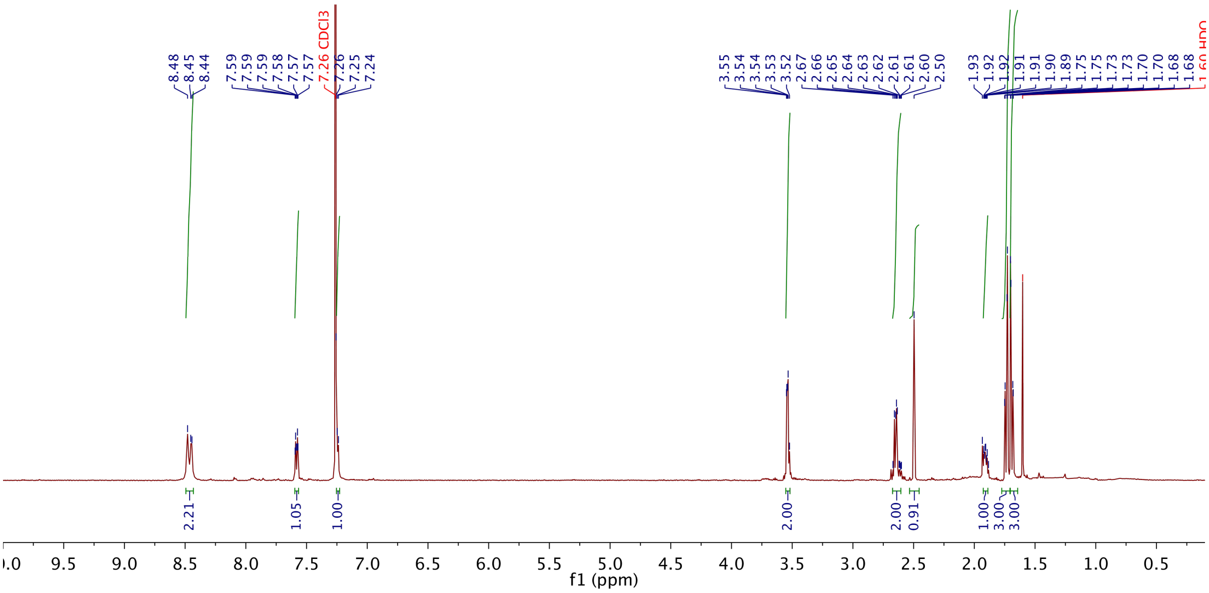

^{13}C NMR (126 MHz, $CDCl_3$)

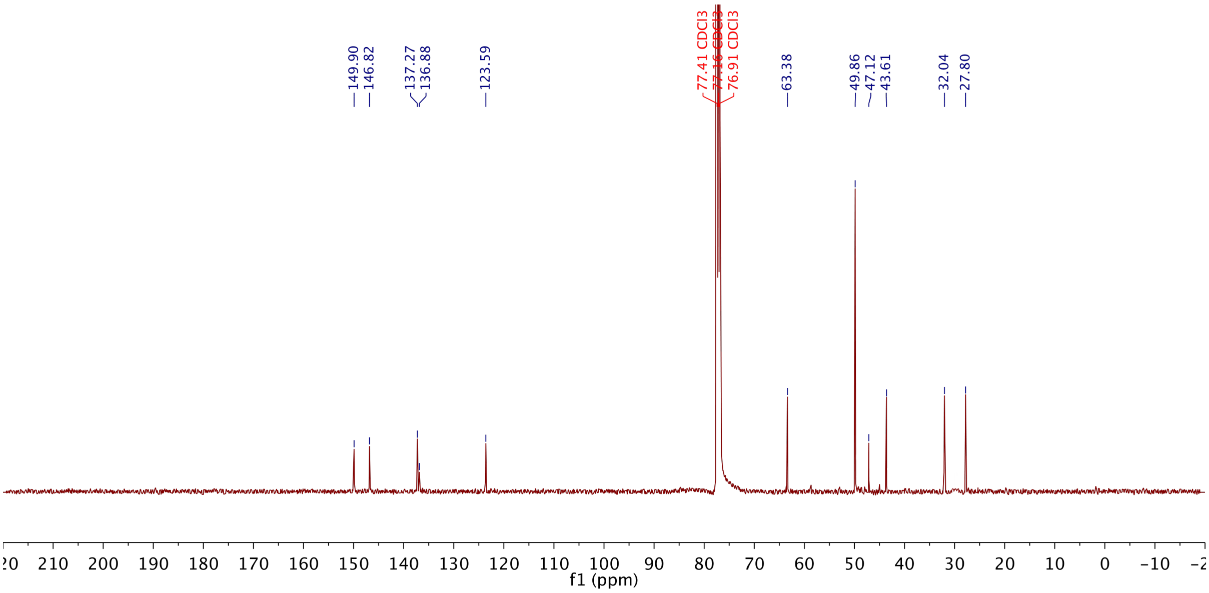

(*R*)-3-Phenyl-2-(3-(phenylthio)bicyclo[1.1.1]pentan-1-yl)propan-1-ol, 30

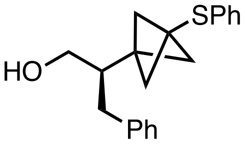

^{1}H NMR (500 MHz, $CDCl_3$)

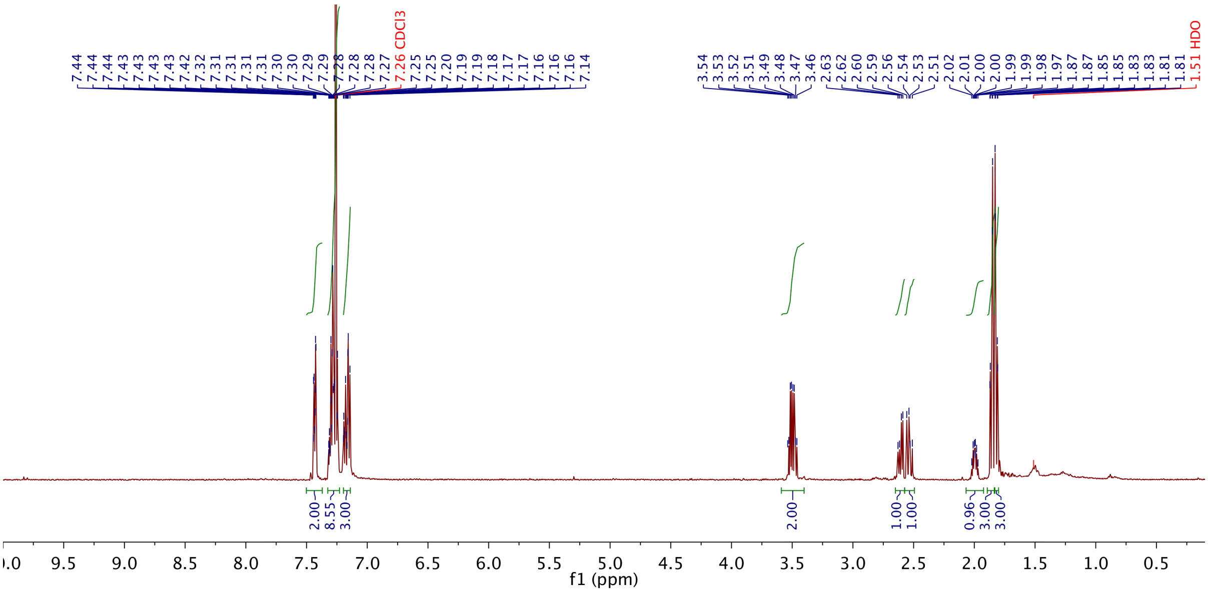

^{13}C NMR (126 MHz, $CDCl_3$)

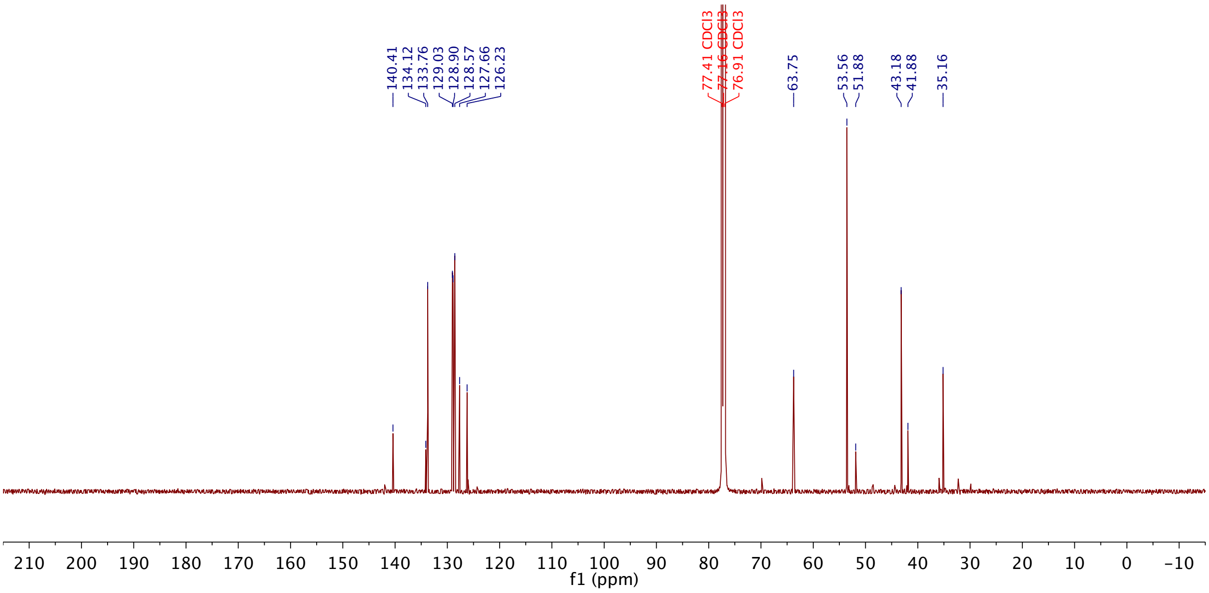

(*R*)-2-(Bicyclo[1.1.1]pentan-1-yl)-3-phenylpropanoic acid, 31

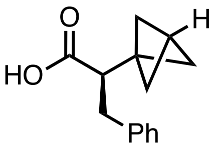

^{1}H NMR (500 MHz, $CDCl_3$)

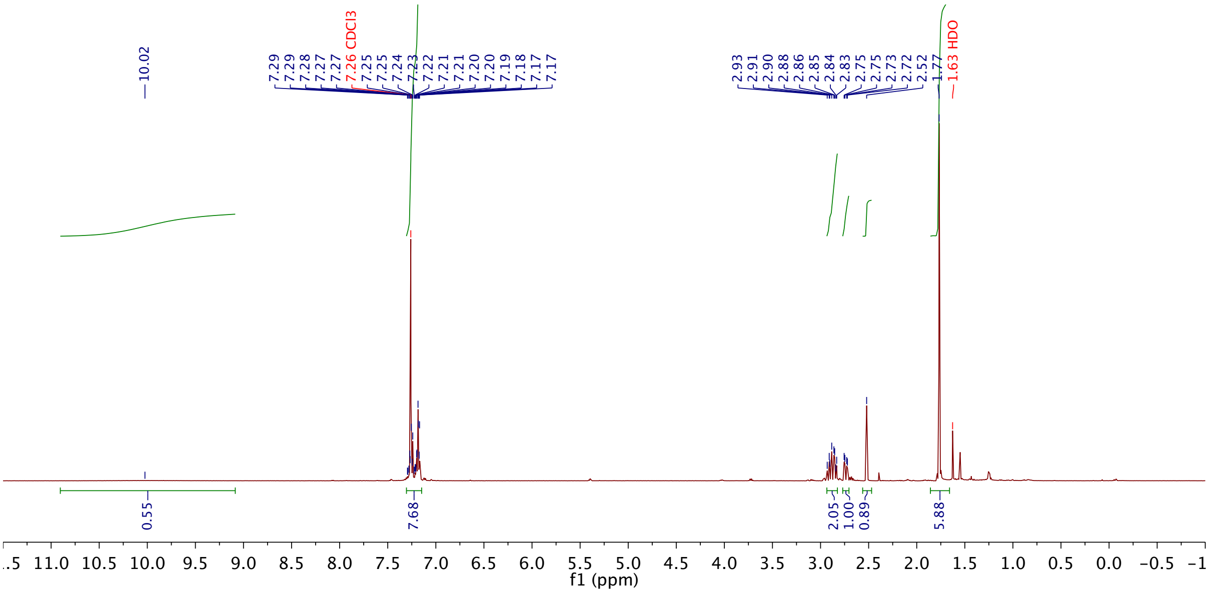

^{13}C NMR (126 MHz, $CDCl_3$)

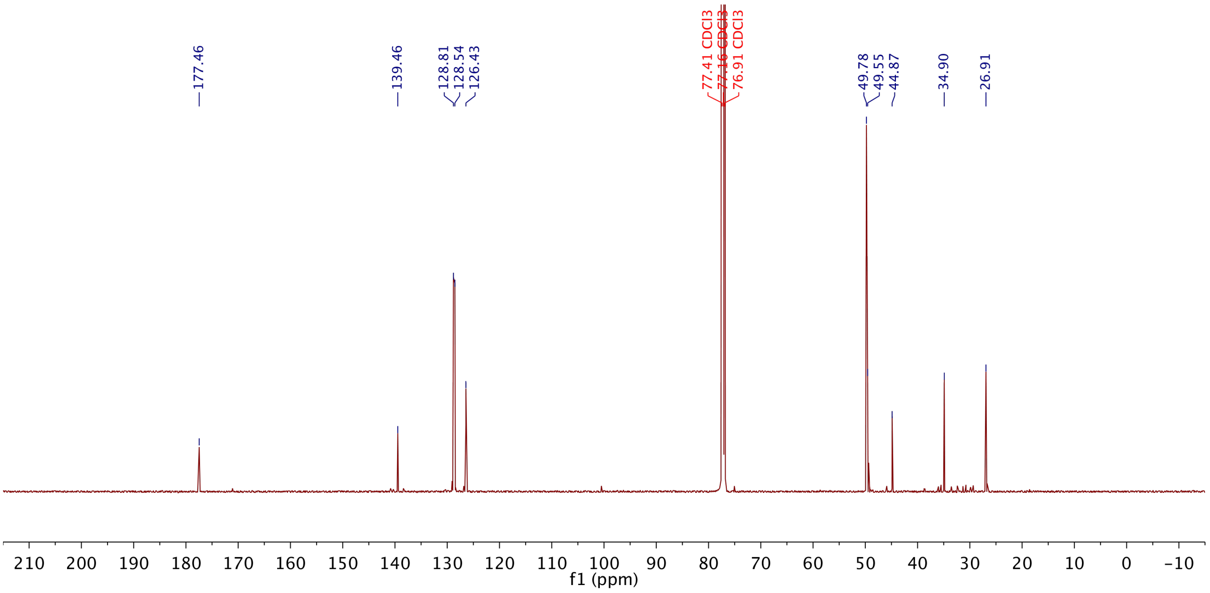

(*R*)-*N*-Benzyl-2-(bicyclo[1.1.1]pentan-1-yl)-3-phenylpropan-1-amine, 32

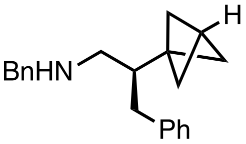

^{1}H NMR (500 MHz, $CDCl_3$)

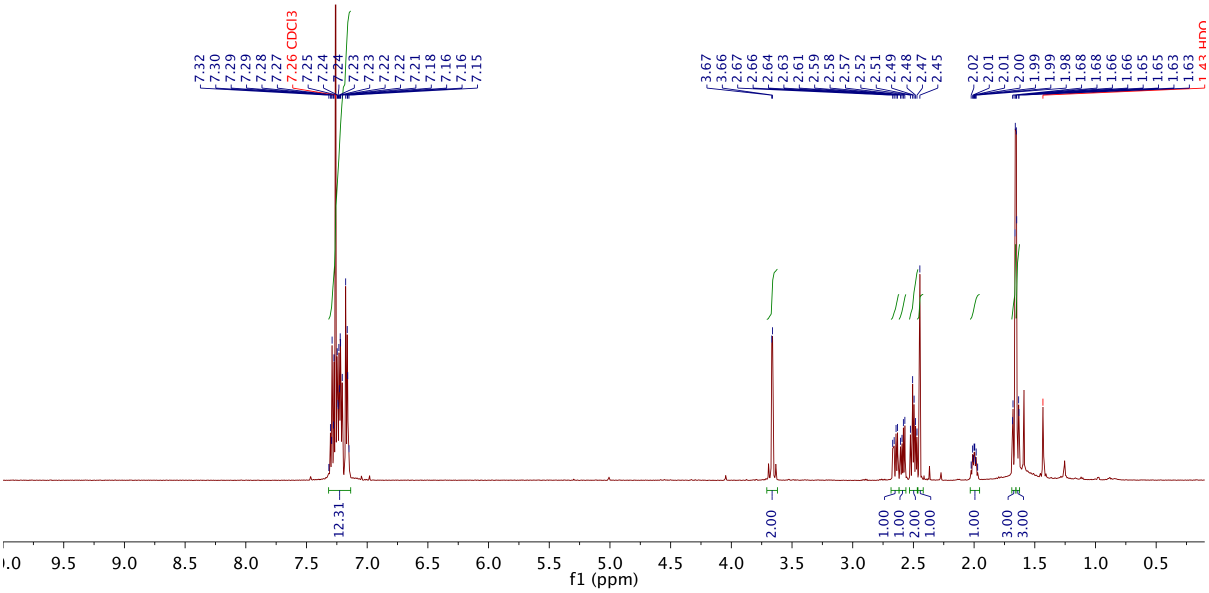

^{13}C NMR (126 MHz, $CDCl_3$)

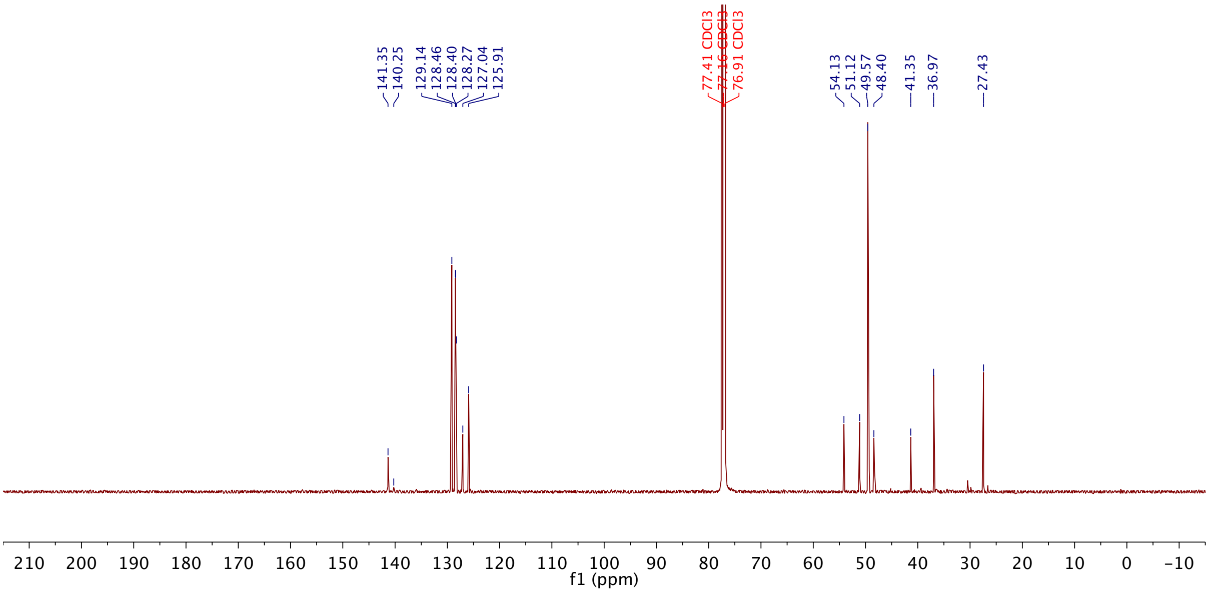

(1*S*,2*R*)-2-(Bicyclo[1.1.1]pentan-1-yl)-1-(4-methoxyphenyl)-3-phenylpropan-1-ol, 33

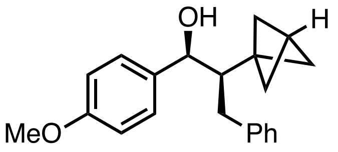

^{1}H NMR (500 MHz, $CDCl_3$)

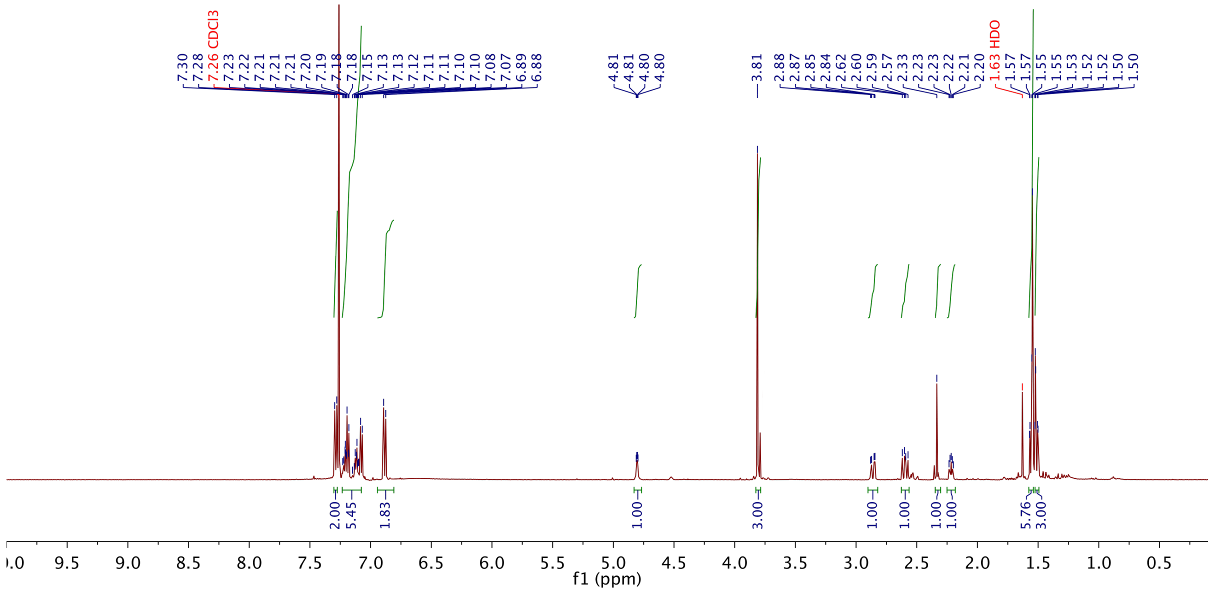

^{13}C NMR (126 MHz, $CDCl_3$)

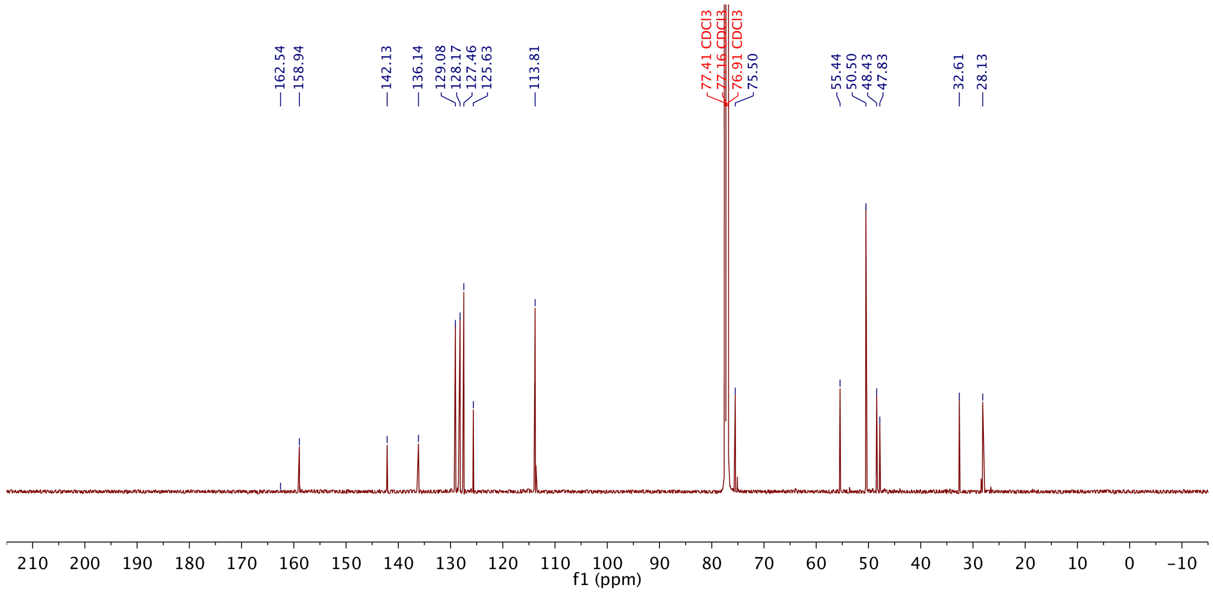

(*R*)-1-(1-Phenylbut-3-yn-2-yl)bicyclo[1.1.1]pentane, 34

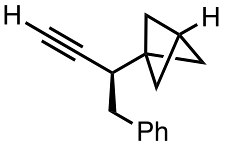

^{1}H NMR (500 MHz, $CDCl_3$)

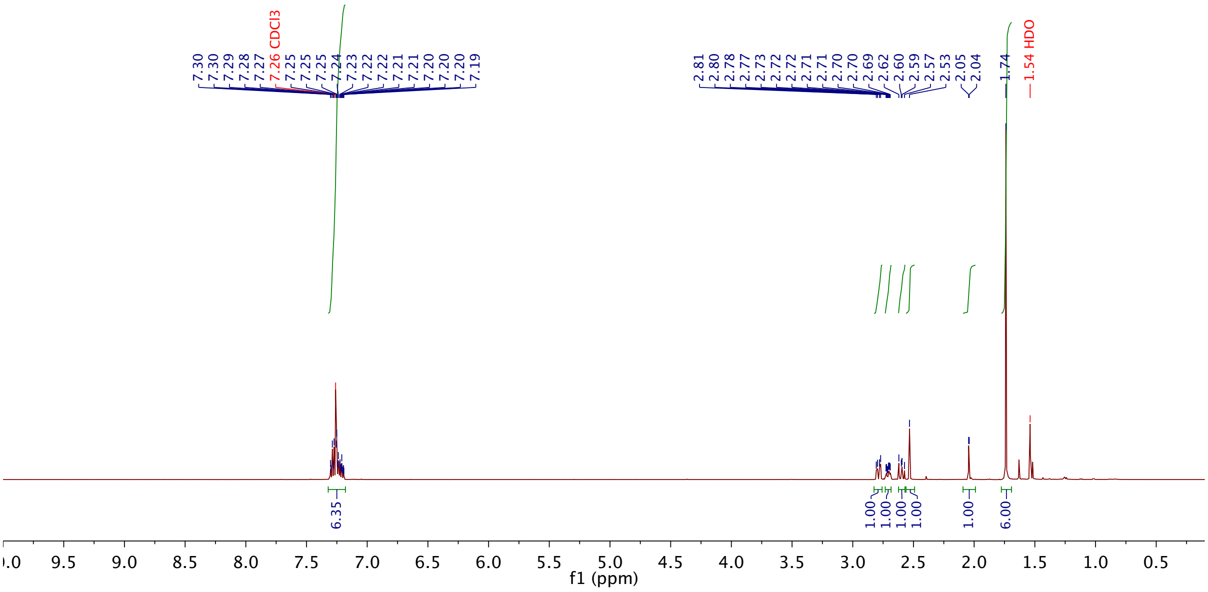

^{13}C NMR (126 MHz, $CDCl_3$)

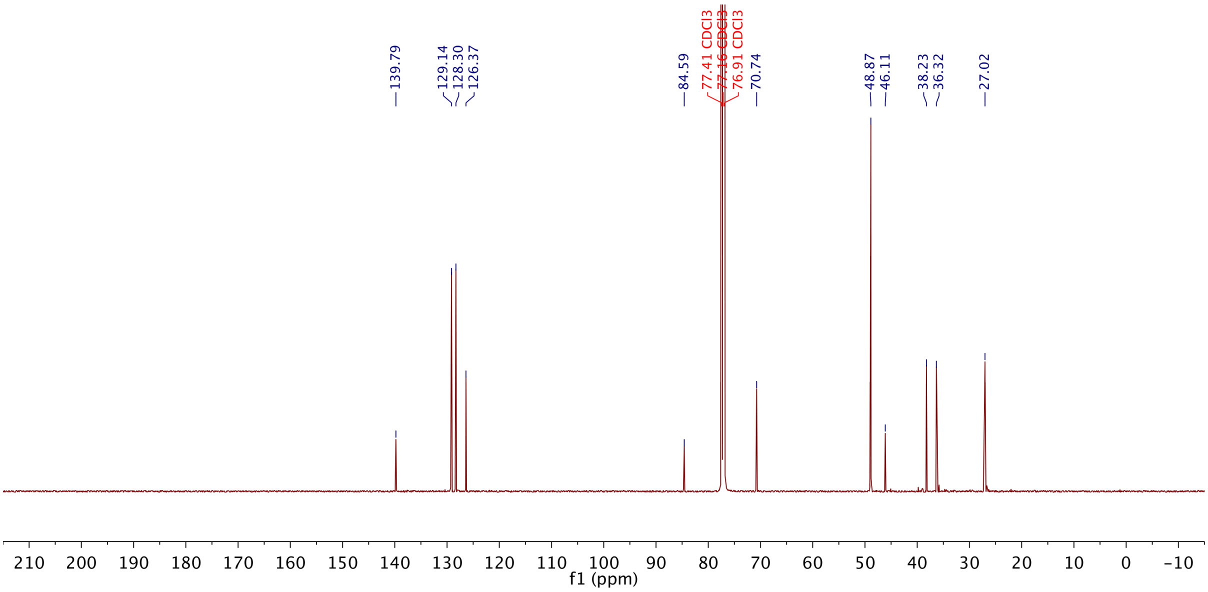

2,4,6-Tri-*tert*-butylbenzeneselenol, S1

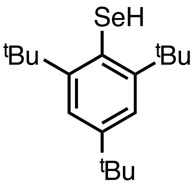

^{1}H NMR (500 MHz, $CDCl_3$)

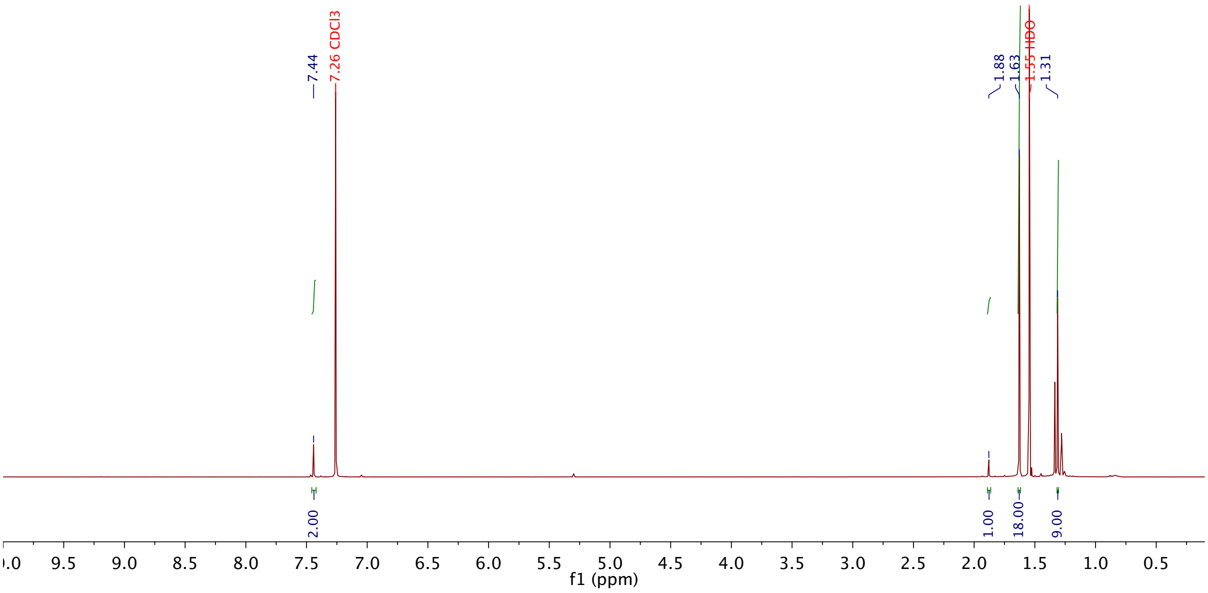

^{13}C NMR (126 MHz, $CDCl_3$)

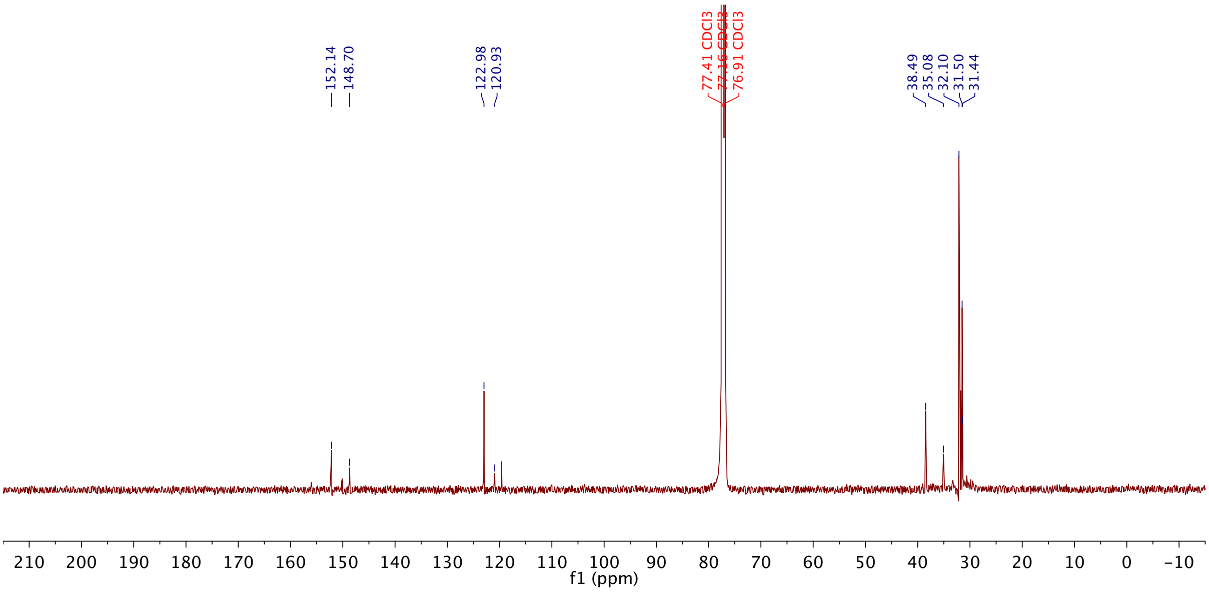

2,4,6-Tricyclohexylbenzenethiol, S3

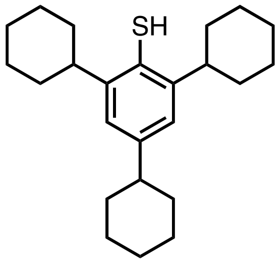

^{1}H NMR (500 MHz, $CDCl_3$)

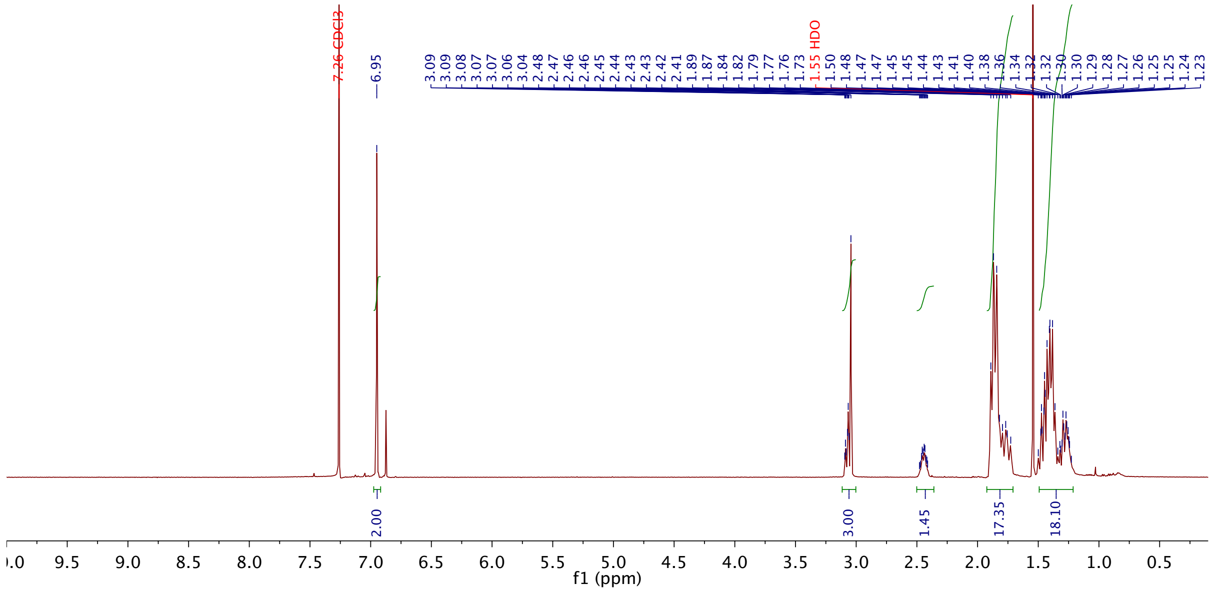

^{13}C NMR (126 MHz, $CDCl_3$)

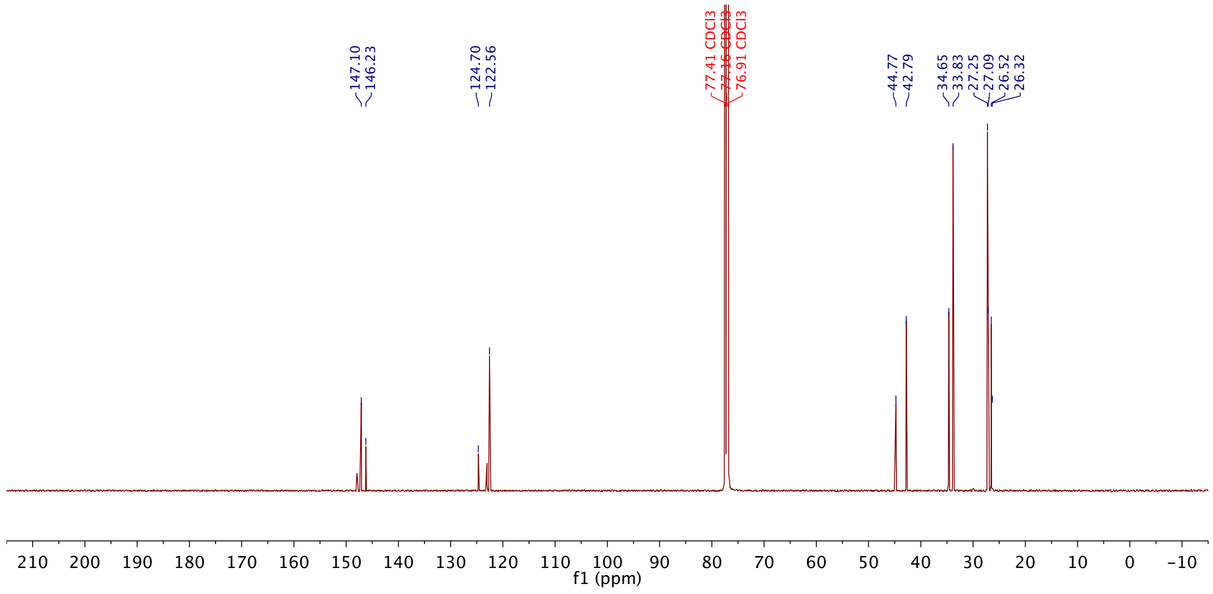

2,4,6-Tricyclopentylbenzenethiol, S4

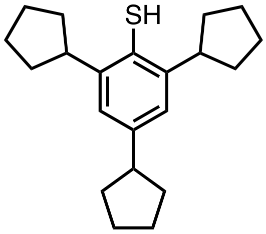

^{1}H NMR (400 MHz, $CDCl_3$)

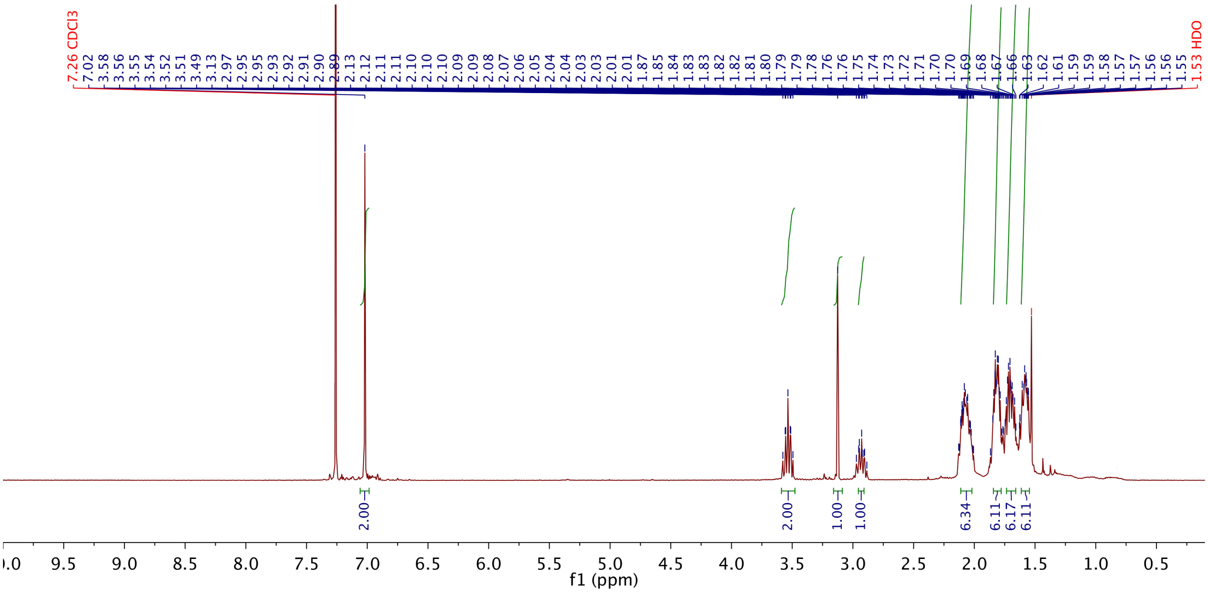

^{13}C NMR (126 MHz, $CDCl_3$)

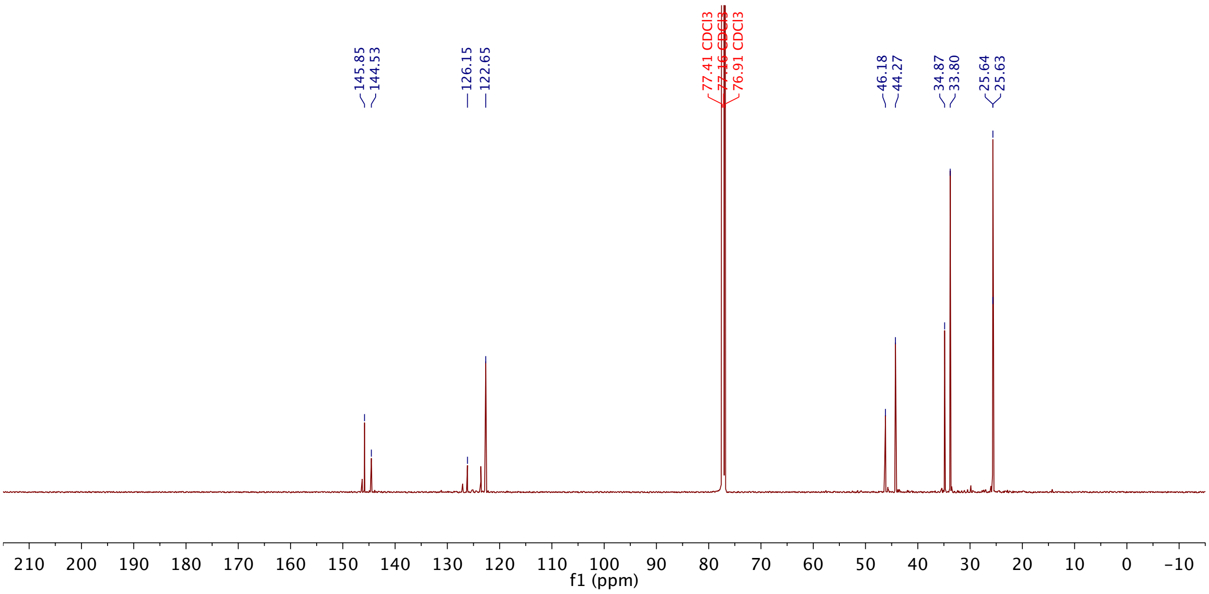

2,6-Diisopropyl-4-methoxybenzenethiol, S6

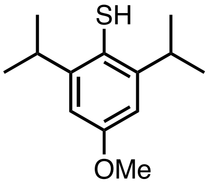

¹H NMR (400 MHz, $CDCl_3$)

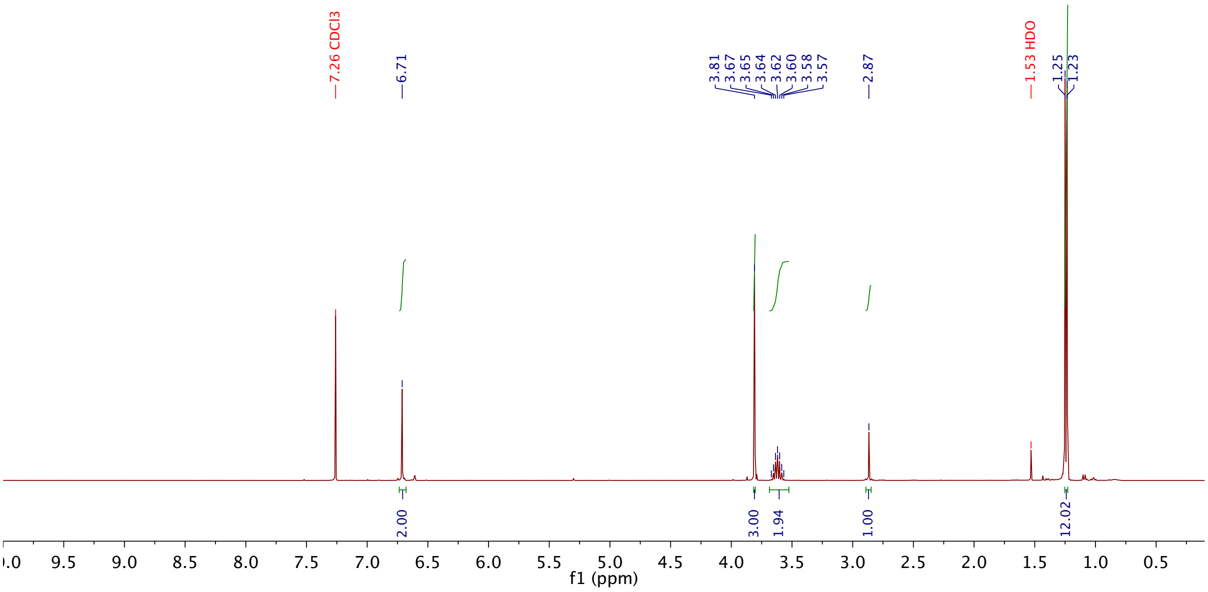

¹³C NMR (126 MHz, $CDCl_3$)

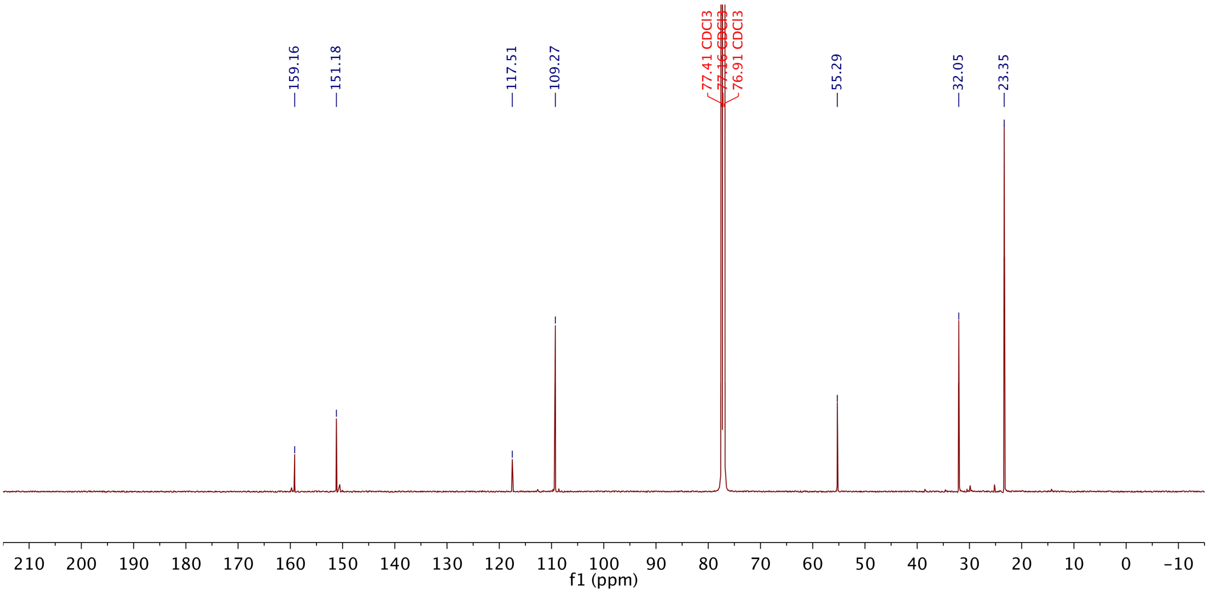

3,5-Di-*tert*-butylbenzenethiol, S7

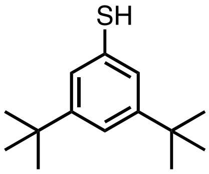

^{1}H NMR (500 MHz, $CDCl_3$)

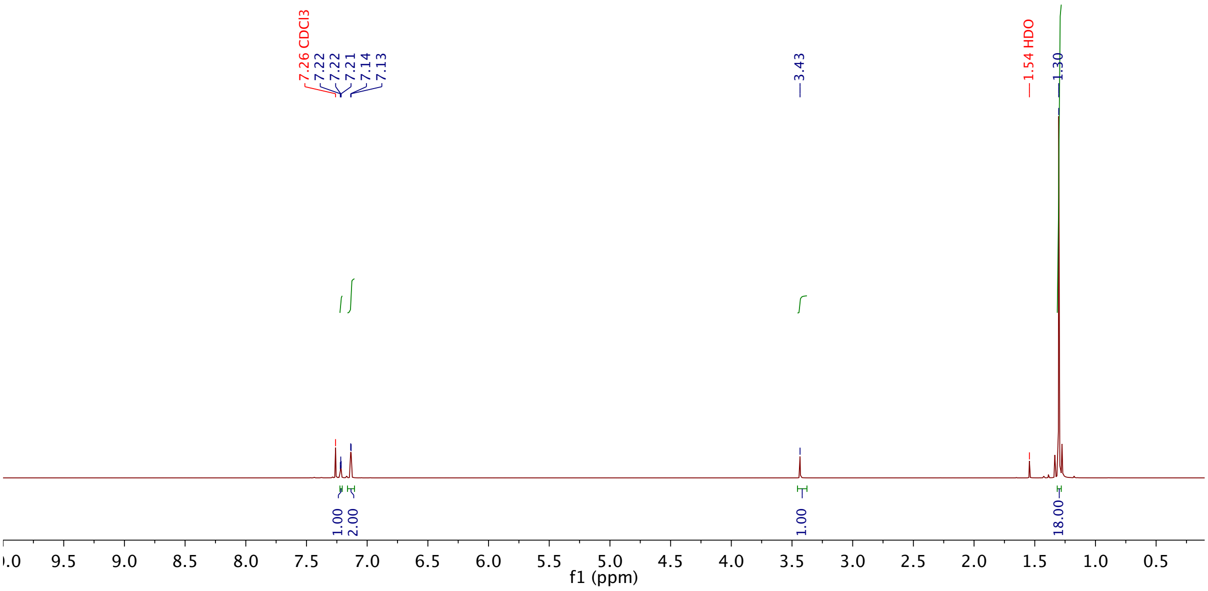

^{13}C NMR (126 MHz, $CDCl_3$)

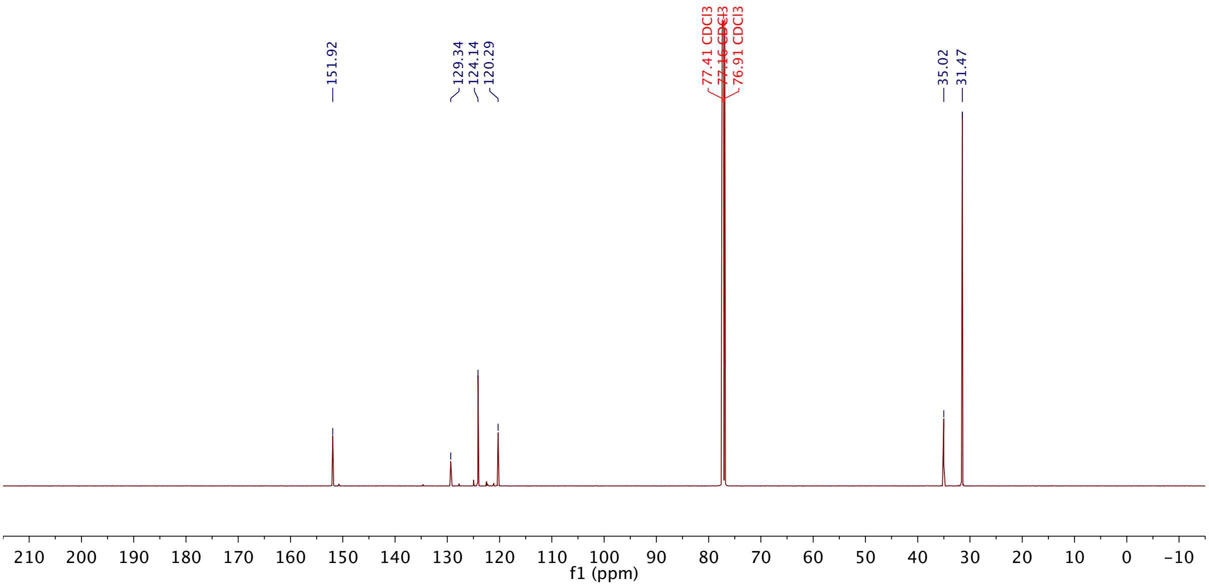

2,4,6-Triisopropyl-*N*-(pyridin-3-yl)benzenesulfonamide, S10

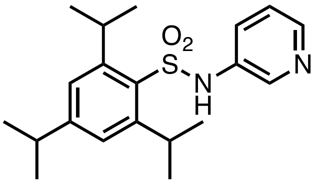

^{1}H NMR (500 MHz, $CDCl_3$)

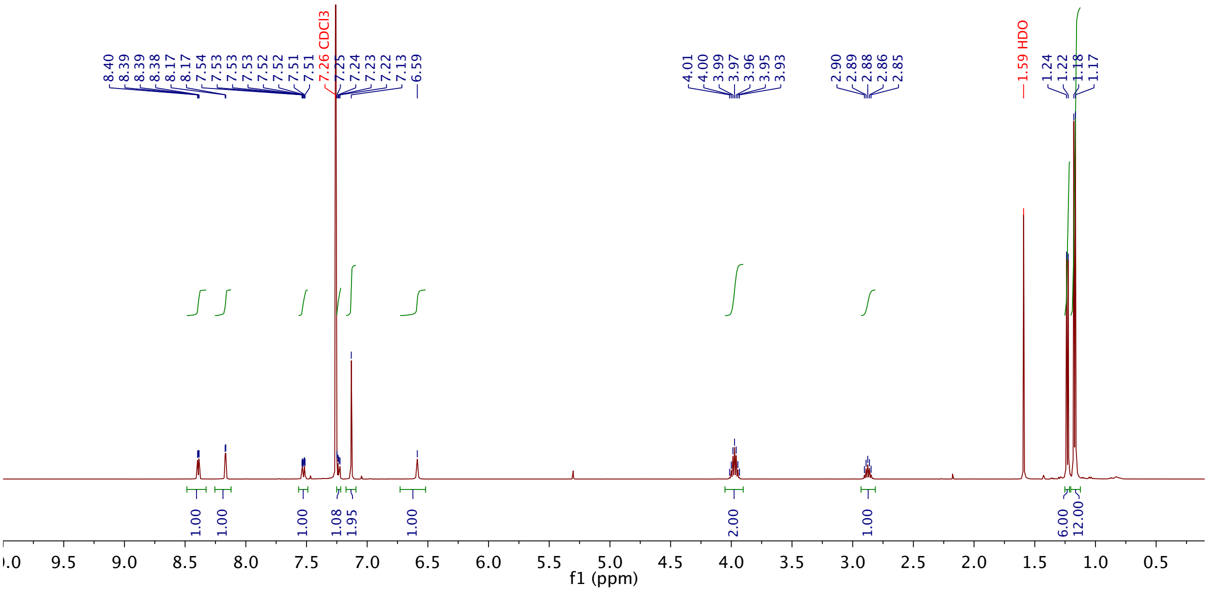

^{13}C NMR (126 MHz, $CDCl_3$)

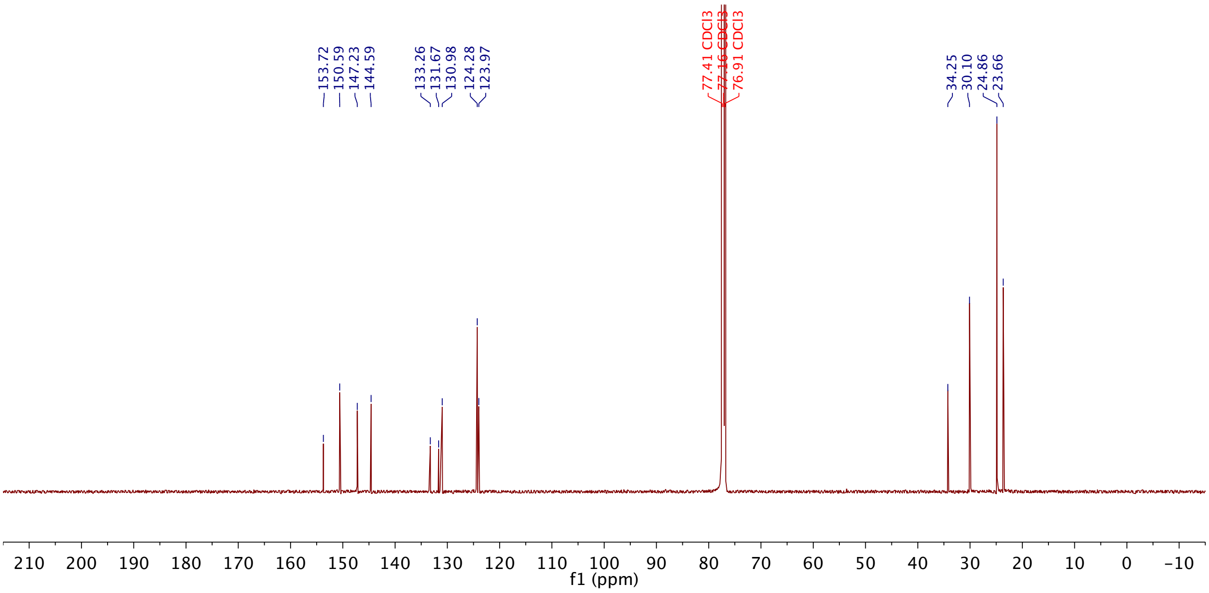

Bicyclo[1.1.1]pentan-1-yl(2,4,6-tri-*tert*-butylphenyl)sulfane, S28

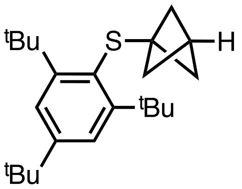

^{1}H NMR (500 MHz, $CDCl_3$)

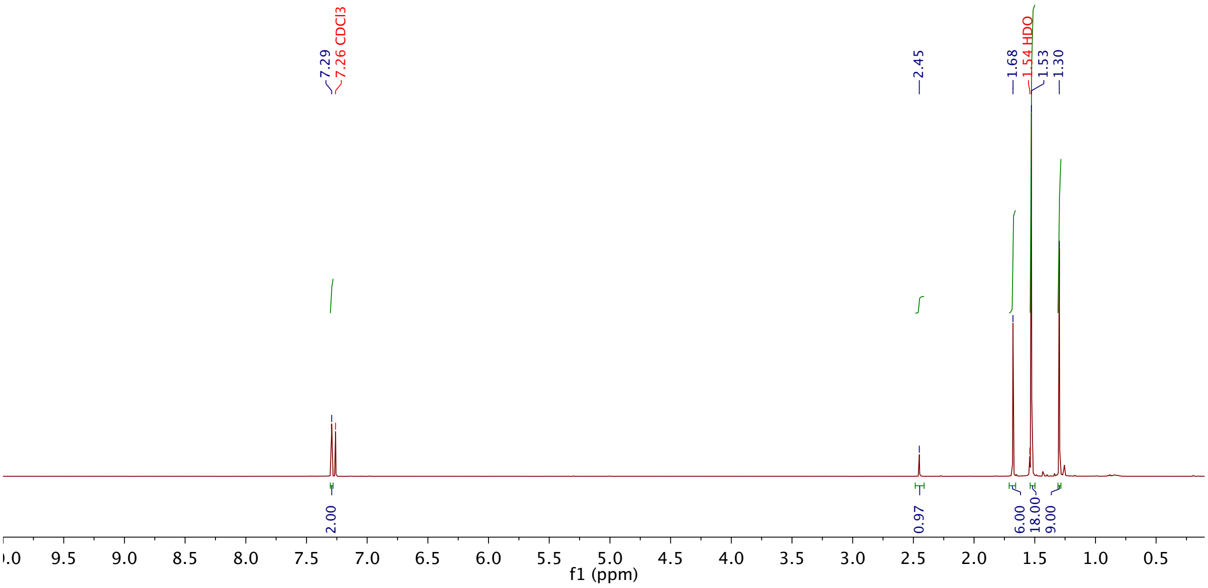

^{13}C NMR (126 MHz, $CDCl_3$)

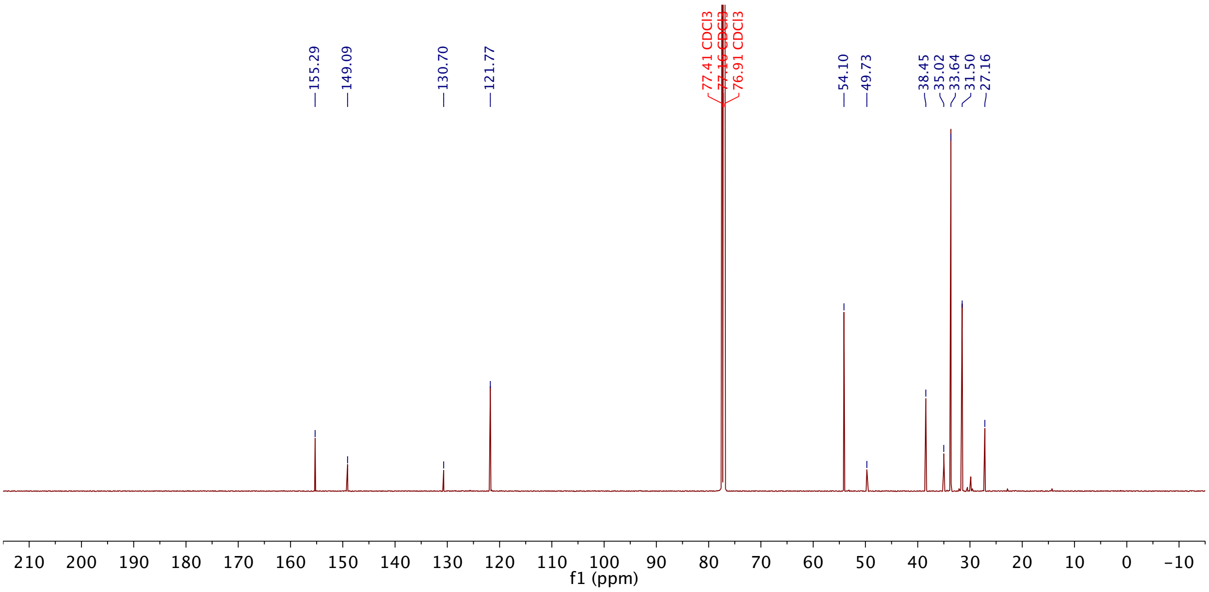

(*S*)-2-(Bis(3,5-bis(trifluoromethyl)phenyl)((trimethylsilyl)oxy)methyl)-4,4-difluoropyrrolidine, S39

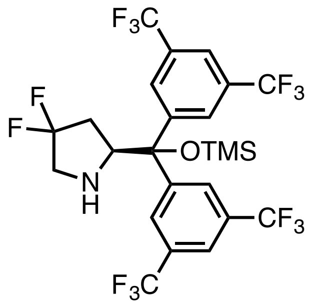

^{1}H NMR (400 MHz, $CDCl_3$)

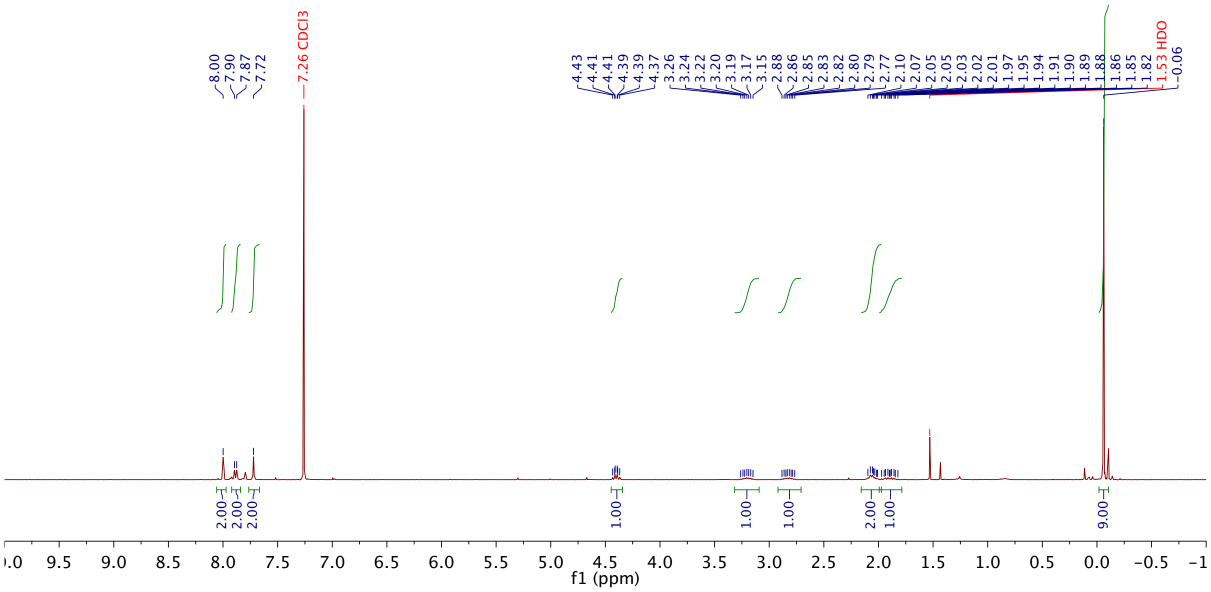

^{13}C NMR (126 MHz, $CDCl_3$)

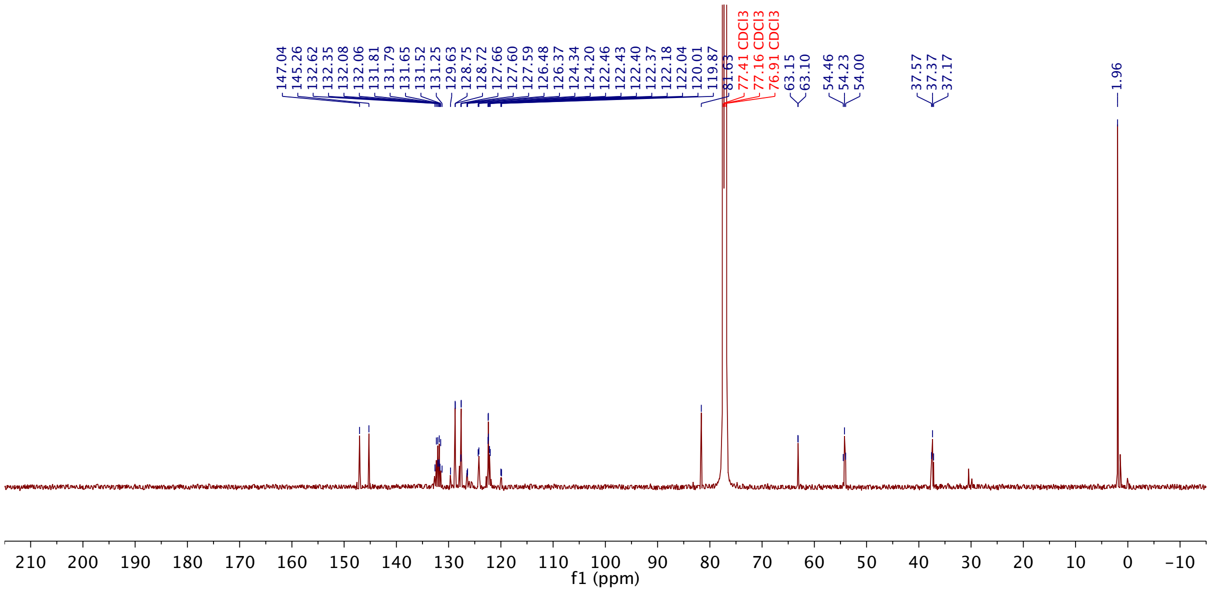

^{19}**F NMR** (471 MHz, $CDCl_3$)

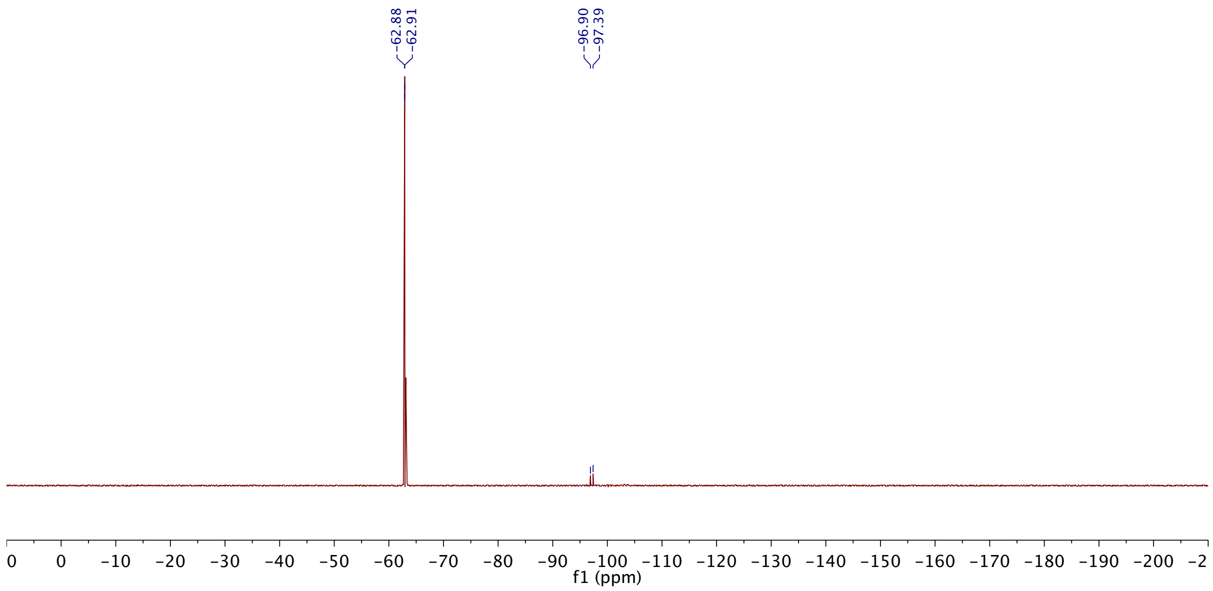

(2*S*,4*R*)-2-(Bis(3,5-bis(trifluoromethyl)phenyl)((trimethylsilyl)oxy)methyl)-4-((trimethylsilyl)oxy)pyrrolidine, S40

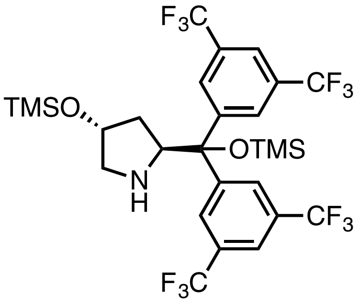

^{1}H NMR (400 MHz, $CDCl_3$)

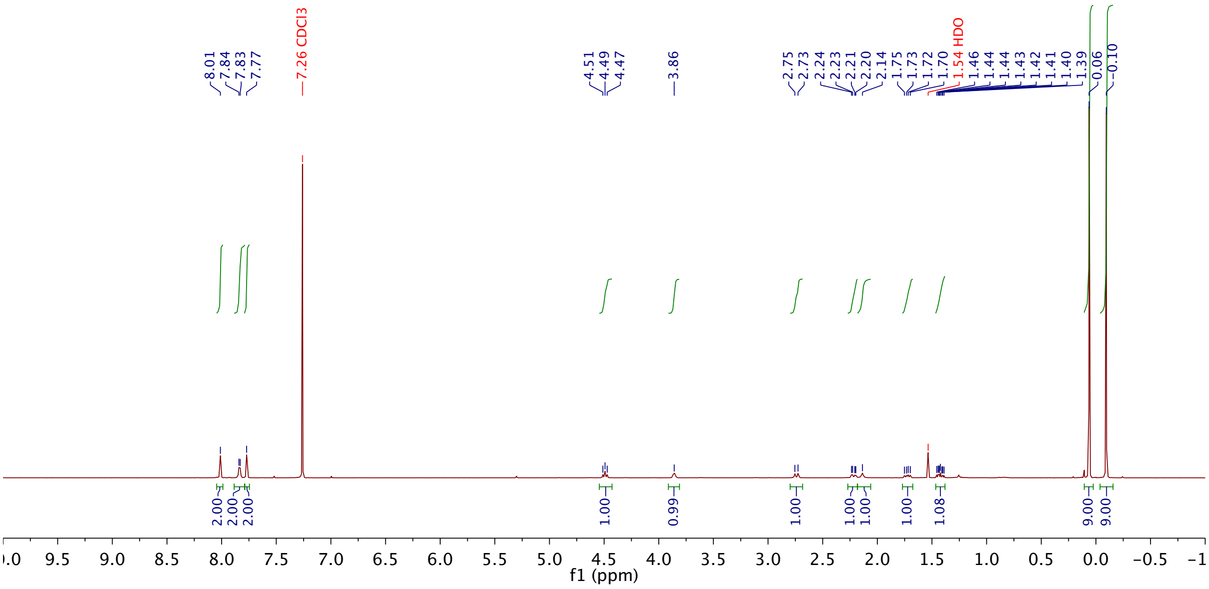

^{13}C NMR (126 MHz, $CDCl_3$)

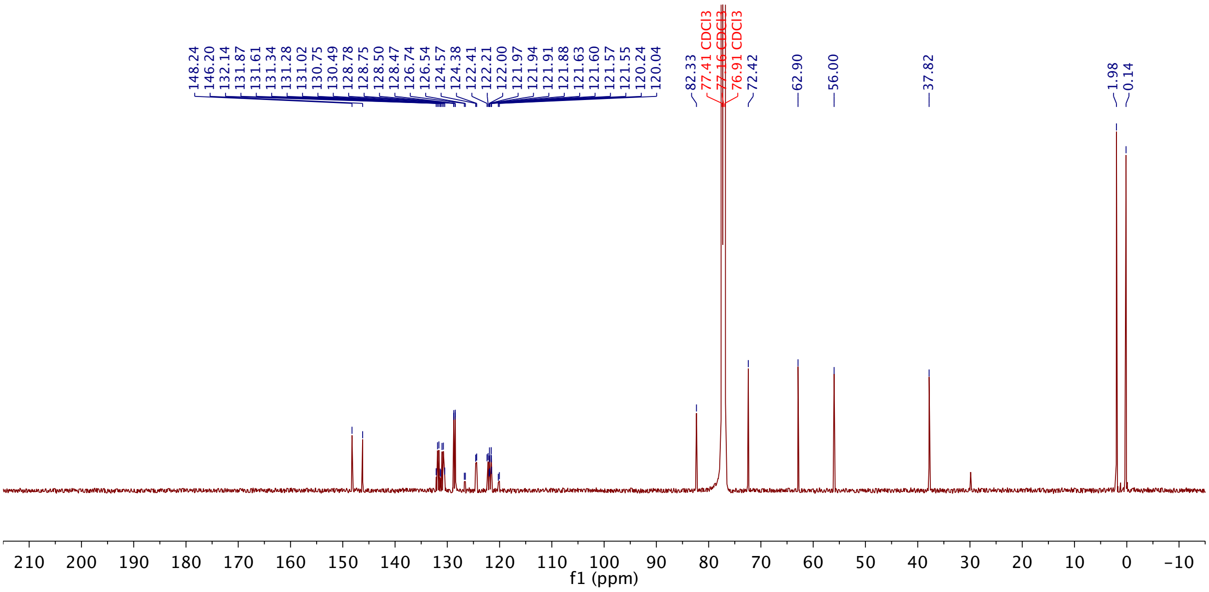

^{19}F NMR (471 MHz, $CDCl_3$)

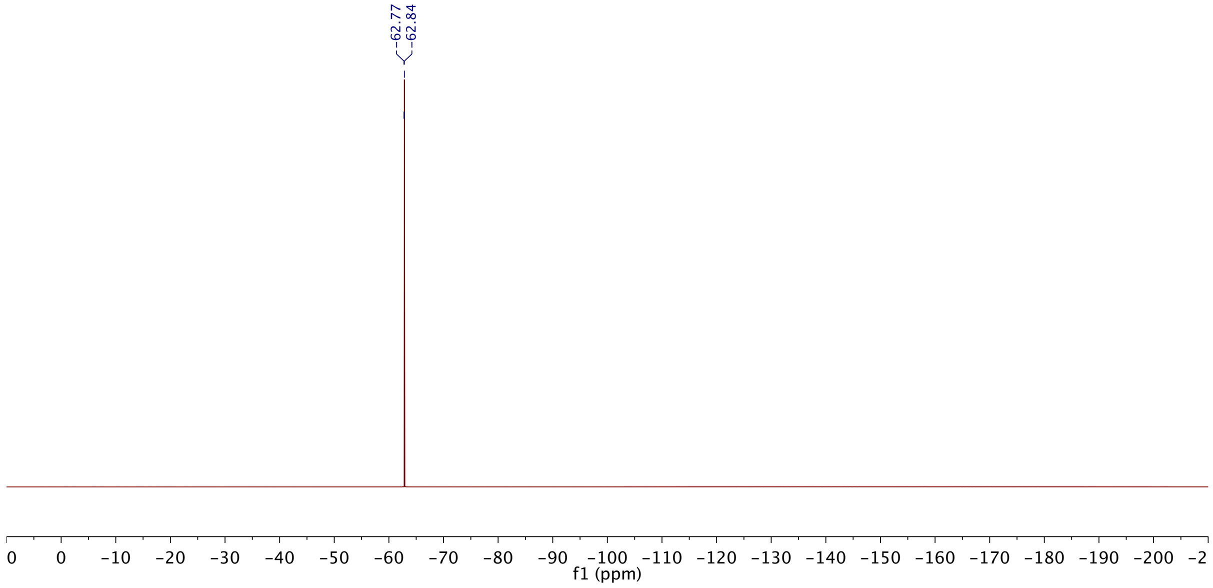

2-(Bicyclo[1.1.1]pentan-1-yl)propyl 4-(dimethylamino)benzoate, S41

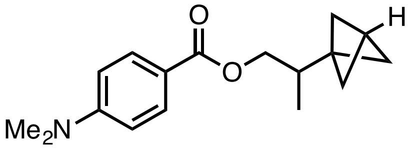

^{1}H NMR (400 MHz, $CDCl_3$)

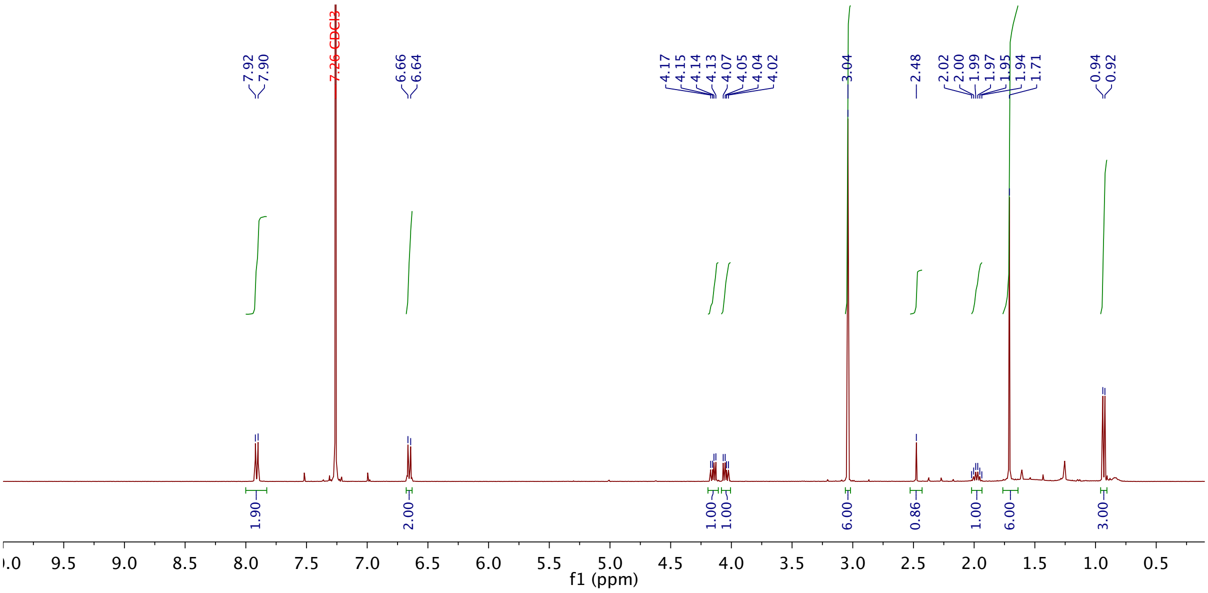

^{13}C NMR (151 MHz, $CDCl_3$)

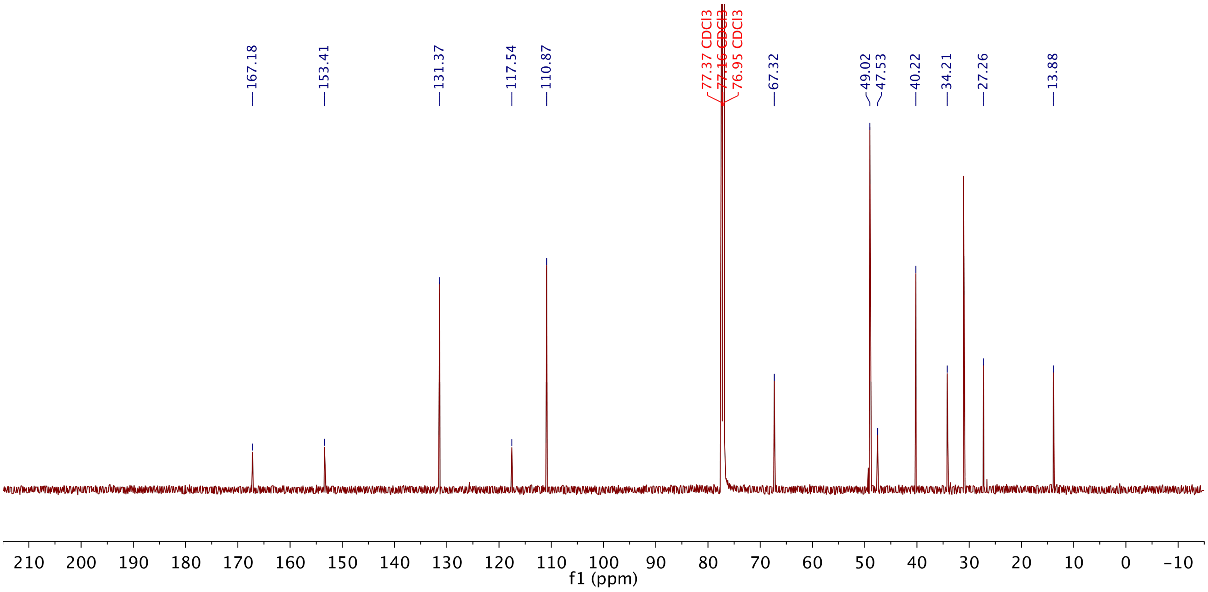

2-(Bicyclo[1.1.1]pentan-1-yl)-3-methylbutyl 4-(dimethylamino)benzoate, S42

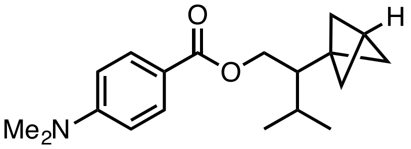

^{1}H NMR (500 MHz, $CDCl_3$)

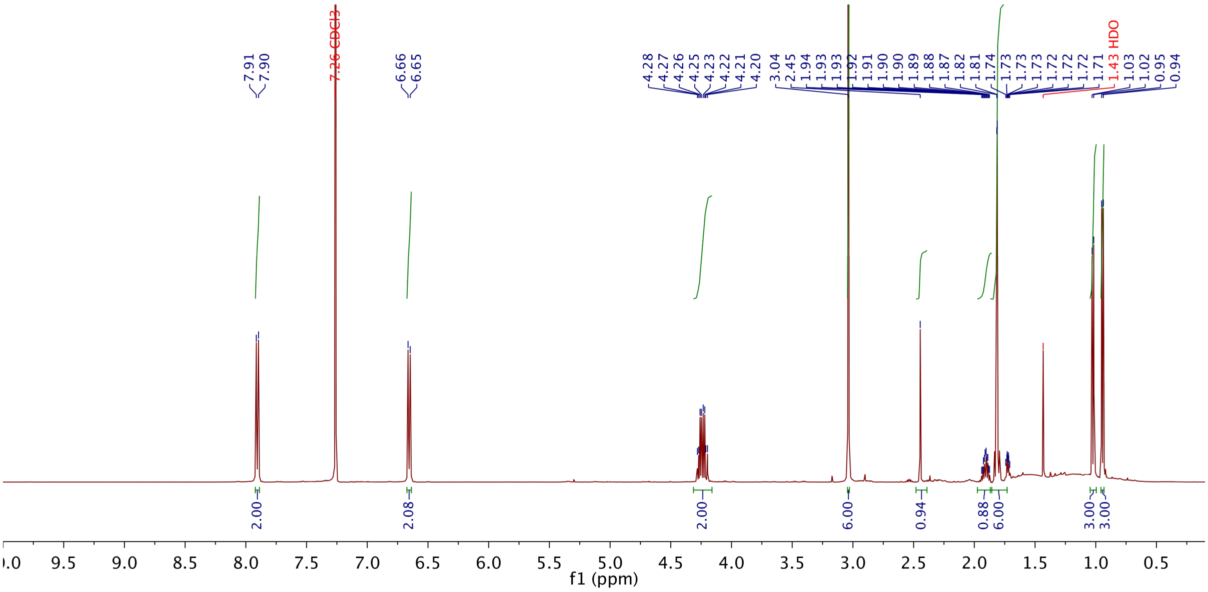

^{13}C NMR (126 MHz, $CDCl_3$)

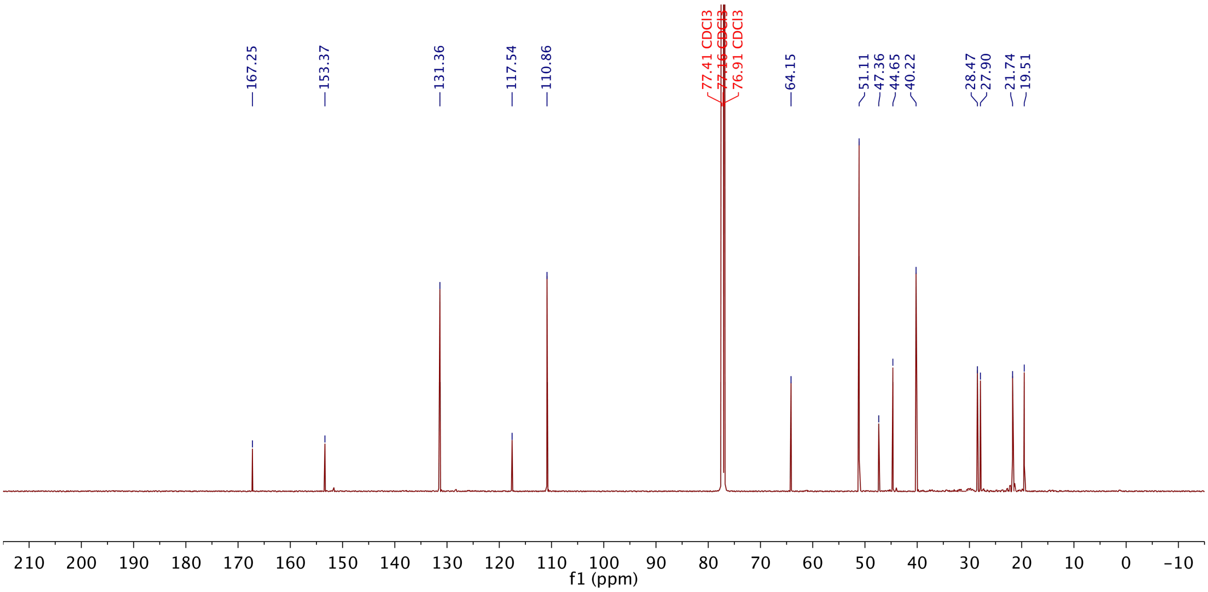

2-(Bicyclo[1.1.1]pentan-1-yl)-3,3-dimethylbutyl 4-(dimethylamino)benzoate, S43

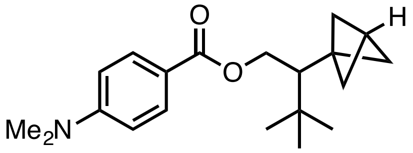

[1]**H NMR** (500 MHz, $CDCl_3$)

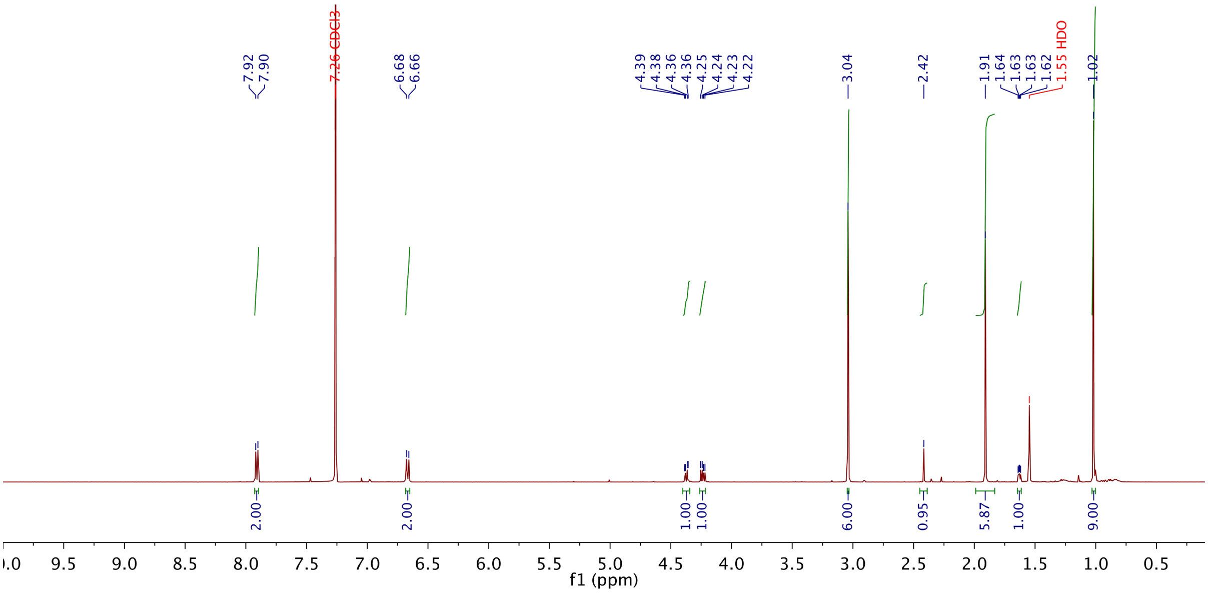

[13]**C NMR** (126 MHz, $CDCl_3$)

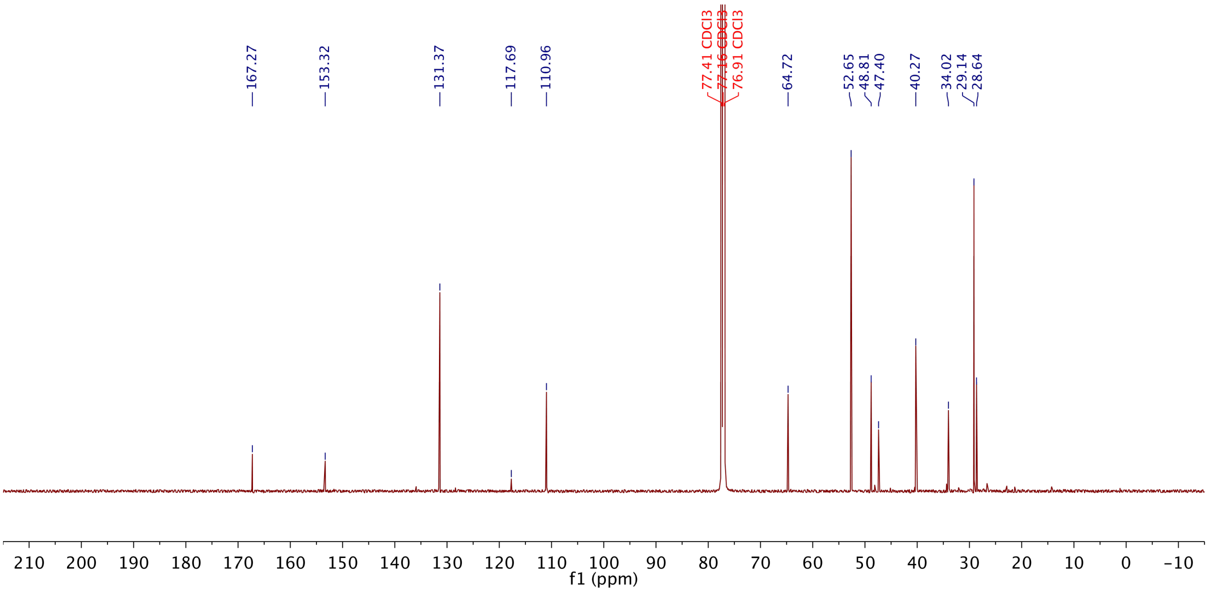

2-(Bicyclo[1.1.1]pentan-1-yl)octyl 4-(dimethylamino)benzoate, S44

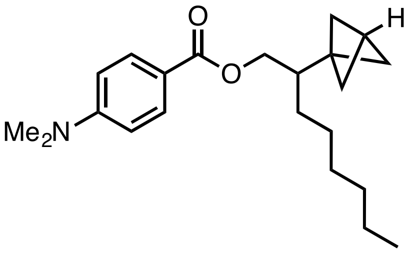

^{1}H NMR (500 MHz, $CDCl_3$)

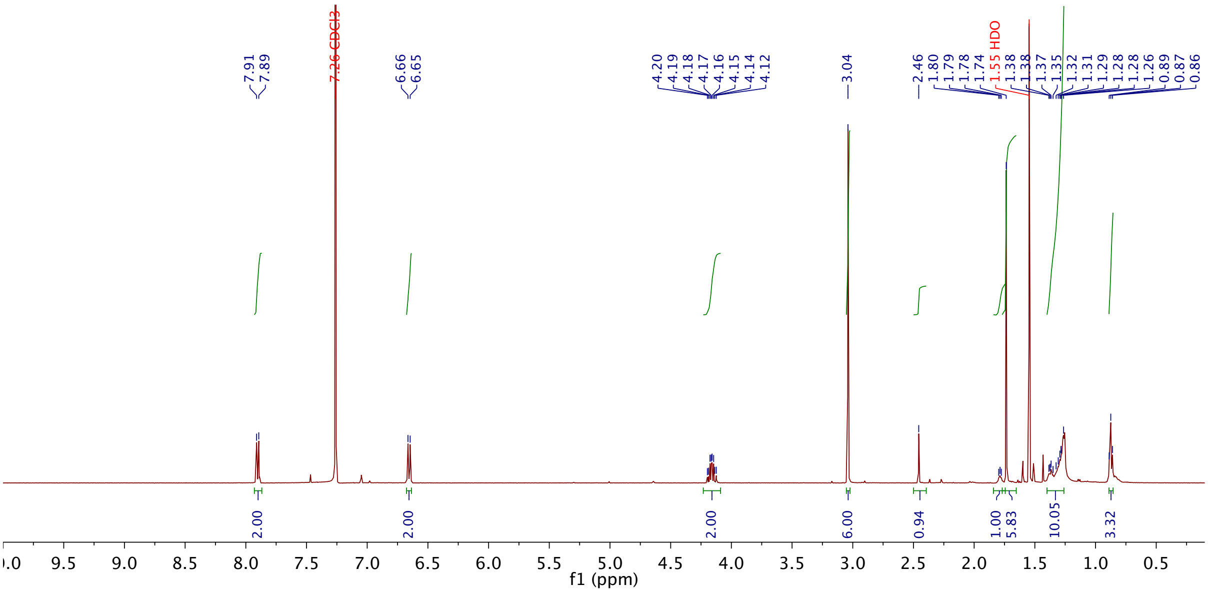

^{13}C NMR (126 MHz, $CDCl_3$)

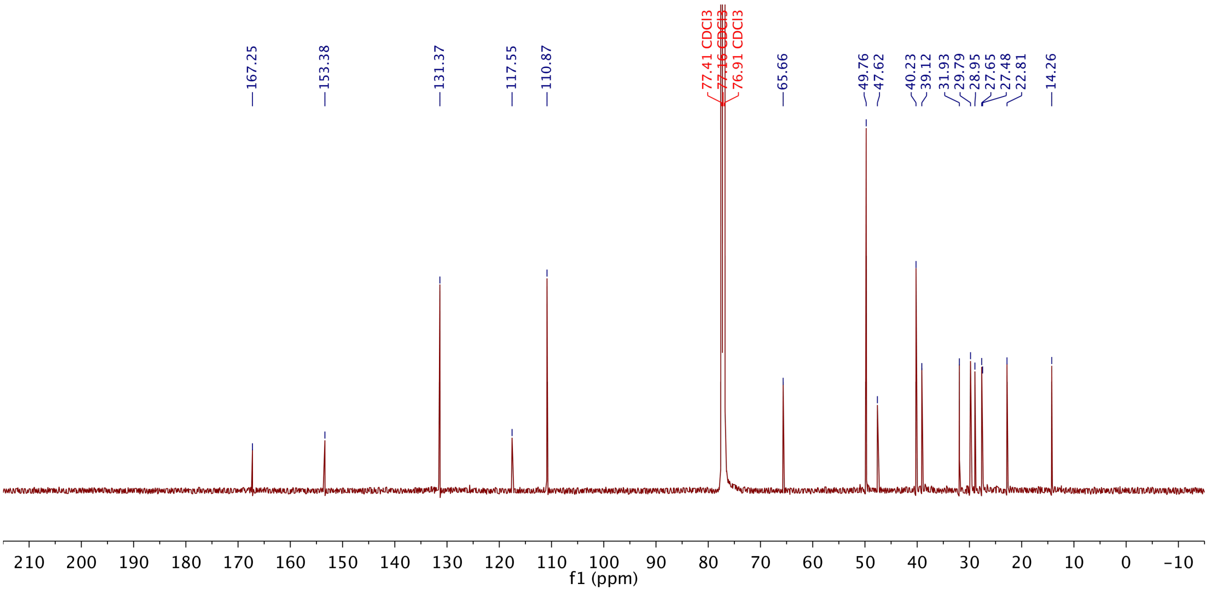

(*Z*)-2-(Bicyclo[1.1.1]pentan-1-yl)hept-4-en-1-yl 4-(dimethylamino)benzoate, S45

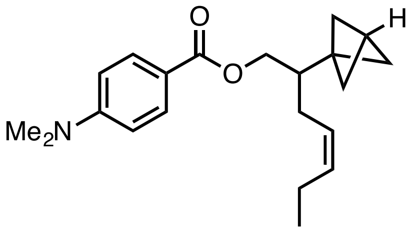

^{1}H NMR (500 MHz, $CDCl_3$)

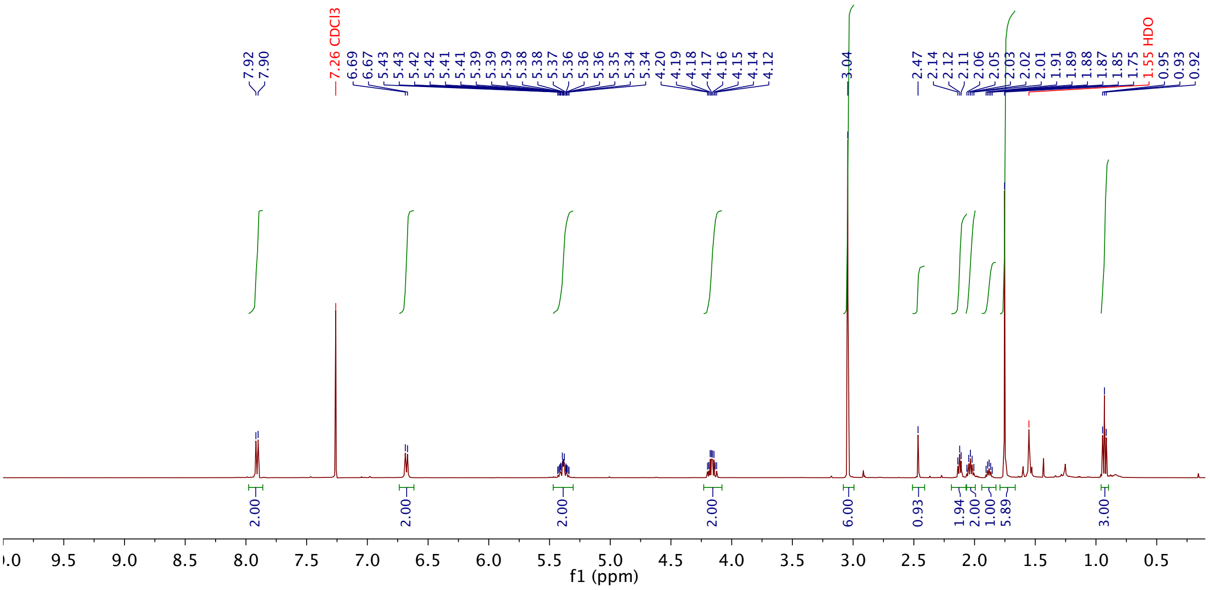

^{13}C NMR (126 MHz, $CDCl_3$)

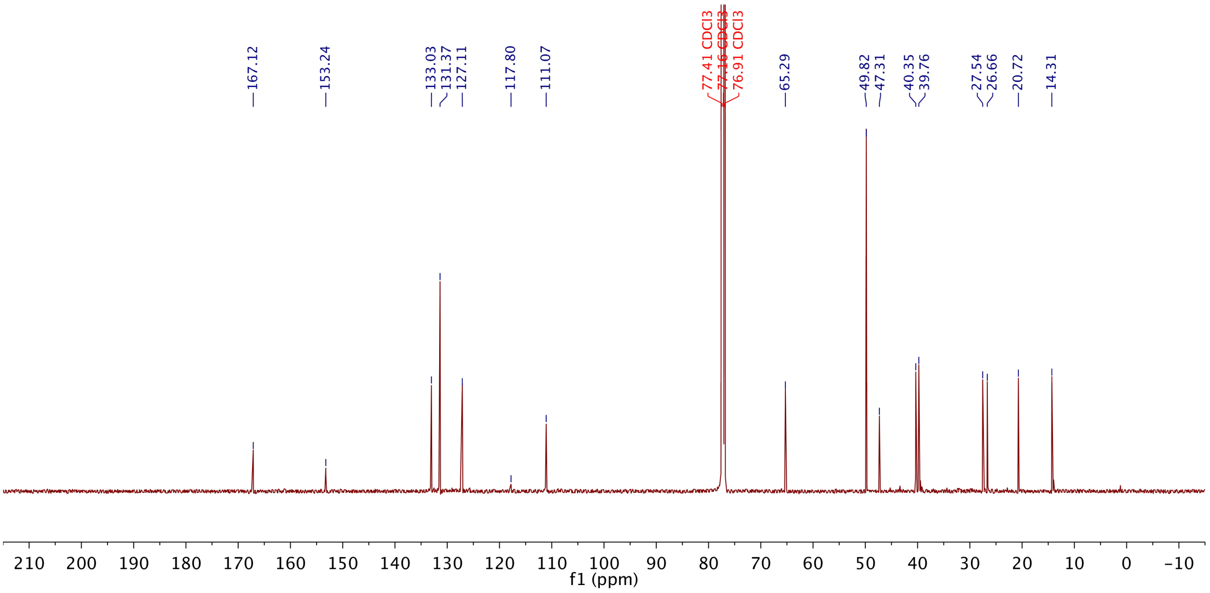

2-(Bicyclo[1.1.1]pentan-1-yl)-3-(methylthio)propyl 4-(dimethylamino)benzoate, S46

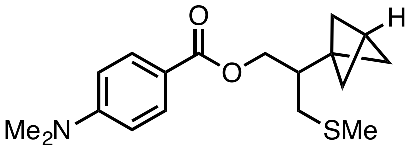

^{1}H NMR (500 MHz, $CDCl_3$)

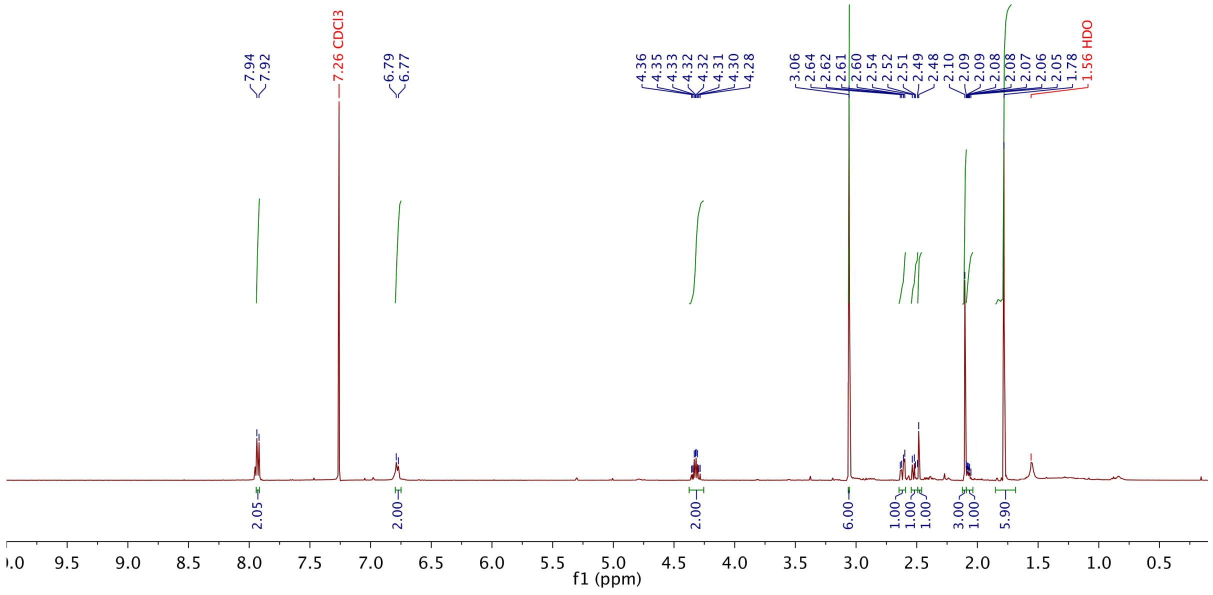

^{13}C NMR (126 MHz, $CDCl_3$)

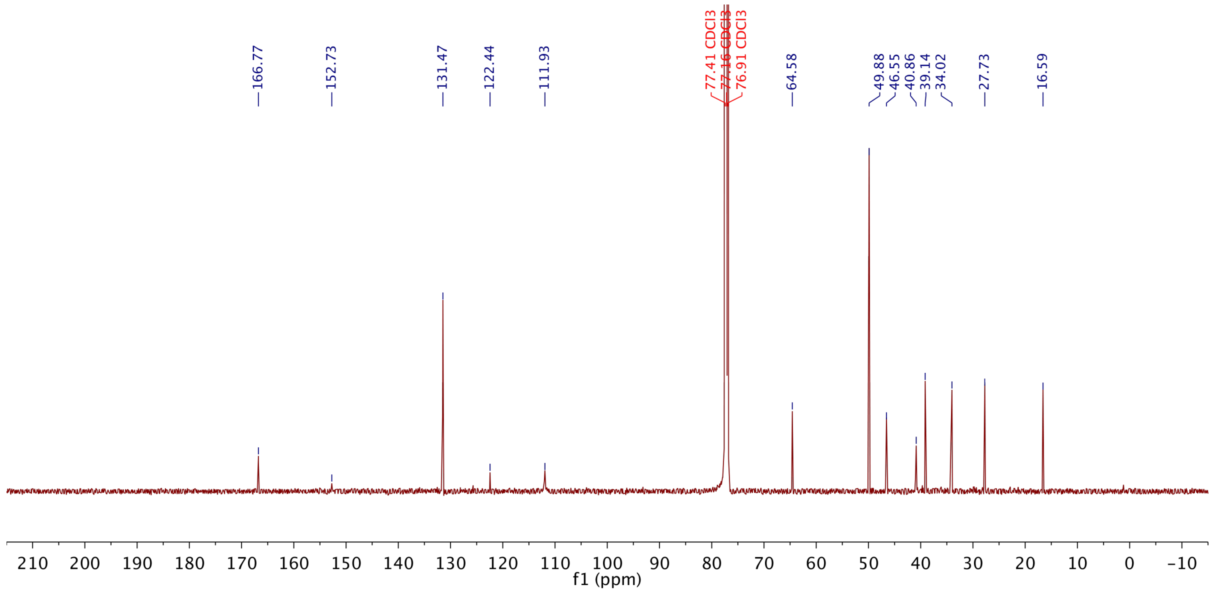

2-(Bicyclo[1.1.1]pentan-1-yl)-6-chlorohexyl 4-(dimethylamino)benzoate, S47

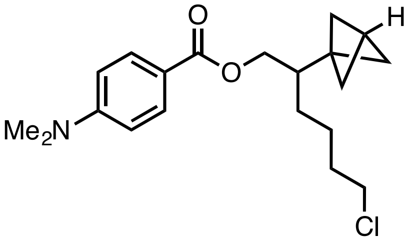

^{1}H NMR (500 MHz, $CDCl_3$)

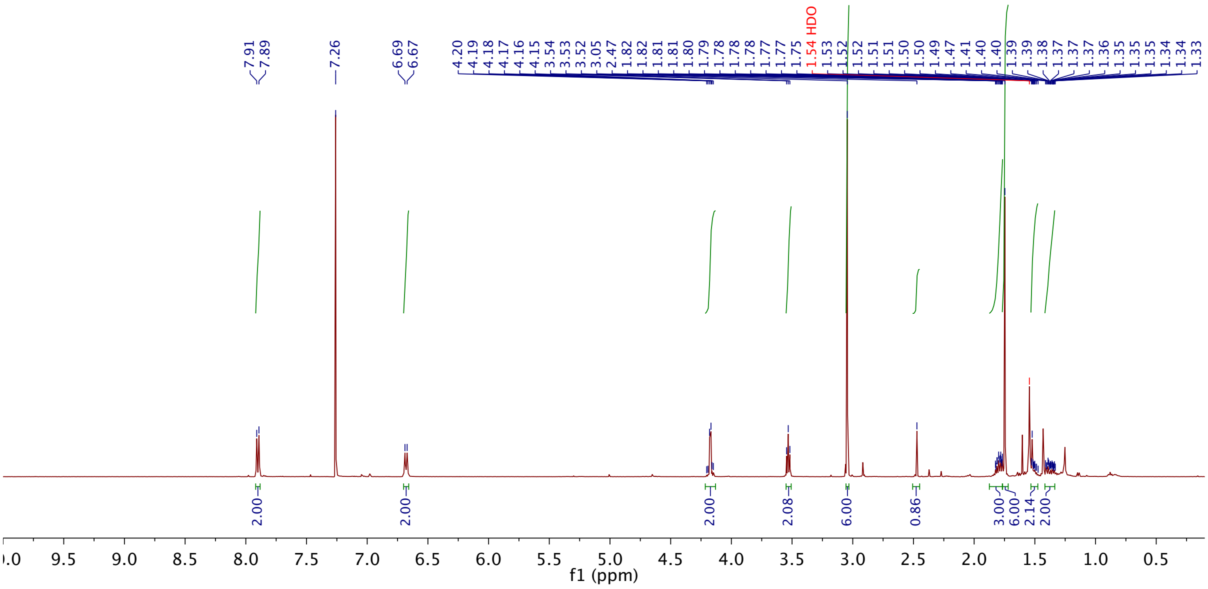

^{13}C NMR (126 MHz, $CDCl_3$)

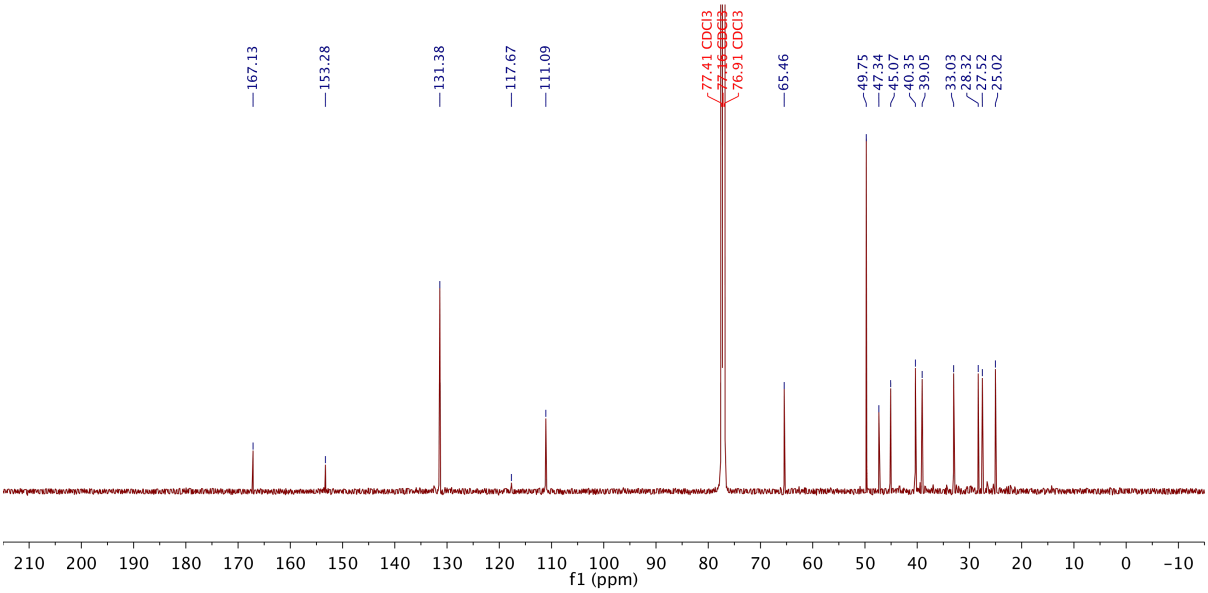

2-(Bicyclo[1.1.1]pentan-1-yl)-6-ethoxy-6-oxohexyl 4-(dimethylamino)benzoate, S48

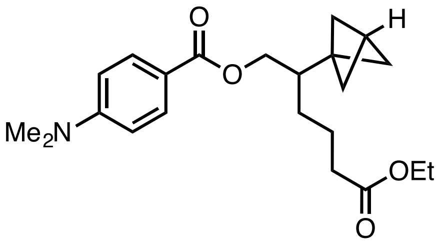

^{1}H NMR (500 MHz, $CDCl_3$)

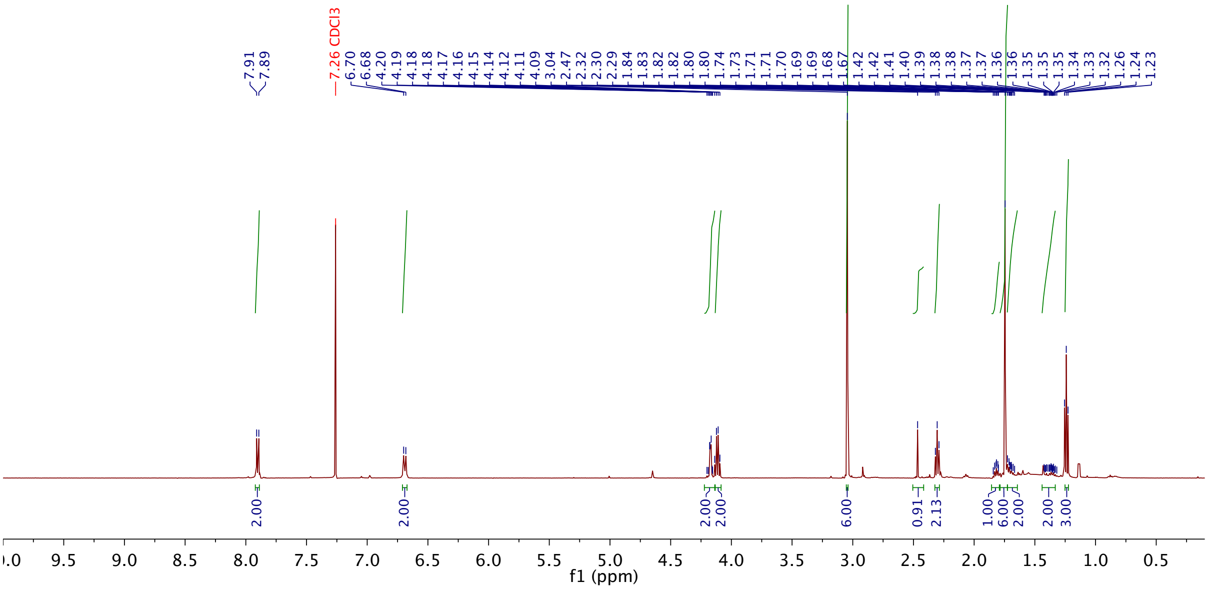

^{13}C NMR (126 MHz, $CDCl_3$)

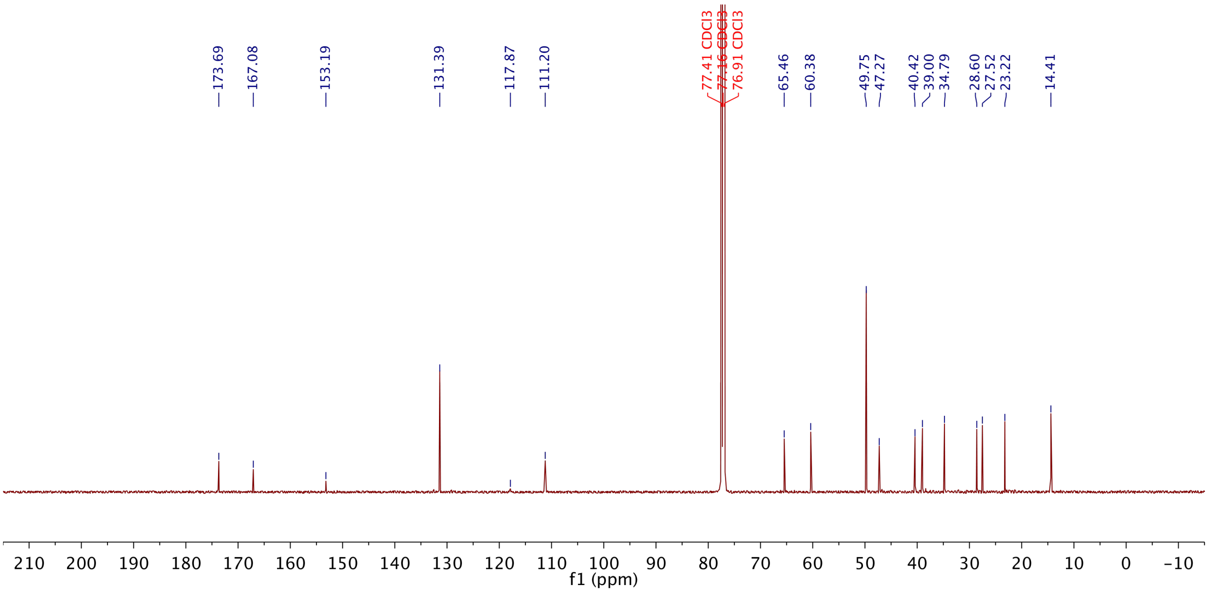

***tert*-Butyl 4-(1-(bicyclo[1.1.1]pentan-1-yl)-2-((4-(dimethylamino)benzoyl)oxy)ethyl)-piperidine-1-carboxylate, S49**

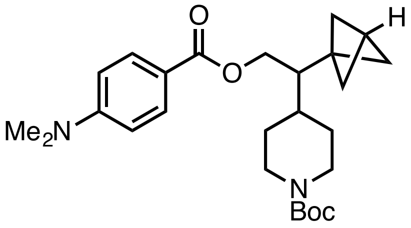

^{1}H NMR (500 MHz, $CDCl_3$)

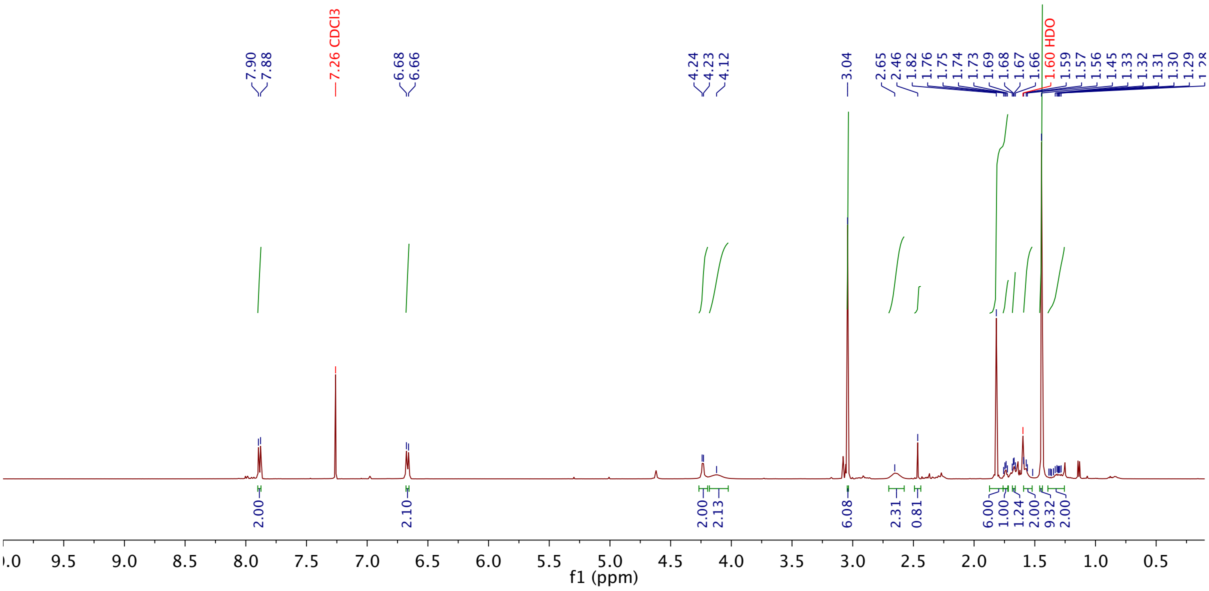

^{13}C NMR (126 MHz, $CDCl_3$)

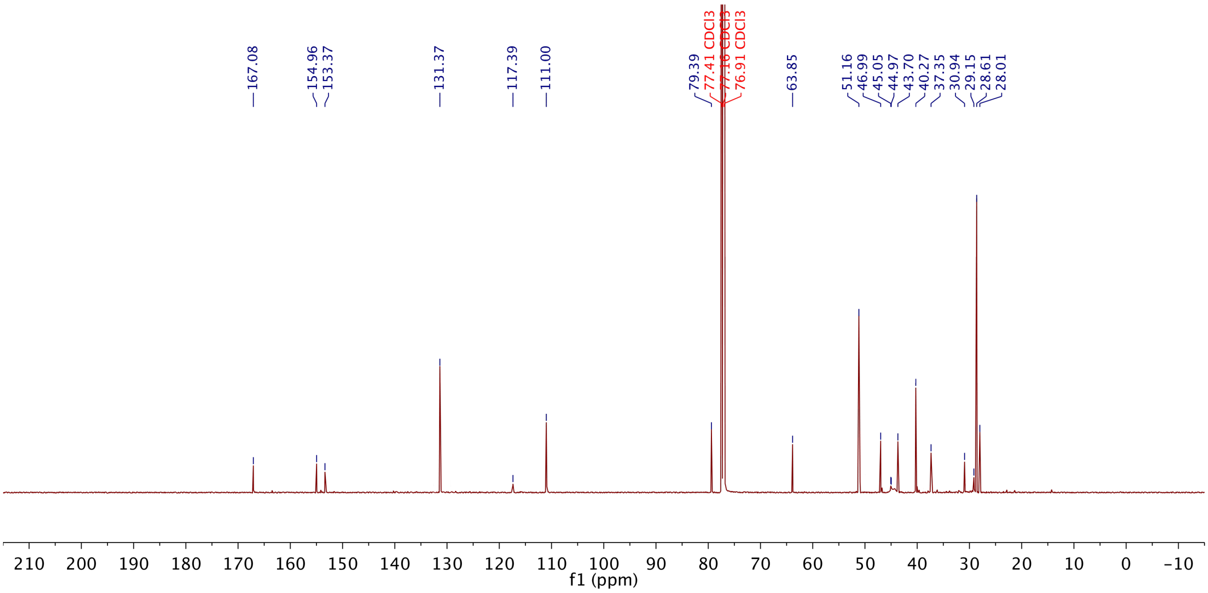

2-(Bicyclo[1.1.1]pentan-1-yl)-4-(5,5-dimethyl-1,3-dioxan-2-yl)butyl 4-(dimethylamino)-benzoate, S50

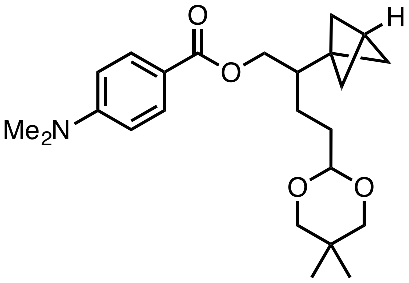

^{1}H NMR (500 MHz, $CDCl_3$)

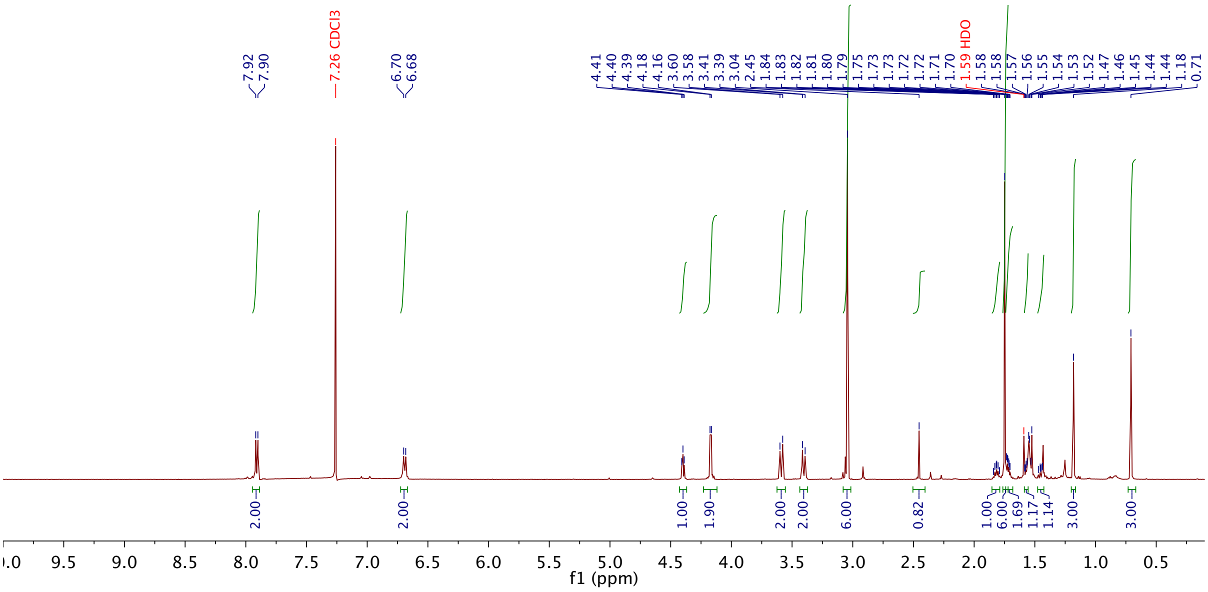

^{13}C NMR (126 MHz, $CDCl_3$)

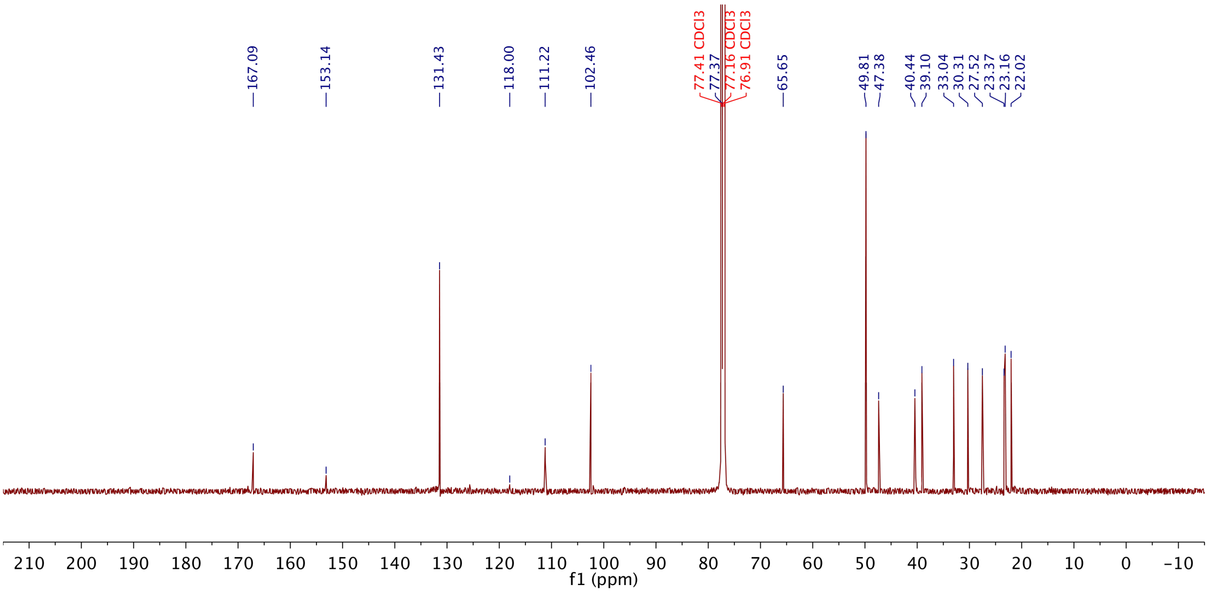

***tert*-Butyl (*S*)-2-(bis(3,5-bis(trifluoromethyl)phenyl)(hydroxy)methyl)-4,4-difluoropyrrolidine-1-carboxylate, S70**

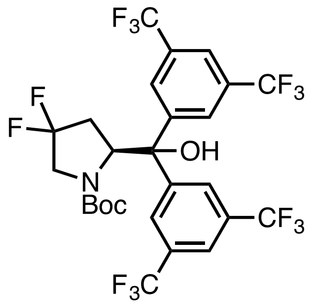

^{1}H NMR (400 MHz, $CDCl_3$)

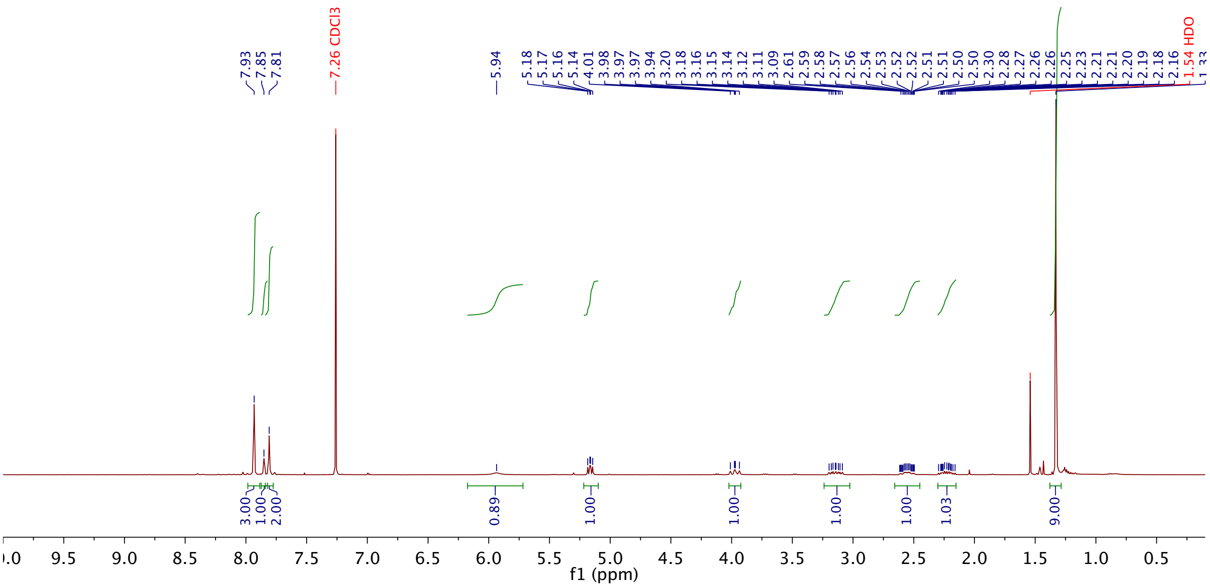

^{13}C NMR (126 MHz, $CDCl_3$)

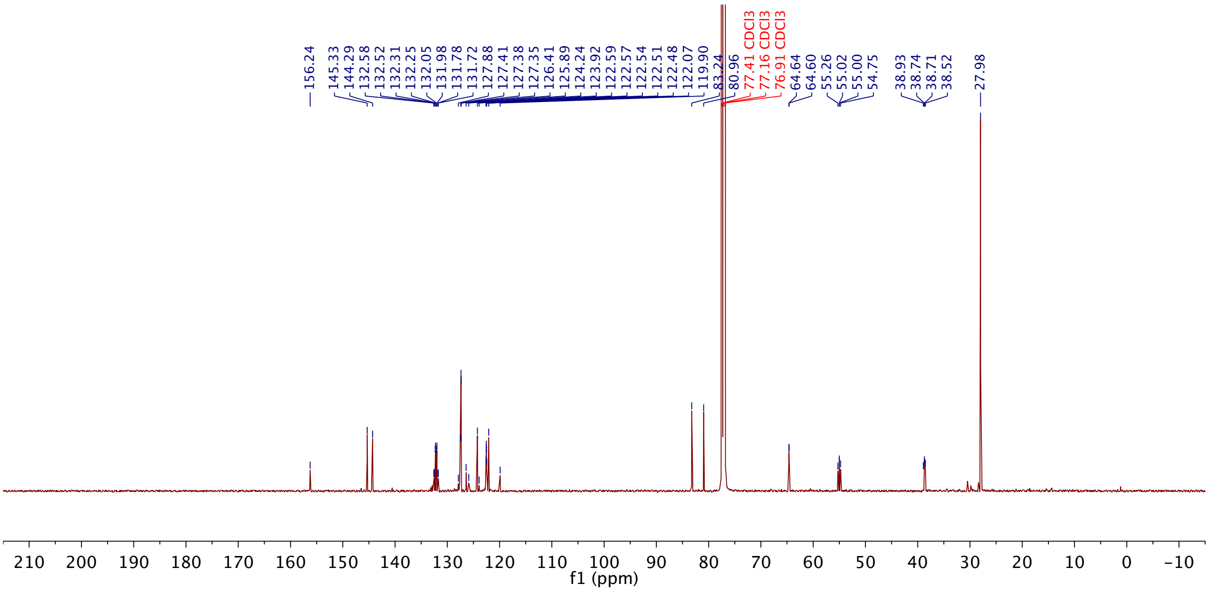

^{19}F NMR (471 MHz, $CDCl_3$)

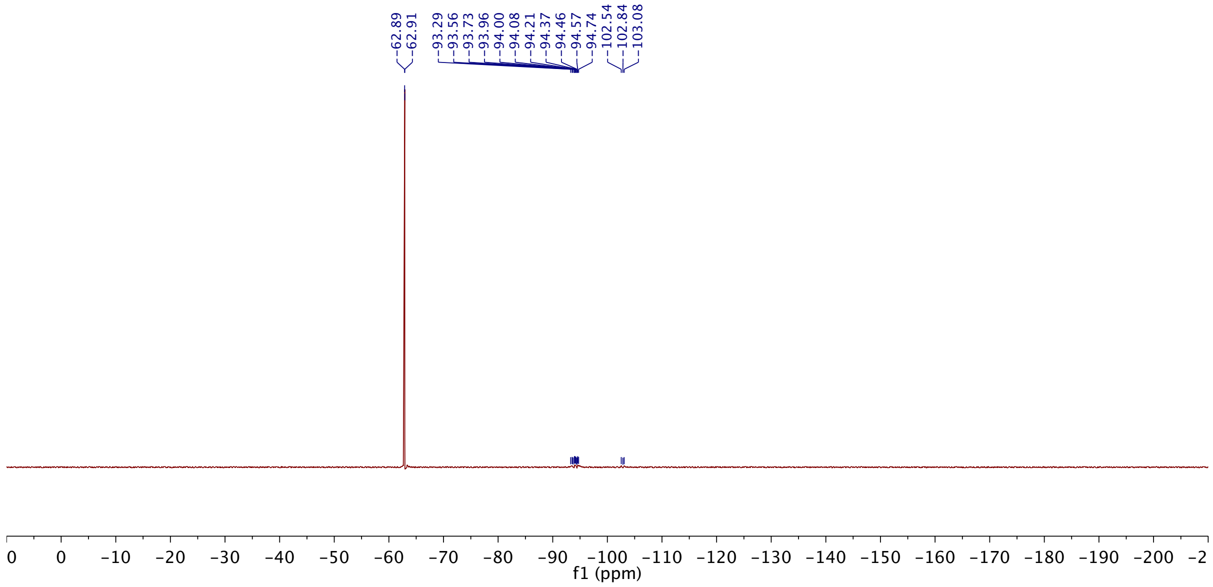

(*S*)-Bis(3,5-bis(trifluoromethyl)phenyl)(4,4-difluoropyrrolidin-2-yl)methanol, S71

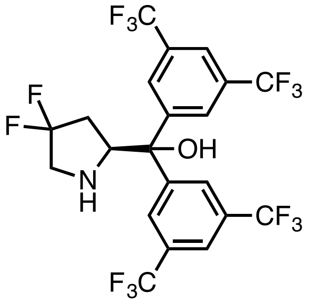

^{1}H NMR (400 MHz, $CDCl_3$)

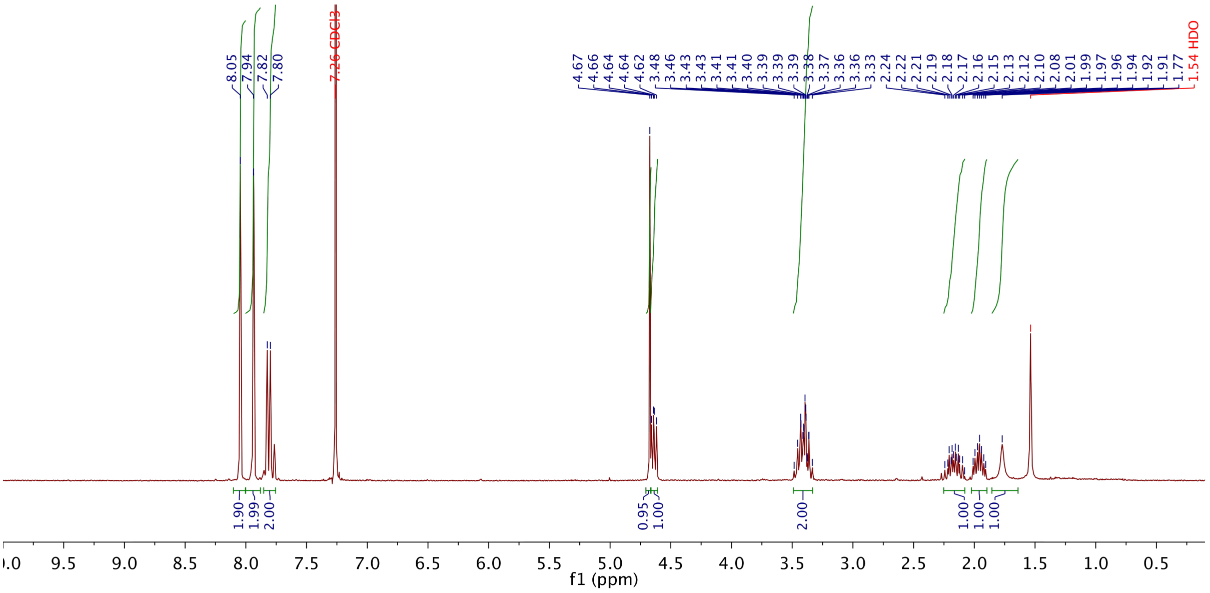

^{13}C NMR (126 MHz, $CDCl_3$)

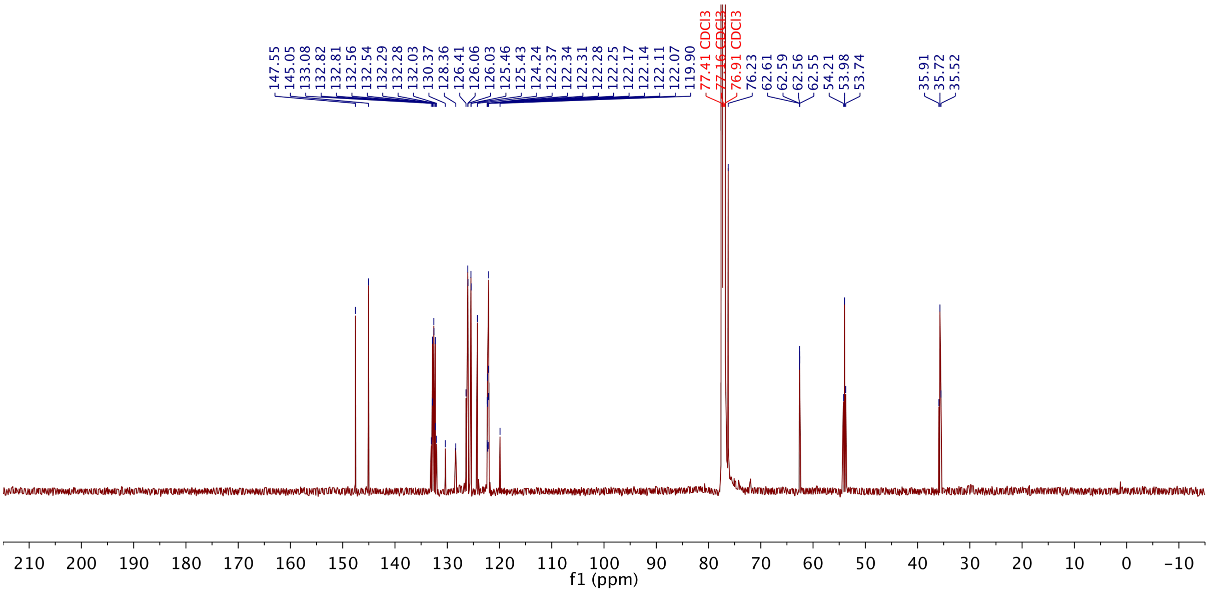

^{19}F NMR (471 MHz, $CDCl_3$)

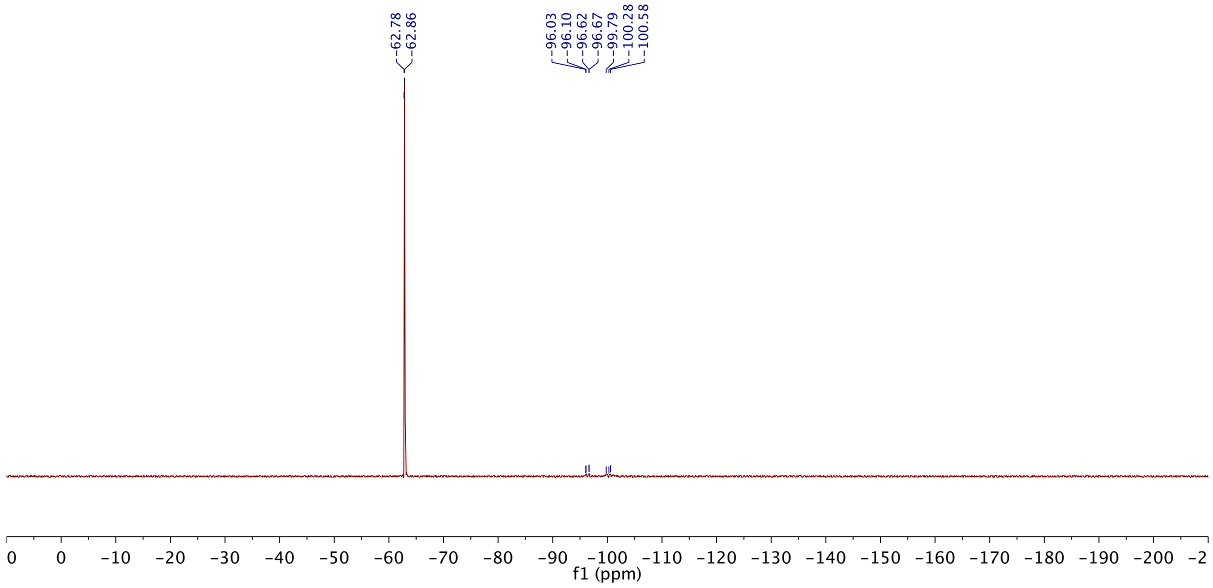

7. Supplementary references

1 Bär, R. M., Kirschner, S., Nieger, M. & Bräse, S. Alkyl and Aryl Thiol Addition to [1.1.1]Propellane: Scope and Limitations of a Fast Conjugation Reaction. *Chem. Eur. J.* **24**, 1373–1382, (2018).

2 Li, Y., Wang, D., Zhang, L. & Luo, S. Redox Property of Enamines. *J. Org. Chem.* **84**, 12071–12090, (2019).

3 Lowry, M. S. *et al.* Single-Layer Electroluminescent Devices and Photoinduced Hydrogen Production from an Ionic Iridium(III) Complex. *Chem. Mater.* **17**, 5712–5719, (2005).

4 Teegardin, K., Day, J. I., Chan, J. & Weaver, J. Advances in Photocatalysis: A Microreview of Visible Light Mediated Ruthenium and Iridium Catalyzed Organic Transformations. *Org. Process Res. Dev.* **20**, 1156–1163, (2016).

5 Choi, G. J., Zhu, Q., Miller, D. C., Gu, C. J. & Knowles, R. R. Catalytic alkylation of remote C–H bonds enabled by proton-coupled electron transfer. *Nature* **539**, 268–271, (2016).

6 Romero, N. A. & Nicewicz, D. A. Organic Photoredox Catalysis. *Chem. Rev.* **116**, 10075–10166, (2016).

7 Shang, T.-Y. *et al.* Recent advances of 1,2,3,5-tetrakis(carbazol-9-yl)-4,6-dicyanobenzene (4CzIPN) in photocatalytic transformations. *Chem. Commun.* **55**, 5408–5419, (2019).

8 Roger, M. *et al.* U(SMes*)n, (n = 3, 4) and Ln(SMes*)3 (Ln = La, Ce, Pr, Nd): Lanthanide(III)/Actinide(III) Differentiation in Agostic Interactions and an Unprecedented η3 Ligation Mode of the Arylthiolate Ligand, from X-ray Diffraction and DFT Analysis. *J. Am. Chem. Soc.* **128**, 8790–8802, (2006).

9 Li, J., Lear, M. J. & Hayashi, Y. Sterically Demanding Oxidative Amidation of α-Substituted Malononitriles with Amines Using O2. *Angew. Chem. Int. Ed.* **55**, 9060–9064, (2016).

10 Nugent, J. *et al.* A General Route to Bicyclo[1.1.1]pentanes through Photoredox Catalysis. *ACS Catal.* **9**, 9568–9574, (2019).

11 Gianatassio, R. *et al.* Strain-release amination. *Science* **351**, 241–246, (2016).

12 Bunker, K. D., Sach, N. W., Huang, Q. & Richardson, P. F. Scalable Synthesis of 1-Bicyclo[1.1.1]pentylamine via a Hydrohydrazination Reaction. *Org. Lett.* **13**, 4746–4748, (2011).

13 Wong, M. L. J., Mousseau, J. J., Mansfield, S. J. & Anderson, E. A. Synthesis of Enantioenriched α-Chiral Bicyclo[1.1.1]pentanes. *Org. Lett.* **21**, 2408–2411, (2019).

14 Xiong, F. *et al.* A bioinspired and biocompatible ortho-sulfiliminyl phenol synthesis. *Nat. Commun.* **8**, 15912, (2017).

15 Saravanan, P. & Anbarasan, P. Palladium Catalyzed Aryl(alkyl)thiolation of Unactivated Arenes. *Org. Lett.* **16**, 848–851, (2014).

16 Salvi, L., Davis, N. R., Ali, S. Z. & Buchwald, S. L. A New Biarylphosphine Ligand for the Pd-Catalyzed Synthesis of Diaryl Ethers under Mild Conditions. *Org. Lett.* **14**, 170–173, (2012).

17 Zhu, Q., Graff, D. E. & Knowles, R. R. Intermolecular Anti-Markovnikov Hydroamination of Unactivated Alkenes with Sulfonamides Enabled by Proton-Coupled Electron Transfer. *J. Am. Chem. Soc.* **140**, 741–747, (2018).

18 Renard, M. & Ghosez, L. A. A convergent asymmetric synthesis of γ-butenolides. *Tetrahedron* **57**, 2597–2608, (2001).

19 Ramsay, W. J. *et al.* Au(I)Cl-bound N-heterocyclic carbene ligands form MII4(LAuCl)6 integrally gilded cages. *Chem. Sci.* **6**, 7326–7331, (2015).

20 Sasaki, S. *et al.* Synthesis of crowded triarylphosphines carrying functional sites. *J. Organomet. Chem* **690**, 2664–2672, (2005).

21 Wolter, M., Nordmann, G., Job, G. E. & Buchwald, S. L. Copper-Catalyzed Coupling of Aryl Iodides with Aliphatic Alcohols. *Org. Lett.* **4**, 973–976, (2002).

22 Olsen, E. P. K., Arrechea, P. L. & Buchwald, S. L. Mechanistic Insight Leads to a Ligand Which Facilitates the Palladium-Catalyzed Formation of 2-(Hetero)Arylaminooxazoles and 4-(Hetero)Arylaminothiazoles. *Angew. Chem. Int. Ed.* **56**, 10569–10572, (2017).

23 Koley, D., Colón, O. C. & Savinov, S. N. Chemoselective Nitration of Phenols with tert-Butyl Nitrite in Solution and on Solid Support. *Org. Lett.* **11**, 4172–4175, (2009).

24 Juárez-Ornelas, K. A., Jiménez-Halla, J. O. C., Kato, T., Solorio-Alvarado, C. R. & Maruoka, K. Iodine(III)-Catalyzed Electrophilic Nitration of Phenols via Non-Bronsted Acidic NO2(+) Generation. *Org. Lett.* **21**, 1315–1319, (2019).

25 Tanaka, H., Sakai, K., Kawamura, A., Oisaki, K. & Kanai, M. Sulfonamides as new hydrogen atom transfer (HAT) catalysts for photoredox allylic and benzylic C-H arylations. *Chem. Commun.* **54**, 3215–3218, (2018).

26 Puente, Á., Ofial, A. R. & Mayr, H. Nucleophilic Reactivities of Bis-Acceptor-Substituted Benzyl Anions. *Eur. J. Org. Chem.*, 1196–1202, (2017).

27 Peterson, J. P., Geraskina, M. R., Zhang, R. & Winter, A. H. Effect of Substituents on the Bond Strength of Air-Stable Dicyanomethyl Radical Thermochromes. *J. Org. Chem.* **82**, 6497–6501, (2017).

28 Hitomi, S., Hideki, K. & Takuji, O. Anomalous Reaction of Arylmalononitriles with Nitric Acid. Para-Directing Nature of Dicyanomethyl Group and a Through-Ring

Nitro/aci-Nitro Tautomerism of 4-Nitrophenylmalononitrile. *Bull. Chem. Soc. Jpn.* **61**, 501–504, (1988).

29 Atkins, J. M., Moteki, S. A., DiMagno, S. G. & Takacs, J. M. Single Enantiomer, Chiral Donor-Acceptor Metal Complexes from Bisoxazoline Pseudoracemates. *Org. Lett.* **8**, 2759–2762, (2006).

30 Itoi, H. Amine compound and organic electroluminescence device including the same. US20180287062A1 (2018).

31 Zhao, Z. *et al.* Stereoselective synthesis of folded luminogens with arene-arene stacking interactions and aggregation-enhanced emission. *Chem. Commun.* **50**, 1131–1133, (2014).

32 Liu, Z. *et al.* Transition-Metal-Free Intramolecular Carbene Aromatic Substitution/Büchner Reaction: Synthesis of Fluorenes and [6,5,7]Benzo-fused Rings. *Angew. Chem. Int. Ed.* **54**, 3056–3060, (2015).

33 Zhou, J., He, J., Wang, B., Yang, W. & Ren, H. 1,7-Palladium Migration via C-H Activation, Followed by Intramolecular Amination: Regioselective Synthesis of Benzotriazoles. *J. Am. Chem. Soc.* **133**, 6868–6870, (2011).

34 Liao, W. *et al.* 1,2,3-Triazole-boranes: stable and efficient reagents for ketone and aldehyde reductive amination in organic solvents or in water. *Chem. Commun.*, 6436–6438, (2009).

35 Lin, Q. *et al.* Enantioselective Synthesis of Janus Kinase Inhibitor INCB018424 via an Organocatalytic Aza-Michael Reaction. *Org. Lett.* **11**, 1999–2002, (2009).

36 Haraguchi, R. & Matsubara, S. Catalytic Asymmetric Aldol-Type Reaction of Zinc Enolate Equivalent of Amides. *Org. Lett.* **15**, 3378–3380, (2013).

37 Manzano, R., Datta, S., Paton, R. S. & Dixon, D. J. Enantioselective Silver and Amine Co-catalyzed Desymmetrizing Cycloisomerization of Alkyne-Linked Cyclohexanones. *Angew. Chem. Int. Ed.* **56**, 5834–5838, (2017).

38 Dalicsek, Z., Pollreisz, F. & Soós, T. Efficient separation of a trifluoromethyl substituted organocatalyst: just add water. *Chem. Commun.*, 4587–4589, (2009).

39 Gotoh, H. & Hayashi, Y. Diarylprolinol Silyl Ether as Catalyst of an exo-Selective, Enantioselective Diels-Alder Reaction. *Org. Lett.* **9**, 2859–2862, (2007).

40 Murar, C. E., Thuaud, F. & Bode, J. W. KAHA Ligations That Form Aspartyl Aldehyde Residues as Synthetic Handles for Protein Modification and Purification. *J. Am. Chem. Soc.* **136**, 18140–18148, (2014).

41 Dálaigh, C. Ó. & Connon, S. J. Nonenzymatic Acylative Kinetic Resolution of Baylis-Hillman Adducts. *J. Org. Chem.* **72**, 7066–7069, (2007).

42 Claraz, A. *et al.* A Catalyst Designed for the Enantioselective Construction of Methyl- and Alkyl-Substituted Tertiary Stereocenters. *Angew. Chem. Int. Ed.* **55**, 669–673, (2016).

43 Marigo, M., Wabnitz, T. C., Fielenbach, D. & Jørgensen, K. A. Enantioselective Organocatalyzed α-Sulfenylation of Aldehydes. *Angew. Chem. Int. Ed.* **44**, 794–797, (2005).

44 Novacek, J., Roiser, L., Zielke, K., Robiette, R. & Waser, M. Towards a General Understanding of Carbonyl-Stabilised Ammonium Ylide-Mediated Epoxidation Reactions. *Chem. Eur. J.* **22**, 11422–11428, (2016).

45 Kemppainen, E. K., Sahoo, G., Valkonen, A. & Pihko, P. M. Mukaiyama-Michael Reactions with Acrolein and Methacrolein: A Catalytic Enantioselective Synthesis of the C17-C28 Fragment of Pectenotoxins. *Org. Lett.* **14**, 1086–1089, (2012).

46 Burnett, D. A., Bursavich, M. G. & Mcriner, A. J. Fused morpholinopyrimidines and methods of use thereof. WO2015109109A1 (2015).

47 Mahender Reddy, K. *et al.* Cationic Chiral Fluorinated Oxazaborolidines. More Potent, Second-Generation Catalysts for Highly Enantioselective Cycloaddition Reactions. *J. Am. Chem. Soc.* **138**, 2443–2453, (2016).

48 Martínez, J. I., Reyes, E., Uria, U., Carrillo, L. & Vicario, J. L. Optimizing the Structure of 4-Dialkylamino-α,α-diarylprolinol Ethers as Catalysts for the Enantioselective Cyclopropanation of α,β-Unsaturated Aldehydes in Water. *ChemCatChem* **5**, 2240–2247, (2013).

49 Mengozzi, L., Gualandi, A. & Cozzi, P. G. Organocatalytic Stereoselective Addition of Aldehydes to Acylquinolinium Ions. *Eur. J. Org. Chem.* **2016**, 3200–3207, (2016).

50 Jui, N. T., Garber, J. A., Finelli, F. G. & MacMillan, D. W. C. Enantioselective Organo-SOMO Cycloadditions: A Catalytic Approach to Complex Pyrrolidines from Olefins and Aldehydes. *J. Am. Chem. Soc.* **134**, 11400–11403, (2012).

51 Ren, W. *et al.* An Effective Pd-Catalyzed Regioselective Hydroformylation of Olefins with Formic Acid. *J. Am. Chem. Soc.* **138**, 14864–14867, (2016).

52 Huang, H. *et al.* Chemo- and Regioselective Organo-Photoredox Catalyzed Hydroformylation of Styrenes via a Radical Pathway. *J. Am. Chem. Soc.* **139**, 9799–9802, (2017).

53 Costello, J. P. & Ferreira, E. M. Regioselectivity Influences in Platinum-Catalyzed Intramolecular Alkyne O-H and N-H Additions. *Org. Lett.* **21**, 9934–9939, (2019).

54 Holt, C., Alachouzos, G. & Frontier, A. J. Leveraging the Halo-Nazarov Cyclization for the Chemodivergent Assembly of Functionalized Haloindenes and Indanones. *J. Am. Chem. Soc.* **141**, 5461–5469, (2019).

55 Colombo, D. *et al.* Chemoselective Biohydrogenation of Alkenes in the Presence of Alkynes for the Homologation of 2-Alkynals/3-Alkyn-2-ones into 4-Alkynals/Alkynols. *Adv. Synth. Catal.* **361**, 2638–2648, (2019).

56 Xu, B., Lumb, J.-P. & Arndtsen, B. A. A TEMPO-Free Copper-Catalyzed Aerobic Oxidation of Alcohols. *Angew. Chem. Int. Ed.* **54**, 4208–4211, (2015).

57 Uyanik, M., Suzuki, D., Yasui, T. & Ishihara, K. In Situ Generated (Hypo)Iodite Catalysts for the Direct α-Oxyacylation of Carbonyl Compounds with Carboxylic Acids. *Angew. Chem. Int. Ed.* **50**, 5331–5334, (2011).

58 Spallarossa, M., Wang, Q., Riva, R. & Zhu, J. Synthesis of Vinyl Isocyanides and Development of a Convertible Isonitrile. *Org. Lett.* **18**, 1622–1625, (2016).

59 Amatore, M., Beeson, T. D., Brown, S. P. & MacMillan, D. W. C. Enantioselective Linchpin Catalysis by SOMO Catalysis: An Approach to the Asymmetric α-Chlorination of Aldehydes and Terminal Epoxide Formation. *Angew. Chem. Int. Ed.* **48**, 5121–5124, (2009).

60 Shang, Y., Jie, X., Jonnada, K., Zafar, S. N. & Su, W. Dehydrogenative desaturation-relay via formation of multicenter-stabilized radical intermediates. *Nat. Commun.* **8**, 2273, (2017).

61 Neese, F. Software update: the ORCA program system, version 4.0. *WIREs Comput. Mol. Sci.* **8**, e1327, (2018).

62 Weigend, F. & Ahlrichs, R. Balanced basis sets of split valence, triple zeta valence and quadruple zeta valence quality for H to Rn: Design and assessment of accuracy. *Phys. Chem. Chem. Phys.* **7**, 3297–3305, (2005).

63 National Center for Biotechnology Information. *PubChem Compound Summary for CID 8071, 1,2-Dimethoxyethane,* https://pubchem.ncbi.nlm.nih.gov/compound/1_2-Dimethoxyethane. Accessed 26 Jun 2020

64 Marenich, A. V., Cramer, C. J. & Truhlar, D. G. Universal Solvation Model Based on Solute Electron Density and on a Continuum Model of the Solvent Defined by the Bulk Dielectric Constant and Atomic Surface Tensions. *J. Phys. Chem. B* **113**, 6378–6396, (2009).

65 Grimme, S., Antony, J., Ehrlich, S. & Krieg, H. A consistent and accurate ab initio parametrization of density functional dispersion correction (DFT-D) for the 94 elements H-Pu. *J. Chem. Phys.* **132**, 154104, (2010).

66 Karton, A., Tarnopolsky, A., Lamère, J.-F., Schatz, G. C. & Martin, J. M. L. Highly Accurate First-Principles Benchmark Data Sets for the Parametrization and Validation of Density Functional and Other Approximate Methods. Derivation of a Robust, Generally Applicable, Double-Hybrid Functional for Thermochemistry and Thermochemical Kinetics. *J. Phys. Chem. A* **112**, 12868–12886, (2008).

67 Sterling, A. J., Dürr, A. B., Smith, R. C., Anderson, E. A. & Duarte, F. Rationalizing the diverse reactivity of [1.1.1]propellane through σ–π-delocalization. *Chem. Sci.* **11**, 4895–4903, (2020).

68 Riplinger, C. & Neese, F. An efficient and near linear scaling pair natural orbital based local coupled cluster method. *J. Chem. Phys.* **138**, 034106, (2013).

69 Grimme, S. Supramolecular Binding Thermodynamics by Dispersion-Corrected Density Functional Theory. *Chem. Eur. J.* **18**, 9955–9964, (2012).

70 Tom Young (10 Jul 2019). duartegroup/otherm: Initial release (Version v1.0.0-alpha). Zenodo. http://doi.org/10.5281/zenodo.3294011.

71 Bannwarth, C., Ehlert, S. & Grimme, S. GFN2-xTB-An Accurate and Broadly Parametrized Self-Consistent Tight-Binding Quantum Chemical Method with Multipole Electrostatics and Density-Dependent Dispersion Contributions. *J. Chem. Theory Comput.* **15**, 1652–1671, (2019).

72 Tom Young (10 Jul 2019). duartegroup/autodE: Initial release (Version v1.0.0-alpha). Zenodo. http://doi.org/10.5281/zenodo.3294219.

73 Bickelhaupt, F. M. & Houk, K. N. Analyzing Reaction Rates with the Distortion/Interaction-Activation Strain Model. *Angew. Chem. Int. Ed.* **56**, 10070–10086, (2017).

74 Lu, T. & Chen, F. Multiwfn: A multifunctional wavefunction analyzer. *J. Comput. Chem.* **33**, 580–592, (2012).
